# RNA interference targeting ANGPTL3 for triglyceride and cholesterol lowering: phase 1 basket trial cohorts

**DOI:** 10.1038/s41591-023-02494-2

**Published:** 2023-08-25

**Authors:** Gerald F. Watts, Christian Schwabe, Russell Scott, Patrick A. Gladding, David Sullivan, John Baker, Peter Clifton, James Hamilton, Bruce Given, Stacey Melquist, Rong Zhou, Ting Chang, Javier San Martin, Daniel Gaudet, Ira J. Goldberg, Joshua W. Knowles, Robert A. Hegele, Christie M. Ballantyne

**Affiliations:** 1 School of Medicine, University of Western Australia, Perth, Western Australia Australia; 2 Auckland Clinical Studies, Auckland, New Zealand; 3 New Zealand Clinical Research Christchurch, Christchurch, New Zealand; 4 Te Whatu Ora Waitematā, Auckland, New Zealand; 5 Royal Prince Alfred Hospital, Sydney, New South Wales Australia; 6 Middlemore Hospital, Auckland, New Zealand; 7 Royal Adelaide Hospital, Adelaide, South Australia Australia; 8 Arrowhead Pharmaceuticals, Inc., Pasadena, CA USA; 9 Department of Medicine, Université de Montréal and ECOGENE 21 Clinical Research Center, Chicoutimi, Quebec Canada; 10 NYU School of Medicine, NYU Langone Health, New York City, NY USA; 11 Stanford Division of Cardiovascular Medicine and Cardiovascular Institute, School of Medicine, Stanford, CA USA; 12 University of Western Ontario, London, Ontario Canada; 13 Baylor College of Medicine, Houston, TX USA

**Keywords:** Drug development, RNAi therapy, Drug delivery, Dyslipidaemias

## Abstract

Elevated triglycerides and non-high-density lipoprotein cholesterol (HDL-C) are risk factors for atherosclerotic cardiovascular disease (ASCVD). ARO-ANG3 is an RNA interference therapy that targets angiopoietin-like protein 3 (ANGPTL3), a regulator of lipoprotein metabolism. This first-in-human, phase 1, randomized, placebo-controlled, open-label trial investigated single and repeat ARO-ANG3 doses in four cohorts of fifty-two healthy participants and one cohort of nine participants with hepatic steatosis, part of a basket trial. Safety (primary objective) and pharmacokinetics (in healthy participants) and pharmacodynamics (secondary objectives) of ARO-ANG3 were evaluated. ARO-ANG3 was generally well tolerated, with similar frequencies of treatment-emergent adverse events in active and placebo groups. Systemic absorption of ARO-ANG3 in healthy participants was rapid and sustained, with a mean *T*_max_ of 6.0–10.5 h and clearance from plasma within 24–48 h after dosing with a mean *t*_½_ of 3.9–6.6 h. In healthy participants, ARO-ANG3 treatment reduced ANGPTL3 (mean −45% to −78%) 85 days after dose. Reductions in triglyceride (median −34% to −54%) and non-HDL-C (mean −18% to −29%) (exploratory endpoints) concentrations occurred with the three highest doses. These early-phase data support ANGPTL3 as a potential therapeutic target for ASCVD treatment. ClinicalTrials.gov identifier: NCT03747224

## Abstract (web summary)

First-in-human results from five cohorts of healthy volunteers and individuals with hepatic steatosis show that RNA interference treatment targeting ANGPTL3 was well tolerated and led to reduction in triglycerides and non-HDL cholesterol.

## Main

Atherosclerotic cardiovascular disease (ASCVD) is the leading cause of mortality worldwide^1^. Mixed dyslipidemia, with elevated triglycerides (TGs) and non-high-density lipoprotein cholesterol (non-HDL-C) (including low-density lipoprotein cholesterol (LDL-C)), is associated with increased risk of ASCVD. Although statins and other therapies can lower LDL-C, substantial residual risk of ASCVD remains due to additional independent risk factors (for example, TG-rich lipoproteins (TRLs)) and limited response in LDL-C with available therapies^2,3^.

Angiopoietin-like protein 3 (ANGPTL3), a member of the angiopoietin-like family of proteins, is a hepatokine exclusively secreted by the liver and a key regulator of serum lipid and lipoprotein metabolism. ANGPTL3 inhibits lipoprotein lipase (LPL), thereby regulating the intravascular clearance of TG. It also inhibits endothelial lipase, which is involved in the catabolism of HDL-C and very-low-density lipoprotein cholesterol (VLDL-C)^4,5^. ANGPTL3 may also control the production and clearance of LDL-C^6,7^.

In humans, homozygous loss-of-function mutations in *ANGPTL3* cause familial combined hypolipidemia, characterized by low concentrations of TG, LDL-C and HDL-C^6,8^. Heterozygote carriers have a 34–39% lower risk of coronary artery disease than non-carriers^9,10^ and no apparent adverse clinical phenotype^9^. *ANGPTL3* loss-of-function variants appear protective against ASCVD despite lowering of HDL-C^10^.

Pharmacological inhibition of ANGPTL3 with evinacumab, a monoclonal antibody, and with vupanorsen, an antisense oligonucleotide (ASO), have been shown to replicate the phenotype of *ANGPTL3* loss-of-function carriers^11,12^. Studies with evinacumab targeting circulating ANGPTL3 in healthy paticipants (HPs)^9^ and in individuals with familial hypercholesterolemia^11,13^ have shown potent reductions in LDL-C, HDL-C and TG. Importantly, the LDL-C-lowering effect is independent of an intact LDL receptor (LDLR), as supported by evinacumab’s efficacy in an LDLR-deficient population^13^. However, an antibody approach requires monthly or more frequent intravenous administration, which may be inconvenient for patients and could impact adherence to treatment. Furthermore, the development of vupanorsen has been discontinued because of increases in alanine transaminase (ALT) and hepatic steatosis^12,14^.

ARO-ANG3 is a subcutaneously administered, synthetic, double-stranded small interfering RNA (siRNA) molecule targeted to hepatocytes (via conjugation to *N*-acetylgalactosamine (NAG)) that degrades *ANGPTL3* mRNA within the cytoplasm^14,15^. A hepatocyte-targeted siRNA approach to lipid lowering is feasible based on the recent US Food and Drug Administration (FDA) and European Medicines Agency (EMA) approval of inclisiran, which silences hepatic expression of PCSK9 (https://www.accessdata.fda.gov/drugsatfda_docs/label/2021/214012lbl.pdf and https://www.ema.europa.eu/en/documents/product-information/leqvio-epar-product-information_en.pdf). Animal studies show that ARO-ANG3 is highly effective in inhibiting both the *ANGPTL3* mRNA transcript and the hepatic production of the ANGPTL3 protein, with corresponding reductions in TG, LDL-C and HDL-C^16^. In humans, this effect may lower the risk of ASCVD, particularly in patients lacking sufficient LDLR activity (for example, homozygous familial hypercholesterolemia) or patients with mixed dyslipidemia who have persistently high TG and LDL-C despite current lipid-lowering therapies.

**Extended Data Fig. 1:**
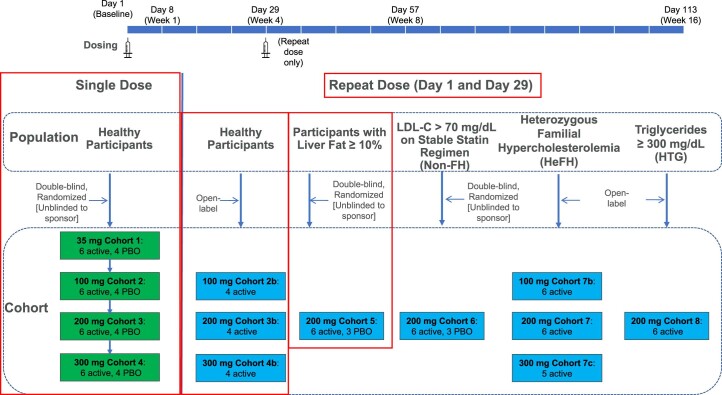
Study Schema. This schema reflects the basket trial approach taken in this Phase 1 study AROANG1001 to evaluate early proof of-concept, in which healthy participants (HPs) and patients with various dyslipidemias were enrolled. In this report, we present the key results from this study showing the safety, tolerability, and pharmacodynamic effects of single ascending doses of ARO-ANG3 in HPs and multiple doses of ARO-ANG3 in both HPs and a cohort of patients with baseline hepatic steatosis. These cohorts are shown in red boxes.

In this phase 1 study (AROANG1001), a basket trial design was adopted to initially evaluate therapeutic proof of concept in HPs and participants with various dyslipidemias (Extended Data Fig. 1). Here we report key results describing the safety, tolerability and pharmacodynamic effects of single and multiple ascending doses of ARO-ANG3 in HPs. Because ASO therapy targeting ANGPTL3 has been associated with increases in aminotransferases and liver fat^17^, we also report findings from a repeat-dose cohort evaluating ARO-ANG3 in participants with baseline hepatic steatosis.

## Results

### Participant characteristics and dosing

In the HP single ascending dose (SAD) cohorts, 24 participants received subcutaneously administered ARO-ANG3 (35 mg, 100 mg, 200 mg or 300 mg), and 16 participants received placebo on day 1 (cohorts 1–4; Supplementary Table 1). In the HP multiple ascending dose (MAD) cohorts, 12 participants received ARO-ANG3 100 mg, 200 mg or 300 mg on days 1 and 29. In the hepatic steatosis cohort, six participants received repeat doses (200 mg) of ARO-ANG3 and three participants received placebo on days 1 and 29. Participants in this cohort were required to have baseline hepatic steatosis defined as a liver fat content of ≥10%, measured as the magnetic resonance imaging-estimated proton density fat fraction (MRI-PDFF).

Demographics and baseline characteristics for the HP SAD cohorts (*n* = 40) and the HP MAD cohort (*n* = 12) are described in Table 1. For the hepatic steatosis cohort, demographics and baseline characteristics are shown in Table 2.

**Table 1:** Demographic and baseline metabolic characteristics of the HPs in the SAD and MAD cohorts

	(A) Single dose (day 1) HPs					(B) Repeat dose (days 1 and 29)		
	Pooled placebo	ARO-ANG3 35 mg	ARO-ANG3 100 mg	ARO-ANG3 200 mg	ARO-ANG3 300 mg	ARO-ANG3 100 mg	ARO-ANG3 200 mg	ARO-ANG3 300 mg
Characteristic	*n* = 16	*n* = 6	*n* = 6	*n* = 6	*n* = 6	*n* = 4	*n* = 4	*n* = 4
Age^a^ (years)	39.5 (22, 61)	37.5 (19, 58)	51.0 (43, 61)	42.5 (32, 56)	50.0 (36, 64)	53 (20, 62)	44.5 (29, 55)	29.5 (22, 42)
Male sex^b^ – no. (%)	12 (75.0%)	4 (66.7%)	3 (50.0%)	5 (83.3%)	5 (83.3%)	2 (50.0%)	4 (100.0%)	2 (50.0%)
Female sex^b^ – no. (%)	4 (25.0%)	2 (33.3%)	3 (50.0%)	1 (16.7%)	1 (16.7%)	2 (50.0%)	0	2 (50.0%)
Race^b,c^ – no. (%)								
White	11 (68.8%)	5 (83.3%)	5 (83.3%)	3 (50.0%)	5 (83.3%)	3 (75.0%)	1 (25.0%)	2 (50.0%)
Asian	2 (12.5%)	1 (16.7%)	0	1 (16.7%)	1 (16.7%)	1 (25.0%)	1 (25.0%)	0
Native Hawaiian or Other Pacific Islander	1 (6.3%)	0	1 (16.7)	2 (33.3%)	0	0	1 (25.0%)	0
Other	2 (12.5%)	0	0	0	0	0	1 (25.0%)	2 (50.0%)
BMI^d^ (kg m^−^^2^)	28.01 (3.92)	29.52 (2.85)	29.75 (5.07)	29.90 (3.95)	26.25 (3.70)	25.13 (2.69)	25.28 (2.72)	32.03 (7.08)
ANGPTL3^d^ (μg l^−1^)	81.13 (18.57)	88.23 (17.94)	94.93 (36.01)	90.57 (26.97)	82.87 (23.73)	98.75 (9.07)	107.08 (12.75)	91.35 (16.63)
TG^a^ (mg dl^−1^)	110.00 (54, 727)	92.5 (79, 205)	147.0 (87, 292)	173.0 (117, 350)	133.5 (91, 369)	157.5 (72, 204)	144.0 (112, 164)	134.0 (57, 324)
Non-HDL-C^d^ (mg dl^−1^)	155.0 (31.5)	169.0 (49.0)	197.7 (45.7)	183.2 (50.5)	173.3 (35.0)	196.8 (44.0)	163.5 (18.9)	151.3 (35.4)
VLDL-C^d^ (mg dl^−1^)	24.0 (11.6)	22.3 (9.4)	32.3 (15.3)	42.5 (19.2)	33.8 (20.4)	29.5 (12.1)	28.3 (5.6)	32.3 (23.1)
LDL-C^d,e^ (mg dl^−1^)	125.1 (37.0)	146.7 (41.8)	165.3 (36.8)	140.7 (52.2)	139.5 (32.5)	167.3 (32.8)	135.3 (23.0)	119.0 (25.6)
HDL-C^d^ (mg dl^−1^)	43.1 (8.4)	47.8 (8.1)	47.8 (11.5)	41.0 (10.5)	45.5 (13.3)	50.0 (17.7)	39.5 (3.4)	36.8 (12.7)
ApoB^d^ (mg dl^−1^)	95.88 (17.86)	111.63 (28.77)	122.95 (30.63)	114.25 (29.09)	105.82 (17.54)	116.13 (29.60)	96.30 (11.63)	90.68 (21.18)

HPs in the SAD cohorts received placebo or SADs of 35, 100, 200 or 300 mg of ARO-ANG3 (A), and HPs in the MAD cohorts received 100, 200 or 300 mg of ARO-ANG3 (B).

^a^Median (range) values are noted for age and TG.

^b^Sex and race were self-reported.

^c^No participants identified as Black or African American, American Indian or Alaska Native.

^d^Mean ± s.d. was noted for BMI, ANGPTL3, non-HDL-C, VLDL-C, LDL-C, HDL-C and ApoB.

^e^LDL-C was derived using the Friedewald calculation.

Note: To convert the values for TGs to millimoles per liter, multiply by 0.01129. To convert the values for non-HDL-C and HDL-C to millimoles per liter, multiply by 0.02586.

Note: Medpace Reference Laboratories’ reference ranges were used.

**Table 2:** Demographic and baseline metabolic characteristics of participants with hepatic steatosis receiving placebo or a repeat dose of 200 mg of ARO-ANG3

	Hepatic steatosis cohort	
	Placebo	ARO-ANG3 200 mg
Characteristic	*n* = 3	*n* = 6
Age^a^ (years)	45.0 (45, 60)	54.5 (44, 65)
Male sex^b^ – no. (%)	2 (66.7%)	3 (50.0%)
Female sex^b^ – no. (%)	1 (33.3%)	3 (50.0%)
Race^b,c^ – no (%)		
White	2 (66.7%)	2 (33.3%)
Asian	1 (33.3%)	0
Native Hawaiian or Other Pacific Islander	0	2 (33.3%)
Other	0	2 (33.3%)
BMI^d^ (kg m^−^^2^)	31.27 (8.57)	33.67 (4.34)
ANGPTL3^d^ (μg l^−1^)	77.77 (19.96)	138.30 (38.55)
TGs^a^ (mg dl^−1^)	110.0 (102, 350)	105.0 (75, 182)
Non-HDL-C^d^ (mg dl^−1^)	101.3 (26.4)	130.0 (39.3)
VLDL-C^d^ (mg dl^−1^)	37.3 (28.3)	23.3 (7.3)
LDL-C^d,e^ (mg dl^−1^)	64.0 (28.8)	106.7 (37.3)
HDL-C^d^ (mg dl^−1^)	35.3 (2.1)	50.0 (7.9)
MRI-PDFF^d^ (%)	20.4 (5.7)	21.2 (6.7)

^a^Median (range) values are noted for age and TG.

^b^Sex and race were self-reported.

^c^No participants identified as Black or African American, American Indian or Alaska Native.

^d^Mean ± s.d. was noted for BMI, ANGPTL3, non-HDL-C, VLDL-C, LDL-C, HDL-C and MRI-PDFF.

^e^LDL-C was derived using the Friedewald calculation.

Note: To convert the values for TGs to millimoles per liter, multiply by 0.01129. To convert the values for non-HDL-C and HDL-C to millimoles per liter, multiply by 0.02586.

Note: Medpace Reference Laboratories’ reference ranges were used.

### Overall safety

ARO-ANG3 was generally well tolerated when administered subcutaneously as a single dose in HPs and repeat doses in HPs and participants with hepatic steatosi. There were no deaths, life-threatening treatment-emergent adverse events (TEAEs) or TEAEs leading to drug discontinuation or premature withdrawal of any participant from the study. There were no treatment-related serious adverse events (SAEs), and most TEAEs were either mild or moderate.

ARO-ANG3 was not associated with any consistent pattern of adverse changes in laboratory parameters. There were no reported clinically meaningful declines in platelet count. Plots for total bilirubin versus ALT at peak post-dose values and at the end of the study are presented in Supplementary Figs. 1 and 2. Hepatic function and platelet counts that met certain thresholds during the study period are presented in Supplementary Table 2.

#### HP cohorts, SAD

In total, 22 out of 24 (91.7%) HPs who received at least a single dose of ARO-ANG3 reported at least one TEAE, with a total of 65 reported TEAEs (Table 3). There was no apparent dose-dependent increase in the incidence of TEAEs. There were no SAEs reported in participants receiving ARO-ANG3 in the HP SAD cohorts. There was one SAE of pulmonary embolism reported in a participant receiving placebo.

**Table 3:** Overall summary of TEAEs in HP cohorts receiving placebo or 35 mg, 100 mg, 200 mg or 300 mg of ARO-ANG3 (SADs) and cohorts receiving placebo or 100 mg, 200 mg or 300 mg of ARO-ANG3 (MADs)

	HPs (SAD)						HPs (MAD)			
	Pooled placebo (*n* = 16)	Pooled active (*n* = 24)	ARO-ANG3 (35 mg) (*n* = 6)	ARO-ANG3 (100 mg) (*n* = 6)	ARO-ANG3 (200 mg) (*n* = 6)	ARO-ANG3 (300 mg) (*n* = 6)	Pooled Active (*n* = 12)	ARO-ANG3 (100 mg) (*n* = 4)	ARO-ANG3 (200 mg) (*n* = 4)	ARO-ANG3 (300 mg) (*n* = 4)
Number of TEAEs	34	65	12	13	23	17	44	17	6	21
Number of participants reporting at least one:										
TEAE	14 (87.5%)	22 (91.7%)	5 (83.3%)	6 (100%)	6 (100%)	5 (83.3%)	10 (83.3%)	4 (100%)	2 (50.0%)	4 (100%)
Serious TEAE	1 (6.3%)	0	0	0	0	0	0	0	0	0
Severe TEAE	1 (6.3%)	0	0	0	0	0	0	0	0	0
Related TEAE	2 (12.5%)	4 (16.7%)	0	1 (16.7%)	2 (33.3%)	1 (16.7%)	2 (16.7%)	0	0	2 (50.0%)
Related serious TEAE	0	0	0	0	0	0	0	0	0	0
Related severe TEAE	0	0	0	0	0	0	0	0	0	0
TEAEs leading to drug/study withdrawal	0	0	0	0	0	0	0	0	0	0
Number of participants reporting TEAEs by severity:										
Mild	12 (75.0%)	20 (83.3%)	5 (83.3%)	5 (83.3%)	6 (100%)	4 (66.7%)	10 (83.3%)	4 (100%)	2 (50.0%)	4 (100%)
Moderate	5 (31.3%)	5 (20.8%)	1 (16.7%)	1 (16.7%)	1 (16.7%)	2 (33.3%)	0	0	0	0
Severe	1 (6.3%)	0	0	0	0	0	0	0	0	0
Number of participants reporting TEAEs by relationship to study treatment:										
Not related	14 (87.5%)	22 (91.7%)	5 (83.3%)	6 (100%)	6 (100%)	5 (83.3%)	10 (83.3%)	4 (100%)	2 (50.0%)	4 (100%)
Possibly	2 (12.5%)	3 (12.5%)	0	1 (16.7%)	1 (16.7%)	1 (16.7%)	1 (8.3%)	0	0	1 (25.0%)
Probably	0	1 (4.2%)	0	0	1 (16.7%)	0	1 (8.3%)	0	0	1 (25.0%)

Notes: MedDRA version 21.1; related denotes possibly or probably.

TEAEs reported in more than one participant are presented in Extended Data Table 1. For participants receiving ARO-ANG3, the most frequently reported TEAEs were upper respiratory tract infection, headache and diarrhea. One participant in the pooled placebo cohort and one participant in the ARO-ANG3 35-mg cohort experienced a TEAE associated with ALT elevations >2× the upper limit of normal (ULN) and >3× ULN, respectively (Supplementary Table 2 and Supplementary Fig. 1a,b); neither TEAE was considered treatment related. The participant who received a single dose of ARO-ANG3 35 mg had been taking an herbal supplement known to be associated with liver injury. Notably, this participant’s elevation in ALT was transient, with return to near baseline by the end of the study and with cessation of the herbal supplement.

#### HP cohorts, MAD

Ten of 12 (83.3%) HPs who received a repeat dose of ARO-ANG3 reported at least one TEAE, with a total of 44 reported TEAEs (Table 3). There was no apparent dose-dependent increase in the incidence of TEAEs. There were no SAEs reported in participants receiving ARO-ANG3.

TEAEs reported in more than one participant are presented in Extended Data Table 1. The most frequently reported TEAEs in the HP MAD cohorts were headache, upper respiratory tract infection and vascular access site bruising. No participants in the MAD cohort experienced a TEAE associated with aminotransferase elevations.

#### Hepatic steatosis cohort, repeat dose

In participants with hepatic steatosis, 22 TEAEs were reported in 5 out of 6 (83.3%) participants receiving ARO-ANG3 compared to 8 TEAEs reported in all 3 (100%) participants receiving placebo (Extended Data Table 3).

There were no serious or severe TEAEs and no TEAEs leading to drug or study withdrawal in any participant receiving ARO-ANG3. One placebo participant experienced an SAE of moderate pancreatitis (Extended Data Table 3).

Two out of six (33.3%) participants reported TEAEs of injection site erythema that were deemed to be related to ARO-ANG3. In the placebo group, there were no TEAEs that were considered treatment related (Extended Data Table 3). The most frequently reported TEAEs (that is, those occurring in more than one participant) in the ARO-ANG3 group were headache and injection site erythema. In the placebo group, there were no TEAEs reported in more than one participant. No TEAEs related to adverse changes in markers of liver injury or function were reported. One participant receiving ARO-ANG3 demonstrated a post-dose peak increase in ALT >3× ULN, which was transitory (Supplementary Table 2 and Supplementary Fig. 2a,b).

**Extended Data Fig. 2:**
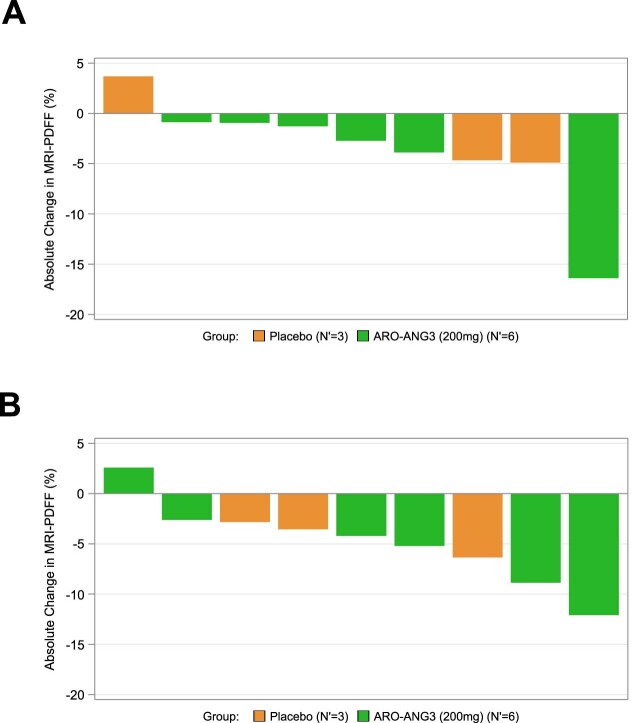
Individual absolute changes in liver fat content measured using magnetic resonance imaging-proton density fat fraction at Day 71 (A) and Day 168 (B) post dose for participants with hepatic steatosis receiving repeat doses of 200 mg ARO-ANG3 or placebo. Waterfall plots showing individual absolute changes in liver fat content, measured using magnetic resonance imaging-proton density fat fraction (MRI-PDFF), at post-dose Day 71 (A) and Day 168 (B) for participants with hepatic steatosis receiving repeat doses of 200 mg ARO-ANG3 or receiving placebo.

Waterfall plots show individual absolute changes in liver fat content, measured using MRI-PDFF at post-dose day 71 (Extended Data Fig. 2a) and at post-dose day 168 (Extended Data Fig. 2b) for participants receiving repeat doses of 200 mg of ARO-ANG3 or receiving placebo. The absolute change from baseline in liver fat at day 71 ranged from −0.87% to −16.39% (mean absolute change of −4.35%) with relative changes from baseline ranging from −4.74% to −69.6% (mean relative change of −18.23%) in the active treatment group. In the placebo group at day 71, absolute change in liver fat from baseline ranged from +3.68% to −4.89% (mean absolute change of −1.96%) with relative changes ranging from +20.91% to −29.32% (mean relative change of −8.56%). At day 168 in the active treatment group, absolute changes from baseline in liver fat ranged from +2.58% to −12.09% (mean change of −5.1%). Relative changes from baseline in liver fat ranged from +7.81% to −57.34% (mean relative change of −28.17%). In the placebo group, absolute change from baseline in liver fat ranged from −2.84% to −6.35% (mean change of −4.25%), with relative changes ranging from −16.14% to −23.52% (mean change of −20.33%).

### Pharmacokinetic response

ARO-ANG3 pharmacokinetic parameters were evaluated in HP cohorts only. ARO-ANG3 systemic absorption in HPs was rapid and sustained, with mean time to maximum plasma concentration (*T*_max_) ranging from 6.0 h to 10.5 h. ARO‑ANG3 was cleared from plasma compartment within 24–48 h after dosing, with a mean elimination half-life (*t*_½_) ranging from 3.9 h to 6.6 h. Full pharmacokinetic results for HPs are presented in Table 4.

**Table 4:** Summary of ARO-ANG3 plasma pharmacokinetic parameters after a subcutaneous injection of ARO-ANG3 in HPs (day 1 SAD cohorts; days 1 and 29 MAD cohorts)

	Dose	*C*_max_ (ng ml^−1^)	*T*_max_ (h)	AUC_last_ (h × ng ml^−1^)	AUC_inf_ (h × ng ml^−1^)	*t*_1/2_ (h)	CL/*F* (l h^−1^)	Vz/*F* (l)
SAD (dose 1, day 1)	35 mg	49.6 ± 17.5; 6	6.0 (6.0–9.2); 6	651 ± 161; 6	682 ± 156; 6	4.5 ± 1.4; 6	53.6 ± 12.2; 6	356 ± 151;
	100 mg	222 ± 82.3; 6	6.0 (6.0–9.0); 6	3,060 ± 1,040; 6	3,180 ± 1,120; 6	4.5 ± 1.0; 6	34.4 ± 10.5; 6	221 ± 72.3; 6
	200 mg	368 ± 254; 6	9.0 (0.5–12.1); 6	6,060 ± 3,160; 6	6,220 ± 3,050; 6	6.6 ± 4.6; 6	36.4 ± 10.6; 6	368 ± 309; 6
	300 mg	635 ± 214; 6	10.5 (6.0–18.0); 6	11,600 ± 3,360; 6	11,200 ± 3,510; 5	4.8± 1.4; 5	29.0 ± 9.30; 5	193 ± 53.6; 5
MAD (dose 1, day 1)	100 mg	273 ± 100; 4	7.5 (3.0–9.0); 4	3,310 ± 792; 4	3,020 ± 379; 3	3.9 ± 1.1; 3	33.4 ± 4.05; 3	182 ± 39.3; 3
	200 mg	583 ± 187; 4	10.5 (2.0–18.0); 4	8,870 ± 689; 4	8,910± 688; 4	5.0 ± 0.8; 4	22.6 ± 1.75; 4	162 ± 32.2; 4
	300 mg	779 ± 452; 4	9.0 (9.0–18.0); 4	12,400 ± 4,100; 4	14,000 ± 3,270; 3	5.3 ± 1.0; 3	22.2 ± 4.58; 3	175± 64.7; 3
MAD (dose 2, day 29)	100 mg	256 ± 55.3; 4	6.0 (6.0–6.0); 4	3,290± 612; 4	3,370 ± 564; 4	4.6 ± 0.6; 4	30.2 ± 4.59; 4	198 ± 34.7; 4
	200 mg	596 ± 81.7; 4	6.0 (3.0–9.0); 4	9,210 ± 777; 4	9,240± 777; 4	4.9 ± 0.3; 4	21.8 ± 1.83; 4	155 ± 19.1; 4
	300 mg	745 ± 438; 4	7.5 (1.0–9.0); 4	12,500 ± 3,650; 4	14,100 ± 2,550; 3	5.5 ± 1.4; 3	21.7 ± 3.60; 3	177 ± 63.7; 3

*C*_max_, maximum observed plasma concentration; AUC_last_, area under the plasma concentration versus time curve from the zero to the last quantifiable plasma concentration; AUC_inf_, area under the plasma concentration versus time curve from zero to infinity; CL/*F*, plasma clearance; Vz/*F*, terminal-phase volume of distribution.

Note: *T*_max_ is presented as median (minimum–maximum); *n*; all other parameters are presented as arithmetic mean ± s.d.; *n*.

As noted in the Methods, pharmacokinetic parameters were evaluated in HP cohorts only.

### Efficacy and pharmacodynamic responses

Results across multiple cohorts suggested robust, consistent and durable pharmacodynamic effects through at least 12 weeks, after single (day 1) or repeat (days 1 and 29) doses of ARO-ANG3 in HPs and repeat (days 1 and 29) doses for hepatic steatosis cohorts. Therefore, results summarized in the following sections report key pharmacodynamic and lipid data (ANGPTL3, TG, non-HDL-C, VLDL-C, LDL-C, HDL-C and ApoB concentrations) 12 weeks after the last dose, corresponding to day 85 for single dose (HP SAD cohorts) and day 113 for multiple doses (HP MAD and hepatic steatosis cohorts). Results from these analyses can be found in Table 5, Extended Data Table 2 (mixed model repeated measures (MMRM) analyses) and in the Supplementary Information (Supplementary Tables 3 and 4 (summary statistics)).

**Table 5:** Pharmacodynamic effects of ARO-ANG3 on serum ANGPTL3 and lipid-related variables

	Pooled placebo	ARO-ANG3 (35 mg)	ARO-ANG3 (100 mg)	ARO-ANG3 (200 mg)	ARO-ANG3 (300 mg)
ANGPTL3					
LS mean (s.e.) % change	6.20 (6.26)	−41.39 (10.21)	−56.83 (10.02)	−63.70 (9.97)	−78.12 (9.95)
LS mean of difference versus placebo (s.e.)	—	−47.59 (11.97)**	−63.03 (11.81)**	−69.91 (11.77)**	−84.32 (11.76)**
TG					
LS mean (s.e.) % change	7.53 (8.95)	18.79 (18.92)	−35.36 (14.46)	−49.46 (15.73)	−51.05 (14.48)
LS mean of difference versus placebo (s.e.)	—	11.26 (20.93)^NS^	−42.89 (17.01)*	−57.00 (18.10)*	−58.58 (17.02)*
Non-parametric analysis of % change from baseline to day 85					
Median difference and 95% confidence limit	—	−13.87 (−63.8, 26.2)^NS^	−35.48 (−82.1, −6.6)*	−53.95 (−99.2, −17.7)*	−54.91 (−102.5, −19.5)*
Non-HDL-C					
LS mean (s.e.)	−6.22 (4.98)	−18.00 (8.04)	−32.42 (8.12)	−14.61 (7.86)	−23.80 (7.81)
LS mean of difference versus placebo (s.e.)	—	−11.78 (9.46)^NS^	−26.20 (9.52)*	−8.39 (9.31)^NS^	−17.58 (9.26)^NS^
VLDL-C					
LS mean (s.e.)	6.08 (9.36)	16.56 (15.70)	−32.86 (14.19)	−46.12 (15.50)	−49.22 (14.22)
LS mean of difference versus placebo (s.e.)	—	10.47 (18.27)^NS^	−38.95 (17.00)*	−52.20 (18.10)*	−55.30 (17.02)*
LDL-C					
LS mean (s.e.)	−3.74 (7.87)	−22.30 (12.84)	−27.97 (13.13)	4.71 (12.65)	−12.88 (12.64)
LS mean of difference versus placebo (s.e.)	—	−18.56 (15.06)^NS^	−24.23 (15.31)^NS^	8.45 (14.89)^NS^	−9.14 (14.89)^NS^
HDL-C					
LS mean (s.e.)	6.10 (4.31)	−2.17 (7.10)	2.69 (6.96)	−10.17 (6.99)	−12.52 (6.90)
LS mean of difference versus placebo (s.e.)	—	−8.27 (8.31)^NS^	−3.41 (8.18)^NS^	−16.27 (8.21)^NS^	−18.62 (8.14)*
ApoB					
LS mean (s.e.)	−3.08 (4.74)	−19.11 (7.49)	−22.62 (7.62)	−5.78 (7.34)	−12.45 (7.25)
LS mean of difference versus placebo (s.e.)	—	−16.02 (8.87)^NS^	−19.53 (8.98)*	−2.70 (8.74)^NS^	−9.37 (8.67)^NS^

NS, not significant.

Note: Friedewald calculation was used for LDL-C measurements, unless TGs were >400 mg dl^−1^, wherein a direct LDL-C measurement was used.

**P < 0.001 versus placebo; *P < 0.05 versus placebo; NS *P* ≥ 0.05 versus placebo.

### HPs: effects on ANGPTL3 levels

#### HP cohorts, SAD

**Extended Data Fig. 3:**
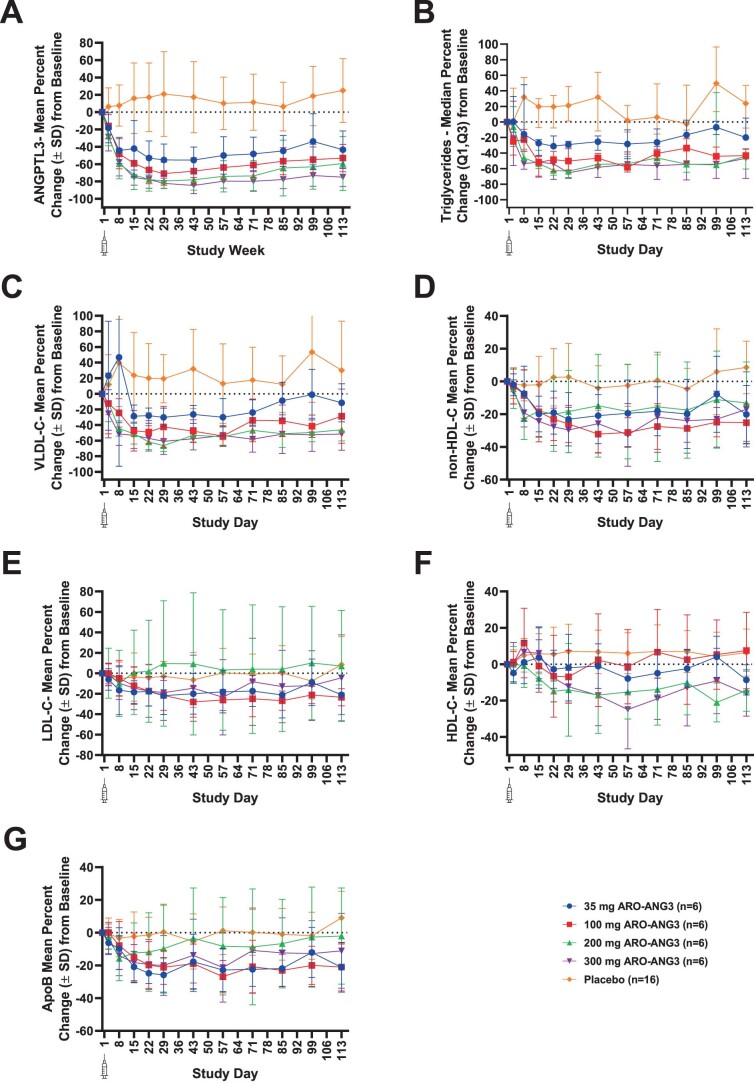
Reductions in serum ANGPTL3, TG, LDL-C, VLDL-C, HDL-C, and non-HDL-C concentrations in healthy participants with single ascending doses of ARO-ANG3 or placebo. Median percent change (+/-Q1,Q3) from baseline in triglycerides (B). Mean percent change (±SD) from baseline in ANGPTL3 (A), VLDL-C (C), non-HDL-C (D), LDL-C (E), HDL-C (F), and ApoB (G) in healthy participants receiving a subcutaneous dose of ARO-ANG3 (n = 4 per cohort of 35 mg [blue] 100 mg [red], 200 mg [green], 300 mg [purple]) or placebo [orange] on Day 1). Abbreviations: ApoB = Apolipoprotein B; HDL-C = high density lipoprotein cholesterol; Q1, Q3 = quartile 1, quartile 3; LDL-C = low density lipoprotein cholesterol; SD = standard deviation; VLDL = very low density cholesterol.

Dosing with ARO-ANG3 in HP cohorts reduced serum ANGPTL3 concentrations starting at day 3, reaching maximal mean reductions between 2 weeks and 6 weeks after the single dose (Extended Data Fig. 3). ANGPTL3 reduction occurred in a dose-dependent manner, with mean (s.d.) percentage change from baseline at day 85 ranging from −44.7% (17.9%) to −77.8% (10.7%) at 35 mg and 300 mg, respectively, compared to an increase of 6.4% (28.1%) for placebo (Supplementary Table 3).

#### HP cohorts, MAD

Open-label, repeat doses of ARO-ANG3 demonstrated changes in most pharmacodynamic parameters similar to single-dose ARO-ANG3. The HP MAD cohorts were not placebo controlled. However, given similarities between the HP SAD and MAD cohorts, data pooled from the placebo groups in the SAD cohorts were compared to results from the MAD HPs.

**Extended Data Fig. 4:**
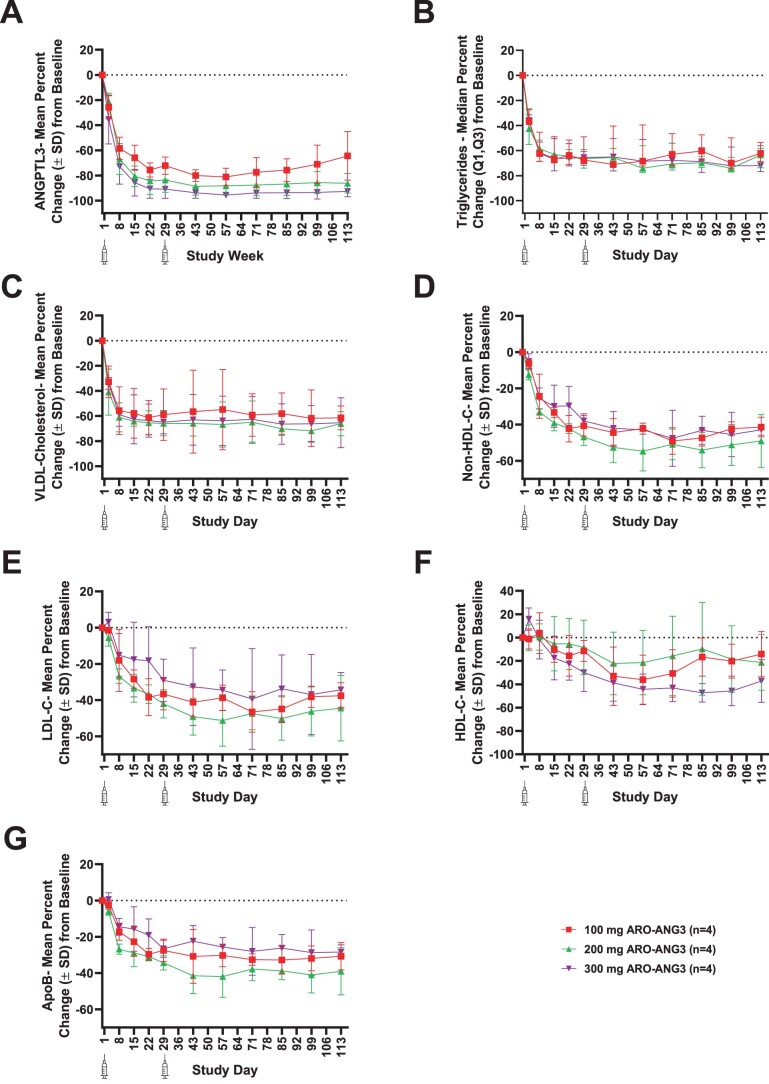
Reductions in serum ANGPTL3, TG, LDL-C, VLDL-C, HDL-C, non-HDL-C, and ApoB concentrations in healthy participants with multiple ascending doses of ARO-ANG3. Median percent change (Q1, Q3) from baseline in triglycerides (B). Mean percent change (±SD) from baseline in ANGPTL3 (A), VLDL-C (C), non-HDL-C (D), LDL-C (E), HDL-C (F), and ApoB (G) in healthy participants receiving a subcutaneous dose of ARO-ANG3 (n = 4 per cohort of 100 mg [red], 200 mg [green] or 300 mg [purple]) on Days 1 and 29). Abbreviations: ApoB = Apolipoprotein B; HDL-C = high density lipoprotein cholesterol; Q1 = quartile 1; Q3 = quartile 3; LDL-C = low density lipoprotein cholesterol; SD = standard deviation; VLDL = very low density cholesterol.

Dosing with ARO-ANG3 reduced ANGPTL3 concentrations beginning at day 3. Reduction occurred in a dose-dependent manner, with mean (s.d.) percentage change from baseline at day 113 ranging from −64.4% (19.3%) to −92.7% (4.3%) for 100 mg and 300 mg, respectively, compared to an increase of 25.0% (36.8%) for the SAD pooled placebo (Extended Data Fig. 4 and Supplementary Table 4).

### HPs: effects on exploratory parameters

#### HP cohorts, SAD

Reductions in fasting TG concentrations were observed starting at day 3 and sustained until the end of the study. Median percentage change from baseline to day 85 ranged from −16.6% to −54.4% for the 35-mg and 300-mg doses, respectively, compared to −2.6% for placebo. VLDL-C concentrations decreased, with mean (s.d.) percentage change from baseline at day 85 ranging from −8.8% (23.4%) to −51.7% (24.8%) for the 35-mg and 300-mg doses, respectively, compared to 12.3% (36.4%) for pooled placebo (Extended Data Fig. 3).

Mean (s.d.) percentage change from baseline in non-HDL-C at day 85 ranged from −28.7% (8.5%) to −17.5% (29.4%) for the 100-mg and 200-mg doses, respectively, compared to −4.6% (12.6%) for placebo (Extended Data Fig. 3).

At day 85, LDL-C mean (s.d.) percentage change from baseline ranged from −26.8% (9.5%) to 4.1% (61.1%) for 100-mg and 200-mg doses, respectively, compared to 0.3% (26.8%) for pooled placebo (Extended Data Fig. 3). Small reductions in mean LDL-C were observed in several participants in the ARO-ANG3 200-mg treatment cohort. However, two out of six HPs receiving ARO-ANG3 had baseline TG concentrations >300 mg dl^−1^, which were associated with a post-dose increase in LDL-C concentrations in some participants. This increase in LDL-C in participants with high baseline TG led to a lack of apparent dose response for non-HDL-C and LDL-C at the higher dose levels. ApoB concentrations were reduced, with mean (s.d.) percentage change from baseline at day 85 ranging from −6.7% (27.1%) to −23.1% (9.9%) for the 200-mg and 100-mg doses, respectively, compared to −1.0% (15.8%) in pooled placebo (Extended Data Fig. 3).

Mean (s.d.) percentage change from baseline in HDL-C at day 85 ranged from 2.5% (24.6%) to −12.9% (21.1%) for the 100-mg and 300-mg doses, respectively, compared to 6.9% (11.5%) in pooled placebo (Extended Data Fig. 3).

Results from MMRM analysis (least squares (LS) mean difference and LS mean difference versus placebo) for ANGPTL3, TG, non-HDL-C, VLDL-C, LDL-C, HDL-C and ApoB at day 85 are presented in Table 5.

Full results (mean percentage change from baseline, MMRM analysis) for other lipids/lipoproteins at day 85 are presented in Extended Data Table 4. Non-parametric (post hoc) analysis of TGs shows results consistent with the MMRM analysis.

#### HP cohorts, MAD

Reductions in TGs were substantial and sustained. Median percentage TG change from baseline to day 113 ranged from −62.2% to −72.0% for the 100-mg and 300-mg doses, respectively, compared to an increase of 23.8% for the SAD pooled placebo (Supplementary Table 4). VLDL-C concentrations decreased, with mean (s.d.) percentage change from baseline at day 113 ranging from −61.5% (9.5%) and −66.1% (9.6%) for the 100-mg and 200-mg doses, respectively, compared to 30.2% (63.0%) in the SAD pooled placebo cohort (Supplementary Table 4 and Extended Data Fig. 4).

After a repeat dose of ARO-ANG3, the mean (s.d.) percentage change from baseline in non-HDL-C at day 113 ranged from −41.4% (5.5%) to −49.0% (14.6%) for the 100-mg and 200-mg doses, respectively, compared to an increase of 8.6% (16.1%) for the SAD pooled placebo cohort (Supplementary Table 4 and Extended Data Fig. 4).

Across all active treatment cohorts, mean (s.d.) LDL-C decreased and remained below baseline levels until day 113. At day 113, LDL-C mean percentage change from baseline ranged from −34.4% (9.6%) to −44.5% (18.1%) for the 300-mg and 200-mg doses, respectively, compared to an increase of 8.5% (27.5%) for the SAD pooled placebo cohort (Supplementary Table 4 and Extended Data Fig. 4). ApoB concentrations were reduced, with mean (s.d.) percentage change from baseline at day 113 ranging from −28.4% (4.0%) to −39.0% (13.0%) for the 300-mg and 200-mg doses, respectively, compared to 9.1% (16.2%) for the SAD pooled placebo (Supplementary Table 4 and Extended Data Fig. 4).

Mean (s.d.) percentage change from baseline in HDL-C at day 113 ranged from −14.1% (19.4%) to −37.2% (18.3%) for the 100-mg and 300-mg doses, respectively, compared to 6.4% (12.9%) in the SAD pooled placebo cohort (Supplementary Table 4 and Extended Data Fig. 4).

For exploratory purposes, we compared the MAD group to the pooled placebo from the SAD group by assuming no impact in pharmacodynamics with different dosing frequency for placebo participants. Results from this post hoc MMRM analysis for ANGPTL3, TG, non-HDL-C, VLDL-C, LDL-C, HDL-C and ApoB at day 113 are presented in Extended Data Table 2. Full results for other lipids/lipoproteins at day 113 are presented in Extended Data Table 5 and Supplementary Table 4.

### Hepatic steatosis cohort: effects on ANGPTL3 levels

Mean (s.d.) percentage change in ANGPTL3 from baseline at day 113 was −85.3% (12.7%) for the 200-mg dose compared to an increase of 13.0% (4.7%) for placebo (Extended Data Table 6).

### Hepatic steatosis cohort: effects on exploratory parameters

The median TG percentage change from baseline at day 113 was −44.1% for the 200-mg dose compared to an increase of 47.1% for placebo (Extended Data Table 6). For VLDL-C, the mean (s.d.) percentage change from baseline was −43.5% (20.8%) for the 200-mg dose compared to 45.9% (50.8%) in the placebo cohort.

The mean (s.d.) LDL-C percentage change from baseline was −34.6% (14.1%) for the 200-mg dose compared to −4.3% (29.3%) for placebo. For ApoB, mean (s.d.) percentage change from baseline at day 113 was −20.5% (15.4%) for the 200-mg dose compared to −0.9% (7.9%) for placebo (Extended Data Table 6).

On day 113, the non-HDL-C mean (s.d.) percentage change from baseline was −36.7% (15.0%) for the 200-mg dose compared to 0.2% (14.3%) in the placebo cohort (Extended Data Table 6). Changes in HDL-C were observed with mean (s.d.) percentage change from baseline at day 113 of −53.0% (9.2%) for the 200-mg dose compared to −10.4% (2.1%) for placebo.

Results from MMRM analysis for ANGPTL3, TG, non-HDL-C, VLDL-C, LDL-C, HDL-C, ApoB and other lipids or lipoproteins at day 113 are presented in Extended Data Tables 5 and 6.

In summary, regardless of study population (HPs or participants with hepatic steatosis), repeat dosing of ARO-ANG3 consistently reduced ANGPTL3 by approximately 80–90%. This reduction was greater than that seen when ARO-ANG3 was given as a single dose. Although the relative change in pharmacodynamic parameters varied by population, and was dependent on baseline concentrations, repeat dosing of ARO-ANG3 consistently provided robust and sustained reductions in key pharmacodynamic parameters (non-HDL-C, VLDL-C, LDL-C and ApoB).

## Discussion

This first-in-human, proof-of-concept, phase 1 study demonstrated that ARO-ANG3, a therapy that acts through an RNA interference (RNAi) mechanism, resulted in robust and sustained reductions from baseline of up to −92.7% in serum ANGPTL3 concentrations, with concomitant reductions in serum TG and atherogenic lipoproteins (LDL-C, non-HDL-C and VLDL-C) in HP participants. ANGPTL3 is a key regulator of circulating levels of TG and TRL and cholesterol levels (for example, LDL-C) through reversible inhibition of the enzymes LPL and endothelial lipase and, therefore, represents a novel therapeutic target for reducing atherogenic lipoproteins^14^. ARO-ANG3 was generally well tolerated with no apparent adverse effects on liver transaminases. In a small sample of participants with hepatic steatosis, ARO-ANG3 also decreased atherogenic lipoproteins, and, notably, no increase in liver fat was observed after repeat dosing, with most participants showing a numerical decline in liver fat content^18^. These results suggest that silencing ANGPTL3 protein synthesis with a hepatocyte-targeted siRNA is a viable approach for reducing residual cardiovascular risk associated with atherogenic lipoproteins.

ARO-ANG3 showed durable pharmacologic effects lasting over 3 months after a single dose. This is a consequence of the unique intracellular mechanism of action of RNAi. After hepatic uptake of ARO-ANG3 and cleavage of its passenger RNA strand, the guide strand loads onto the RNA-induced silencing complex (RISC) and pairs with and degrades the *ANGPTL3* mRNA, reducing protein synthesis. RISC effectuates a catalytic process that prolongs the RNAi effect, providing a durable treatment response^14,19^.

Inherited deficiency in ANGPTL3 yields lower serum TG, LDL-C and HDL-C concentrations and is independently protective against coronary disease^10^. The lipid changes induced by ARO-ANG3 phenocopy *ANGPTL3* loss-of-function mutations. The therapeutic mechanism behind ANGPTL3 inhibition has been partially elucidated. By inhibiting hepatic ANGPTL3 synthesis, ARO-ANG3 enhances LPL activity, which lowers circulating TG levels through hydrolysis of TRLs. The decrease in serum ApoC-III in our study paralleled the reduction of TGs and does not account for the effect of ARO-ANG3 on TRLs, consistent with experimental data^20^. Inhibition of ANGPTL3 promotes VLDL remodeling and preferential removal of VLDL remnants from circulation, thereby limiting conversion to LDL-C^5^. In addition, inhibition of ANGPTL3 synthesis enhances endothelial lipase activity, which lowers HDL-C levels^4,14^. Although studies have shown that ANGPTL3 regulates the clearance of ApoB-containing lipoproteins, its role in regulating hepatic production of ApoB is less clear^6,7,14^. Hence, studies to fully define the mechanism of action of ARO-ANG3 on lipid and lipoprotein metabolism in humans, including those with different dyslipidemias, are required^14,21^.

Clinical studies with vupanorsen and evinacumab, targeting ANGPTL3, have shown that both can reduce LDL-C and TG. However, vupanorsen appears to increase ALT and risk of hepatic steatosis^11,12^. The more robust reductions in LDL-C observed with repeat doses of ARO-ANG3 compared to that observed with vupanorsen^12,17^ suggest the former’s greater potency. This observation also raises the question as to whether a more potent effect of ARO-ANG3 involves the regulation of production and/or clearance of LDL particles, with further studies required^4,5,14,21^. This notion requires verification and may also involve a threshold effect of the inhibition of ANGPTL3 by ARO-ANG3. Evinacumab is approved for the treatment of homozygous familial hypercholesterolemia but requires frequent intravenous infusions. ARO-ANG3 is being investigated in multiple phase 2 clinical trials using subcutaneously administered dose every 3 months (Q3), which is more convenient for patients and likely to enhance patient adherence to treatment. Although no data from clinical outcome trials are available, animal experiments indicate that antagonism of ANGPTL3 can inhibit the development of atherosclerosis^9,22^, with support derived from observational data in humans^9,10^.

ARO-ANG3 has demonstrated a safety profile supportive of late-stage clinical development. Mild injection site reactions were the most frequently reported TEAEs and are common to all subcutaneous injectables. No thrombocytopenia or liver toxicity was observed, even with repeat dosing. Clinical trials have shown that ASO treatment with vupanorsen was associated with hepatic steatosis and elevations in ALT^12,17^. In our clinical study, no meaningful adverse changes in liver fat were observed with ARO-ANG3 treatment in participants with baseline hepatic steatosis. Although transient mild elevations in ALT were observed with ARO-ANG3 in a small number of participants, these cases were associated with use of a concomitant hepatotoxic supplement or medications and were self-limited. Hepatic steatosis has not been reported in carriers of *ANGPTL3* loss-of-function variants^6,8–10,20^, and animal studies support reduction of liver fat with ANGPTL3 inhibition^23,24^. Therefore, the increase in liver fat reported with vupanorsen may be molecule specific or may be due to the use of higher and more frequent ASO dosing regimens.

This phase 1 study was designed as an evaluation of the safety and efficacy of ARO-ANG3 using a basket of diverse dyslipidemic populations to explore different options for later-stage clinical development. This design is consistent with FDA guidance as a ‘master’ protocol intended to address multiple questions in a single study and is an acceptable approach for early-phase studies with a new therapeutic agent that is directed at a common target across a diverse population^25^. However, the study design does have limitations, including small sample sizes and a short-term period of intervention. Only nine participants with hepatic steatosis were included in the study to allow for inclusion of participants with other dyslipidemias, and further investigations required to evaluate the effect of ARO-ANG3 on liver fat content are included in ongoing phase 2b clinical trials. Furthermore, not all interventions in the basket trial design were placebo controlled—for example, HPs in the MAD cohorts. Therefore, in an exploratory analysis, data pooled from the placebo groups in the SAD cohorts were used to compare with results from the MAD HPs. Additional limitations include a lack of data in the post-prandial setting and a predominance of self-reported white male participants enrolling in the study, pointing to the need for further investigations in larger studies with more diverse patient populations. Given the consistent and sustained lipid-lowering effect of ARO-ANG3, combined with the safety profile, results support further clinical development of ARO-ANG3. Two phase 2 dose-finding studies are ongoing in adults with mixed dyslipidemia (NCT04832971) and homozygous familial hypercholesterolemia (NCT05217667) to evaluate the safety and efficacy of ARO-ANG3 in these at-risk patient populations.

In summary, results from this early-stage clinical study indicate that siRNA therapy targeting *ANGPTL3* mRNA was generally well tolerated and can effectively lower circulating concentrations of atherogenic lipoproteins. These early results are encouraging and show the rapid and sustained TG-lowering and TRL-lowering effects of ARO-ANG3 over a 16-week period. ARO-ANG3 also lowers LDL-C, in contrast to reported increases with fibrates and omega-3 fatty acids^26,27^. ARO-ANG3 could address a major gap in the secondary prevention of ASCVD and could be particularly valuable for managing high-risk populations, such as mixed dyslipidemia and familial hypercholesterolemia^14^. Future studies are required to assess the effect of ARO-ANG3 on major ASCVD outcomes in high-risk populations as well as the long-term safety and cost utility of this promising new treatment^14^.

**Fig. 1:**
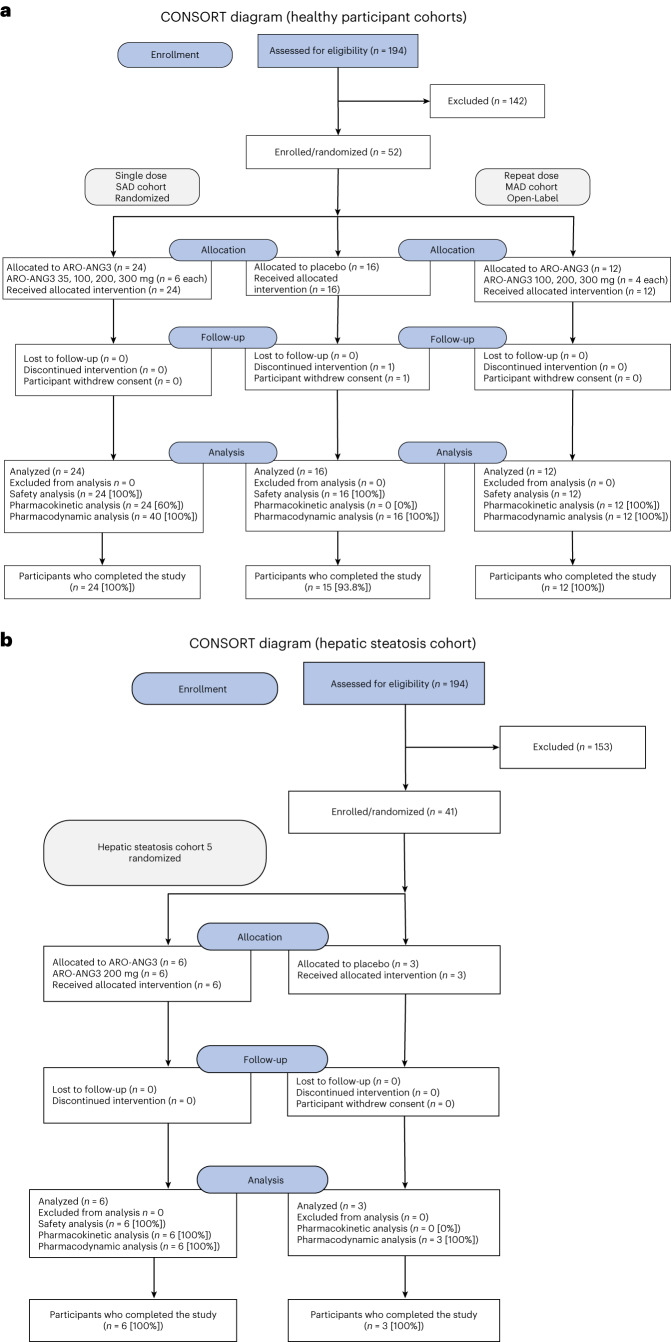
CONSORT diagrams. **a**,**b**, Participant allocation for the SAD and MAD HP cohorts (**a**) and dyslipidemic cohorts (**b**) in the phase 1 AROANG1001 study. Note: The key results from this study are first reported for HPs and individuals with hepatic steatosis.

## Methods

ARO-ANG3 is a synthetic, double-stranded, hepatocyte-targeted NAG-conjugated RNAi trigger designed to target mRNA transcripts from the *ANGPTL3* gene using an RNAi mechanism, thereby reducing hepatic and blood levels of ANGPTL3 protein. Both the antisense and sense strand of ARO-ANG3 comprise 21 2′ modified nucleotide subunits, modified at the 2′ positions of the ribose subunits with either fluorine (2′F) or methoxy (2′MeO) groups. The sense strand additionally contains two inverted abasic subunits and an *N*-acetylgalactosamine targeting moiety. The sequence of the molecule can be found in Supplementary Fig. 3.

### Study design and participants

This phase 1, multicenter, randomized, double-blind, placebo-controlled, open-label single and multiple dose-escalating study (NCT03747224) was approved by the ethics committees (ECs) and institutional review boards (IRBs) of participating centers in Australia (three sites; Linear Clinical Research, Royal Adelaide Hospital and Royal Prince Alfred Hospital) and New Zealand (three sites; Auckland Clinical Studies, Middlemore Clinical Trials and Lipid and Diabetes Research Group). The ECs and IRBs included Bellberry Human Research Ethics Committee (HREC), Northern B Health and Disability Ethics Committee (HDEC) and Central Adelaide Local Health Network (CALHN) HREC.

The study was conducted in accordance with the Declaration of Helsinki and International Council for Harmonisation Good Clinical Practice guidelines. Written informed consent was obtained from all participants. Sex and race were self-reported by all participants. All participants were reimbursed for their time and travel in accordance with local regulations.

The first patient to be consented was consented on 10 December 2018, and the first patient was randomized on 4 January 2019. The last patient was consented on 24 January 2020, and the last patient visit was on 17 May 2021. All cohorts, including those not reported here, have completed the study.

The study comprised six double-blind cohorts and eight open-label cohorts, with SAD and MAD designs. The aim of the study was to determine the safety and efficacy of ARO-ANG3 using escalating single doses of ARO-ANG3 in HPs and multiple doses in participants with hepatic steatosis. Eligible HPs in double-blind cohorts were allocated a unique randomization number, in accordance with the randomization schedule. In each cohort, the first two participants (sentinels) were randomized separately, one to active treatment and one to placebo. Each participant was assigned to either active (ARO-ANG3) or placebo treatment. The allocation of active treatment or placebo was determined by a computer-generated randomization schedule provided to the clinical site pharmacies by Novotech. The randomization schedule specified the treatment allocated to each randomization number. Sites enrolled the participants per the randomization schedule prepared by the pharmacists. After completion of the double-blind cohorts, the sponsor was unblinded, but the principal investigator and study participants remained blinded. This could occur after all participants in a cohort had completed the final planned study visit on day 113. The study schema is shown in Extended Data Fig. 1. CONSORT diagrams for the HP and hepatic steatosis cohorts are shown in Fig. 1a,b.

Cohorts 1–4 included eligible HP participants aged 18–65 years at screening who were on a stable diet for at least 4 weeks with no plans to meaningfully alter diet or body mass index (BMI) during the study. Placebo-controlled HP cohorts 1–4 were required to have a fasting screening TG >100 mg dl^−1^ and LDL-C >70 mg dl^−1^ and to have not received any lipid-lowering or TG-lowering therapy.

A basket trial approach was taken to evaluate early proof of concept, and four cohorts of participants with diverse dyslipidemias were enrolled. These cohorts included participants with hepatic steatosis (defined as baseline MRI-PDFF ≥10%) in cohort 5; participants with LDL-C >70 mg dl^−1^ and on a stable statin regimen in cohort 6; participants with a diagnosis of familial hypercholesterolemia (genetic diagnosis or Dutch Lipid Clinic Network Score ≥6) in cohort 7; and participants with TG ≥300 mg dl^−1^ in cohort 8. Cohort 9 comprised an extension cohort of participants with familial hypercholesterolemia from cohort 7. The cohort summary and dose escalation schedule are shown in Supplementary Tables 5 and 6, respectively.

Full details of eligibility and exclusion criteria are described in the Study Protocol.

### Study treatments and procedures

The cohort dosing schedule is presented in Supplementary Table 1. Cohorts 1–4 were randomized and double-blinded, with all receiving single subcutaneous doses of ARO-ANG3 or placebo at escalating dose levels of 35 mg, 100 mg, 200 mg and 300 mg. Samples were collected for clinical laboratory tests at each clinic visit. Fasting serum samples were collected at baseline and at times specified in the protocol. Participants fasted from food for at least 8 h before serum sample collection.

The Friedewald calculation was used for LDL-C measurements, unless TGs were >400 mg dl^−1^, wherein a direct LDL-C measurement was used. Medpace Research Laboratories reported results for lipid/lipoprotein parameters. Full details of the above assessments and procedures are described in the Study Protocol.

ANGPTL3 was measured in serum sample sets batched by participant using an ELISA (R&D Systems) read for absorbance on a Tecan Sunrise reader.

MRI-PDFF imaging sites used a 3T (preferred) or 1.5T (acceptable) MR system with appropriate abdominal coils to perform imaging of the liver, including T2 coronal liver, T2 axial liver and multi-echo fat quantification as per the image acquisition manual. Images were centrally read and reported by Medpace Core Labs. Lipid and lipoprotein results were reported by Medpace Research Laboratories. All other parameters were reported by Sonic Clinical Trials.

### Pharmacokinetics

Pharmacokinetic samples were collected only for HP cohorts.

On dosing days (days 1 and 29 (MAD cohorts only)), plasma samples were collected at time 0 (pre-dose), 15 min and 0.5, 1, 2, 3, 6, 9, 12, 18, 24 and 48 h post-dose.

The primary objective of the study was to determine the incidence and frequency of adverse events possibly or probably related to treatment as a measure of the safety and tolerability of ARO-ANG3 using escalating single and multiple doses in HPs and multiple doses in patients with dyslipidemia. Adverse events were coded according to the Medical Dictionary for Regulatory Activities (MedDRA) version 19.1.

### Secondary objectives

- To evaluate the single-dose and multi-dose pharmacokinetics of ARO-ANG3 in HPs
- To determine the reduction in fasting serum ANGPTL3 from baseline in response to a single dose and multiple doses of ARO-ANG3 as a measure of drug activity in HPs and in response to multiple doses of ARO-ANG3 in patients with dyslipidemia (all values drawn after at least 8-h fast)

### Exploratory objectives

- To evaluate the effect of ARO-ANG3 on change from baseline in fasting LDL-C, total cholesterol, non-HDL-C, HDL-C, VLDL-C, TG, Lp(a), total ApoB, ApoB 48, ApoB 100, ApoC III, ApoC II, ApoA V, LPL mass (if feasible), hepatic lipase mass (if feasible), cholesteryl ester transfer protein (CETP) mass (if feasible) and ApoA I (all values drawn after at least 8-h fast)
- To evaluate the effect of doses of ARO-ANG3 on changes from baseline in BMI
- To evaluate the effect of ARO-ANG3 on changes from baseline in fasting serum blood glucose, hemoglobin A1c, C peptide, glucose tolerance test and fasting serum insulin
- To evaluate the effect of ARO-ANG3 on change from baseline liver fat content using MRI-PDFF in cohort 5 only
- To evaluate the effect of ARO-ANG3 on change from baseline in post-prandial (post-standardized high-fat/high-carbohydrate meal) serum TGs in specified cohorts
- To evaluate excretion of ARO-ANG3 (full length and metabolites) and identify metabolites in plasma and urine in the multi-dose HP cohorts

#### Endpoints

The primary endpoints were incidence of adverse events or serious advserse events and relationship to study treatment; physical examinations, including height, weight and BMI; vital signs (systolic/diastolic blood pressure, temperature, heart rate and respiratory rate); electrocardiogram (ECG) measurements; injection site reactions; clinical laboratory tests (serum chemistry (including hemoglobin A1c), hematology, coagulation, urinalysis, microscopic urinalysis (if indicated), serology, follicle stimulating hormone (FSH), drug and alcohol use, pregnancy, lipid parameters, serum insulin levels, serum glucose levels and stool occult blood test); concomitant medications/therapy; and reasons for treatment discontinuation due to toxicity.

The secondary pharmacodynamic endpoint was change in fasting serum ANGPTL3 concentration. The exploratory key pharmacodynamic endpoints included fasting TG, fasting non-HDL-C, fasting VLDL-C, fasting LDL-C, fasting HDL-C and ApoB in response to escalating single or multiple doses of ARO-ANG3.

Additional lipid parameters, including ApoC III and total cholesterol, were also assessed as exploratory endpoints.

Full lists of secondary and exploratory endpoints are included below.

#### Statistical analyses

For serum ANGPTL3, lipid, lipoprotein and lipoprotein concentrations, the percentage change from baseline at post-baseline visits were analyzed using a linear mixed model repeated measures (MMRM) approach with fixed effects for treatment, week, treatment by week interaction, baseline value as a continuous covariate and baseline by treatment interaction. As a post hoc analysis, in cases when the statistical assumptions could not be satisfied, especially for the analysis of fasting TG, a non-parametric approach with the Hodges–Lehmann method was used. The sample size chosen for the study was selected without statistical justification but was considered adequate for assessing the study objectives.

Not all interventions in the basket trial design were placebo controlled—for example, HPs in the MAD cohorts. Therefore, in an exploratory post hoc analysis, given similarities between the HP SAD and MAD cohorts, data pooled from the placebo groups in the SAD cohorts were used to compare with results from the MAD HPs.

### Endpoints

#### Secondary endpoints

- Single-dose and multi-dose plasma pharmacokinetics of ARO-ANG3 were assessed by analysis of the following parameters on days 1 and 29:
  - AUC_last_
  - AUC_0-24_^a^
  - AUC_inf_
  - AUC_%extrap_^a^
  - *C*_max_
  - *T*_max_
  - *t*_½_
  - CL/*F*
  - Vz/*F*
- Single-dose and multi-dose urine pharmacokinetics of ARO-ANG3 were assessed by analysis of the following parameters on days 1 and 29:
  - Ae_0-24_^a^
  - Fe_0-24_^a^
  - CLR ^a^
- The reduction in fasting serum ANGPTL3 from baseline was assessed by analysis of fasting serum ANGPTL3 concentration.

^a^Results for plasma pharmacokinetics parameters AUC_0–24_ and AUC_%extrap_ and urine pharmacokinetics parameters Ae_0–24_, Fe_0–24_ and CLR are not described in this manuscript.

#### Exploratory endpoints

- Fasting LDL-C
- Total cholesterol
- Fasting non-HDL-C
- Fasting HDL-C
- Fasting VLDL-C
- Fasting TG
- Fasting Lp(a)
- Fasting total ApoB
- Fasting ApoB 48
- Fasting ApoB 100
- Fasting ApoC III
- Fasting ApoC II
- Fasting ApoA V
- LPL mass (if feasible)
- CETP mass (if feasible)
- Fasting ApoA I
- Hemoglobin A1c
- C peptide
- Fasting serum blood glucose
- Glucose tolerance test
- Fasting serum insulin
- Post-prandial TG test
- Liver fat content using MRI
- BMI

### Inclusion/exclusion criteria

#### Inclusion criteria

Participants who met all of the following criteria at screening were eligible to participate in the study:

1. Male or female participants 18–65 years of age. In cohorts 7, 7b, 7c, 8 and 9, participants up to age 70 years were eligible if otherwise healthy and at the discretion of the investigator.
2. Able and willing to provide written informed consent before the performance of any study-specific procedures.
3. Participants with a BMI between 19.0 kg m^−2^ and 40.0 kg m^−^^2^, inclusive and on a stable diet for at least 4 weeks with no plans to significantly alter diet or BMI over the course of the study.
4. A 12-lead ECG at screening and pre-dose assessment that, in the opinion of the principal investigator, had no abnormalities that compromise participant safety in this study.
5. Non-nursing women.
6. Fasting serum TG >100 mg dl^−1^ (1.13 mmol l^−1^) at screening (applicable to cohorts 1, 2, 3 and 4 only; did not apply to cohorts 2b, 3b or 4b).
7. Fasting serum LDL-C >70 mg dl^−1^ (1.81 mmol l^−1^) at screening (applicable to cohorts 1, 2, 3 and 4 only; did not apply to cohorts 2b, 3b or 4b).
8. Participants using two highly effective forms of contraception (both male and female partners) during the study and for 3 months after the dose of ARO-ANG3. Men were not to donate sperm for at least 3 months after dose of the last study treatment. Male partners of female participants and female partners of male participants were also required to use contraception, if they were of childbearing potential. Women of childbearing potential were required to have a negative urine pregnancy test at screening and on day 1. Women not of childbearing potential were to be postmenopausal (defined as cessation of regular menstrual periods for at least 12 months), confirmed by FSH level in the postmenopausal reference range.

Using twice the normal protection of birth control by using a condom and one of the following:

- Birth control pills
- Depot or injectable birth control
- Intrauterine device
- Birth control patch (for example, Othro Evra)
- Vaginal ring (for example, NuvaRing)

Surgical sterilization (that is, tubal ligation or hysterectomy for women or vasectomy for men or other forms of surgical sterilization) that could be verified in the participant’s medical history was acceptable as a single form of contraception.

Rhythm methods were not considered as highly effective methods of birth control. Participant abstinence for the duration of the study and 3 months after the dose of ARO-ANG3 was acceptable only when this method was in alignment with the normal lifestyle of the participant.

9. Participants who were willing and able to comply with all study assessments and adhere to the protocol schedule.
10. Must have had suitable venous access for blood sampling.
11. Aspartate transaminase (AST) and ALT <1.5× ULN at screening for cohorts 1–4 and 2b through 4b (one repeat screen test was allowed).
12. AST and ALT <3× ULN at screening for cohorts 5, 6, 7, 7b, 7c and 8 (one repeat screen test was allowed).
13. Creatinine levels ≤ULN at screening (one repeat screen test was allowed).
14. MRI-PDFF indicating a liver fat content of ≥10% (cohort 5 only).
15. On a stable regimen of statin therapy for at least 6 months and LDL-C >70 mg dl^−1^ (1.81 mmol l^−1^) at screening (cohort 6 only).
16. Documented genetic diagnosis consistent with familial hypercholesterolemia (homozygous or heterozygous) with genotype documented in a verifiable source document OR Dutch Lipid Clinic Network Score ≥6 (cohorts 7, 7b and 7c only).
17. LDL-C >100 mg dl^−1^ (2.59 mmol l^−1^) despite standard-of-care therapy or LDL-C >70 mg dl^−1^ (1.81 mmol l^−1^) while on a PCSK 9 inhibitor or LDL-C >70 mg dl^−1^ (1.81 mmol l^−1^) in the presence of documented ASCVD (cohorts 7, 7b and 7c only).
18. Screening fasting TG ≥300 mg dl^−1^ (3.39 mmol l^−1^) (cohort 8 only). Up to two repeated fasting TG tests during screening was acceptable.
19. Cohort 9 only: must have had completed all doses in cohorts 7, 7b or 7c.

### Exclusion criteria

Participants who met any of the following criteria at screening were not eligible to participate in the study:

1. Female participants with a positive pregnancy test or who were lactating.
2. Acute signs of hepatitis (for example, moderate fever, jaundice, nausea, vomiting and abdominal pain) at screening or at baseline.
3. Use of prescription medication that, in the opinion of the study investigator or the sponsor, would interfere with study conduct. Stable regimens to lower LDL-C or TG or to treat cardiovascular disease, stable regimens of anti-hypertensives and stable regimens of anti-platelet agents or anti-coagulants were acceptable for cohorts 5, 6, 7 and 8 as long as the participant met other criteria. Stable regimen was defined as on treatment for at least 3 months. Topical products without systemic absorption, over-the-counter and prescription pain medication or hormonal contraceptives (female participants) were acceptable at the investigator’s discretion.
4. Use of more than two tobacco/nicotine-containing or cannabis products (for example, two cigarettes) per month within 6 months before the first intraperitoneal administration (applicable only to HP cohorts 1, 2, 3, 4, 2b, 3b and 4b).
5. HIV infection, as shown by the presence of anti-HIV antibody (seropositive).
6. Seropositive for hepatitis B virus or hepatitis C virus (HCV) (HCV seropositivity required positive test for antibodies confirmed with positive test for HCV RNA).
7. Had uncontrolled hypertension, defined as blood pressure >170/100 mmHg at screening, confirmed by repeat.
8. A history of torsades de pointes, ventricular rhythm disturbances (for example, ventricular tachycardia or fibrillation), pathologic symptomatic bradycardia, 2nd degree or 3rd degree heart block, congenital long QT syndrome, prolonged QT interval due to medications or new elevation or depression in the part of an ECG immediately after the QRS complex and merging into the T wave (ST segment) or new pathologic inverted T waves or new pathologic Q waves on ECG that were deemed clinically significant in the opinion of the PI. Participants with a history of atrial arrhythmias could be discussed with the Sponsor Medical Monitor and the CRO Medical Monitor.
9. A family history of congenital long QT syndrome, Brugada syndrome or unexplained sudden cardiac death.
10. Symptomatic heart failure (per New York Heart Association guidelines), unstable angina, myocardial infarction, severe cardiovascular disease (ejection fraction <20%), transient ischemic attack (TIA) or cerebrovascular accident (CVA) within 6 months before study entry. For cohorts 7, 7b, 7c and 8, known stable (no clinically significant adverse change in last 6 months) cardiovascular or coronary artery disease was acceptable.
11. History of malignancy within the last 1 year except for basal cell carcinoma, squamous cell skin cancer, superficial bladder tumors or in situ cervical cancer. Participants with other treated malignancies who had no evidence of metastatic disease and more than 1 year without evidence of active malignancy could be entered after approval by the Sponsor Medical Monitor.
12. History of major surgery within 3 months of screening.
13. Regular use of alcohol within 1 month before the screening visit (that is, more than 14 units for women and 21 units for men per week (1 unit = 150 ml of wine, 360 ml of beer or 45 ml of 40% alcohol)).
14. Cardiac troponin (troponin I) above ULN at screening.
15. Recent (within 3 months) use of illicit drugs (such as cocaine, phencyclidine or and 3,4-methylenedioxy methamphetamine (MDMA)) or positive test for such drugs of abuse at screening. Participants who were on prescription medications that caused a positive result on urine drug screen were not excluded. Participants with a positive urine drug screen for cannabinoids were not excluded.
16. Use of an investigational agent or device within 30 days before dosing or current participation in an investigational study.
17. Any concomitant medical or psychiatric condition or social situation or any other situation that would make it difficult to comply with protocol requirements or put the participant at additional safety risk (for cohorts 5, 6, 7 and 8, stable diabetes mellitus based on principal investigator discretion, requiring or not requiring insulin, was not exclusionary.)
18. Had a history of clinically meaningful coagulopathy, bleeding diathesis, stroke or myocardial infarction within 6 months of baseline and/or concurrent anti-coagulant medication(s).
19. Participants with any of the following laboratory abnormalities:
  - International normalized ratio >1.5× ULN at screening
  - Platelets <100,000 per microliter at screening
20. Participants who were unable to return for all scheduled study visits.
21. Participants with any contraindications to MRI (cohort 5 only).
22. Donation or loss of whole blood (excluding the volume of blood that was to be drawn during the screening procedures of the study) before administration of the study treatment as follows: 50–499 ml of whole blood within 30 days, or more than 499 ml of whole blood within 56 days, before study treatment administration.

When laboratory value cutoffs were used for inclusion or exclusion, up to two repeat tests (after the initial screening test) were acceptable, and values from repeat testing could be used to determine study eligibility.

### Reporting summary

Further information on research design is available in the Nature Portfolio Reporting Summary linked to this article.

## Online content

Any methods, additional references, Nature Portfolio reporting summaries, source data, extended data, supplementary information, acknowledgements, peer review information; details of author contributions and competing interests; and statements of data and code availability are available at 10.1038/s41591-023-02494-2.

## Supplementary information

Supplementary Information Supplementary Tables 1–6, Supplementary Figs. 1–5, Study Protocol and Statistical Analysis Plan

Reporting Summary

## Figures and Tables

**Extended Data Table 1:**
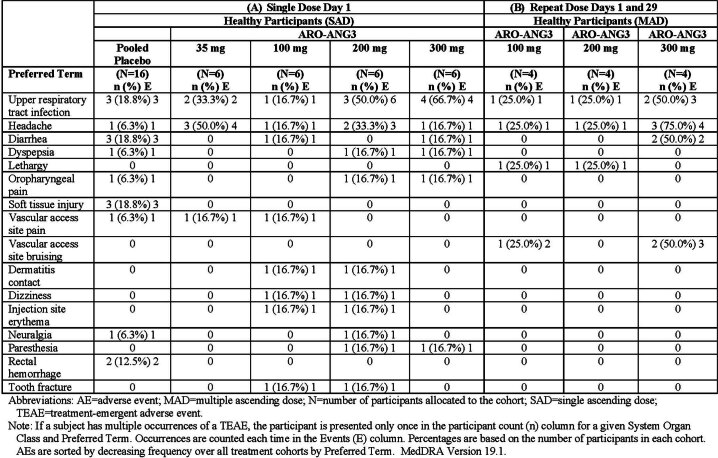
TEAEs, occurring in more than one participant, in HP SAD cohorts receiving placebo or 35 mg, 100 mg, 200 mg or 300 mg of ARO-ANG3 (A) and in HPs in cohorts receiving MADs of 100 mg, 200 mg or 300 mg of ARO-ANG3 (B)

**Extended Data Table 2:**
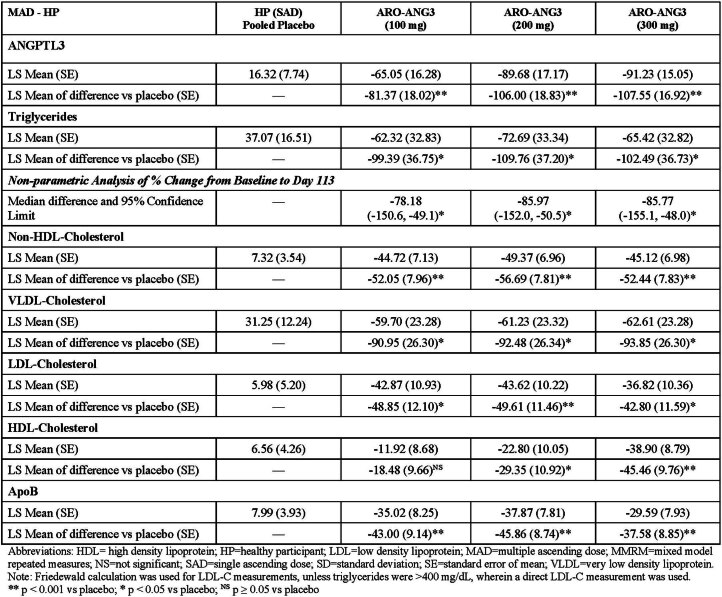
Pharmacodynamic measurements of lipid parameters, results from post hoc analysis: MMRM percent change from baseline to day 113 compared to placebo for ANGPTL3, TG, non-HDL-C, VLDL-C, LDL-C and HDL-C in HPs receiving MADs of ARO-ANG3

**Extended Data Table 3:**
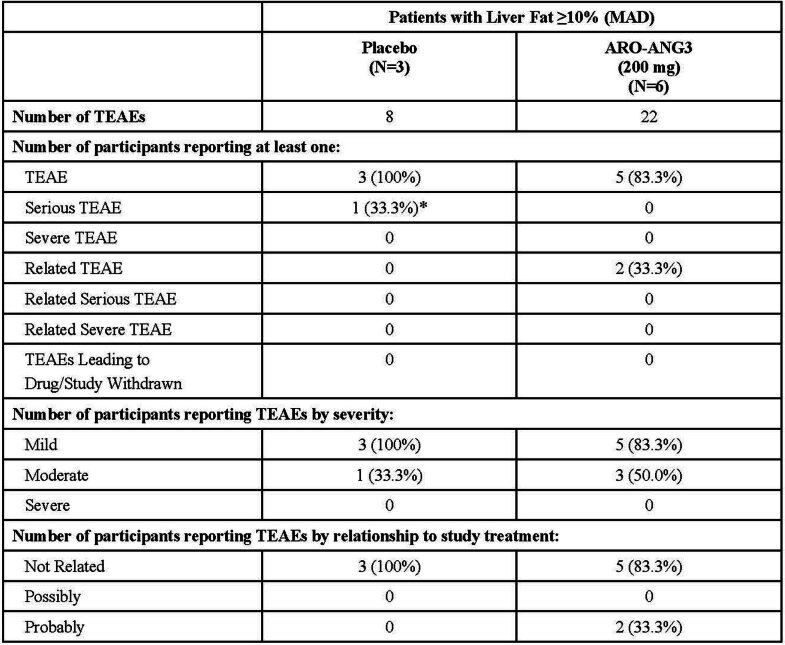
Overall summary of TEAEs in hepatic steatosis cohort receiving placebo or 200 mg of ARO-ANG3

**Extended Data Table 4:**
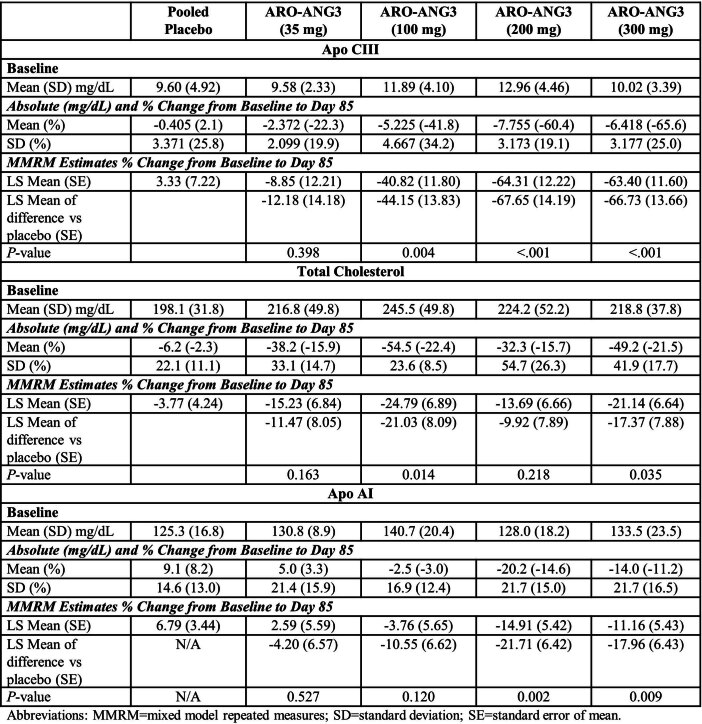
Pharmacodynamic measurements of lipid parameters: percent change from baseline at day 85 and MMRM analysis of change from baseline compared to placebo for Apo CIII, total cholesterol and Apo AI for HP SADs of 35 mg, 100 mg, 200 mg or 300 mg of ARO-ANG3

**Extended Data Table 5:**
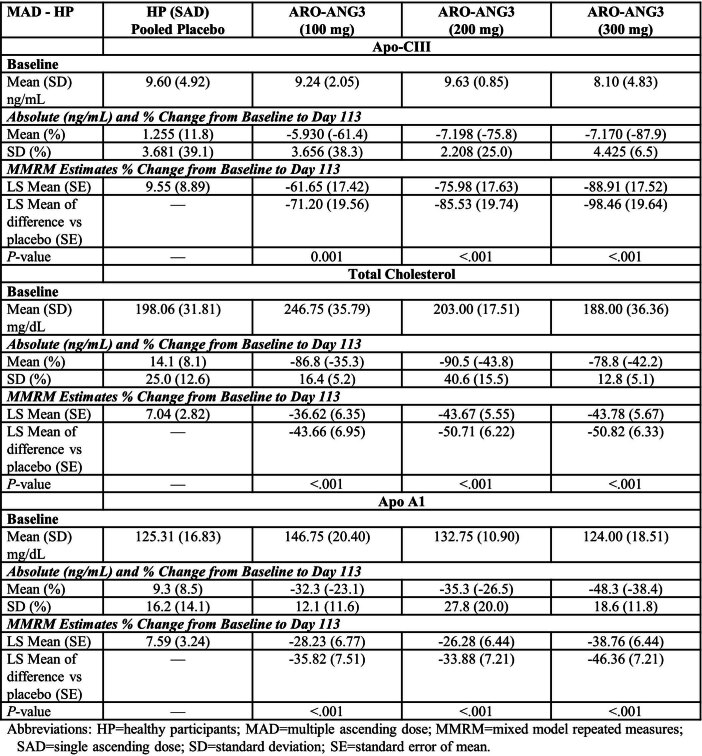
Pharmacodynamic measurements of lipid parameters: percent change from baseline at day 113 and MMRM analysis of change from baseline compared to placebo for Apo CIII, total cholesterol and Apo AI for HPs receiving escalating repeat doses of 100 mg, 200 mg or 300 mg of ARO-ANG3

**Extended Data Table 6:**
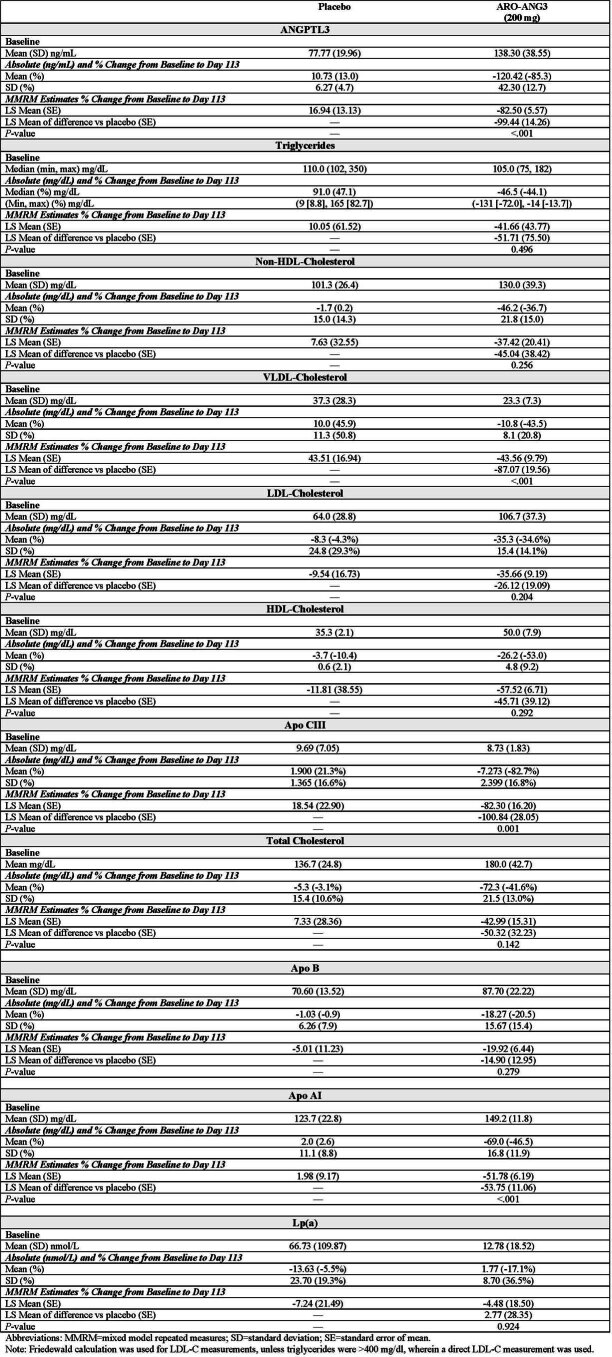
Pharmacodynamic measurements of lipid parameters: percent change from baseline at day 113 and percent change from baseline compared to placebo for ANGPTL3, TG, VLDL-C, non-HDL-C, LDL-C, HDL-C, Apo CIII, total cholesterol, Apo B, Apo AI and Lp(a) in participants with hepatic steatosis receiving repeat doses of 200 mg of ARO-ANG3

## Data Availability

Arrowhead Pharmaceuticals, Inc. is committed to sharing anonymized data from our clinical trials without compromising the privacy of trial participants. The Statistical Analysis Plan and the Final Study Protocol (Original and Final) will be made available upon publication. Data requests may be sent by email to info@arrowheadpharma.com. Analyses based on research proposals that demonstrate scientific merit will be considered. Arrowhead Pharmaceuticals, Inc. intends to share data only once a trial has completed and the product/indication has been approved at least in the United States and the European Union.
