# Supplementary material for: RNA interference targeting ANGPTL3 for triglyceride and cholesterol lowering: phase 1 basket trial cohorts
Source: Nat Med. 2023 Aug 25;29(9):2216–23. doi: 10.1038/s41591-023-02494-2 (PMC10504078; doi:10.1038/s41591-023-02494-2)
Supplement: Supplementary file 1 — Supplementary Tables 1–6, Supplementary Figs. 1–5, Study Protocol and Statistical Analysis Plan [file 41591_2023_2494_MOESM1_ESM.pdf]

nature medicine

Article

https://doi.org/10.1038/s41591-023-02494-2

RNA interference targeting ANGPTL3 for triglyceride and cholesterol lowering: phase 1 basket trial cohorts

In the format provided by the authors and unedited

Supplementary Information

LIST OF TABLES

Table S1: Dosing schedule 1

Table S2: Proportion of participants with defined changes in serum aminotransferases, total bilirubin, and platelet count in the healthy participant cohorts and the patient cohort with elevated liver fat content, after administration of ARO-ANG3 3

Table S3: Percent change in key lipid parameters (single-dose healthy participants) 4

Table S4: Percent change in key lipid parameters (repeat-dose healthy participants) 5

Table S5: Cohort Summary 7

Table S6: Dose Escalation Schedule 8

LIST OF FIGURES

Figure S1: Peak post-dose (A) and end of study (B) total bilirubin vs alanine aminotransferase (single ascending dose healthy participants, Cohorts 1, 2, 3, and 4) (Safety Population) 10

Figure S2: Peak post-dose (A) and end of study (B) total bilirubin vs alanine aminotransferase (hepatic steatosis participants) 12

Figure S3: Duplex sequence of RNAi molecule 13

Figure S4: Scatter plot of lipid parameters - percent change from baseline to Day 85 (single-dose healthy participants) 13

Figure S5: Scatter plot of lipid parameters - percent change from baseline to Day 113 (repeat-dose healthy participants) 15

Table S1: Dosing schedule

Cohort	Population	Blinding	# Participants	Dosing Schedule
1	HPs, TGs >100 mg/dL (1.13 mmol/L), LDL-C >70 mg/dL (1.81 mmol/L)	Double-blind	10 (6 active: 4 PBO)	35 mg Day 1 only
2	HPs, TGs >100 mg/dL (1.13 mmol/L), LDL-C >70 mg/dL (1.81 mmol/L)	Double-blind	10 (6 active: 4 PBO)	100 mg Day 1 only
3	HPs, TGs >100 mg/dL (1.13 mmol/L), LDL-C >70 mg/dL (1.81 mmol/L)	Double-blind	10 (6 active: 4 PBO)	200 mg Day 1 only
4	HPs, TGs >100 mg/dL (1.13 mmol/L), LDL-C >70 mg/dL (1.81 mmol/L)	Double-blind	10 (6 active: 4 PBO)	300 mg Day 1 only

Cohort	Population	Blinding	# Participants	Dosing Schedule
5	Liver fat based on MRI-PDFF ≥10%	Double-blind	9 (6 active: 3 PBO)	200 mg Day 1, 29

Abbreviations: HP=healthy participants; LDL-C=low density lipoprotein cholesterol; MRI-PDFF=magnetic resonance imaging proton density fat fraction; PBO=placebo; TG=triglycerides.

Post-dose changes in hepatic function and platelets that met certain thresholds during the study period are presented in Table S2. At peak post-dose values, 1 participant in the HP cohort and 1 participant in the hepatic steatosis cohort demonstrated a transient elevation in ALT to >3× ULN. However, at EOS ALT in both participants had returned towards baseline and was <3× ULN.

In the HP and hepatic steatosis cohorts, there were no adverse changes in hematology parameters that were considered clinically significant for any participant, regardless of dose level or treatment assignment. There were no TEAEs related to decreased platelet counts.

Table S2: Proportion of participants with defined changes in serum aminotransferases, total bilirubin, and platelet count in the healthy participant cohorts and the patient cohort with elevated liver fat content, after administration of ARO-ANG3

Category	Healthy Participants (SAD) Pooled Active (N=24) n (%)	Healthy Participants (MAD) Pooled Active (N=12) n (%)	Patients with Liver Fat ≥10% (MAD) ARO-ANG3 (200 mg) (N=6) n (%)
ALT >3x ULN	1 (4.2)	0 (0.0)	1 (16.7)
AST >3x ULN	0	0	0
Total Bilirubin >2x ULN	0	0	0
Platelets <150	1 (4.2)	0 (0.0)	0 (0.0)

Abbreviations: ALT=alanine aminotransferase; AST=aspartate aminotransferase; SAD=single ascending dose; MAD=multiple ascending dose; ULN=upper limit of normal

Table S3: Percent change in key lipid parameters (single-dose healthy participants)

	Pooled Placebo	ARO-ANG3 (35 mg)	ARO-ANG3 (100 mg)	ARO-ANG3 (200 mg)	ARO-ANG3 (300 mg)
**ANGPTL3**					
Baseline Mean (SD) ng/mL	81.13 (18.57)	88.23 (17.94)	94.93 (36.01)	90.57 (26.97)	82.87 (23.73)
Absolute (ng/mL) and % Change from Baseline to Day 85					
Mean (%)	3.08 (6.4)	-41.86 (-44.7)	-54.90 (-56.6)	-62.93 (-64.5)	-64.85 (-77.8)
SD (%)	24.53 (28.1)	17.63 (17.9)	24.68 (15.7)	37.35 (32.2)	22.53 (10.7)
**Triglycerides**					
Baseline Mean (SD) mg/dL	157.8 (161.6)	111.5 (46.9)	162.2 (76.0)	213.3 (95.6)	168.0 (101.7)
Median (min, max) mg/dL	110.0 (54, 727)	92.5 (79, 205)	147.0 (87, 292)	173.0 (117, 350)	133.5 (91, 369)
Absolute (ng/mL) and % Change from Baseline to Day 85					
Mean (%)	-20.4 (7.2)	-10.8 (-8.7)	-66.7 (-35.4)	-116.2 (-50.9)	-99.8 (-51.7)
SD (%)	110.8 (39.5)	27.0 (24.3)	59.1 (19.4)	82.1 (18.1)	98.1 (24.6)
Median (%)	-2.0 (-2.6)	-19.0 (-16.6)	-41.5 (-33.6)	-83.5 (-54.2)	-82.0 (-54.4)
**Non-HDL-Cholesterol**					
Baseline Mean (SD) mg/dL	155.0 (31.5)	169.0 (49.0)	197.7 (45.7)	183.2 (50.5)	173.3 (35.0)
Absolute (mg/dL) and % Change from Baseline to Day 85					
Mean (%)	-8.8 (-4.6)	-37.2 (-19.9)	-56.3 (-28.7)	-26.8 (-17.5)	-43.5 (-24.1)
SD (%)	20.2 (12.6)	33.9 (21.2)	19.5 (8.5)	49.0 (29.4)	38.8 (19.8)
**VLDL-Cholesterol**					
Baseline Mean (SD) mg/dL	24.0 (11.6)	22.3 (9.4)	32.3 (15.3)	42.5 (19.2)	33.8 (20.4)
Absolute (mg/dL) and % Change from Baseline to Day 85					
Mean (%)	1.5 (12.3)	-2.2 (-8.8)	-13.2 (-34.5)	-23.2 (-51.1)	-20.2 (-51.7)
SD (%)	7.2 (36.4)	5.3 (23.4)	12.0 (20.3)	16.3 (17.7)	19.8 (24.8)
**LDL-Cholesterol**					
Baseline Mean (SD) ng/mL	125.1 (37.0)	146.7 (41.8)	165.3 (36.8)	140.7 (52.2)	139.5 (32.5)

	Pooled Placebo	ARO-ANG3 (35 mg)	ARO-ANG3 (100 mg)	ARO-ANG3 (200 mg)	ARO-ANG3 (300 mg)
Absolute (ng/mL) and % Change from Baseline to Day 85					
Mean (%)	-6.7 (0.3)	-35.0 (-21.2)	-43.2 (-26.8)	-3.7 (4.1)	-23.3 (-12.9)
SD (%)	22.6 (26.8)	31.6 (22.4)	13.3 (9.5)	62.3 (61.1)	49.7 (35.4)
**HDL-Cholesterol**					
Baseline Mean (SD) mg/dL	43.1 (8.4)	47.8 (8.1)	47.8 (11.5)	41.0 (10.5)	45.5 (13.3)
Absolute (mg/dL) and % Change from Baseline to Day 85					
Mean (%)	2.6 (6.9)	-1.0 (-2.4)	1.8 (2.5)	-5.5 (-10.1)	-5.7 (-12.9)
SD (%)	4.8 (11.5)	5.1 (10.3)	12.5 (24.6)	8.4 (17.7)	9.7 (21.1)
**ApoB**					
Baseline Mean (SD) mg/dL	95.88 (17.86)	111.63 (28.77)	122.95 (30.63)	114.25 (29.09)	105.82 (17.54)
Absolute (mg/dL) and % Change from Baseline to Day 85					
Mean (%)	-2.36 (-1.0)	-24.76 (-21.9)	-29.25 (-23.1)	-6.00 (-6.7)	-14.43 (-12.4)
SD (%)	15.18 (15.8)	9.95 (10.9)	16.97 (9.9)	29.07 (27.1)	24.41 (21.5)

Abbreviations: ApoB=Apolipoprotein B, HDL=high density lipoprotein; LDL=low density lipoprotein; SD=standard deviation, VLDL=very low density lipoprotein.

Note: Friedewald calculation was used for LDL-C measurements, unless triglycerides were >400 mg/dl, wherein a direct LDL-C measurement was used.

Table S4: Percent change in key lipid parameters (repeat-dose healthy participants)

MAD – HP	HP (SAD) Pooled Placebo	ARO-ANG3 (100 mg)	ARO-ANG3 (200 mg)	ARO-ANG3 (300 mg)
**ANGPTL3**				
Baseline Mean (SD) ng/mL	81.13 (18.57)	98.75 (9.07)	107.08 (12.75)	91.35 (16.63)
Absolute (ng/mL) and % Change from Baseline to Day 113				
Mean (%)	18.84 (25.0)	-64.53 (-64.4)	-92.38 (-86.2)	-85.05 (-92.7)
SD (%)	27.55 (36.8)	23.41 (19.3)	14.80 (7.8)	17.85 (4.3)
**Triglycerides**				
Baseline Mean (SD) mg/dL	157.8 (161.6)	147.75 (60.09)	141.00 (26.52)	162.25 (114.20)
Median (min, max) mg/dL	110.00 (54, 727)	157.50 (72.00, 204.00)	144.00 (112.00, 164.00)	134.00 (57.00, 324.00)
Absolute (ng/mL) and % Change from Baseline to Day 113				
Mean (%)	-28.3 (31.4)	-94.5 (-61.1)	-93.5 (-66.0)	-120.0 (-66.5)

**MAD – HP**	**HP (SAD) Pooled Placebo**	**ARO-ANG3 (100 mg)**	**ARO-ANG3 (200 mg)**	**ARO-ANG3 (300 mg)**
SD (%)	72.9 (61.8)	49.1 (9.3)	25.5 (9.7)	102.3 (18.0)
Median (%)	-16.0 (23.8)	-99.5 (-62.2)	-86.0 (-63.6)	-96.5 (-72.0)
**Non-HDL-Cholesterol**				
Baseline Mean (SD) mg/dL	155.0 (31.5)	196.75 (43.98)	163.50 (18.86)	151.25 (35.42)
Absolute (ng/mL) and % Change from Baseline to Day 113				
Mean (%)	11.7 (8.6)	-81.0 (-41.4)	-82.0 (-49.0)	-64.8 (-42.9)
SD (%)	25.5 (16.1)	18.8 (5.5)	34.9 (14.6)	14.4 (3.0)
**VLDL-Cholesterol**				
Baseline Mean (SD) ng/mL	24.0 (11.6)	29.50 (12.12)	28.25 (5.62)	32.25 (23.11)
Absolute (ng/mL) and % Change from Baseline to Day 113				
Mean (%)	4.4 (30.2)	-19.0 (-61.5)	-18.8 (-66.1)	-23.8 (-65.3)
SD (%)	8.7 (63.0)	9.9 (9.5)	5.2 (9.6)	20.8 (19.9)
**LDL-Cholesterol**				
Baseline Mean (SD) ng/mL	125.1 (37.0)	167.25 (32.76)	135.25 (23.00)	119.00 (25.60)
Absolute (ng/mL) and % Change from Baseline to Day 113				
Mean (%)	3.8 (8.5)	-62.0 (-37.6)	-63.3 (-44.5)	-41.0 (-34.4)
SD (%)	25.6 (27.5)	12 (7.2%)	37.8 (18.1)	16.6 (9.6)
**HDL-Cholesterol**				
Baseline Mean (SD) mg/dL	43.1 (8.4)	50.00 (17.66)	39.50 (3.42)	36.75 (12.71)
Absolute (ng/mL) and % Change from Baseline to Day 113				
Mean (%)	2.5 (6.4)	-5.8 (-14.1)	-8.5 (-21.4)	-14.0 (-37.2)
SD (%)	5.5 (12.9)	7.6 (19.4)	9.6 (23.9)	7.7 (18.3)
**ApoB**				
Baseline Mean (SD) mg/dL	95.88 (17.86)	116.13 (29.60)	96.30 (11.63)	90.68 (21.18)
Absolute (ng/mL) and % Change from Baseline to Day 113				
Mean (%)	7.34 (9.1)	-36.55 (-30.7)	-38.63 (-39.0)	-25.60 (-28.4)
SD (%)	14.20 (16.2)	16.09 (7.6)	17.15 (13.0)	5.93 (4.0)

Abbreviations: ApoB=apolipoprotein B; HDL=high density lipoprotein; HP=healthy participants; LDL=low density lipoprotein; MAD=multiple ascending dose; SD=standard deviation VLDL=very low density lipoprotein.

Note: Friedewald calculation was used for LDL-C measurements, unless triglycerides were >400 mg/dl, wherein a direct LDL-C measurement was used.

Table S5: Cohort Summary

Cohort	Population	Blinding	# Participants	Dosing Schedule
1	HPs TGs > 100 mg/dL (1.13 mmol/L), LDL-C > 70 mg/dL (1.81 mmol/L)	Double-blind	10 (6 active: 4 PBO)	35 mg Day 1 only
2	HPs TGs > 100 mg/dL (1.13 mmol/L), LDL-C > 70 mg/dL (1.81 mmol/L)	Double-blind	10 (6 active: 4 PBO)	100 mg Day 1 only
2b	HPs	Open-label	4 active	100 mg Day 1, 29
3	HPs TGs > 100 mg/dL (1.13 mmol/L), LDL-C > 70 mg/dL (1.81 mmol/L)	Double-blind	10 (6 active: 4 PBO)	200 mg Day 1 only
3b	HPs	Open-label	4 active	200 mg Day 1, 29
4	HPs TGs > 100 mg/dL (1.13 mmol/L), LDL-C > 70 mg/dL (1.81 mmol/L)	Double-blind	10 (6 active: 4 PBO)	300 mg Day 1 only
4b	HPs	Open-label	4 active	300 mg Day 1, 29
5	HPs with baseline liver fat content based on MRI-PDFF ≥10%	Double-blind	9 (6 active: 3 PBO)	200 mg Day 1, 29
6	LDL-C > 70 mg/dL (1.81 mmol/L) on stable statin regimen	Double-blind	9 (6 active: 3 PBO)	200 mg Day 1, 29
7	Familial Hypercholesterolemia	Open-label	≤ 6 active	200 mg Day 1, 29
7b	Familial Hypercholesterolemia	Open-label	≤ 6 active	100 mg Day 1, 29
7c	Familial Hypercholesterolemia	Open-label	≤ 6 active	300 mg Day 1, 29
8	TGs ≥300 mg/dL (3.39 mmol/L) at Screening	Open-label	≤ 6 active	200 mg Day 1, 29
9	Familial Hypercholesterolemia Extension Cohort (have completed Cohort 7, 7b, or 7c)	Open-label	≤ 18 active	200 mg Day 113, 197, 281, 365

Abbreviations: LDL-C=low-density lipoprotein cholesterol; MRI-PDFF=magnetic resonance imaging proton density fat fraction; HP=healthy participants; PBO=placebo; TG=triglyceride.

Table S6: Dose Escalation Schedule

**Single Dose Healthy Participants (double blind in Cohorts 1, 2, 3, 4)**			**Multi-dose Patients (Double-Blind in Cohorts 5, 6, Open-label in 2b, 3b, 4b, 7, 7b, 7c, 8, 9)**
Cohort*	Dose (Day 1)	Day 8 safety evaluation	Dose Regimen
Cohort 1**	35 mg		NA
Cohort 2**	100 mg		NA
Cohort 3**	200 mg		NA
Cohort 4**	300 mg		NA
			Cohort 5***: 200 mg or PBO dosed on Day 1, 29
			Cohort 6***: 200 mg or PBO dosed on Day 1, 29
			Cohort 8***: 200 mg dosed on Day 1, 29
			Cohort 2b†: 100 mg dosed on Day 1, 29
			Cohort 3b†: 200 mg dosed on Day 1, 29
			Cohort 4b†: 300 mg dosed on Day 1, 29
			Cohort 7***: 200 mg dosed on Day 1, 29
			Cohort 7b†: 100 mg dosed on Day 1, 29
			Cohort 7c†: 300 mg dosed on Day 1, 29
			Cohort 9 ‡: 200 mg dosed on Days 113, 197, 281, 365

Abbreviations: DSC=Data Safety Committee; IEC=Independent Ethics Committee; PBO=placebo.
Notes: *Cohorts 1, 2, 3, and 4 used sentinel participants.
** Dose escalation to the next highest dose level or to multiple dosing occurred after cumulative safety data through Day 8 for Cohorts 1, 2, 3, and 4 had been evaluated by the DSC.
*** Screening or enrollment into Cohorts 5, 6, 7, and 8 did not occur until an amended protocol justifying the dose to be used in these cohorts had been approved by the IEC.
† No DSC vote was required to open Cohorts 2b, 3b, and 4b. These cohorts could enroll in parallel.
† No DSC vote was required to open Cohorts 7b and 7c. These cohorts could enroll in parallel.
‡ Participants from Cohorts 7, 7b, and 7c could elect to continue to receive up to four 200 mg doses administered approximately every 12 weeks. No DSC vote was required for a participant to roll over into Cohort 9.

Plots for total bilirubin versus alanine transaminase (ALT) at peak postdose values and at end of study (EOS) are presented in Figure S1A and Figure S1B, respectively, for SAD cohorts. One participant in the pooled placebo cohort and 1 participant in the ARO-ANG3 35 mg cohort (purple solid triangle) experienced a treatment-emergent adverse event (TEAE) associated with ALT elevations >2× and >3× the ULN, respectively; neither TEAE was considered treatment-related (**A**). At EOS, ALT in both participants had returned towards baseline and was <3× ULN (**B**). There were no Hy's Law cases.

Figure S1: Peak post-dose (A) and end of study (B) total bilirubin vs alanine aminotransferase (single ascending dose healthy participants, Cohorts 1, 2, 3, and 4) (Safety Population)

Post peak-dose

A

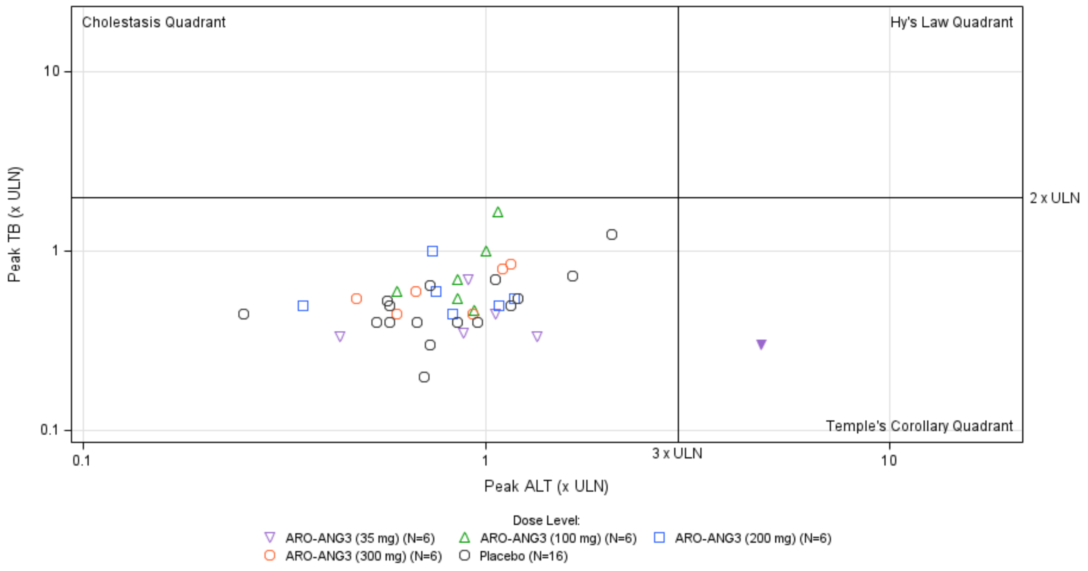

End of Study

B

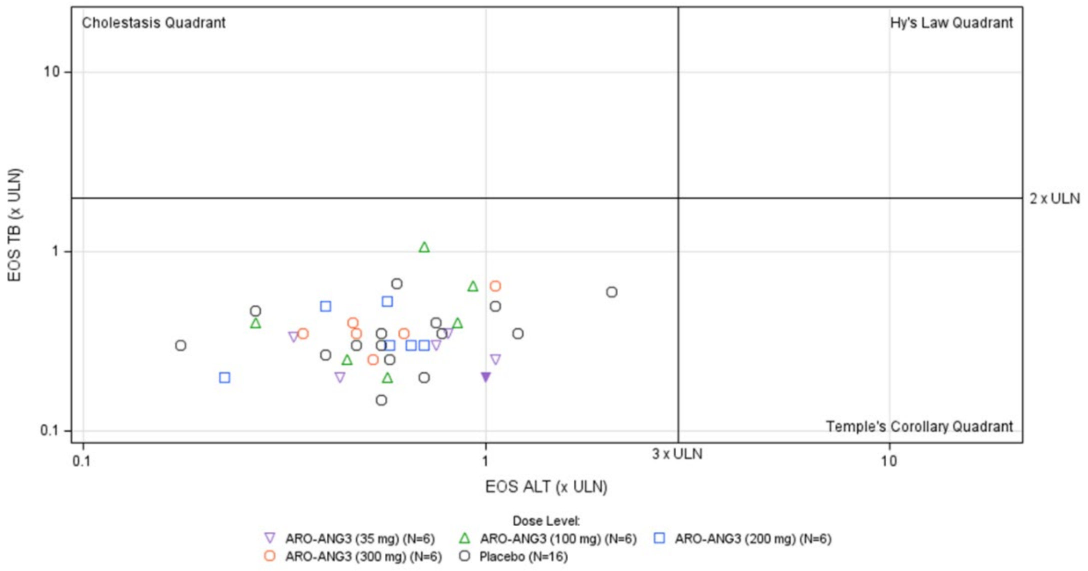

Abbreviations: ALT=alanine aminotransferase; SAD=single ascending dose; TB=total bilirubin; ULN=upper limit of normal.
Notes: Upper limit of normal is different for males and females.

Plots for total bilirubin versus ALT at peak postdose values and at EOS are presented in Figure S2A and Figure S2B, respectively, for the hepatic steatosis cohort. One participant

receiving 200 mg ARO-ANG3 (solid blue square) demonstrated a post-dose peak increase in ALT to >3× the ULN, which was transitory (**A**). At EOS, ALT had returned towards baseline and was <3× ULN (**B**). There were no Hy's Law cases.

Figure S2: Peak post-dose (A) and end of study (B) total bilirubin vs alanine aminotransferase (hepatic steatosis participants)

Peak Post-Dose

A

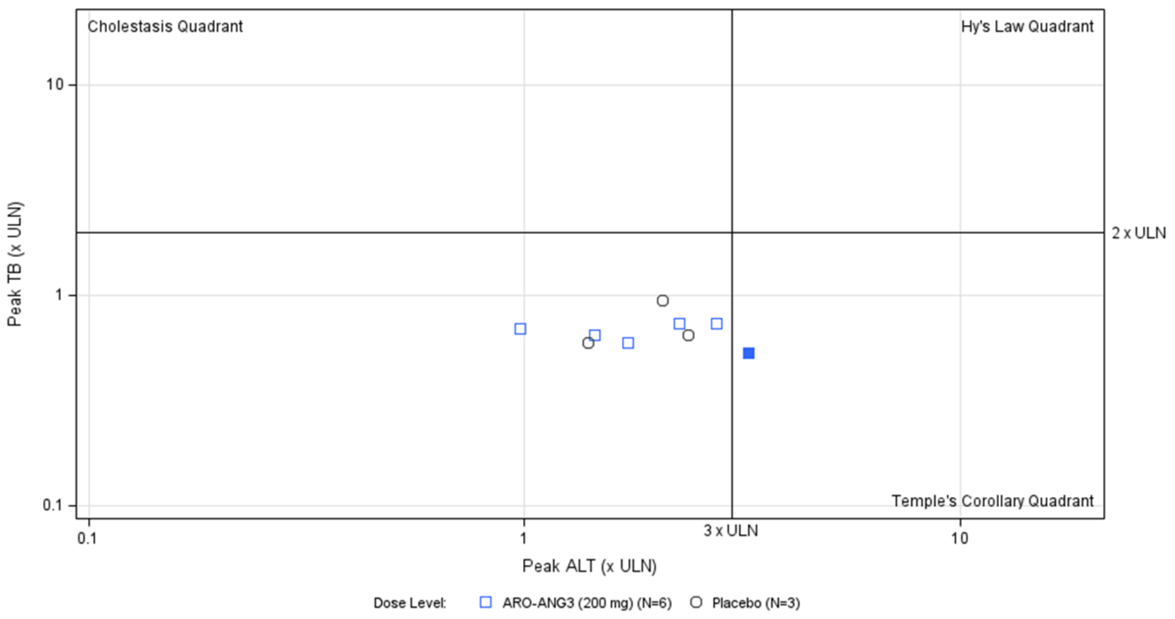

End of Study

B

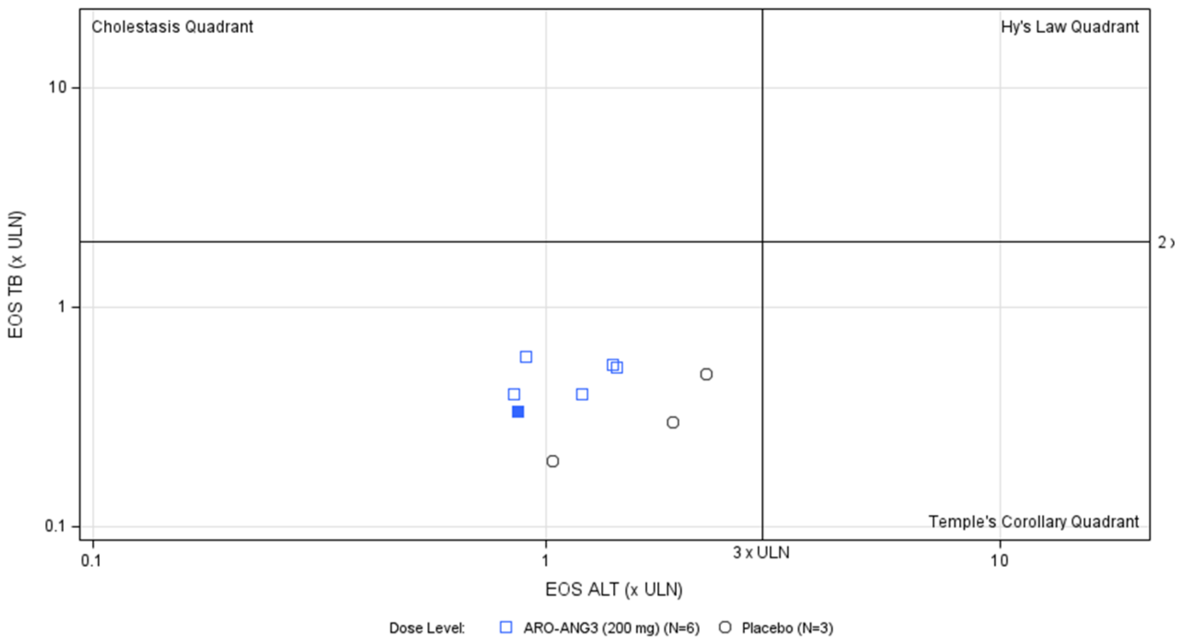

Abbreviations: ALT=Alanine aminotransferase; TB=Total bilirubin; ULN=Upper limit of normal.
Notes: Upper limit of normal is different for males and females.

Figure S3: Duplex sequence of RNAi molecule

Sense Strand (5′→3′): GCUCAACAUAUUUGAUCAGUA

Antisense (guide) Strand (5′→3′): UACUGAUCAAAUAUGUUGAGC

Figure S4 and Figure S5 scatterplots present within- participant analysis of the relationship between plasma ANGPTL3 lowering and changes in lipids (TGs, LDL-C, HDL-C, ApoB) for single ascending dose healthy participants and multiple ascending dose healthy participants, respectively.

For patients taking ARO-ANG3 treatment, a direct association between reduction in ANGPLT3 and reduction in triglycerides can be observed for healthy participants taking single dose and repeat doses. For healthy participants taking single dose of study drug, the correlation between reduction in ANGPLT3 and reduction in LDL-C is also significant. No obvious correlation is detected between reduction in ANGPLT3 and change in HDL-C or between reduction in ANGPLT3 and change in ApoB.

Figure S4: Scatter plot of lipid parameters - percent change from baseline to Day 85 (single-dose healthy participants)

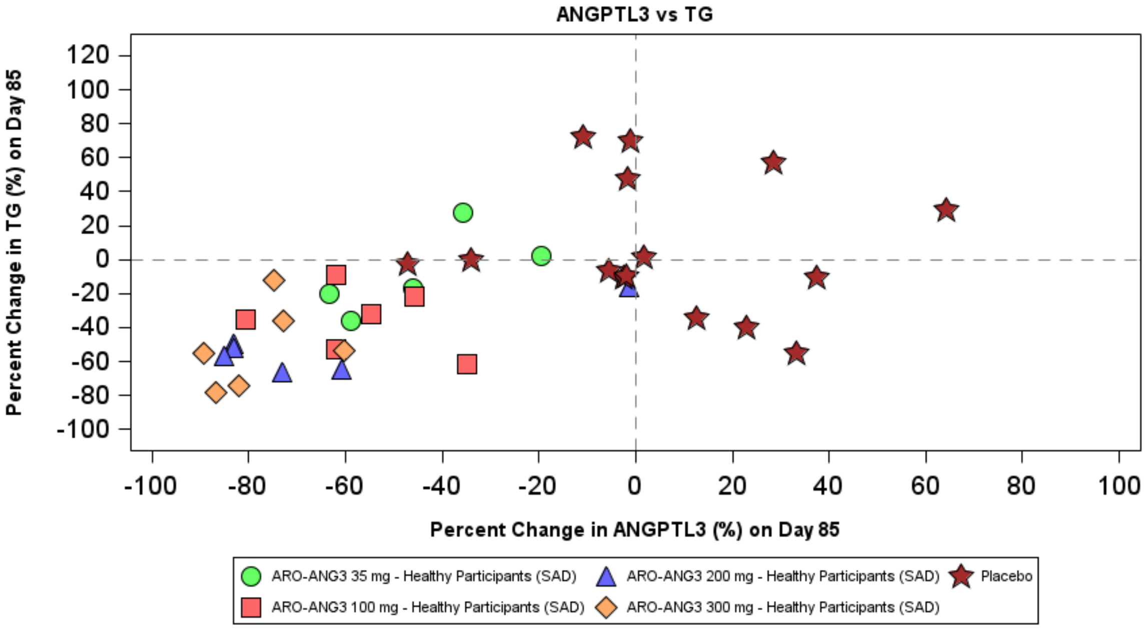

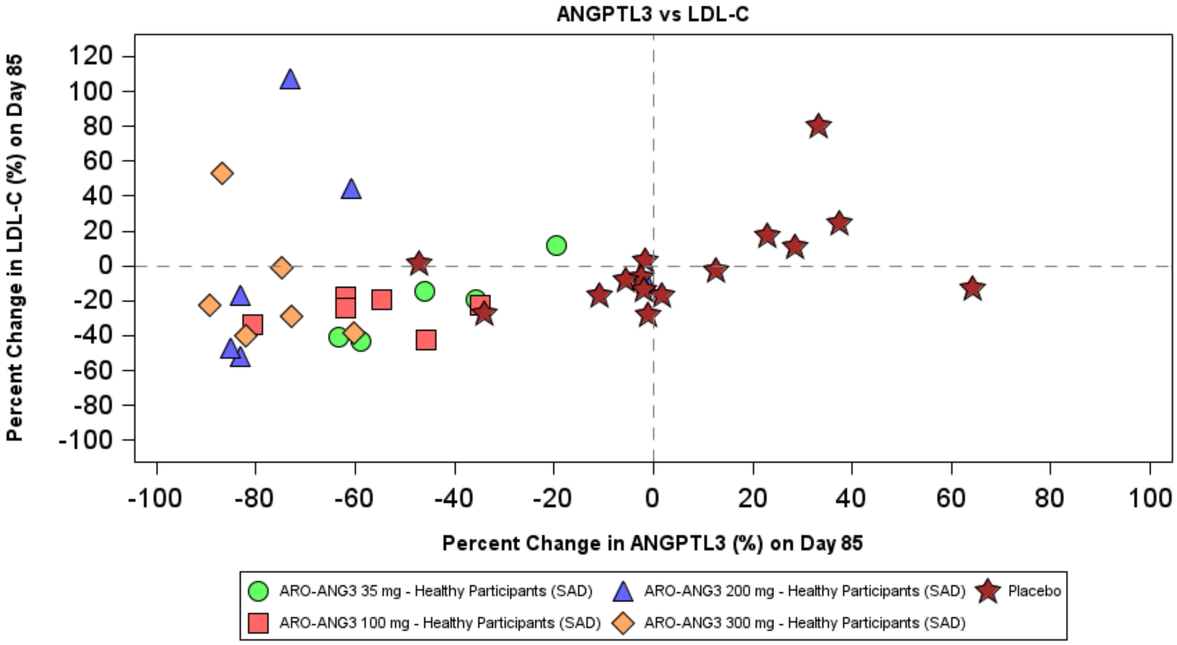
ANGPTL3 vs LDL-C
Percent Change in LDL-C (%) on Day 85
120
100
80
60
40
20
0
-20
-40
-60
-80
-100
-100
-80
-60
-40
-20
0
20
40
60
80
100
Percent Change in ANGPTL3 (%) on Day 85
ARO-ANG3 35 mg - Healthy Participants (SAD)
ARO-ANG3 200 mg - Healthy Participants (SAD)
Placebo
ARO-ANG3 100 mg - Healthy Participants (SAD)
ARO-ANG3 300 mg - Healthy Participants (SAD)

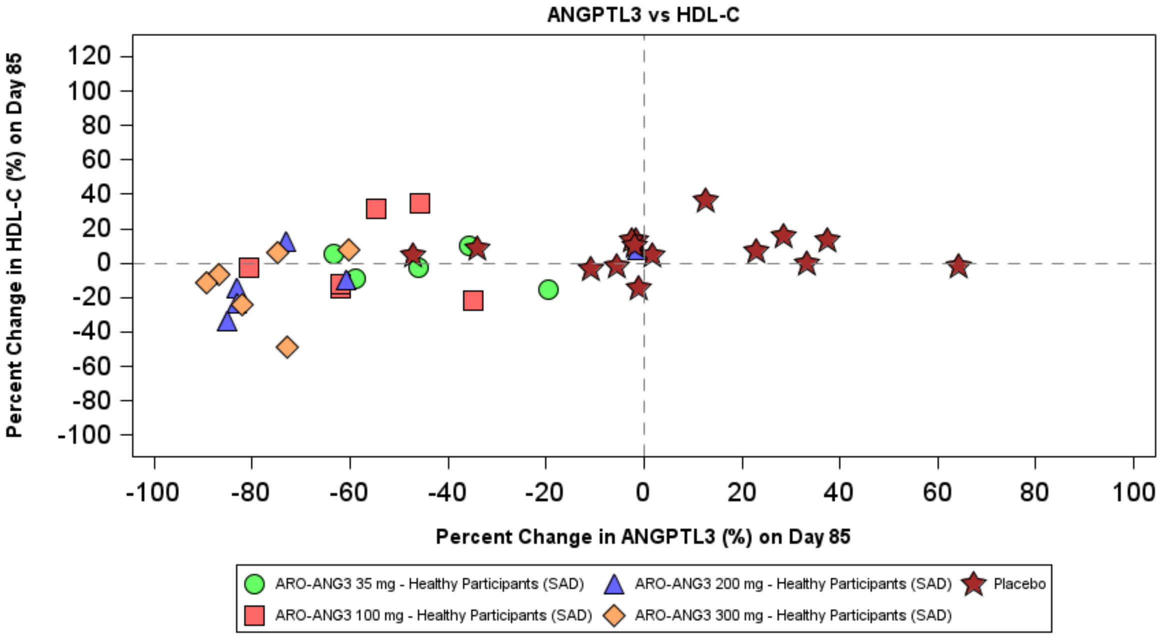
ANGPTL3 vs HDL-C
Percent Change in HDL-C (%) on Day 85
120
100
80
60
40
20
0
-20
-40
-60
-80
-100
-100
-80
-60
-40
-20
0
20
40
60
80
100
Percent Change in ANGPTL3 (%) on Day 85
ARO-ANG3 35 mg - Healthy Participants (SAD)
ARO-ANG3 200 mg - Healthy Participants (SAD)
Placebo
ARO-ANG3 100 mg - Healthy Participants (SAD)
ARO-ANG3 300 mg - Healthy Participants (SAD)

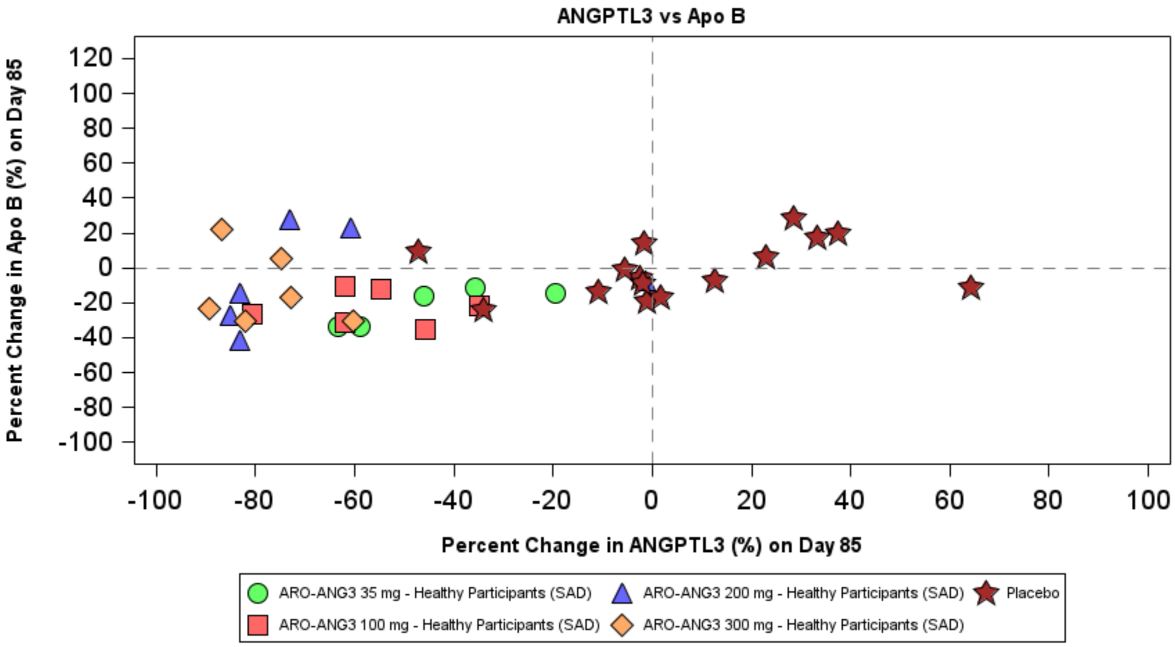

Figure S5: Scatter plot of lipid parameters - percent change from baseline to Day 113 (repeat-dose healthy participants)

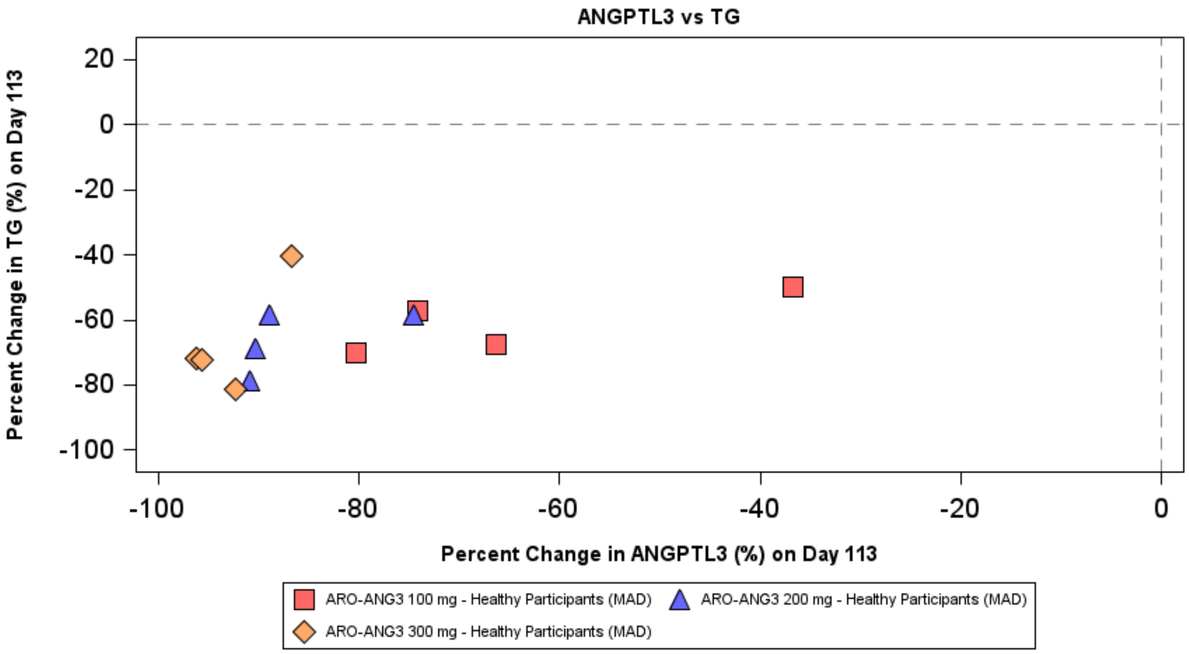

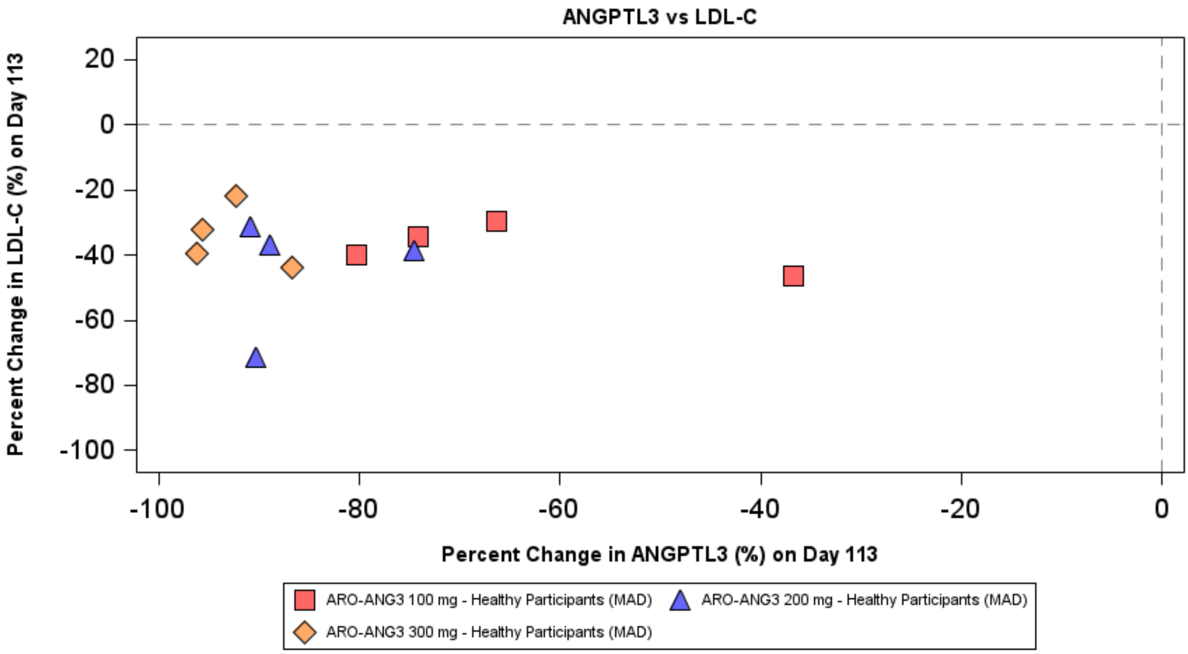
ANGPTL3 vs LDL-C
Percent Change in LDL-C (%) on Day 113
20
0
-20
-40
-60
-80
-100
-100
-80
-60
-40
-20
0
Percent Change in ANGPTL3 (%) on Day 113
ARO-ANG3 100 mg - Healthy Participants (MAD)
ARO-ANG3 200 mg - Healthy Participants (MAD)
ARO-ANG3 300 mg - Healthy Participants (MAD)

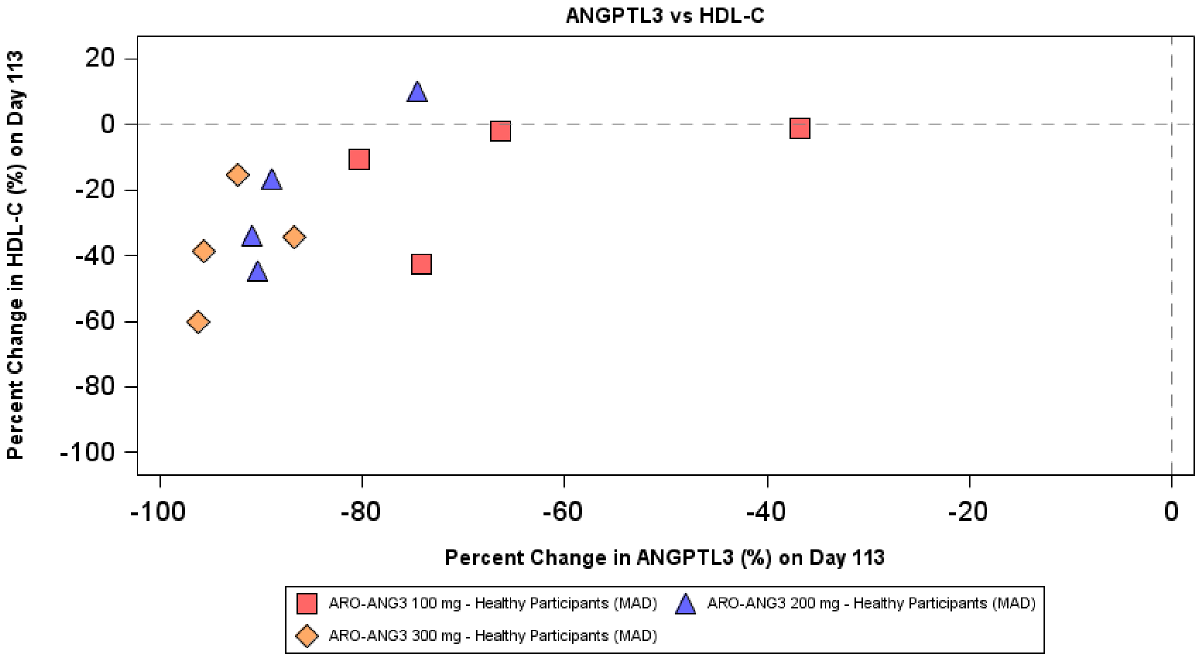
ANGPTL3 vs HDL-C
Percent Change in HDL-C (%) on Day 113
20
0
-20
-40
-60
-80
-100
-100
-80
-60
-40
-20
0
Percent Change in ANGPTL3 (%) on Day 113
ARO-ANG3 100 mg - Healthy Participants (MAD)
ARO-ANG3 200 mg - Healthy Participants (MAD)
ARO-ANG3 300 mg - Healthy Participants (MAD)

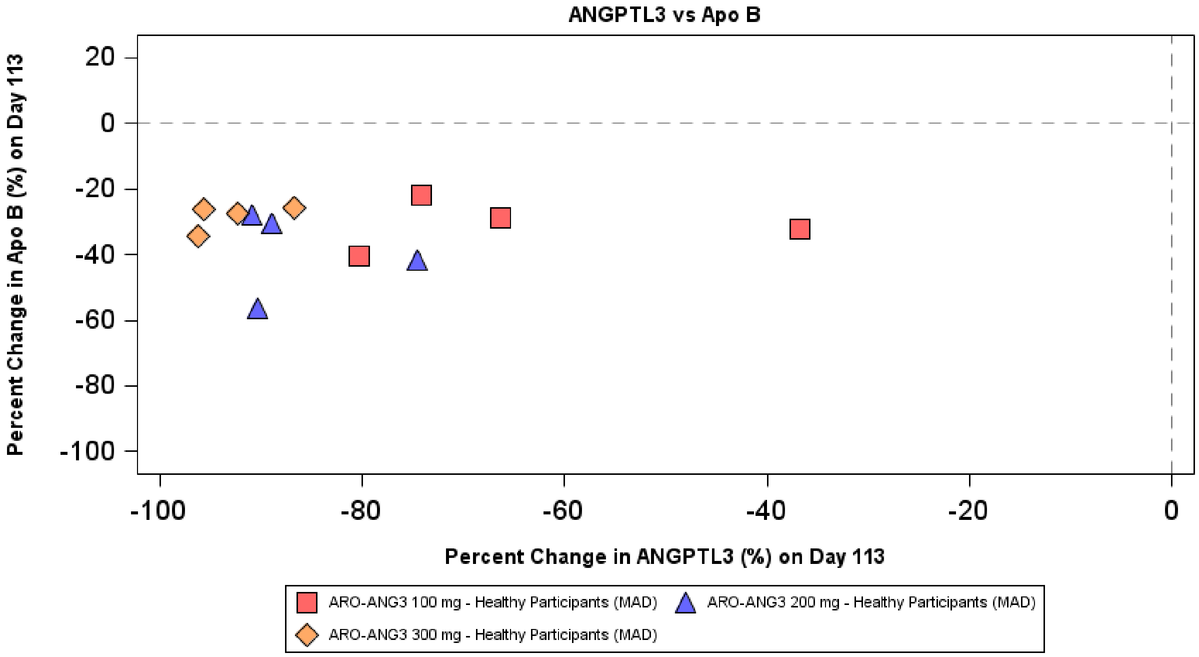
ANGPTL3 vs Apo B
Percent Change in Apo B (%) on Day 113
20
0
-20
-40
-60
-80
-100
-100
-80
-60
-40
-20
0
Percent Change in ANGPTL3 (%) on Day 113
ARO-ANG3 100 mg - Healthy Participants (MAD)
ARO-ANG3 200 mg - Healthy Participants (MAD)
ARO-ANG3 300 mg - Healthy Participants (MAD)

**PROTOCOL NUMBER:**	**AROANG1001**
**STUDY TITLE:**	**A Phase 1 Single and Multiple Dose Study to Evaluate the Safety, Tolerability, Pharmacokinetics and Pharmacodynamic Effects of ARO-ANG3 in Adult Healthy Volunteers and in Dyslipidemic Patients**
**DRUG (Active):**	**ARO-ANG3**
**ROUTE:**	**Subcutaneous Injection**
**STUDY DESIGN:**	**A Phase 1 Single and Multiple Dose Study to Evaluate the Safety, Tolerability, Pharmacokinetics and Pharmacodynamic Effects of ARO-ANG3 in Adult Volunteers and in Dyslipidemic Patients**
**SPONSOR:**	**Arrowhead Pharmaceuticals, Inc.**<br>**177 East Colorado Boulevard, Suite 700**<br>**Pasadena, CA 91105**<br>**Telephone: +1 626 304 3400 Facsimile: +1 626 304 3401**

Amendment 7 adds open-label extension Cohort 9 into which patients from Cohort 7, 7b and 7c may elect to enroll.

Confidential

Information contained in this protocol should not be disclosed, other than to those directly involved in the execution or ethical review of the study, without written authorization from Arrowhead Pharmaceuticals, Inc. It is, however, permissible to provide information to a volunteer to obtain consent.

1 PROTOCOL SYNOPSIS

**Study Title:** A Phase 1 Single and Multiple Dose Study to Evaluate the Safety, Tolerability, Pharmacokinetics and Pharmacodynamic Effects of ARO-ANG3 in Adult Healthy Volunteers and in Dyslipidemic Patients
**Study Number:** AROANG1001
**Phase:** Phase 1, First-in-Human
**Number of Sites:** One or more sites in Australia and New Zealand
**Study Treatments:**<br><br>There will be two study treatments; one active (Test Formulation) and one placebo (Reference Formulation).<br><br>Test Formulation:<br><br>The test formulation is active ARO-ANG3 Injection (also referred to as ARO-ANG3). The active pharmaceutical ingredient (API) contained in ARO-ANG3 is a synthetic, double-stranded, small interfering RNA (siRNA) duplex conjugated to an N-acetyl-galactosamine targeting ligand to facilitate hepatocyte delivery.<br><br>Reference Formulation:<br><br>The reference formulation is placebo (PBO): normal saline (0.9%) administered subcutaneously, volume matched to the corresponding ARO-ANG3 dose volume.
**Study Objectives:**<br><br>**Primary Objective:**<br><br>• To determine the incidence and frequency of adverse events possibly or probably related to treatment as a measure of the safety and tolerability of ARO-ANG3 using escalating single and multiple doses in healthy volunteers and multiple doses in dyslipidemic patients.<br><br>**Secondary Objectives:**<br><br>• To evaluate the single-dose and multi-dose pharmacokinetics of ARO-ANG3 in healthy volunteers.<br><br>• To determine the reduction in fasting serum ANGPTL3 from baseline in response to a single and multiple doses of ARO-ANG3 as a measure of drug activity in healthy volunteers and in response to multiple doses of ARO-ANG3 in dyslipidemic patients (all values drawn after at least 8 hour fast).

Exploratory Objectives:

- To evaluate the effect of single or multiple doses of ARO-ANG3 on change from baseline in fasting LDL-C, Total Cholesterol, non-HDL-C, HDL-C, VLDL-C, Triglycerides, Lp(a), apoB-48, apoB-100, apoC-III apoC-II, apoA-V, lipoprotein lipase mass (if feasible), hepatic lipase mass (if feasible), CETP mass (if feasible) and apoA-I (all values drawn after at least 8 hour fast).
- To evaluate the effect of single or multiple doses of ARO-ANG3 on changes from baseline in BMI.
- To evaluate the effect of single or multiple doses of ARO-ANG3 on changes from baseline in fasting serum blood glucose, C-peptide, hemoglobin A1C, GTT and fasting serum insulin.
- To evaluate the effect of multiple doses of ARO-ANG3 on change from baseline liver fat content using Magnetic Resonance Imaging (using MRI-PDFF) in Cohort 5 only.
- To evaluate the effect of multiple doses of ARO-ANG3 on change from baseline in post-prandial (post standardized high fat/high carbohydrate meal) serum TGs in specified cohorts.
- To evaluate excretion of ARO-ANG3 (full length and metabolites) and identify metabolites in plasma and urine in the multi-dose healthy volunteer cohorts.

Study Population/Patient Number: This study will be conducted in adult males and females, aged 18-65 years (up to age 70 for Cohorts 7, 7b, 7c,8 and 9 if otherwise healthy and at the discretion of the investigator) with BMI between 19.0 and 40.0 kg/m² and:

- Cohorts 1, 2, 3 and 4: All subjects will have fasting Screening triglycerides > 100 mg/dL (1.13 mmol/L) and fasting Screening LDL-C > 70 mg/dL (1.81 mmol/L) and not on any lipid lowering therapy. Each double-blind cohort will enroll ten (10) subjects (6 active: 4 PBO) with all cohorts planned to receive single escalating doses of ARO-ANG3 or PBO at escalating dose levels as per **Figure 1 and 2** of 35, 100, 200, and 300 mg.
- Cohort 5: Cohort is double-blind with up to 9 subjects (6 active and 3 PBO), all receiving multiple doses of ARO-ANG3 or PBO (6 active: 3 PBO). All subjects will have a liver fat fraction of ≥10% based on MRI-PDFF conducted at Screening.
- Cohort 6: Cohort is double-blind with up to 9 subjects (6 active and 3 PBO), all receiving multiple doses of ARO-ANG3 or PBO. All 9 subjects in Cohort 6 will be on a stable drug treatment regimen for elevated LDL-C including a statin for at least 6 months with fasting Screening LDL-C > 70 mg/dL (1.81 mmol/L).
- Cohort 7, 7b, 7c: Cohorts are open-label with up to 6 patients in each cohort with a diagnosis of heterozygous or homozygous familial hypercholesterolemia, defined as documented positive genetic test OR Dutch Lipid Clinic Network Score ≥ 8 with LDL-C > 100 mg/dL (2.59 mmol/L) despite standard of care therapy OR with LDL-C > 70 mg/dL (1.81 mmol/L) while on a PCSK-9 inhibitor OR with LDL-C > 70 mg/dL (1.81 mmol/L) in the presence of documented atherosclerotic cardiovascular disease. All subjects to receive multiple doses of ARO-ANG3. At End of Study for Cohorts 7, 7b and 7c, patients may elect to proceed with the End of Study visit or continue to receive up to four additional quarterly doses of ARO-ANG3 in Cohort 9.
- Cohort 8: Cohort is open-label with up to 6 patients with fasting serum triglycerides of at least 300 mg/dL (3.39 mmol/L). All subjects to receive multiple doses of ARO-ANG3.
- Cohort 9: Patients from Cohorts 7, 7b and 7c may elect to continue to receive up to four 200 mg doses administered approximately every 12 weeks (See Cohort 9 Schedule of Assessments).
- Cohorts 2b, 3b, 4b: Cohorts are open-label with 4 NHVs. Each open-label cohort will enroll four (4) subjects with all cohorts planned to receive multiple escalating doses of ARO-ANG3 at escalating dose levels as per **Figure 1 and 2** of 100, 200, and 300 mg. Cohorts 2b-4b will be enrolled in New Zealand only.

Figure 1: Cohort Summary

Cohort	Population	Blinding	# Subjects	Dosing Schedule
1	NHVs TGs > 100 mg/dL(1.13 mmol/L), LDL-C > 70 mg/dL (1.81 mmol/L)	Double-blind	10 (6 active: 4 PBO)	35 mg on Day 1 only
2	NHVs TGs > 100 mg/dL (1.13 mmol/L), LDL-C > 70 mg/dL(1.81 mmol/L)	Double-blind	10 (6 active: 4 PBO)	100 mg Day 1 only
2b	NHVs	Open-label	4 active	100 mg Day 1, 29
3	NHVs TGs > 100 mg/dL (1.13 mmol/L), LDL-C > 70 mg/dL(1.81 mmol/L)	Double-blind	10 (6 active: 4 PBO)	200 mg Day 1 only
3b	NHVs	Open-label	4 active	200 mg Day 1, 29
4	NHVs TGs > 100 mg/dL (1.13 mmol/L), LDL-C > 70 mg/dL (1.81 mmol/L)	Double-blind	10 (6 active: 4 PBO)	300 mg Day 1 only
4b	NHVs	Open-label	4 active	300 mg Day 1, 29
5	NHVs with liver fat on MRI-PDFF ≥ 10%	Double-Blind	9 (6 active: 3 PBO)	200 mg Day 1, 29

6	LDL-C > 70 mg/dL (1.81 mmol/L) on stable statin regimen	Double-Blind	9 (6 active: 3 PBO)	200 mg Day 1, 29
7	Familial Hypercholesterolemia	Open-label	≤ 6 active	200 mg Day 1, 29
7b	Familial Hypercholesterolemia	Open-label	≤ 6 active	100 mg Day 1, 29
7c	Familial Hypercholesterolemia	Open-label	≤ 6 active	300 mg Day 1, 29
8	TGs ≥ 300 mg/dL (3.39 mmol/L)	Open-label	≤ 6 active	200 mg Day 1, 29
9	Familial Hypercholesterolemia Extension Cohort (have completed Cohort 7, 7b or 7c)	Open-label	≤ 18 active	200 mg Days 113, 197, 281, 365, 200

A total of up to 94 subjects may be enrolled in the study (not including replacements).

Number of Doses per Treatment: Single dose (Cohort 1, 2, 3, 4) or up to two doses (Cohorts 2b, 3b, 4b, 5, 6, 7, 7b, 7c, 8) dosed once every 28 days. Cohort 9 will receive four doses dosed every 12 weeks.

Study Duration: For each subject in the study, the duration of the study clinic visits is approximately 25 weeks from screening to the Day 113 End-of-Study examination (not including 90-day post-last dose follow-up phone call). The full duration of the study for Cohort 9 participants is approximately 77 weeks.

Study Confinement: For all cohorts (Cohorts 1, 2, 2b, 3, 3b, 4, 4b) except cohorts 5, 6, 7, 7b, 7c, 8 and 9, clinical facility confinement will be approximately 3 days for first dose administration (Day -2 through 24-hour assessments) with discharge on Day 2. Cohorts 2b, 3b and 4b will be confined approximately 1.5 days at Day 29 (Day 29 through 24 hour PK sample collection.) Subjects will return to the clinical facility for outpatient visits per the Schedule of Assessments. There will be no planned confinement for cohorts 5, 6, 7, 7b, 7c, 8 and 9 as all visits will take place as outpatient.

Study Design/Methods:

Participants who have signed an EC approved informed consent form and have met all the protocol eligibility criteria during screening may be enrolled into the study in a double-blind or open label fashion depending on the cohort. Cohorts 1 through 4 will begin with administration of ARO-ANG3 or PBO to two sentinel participants (one ARO-ANG3, one PBO). Following the Day 3 evaluation in these participants, if there are no significant safety concerns, the remaining participants in the cohort will be treated at the discretion of the Principal Investigator (PI). Dosing of participants will be staggered by at least 30 minutes such that no two participants will be dosed simultaneously.

Dose levels by cohort are outlined in **Figures 1 and 2.** Cohorts 1 through 4 will enroll sequentially. Cohorts 5, 6, 7, and 8 may be opened after review of cumulative safety data from all previous cohorts including through Day 8 of Cohort 4. After review of such cumulative safety data by the DSC, an amended protocol justifying the dose for Cohorts 5, 6, 7 and 8 will be submitted to the EC for approval (See Protocol Section 4.8). Screening and enrollment in Cohorts 5-8 will not occur until this amended protocol is approved by the EC. These multi-dose patient cohorts may enroll in parallel after they are opened for enrollment by the Data Safety Committee (DSC) and after EC approval of the amended protocol.

In the dose escalation part of the study (Cohorts 1 through 4), dose escalation will require approval by the DSC based on all cumulative available safety data for prior cohorts, and through at least Day 8 of the current NHV cohort (i.e. cohorts 1 through 4). Based on available safety data through Day 8, the DSC will vote to approve opening for enrollment of the next planned cohort/dose level. DSC decisions will be based on all aggregate safety data available including all data available at least through Day 8 of the current cohort as shown in **Figure 2**. Escalation to the next highest dose level will proceed until the dose level of 300 mg is completed, or the trial is halted prematurely by the PI, DSC, or Sponsor due to safety or other reasons. All subjects who withdraw from the study prior to their End of Study visit, for reasons other than an adverse event, may be replaced.

Cohort 9 is only open to patients who have completed Cohorts 7, 7b and 7c. At End of Study for Cohorts 7, 7b and 7c, patients may elect to proceed with the End of Study visit or continue to receive up to four additional doses of ARO-ANG3 administered every 12 weeks in Cohort 9.

Clinical facility confinement will be approximately 3 days for first dose administration (Day -2 through 24-hour assessments) for Cohorts 1 through 4 and 2 hours on the dosing days for Cohorts 5 through 9. In addition, Cohorts 2b, 3b and 4b will also be confined approximately 1.5 days at Day 29 (Day 29 through 24 hour PK sample collection.) Blood samples will be drawn pre-dose on Day 1 for baseline measurements. Height and weight will be measured at Screening to calculate BMI and as otherwise specified in the Schedule of Assessments.

In double-blind cohorts, blinding will be preserved to the extent possible (or unless otherwise specified); however, treatment un-blinding will occur, at the PI's discretion, where deemed necessary for treatment of an AE or for a decision to be made regarding trial continuation. After all subjects in a cohort have completed the final planned study visit on Day 113 (not including the 90-day follow-up call), Sponsor may be unblinded at Sponsor's request but PI and study participants will remain blinded. For clarity, Cohort 5 may be unblinded to Sponsor on Day 113, prior to Day 168 MRI.

Sponsor **may** request an interim descriptive analysis of the change from baseline in ANGPTL3, apoC-III and other measured lipid parameters any time after all subjects planned for enrollment in each cohort have received at least one dose of ARO-ANG3 or PBO. This interim analysis is for the planning of future studies and will not impact the conduct of this study. Sponsor will remain blinded to all subject treatment assignments. Descriptive statistics (that do not inadvertently unblind the trial) for change from baseline in pharmacodynamic measures will be calculated for all active subjects per cohort and for a pooled PBO group by an unblinded statistician and provided to Sponsor. For any AEs occurring more than once, the frequency of AEs for a specific preferred term will be calculated for pooled active and pooled PBO groups in such a way not to inadvertently break the subject-blind of the trial.

Single and multiple doses of ARO-ANG3 will be evaluated in a sequential manner as shown in **Figure 2**.

Figure 2: Dose Escalation Schedule

Single Dose Healthy Volunteers (double blind in Cohorts 1, 2, 3, 4)	Multi-dose Patients (Double-Blind in Cohorts 5, 6, Open-label in 2b, 3b, 4b, 7, 7b, 7c, 8, 9)

Cohort*	Dose (Day 1)	Day 8 safety evaluation	Dose Regimen
Cohort 1**	35 mg		NA
Cohort 2**	100 mg		NA
Cohort 3**	200 mg		NA
Cohort 4**	300 mg		NA
			Cohort 5***: 200 mg or PBO dosed on Day 1, 29
			Cohort 6***: 200 mg or PBO dosed on Day 1, 29
			Cohort 8***: 200 mg dosed on Day 1, 29
			Cohort 2b†: 100 mg dosed on Day 1, 29
			Cohort 3b†: 200 mg dosed on Day 1, 29
			Cohort 4b†: 300 mg dosed on Day 1, 29
			Cohort 7***: 200 mg dosed on Day 1, 29
			Cohort 7b†: 100 mg dosed on Day 1, 29
			Cohort 7c†: 300 mg dosed on Day 1, 29
			Cohort 9 ‡: 200 mg dosed on Days 113, 197, 281, 365

*Cohorts 1, 2, 3, and 4 will use sentinel subjects.

** Dose escalation to the next highest dose level or to multiple dosing will occur after cumulative safety data through Day 8 for Cohorts 1, 2, 3, and 4 have been evaluated by the DSC

*** Screening and enrollment into Cohorts 5, 6, 7 and 8 may not occur until an amended protocol justifying the dose to be used in these cohorts has been approved by the EC.

† No DSC vote is required to open cohorts 2b, 3b and 4b. These cohorts may enroll in parallel.

† No DSC vote is required to open cohorts 7b, 7c. These cohorts may enroll in parallel.

‡ Patients from Cohorts 7, 7b and 7c may elect to continue to receive up to four 200 mg doses administered approximately every 12 weeks. No DSC vote is required for patient to roll over into Cohort 9.

Unblinding of healthy volunteer cohorts may occur at Sponsor discretion on a cohort by cohort basis after all subjects in a cohort have completed their last planned on-site study visit (Day 113 End of Study). Study participants as well

as sites will remain blinded. Additional intermediate dose cohorts may be added if approved by Sponsor, EC and by the DSC.

Adverse event monitoring

Safety assessments will include: AEs/SAEs, physical examinations, vital sign measurements (blood pressure, heart rate, temperature, and respiratory rate), ECGs, clinical laboratory tests, concomitant medications/therapy, and reasons for treatment discontinuation. Safety assessments will be performed at specified time points and prior to study completion.

The AE/SAE reporting period for an enrolled participant begins when the participant provides informed consent. Treatment-emergent AEs/SAEs are defined as those following study drug administration or a pre-existing condition exacerbated by study drug. All AEs that occur during the AE reporting period specified in the protocol must be reported to Arrowhead via electronic case report forms within approximately 48 hours. All SAEs that occur during the reporting period, in addition to reporting via electronic case report forms, must also be reported to Arrowhead via the SAE report form within 24 hours of awareness. All AEs/SAEs will be followed until resolution, until the condition stabilizes, until the event is otherwise explained, or until the participant is lost to follow-up. If the PI learns of any SAE, including a death, at any time after a patient has been discharged from the study, and he/she considers the event reasonably related to the investigational product, the PI will promptly notify the Sponsor. Laboratory abnormalities will be reported as AEs if considered clinically significant by the PI. Laboratory abnormalities not reported as AEs are not to be reported as Clinically Significant (CS) in the study database.

Treatment Stopping Rules:
Escalation to the next cohort will proceed according to the study design until the last cohort is completed, unless the trial is stopped early by the Data Safety Committee (DSC), PI or Sponsor. A decision to stop the trial early or discontinue drug in an individual subject or group of subjects **may** be indicated based on any of the following:

1. Two or more similar Serious Adverse Event (SAE, defined in Section 9.1) considered at least possibly related to ARO-ANG3.
2. One of the following abnormal results at least possibly related to ARO-ANG3:

    - Treatment emergent AST and/or ALT > 8X ULN which must be confirmed by repeat blood draw within 48 hours of initial results OR a treatment emergent AST or ALT >3X ULN with a total bilirubin >2X ULN which must be confirmed by repeat blood draw within 48 hours of initial results OR a treatment emergent AST or ALT >3X ULN with an INR > 1.5 (both of which must be confirmed on repeat) OR AST or ALT >3X ULN (which must be confirmed on repeat) with symptoms (e.g. nausea & vomiting, RUQ pain, fever, rash) or with eosinophilia. For Cohorts 5, 6, 7, 8 and 9 patients with NAFLD and/or dyslipidemia or on statins may have elevated ALT and/or ASL at baseline. For this reason, liver related stopping rules will follow those proposed by Chalasani et al., 2016 which are described in Appendix 1.

    - Two or more occurrences of treatment emergent platelet count < 70,000 per microliter which must be confirmed by repeat blood draw within 48 hours of initial results.

    - Two or more occurrences of treatment emergent serum creatinine increase of > 0.3 mg/dL (26.5 µmol/L) AND >50% increase from pre-dose baseline both of which must occur in the first 8 days of dosing and which must be confirmed by repeat blood draw within 48 hours of initial results.

Sponsor or PI can discontinue any subject at any time with or without DSC consultation. If such events (as described in #1, #2 above) occur and the subject is not discontinued from the study, the reason for not discontinuing the subject will be included in DSC meeting minutes. Including, but not limited to the events listed above, the DSC **may** pause the study to additional dosing or dose escalation to provide time to evaluate safety data and recommend the action to be taken, which may include, but is not limited to, one of the following:

3. Discontinuation of a subject or group of subjects from the study

4. The study is stopped immediately with no further dosing
5. The study will continue until the current cohort is completed
6. The study will continue, but the next dose escalation will be to a level midway between the current level and the next level specified in Section 6.3
7. The study will continue as planned

Study Assessments:

Safety Assessments:
Safety assessments will be performed at specified time points per the Schedule of Assessments and will include the following:

- Vital signs: Resting heart rate, semi-supine systolic/diastolic blood pressure, respiratory rate and temperature
- Clinical laboratory measurements (e.g., chemistry, hemoglobin A1C, hematology, coagulation and urinalysis)
- Resting ECG measurements (measured after participant is semi-supine for at least 3 minutes).
- At each visit, participants will be asked about concomitant medications/therapy and will be instructed to volunteer any information regarding AEs and SAEs that they may have experienced. Any known untoward event that occurs beyond the AE reporting period that the PI considers an SAE and possibly related to study treatment will be reported to Arrowhead.
- Injection site reactions (ISRs): Injection site reactions will be defined and graded as mild, moderate or severe based on clinical findings. ISRs will be photographed at time of reporting and at time of resolution.
- 90-day post-last dose pregnancy follow-up phone call.
- Stool occult blood test.

Pharmacodynamic assessments
Pharmacodynamic assessments will be performed at specified time points per the Schedule of Assessments and will include fasting serum ANGPTL3, fasting LDL-C, Total Cholesterol, non-HDL-C, HDL-C, VLDL-C, Triglycerides, Lp(a), apoB-48, apoB-100, apoC-III and apoA-I, liver fat content (%) based on MRI-PDFF, GTT, serum insulin, serum glucose, Hemoglobin A1C, apoC-II, C-peptide, apoA-V, lipoprotein lipase mass (if feasible), hepatic lipase mass (if feasible), CETP mass (if feasible). All serum tests will be completed after an 8-hour fast unless as otherwise specified. Results, percent change, and duration of response (when applicable) from baseline to 4 weeks (or longer as necessary) will be analyzed and summarized by dose cohort and treatment group. For lipid related, lipoprotein and serum pharmacodynamic assessments, baseline is defined as the pre-dose value obtained nearest to the first dose.

If a subject’s serum ANGPTL3 level has not returned to above 50% of baseline value by EOS then additional monthly follow up visits **may** be completed (per Sponsor discretion) until serum ANGPTL3 level is above 50% of baseline.

Immunogenicity:
For Cohorts 5, 6, 7, 7b, 7c and 8, blood samples for anti-drug antibodies testing will be collected at pre-dose, Day 57, and at the End of Study visit (Day 113) or at Early Termination as per Schedule of Assessments.

Pharmacokinetics:
Blood samples will be collected from each subject for pharmacokinetic analysis after Dose 1 (Cohorts 1, 2, 2b, 3, 3b, 4, 4b) and Day 29 (Cohorts 2b, 3b, 4b) per the Schedule of Assessments.

Excretion and Metabolism:

Urine collections will be performed after dose 1 (Cohorts 2b, 3b, 4b) for metabolic analysis after dose 1 and dose 2 (Day 29) per the Schedule of Assessments, along with spot checks between doses to measure elimination PK.

Data Analysis:

Screening, Compliance, Tolerability and Safety Data:
In general, safety analyses will be performed and the results summarized by cohort. Post-treatment safety assessments will be compared with measurements recorded at baseline. Treatment emergent AEs will be summarized using the latest version of the Medical Dictionary for Regulatory Activities (MedDRA) by System Organ Class (SOC) and Preferred Term (PT). The incidence and frequency of AEs, SAEs, related AEs, related SAEs, and AEs leading to discontinuation, will be summarized by cohort per SOC, PT, and severity. All Adverse Events will also be presented in listings. The duration of AEs will be determined and included in listings, along with the action taken and outcome. The incidence of laboratory abnormalities will be assessed using descriptive summary statistics and shift tables. Vital sign measurements will be summarized at each scheduled time point using descriptive statistics. Abnormal physical examination findings will be summarized by time point and presented in subject listings. ECG parameters, changes from baseline, and qualitative assessments will be summarized. Pregnancy and FSH test results will be listed separately by time point.

Safety population: All participants that received at least one dose of study treatment.

Pharmacodynamic analysis:
Data will be summarized by cohort as applicable for the following: Serum ANGPTL3, LDL-C, Total Cholesterol, non-HDL-C, HDL-C, VLDL-C, Triglycerides, Lp(a), apoB-48, apoB-100, apoC-III, apoA-I, changes in % liver fat based on MRI-PDFF, changes in serum insulin, changes in serum glucose, changes in GTT. For lipid related, lipoprotein and serum pharmacodynamic assessments, baseline is defined as the pre-dose value obtained nearest to the first dose.

- Percent change from Day 1 pre-dose baseline to nadir for each serum marker
- Duration of response from nadir back to above 30% of baseline (if available by EOS) for each serum marker
- Absolute and relative change in % liver fat from pre-dose versus post-dose measurements

Descriptive statistics of biomarker (Serum ANGPTL3, LDL-C, Total Cholesterol, non-HDL-C, HDL-C, VLDL-C, Triglycerides, Lp(a), apoB-100, apoB-48, apoC-III, apoA-I, serum glucose, serum insulin, Hemoglobin A1C, apoC-II, C-peptide, apoA-V, lipoprotein lipase mass (if feasible), hepatic lipase mass (if feasible), CETP mass (if feasible), GTT, % liver fat based on MRI-PDFF) changes will include mean, median SD, minimum, and maximum. Additional details will be provided in the statistical analysis plan.

If a subject's serum ANGPTL3 level has not returned to above 50% of baseline value by EOS then additional monthly follow up visits **may** be completed (per Sponsor discretion) until serum ANGPTL3 level is above 50% of baseline. The sampling at these optional follow-up visits would include ANGPTL3 measurements, along with lipid parameters described (LDL-C, HDL-C and Triglycerides).

Pre-specified separate analysis will be performed for subjects by cohort.

Pharmacokinetics:
Plasma concentrations of ARO-ANG3 product constituents will be used to calculate the following PK parameters: maximum observed plasma concentration (C_{max}), area under the plasma concentration time curve (AUC) from time 0 to 24 hours (AUC_{0-24}), AUC from time 0 extrapolated to infinity (AUC_{inf}), and terminal elimination half-life ($t_{½}$). Pharmacokinetic parameters will be determined using non-compartmental methods. Descriptive statistics of PK parameters will include mean, standard deviation (SD), coefficient of variation, median, minimum, and maximum. PK results will be analyzed for dose proportionality, and sex differences.

Urine concentrations of ARO-ANG3 product constituents will be used to calculate the following PK parameters: Renal clearance rate (CL_R), elimination half-life. Pharmacokinetic parameters will be determined using non-

compartmental methods. Descriptive statistics of PK parameters will include mean, standard deviation (SD), coefficient of variation, median, minimum, and maximum. PK results will be analyzed for dose proportionality, and sex differences. Urine samples will also be used to identify metabolites of full-length parent ARO-ANG3.

PK population: All subjects that received at least one dose of active study treatment (ARO-ANG3).

<u>Immunogenicity (Anti-Drug Antibodies):</u>
Changes from assay negative to positive will be summarized by dose and number of doses administered. Descriptive statistics of immunogenicity parameters will include mean, SD, minimum, and maximum.

Additional details will be provided in the statistical analysis plan.

Table 1a: Cohorts 1-4 Single Dose

Assessment	Screen (Days -60 to -1)	Day -2 or -1 Confine		Day 1	Day 2 Discharge	Day 3	Day 8, 15, 22	Day 29, 43	Day 57, 71, 85, 99	Day 91	Day 113 EOS	Early Term
Informed Consent	X		RANDOMIZE									
Eligibility Criteria	X	X										
Body Mass Index	X										X	X
Demographics	X											
Medical History	X	X*										
Drug Screen	X	X										
Alcohol Breath Test	X	X										
Hepatitis/HIV Serology Screen	X											
Physical Exam[1]	X	X*			X	X	X	X	X		X	X
FSH	X[9]											
Pregnancy test (at Screening and pre-dose on dosing days)	X[6]			X[10]							X	X
ECG	X			X[2]	X							X
Vital Signs (BP, temp, RR, heart rate)	X			X[4]	X	X	X	X	X		X	X
Clinical Labs (heme, coag, chem, serum glucose, lipase, serum insulin, hemoglobin A1C, C-peptide, UA)	X	X		X[8]	X	X	X	X	X		X	X
GTT (may be completed Day -2 to Day -1 after 8 hour overnight fast)		X							X[13]			
Post-prandial TGs (may be completed Day -2 to Day -1 after 8 hour overnight fast)		X							X[14]			
Serum ANGPTL3, apoC-III, LDL-C, VLDL-C, HDL-C, Lp(a), Triglycerides, Total cholesterol, Non-HDL-cholesterol, ApoB-100, ApoB-48, ApoA-I (pre-dose on dosing days)	X			X		X	X	X	X		X	X
PK[3]				X	X	X						

Lipid metabolic genotype (if scientifically warranted) See Appendix 2				X[17]								
Concomitant Meds/Therapies	X	X		X	X	X	X	X	X		X	X
Meals[5]		X		X	X				X[15]			
Adverse Events[7]		X		X	X	X	X	X	X		X	X
Study Treatment				X								
Pregnancy F/U call										X[12]		
Stool occult blood test[16]		X						X			X	

* Repeat if > 2 weeks from Screening

1. Symptom-directed PEs to be performed by visit as necessary.
2. ECGs: Measured pre-dose and at 1 and 2 hours post-dose; more frequently per hour if necessary. Performed prior to other invasive procedures.
3. PK: (plasma) Blood samples collected 0 (pre-dose), 15 min, 0.5, 1, 2, 3, 6, 9, 12, 18, 24 & 48 hours post-dose on Day 1
4. Vitals: Measured pre-dose and at 5 min, 0.5, 1, 2, 3, and 6 hours post-dose on Day 1.
5. Meals: Lunch, Dinner, and snack(s) on Day 1 aligned with fasting requirements stated in section 7.5.
6. Urine pregnancy test for females of childbearing potential only.
7. AE/SAE data capture begins from time of informed consent.
8. Clinical Chemistry, Hematology, Coagulation, Lipase, hemoglobin A1C and Urinalysis pre-dose only.
9. Performed for females not of childbearing-potential to confirm postmenopausal status.
10. Pre-dose on dosing days.
12. Pregnancy Follow-Up phone call: 90 days post last dose ± 5 days.
13. Complete GTT post-dose on Day 85 only ±3 days after an 8 hour overnight fast.
14. On Day 85 ±3 after an overnight 8 hour fast, serum triglycerides measured 2 hours after a high fat/high carbohydrate standardized meal. Post-dose GTT and post-prandial TG assessment must be completed on separate days within the Day 85 window.
15. Provide high fat/high carbohydrate standardized meal after 8 hour fast, 2 hours prior to post-prandial TG measurement on Day 85 ±3 days.
16. Evaluate pre-dose upon confinement on Day -2, then on Day 43 and 113.
17. Lipid metabolic genetic analysis may be drawn on Day 1 or any time after Day 1 through EOS.

Table 1b: Cohort 5: Two Q28 Day doses with MRI-PDFF before and after dosing

Assessment	Screen (Days -60 to -1)	Day 1	Day 2	Day 3	Day 8, 15, 22	Day 29	Day 43, 57, 71, 85, 99	Day 113 EOS	Day 119	Day 168 (± 14 days)	Early Term
Informed Consent	X										
Eligibility Criteria	X	X									
Body Mass Index	X							X			X
Demographics	X										
Medical History	X	X*									
Drug Screen	X	X									
Alcohol Breath Test	X	X									
Hepatitis/HIV Serology Screen	X										
Physical Exam[1]	X	X*	X	X	X	X	X	X			X
FSH	X[7]										
Pregnancy test (at Screening and pre-dose on dosing days)	X[4]	X[8]				X[8]		X			X
ECG	X	X[2]	X			X[2]		X			X
Vital Signs (BP, temp, RR, heart rate)	X	X[3]	X	X	X	X[3]	X	X			X
Clinical Labs (heme, coag, chem, hemoglobin A1C, serum insulin, C-peptide, lipase, serum glucose, UA (pre-dose on dosing days)	X	X[6]	X	X	X	X[6]	X	X			X
MRI (may be completed Day -14 to Day -1)	X[9]						X[9]			X[9]	
GTT (may be completed Day -7 to Day -1 after 8 hour overnight fast), optional in patients with diabetes mellitus	X						X[10]				
Post-prandial TGs (may be completed Day -7 to Day -1 after 8 hour overnight fast)	X						X[12]				

Serum ANGPTL3, apoC-III, LDL-C, VLDL-C, HDL-C, Lp(a), Triglycerides, Total cholesterol, Non-HDL-cholesterol, ApoB-100, ApoB-48, ApoA-I, (pre-dose on dosing days)	X	X		X	X	X	X	X			X
apoC-II, apoA-V, lipoprotein lipase mass (if feasible), hepatic lipase mass (if feasible), CETP mass (if feasible)[14]		X					X	X			X
Anti-drug antibodies[14]		X					X	X			X
Lipid metabolic genotype (if scientifically warranted) See Appendix 2		X[15]									
Concomitant Meds/Therapies	X	X	X	X	X	X	X	X			X
Meals							X[13]				
Adverse Events[5]		X	X	X	X	X	X	X			X
Study Treatment		X				X					
Pregnancy F/U call									X[11]		

* Repeat if > 2 weeks from Screening
1. Symptom-directed PEs to be performed by visit as necessary.
2. ECGs: Measured pre-dose and at 1 and 2 hours post-dose; more frequently per hour if necessary. Performed prior to other invasive procedures.
3. Vitals: Measured pre-dose and at 5 min, 0.5, 1, 2 hours post-dose on dosing days.
4. Urine pregnancy test for females of childbearing potential only.
5. AE/SAE data capture begins from time of informed consent.
6. Clinical Chemistry, Lipase, Hemoglobin A1C, Hematology, Coagulation and Urinalysis pre-dose only
7. Performed for females not of childbearing potential to confirm postmenopausal status
8. Pre-dose
9. Complete pre-dose between Day -14 and Day -1.MRI-PDFF on post-dose Day 71 (±3 days) and 168 (± 14 days).
10. Complete GTT post-dose on Day 85 only ±3 days
11. Pregnancy Follow-Up phone call: 90 days post last dose ± 5 days
12. On Day 85 ±3 days after an overnight 8 hour fast, serum triglycerides measured 2 hours after a high fat/high carbohydrate standardized meal.
13. Provide high fat/high carbohydrate standardized after 8 hour fast, 2 hours prior to post-prandial TG measurement on Day 85 ±3 days.
14. Pre-dose on Day 1; also taken on Day 57 and Day 113 or early termination
15. Lipid metabolic genetic analysis may be drawn on Day 1 or any time after Day 1 through EOS.

Table 1c: Cohort 6, 7, 7b, 7c, and 8: Two Q28 day doses in subjects on stable statin regimen, FH subjects and subjects with triglycerides ≥ 300 mg/dL (3.39 mmol/L)

Assessment	Screen (Days -60 to -1)	Day 1	Day 2	Day 3	Day 8, 15, 22	Day 29	Day 43, 57 71, 85, 99	Day 113 EOS[16]	Day 119	Early Term
Informed Consent	X									
Eligibility Criteria	X	X								
Body Mass Index	X							X		X
Demographics	X									
Medical History	X	X*								
Drug Screen	X	X								
Alcohol Breath Test	X	X								
Hepatitis/HIV Serology Screen	X									
Physical Exam[1]	X	X*	X	X	X	X	X	X		X
FSH	X[8]									
Pregnancy test (at Screening and pre-dose on dosing days)	X[5]	X[9]				X[9]		X		X
ECG	X	X[2]	X			X[2]		X		X
Vital Signs (BP, temp, RR, heart rate)	X	X[4]	X	X	X	X[4]	X	X		X
Clinical Labs (heme, coag, chem, hemoglobin A1C, serum glucose, C-peptide, serum insulin, lipase, UA (pre-dose on dosing days)	X	X[7]	X	X	X	X[7]	X	X		X
**Cohort 8 Only:** GTT (may be completed Day -7 to Day -1 after 8 hour overnight fast), optional in patients with diabetes mellitus	X						X[11]			
**Cohort 8 Only:** Post-prandial TGs (may be completed Day -7 to Day -1 after 8 hour overnight fast).<br>**NOT** to be completed in patients with history of or at risk for post-prandial hypertriglyceridemia related abdominal pain or pancreatitis.	X						X[12]			

Serum ANGPTL3, apoC-III, LDL-C, VLDL-C, HDL-C, Lp(a), Triglycerides, Total cholesterol, Non-HDL-cholesterol, ApoB-100, ApoB-48, ApoA-I, (pre-dose on dosing days)	X	X		X	X	X	X	X		X
apoC-II, apoA-V, lipoprotein lipase mass (if feasible), hepatic lipase mass (if feasible), CETP mass (if feasible)[14]		X					X	X		X
Anti-drug antibodies[14]		X					X	X		X
Concomitant Meds/Therapies	X	X	X	X	X	X	X	X		X
**Cohort 8 Only**: Meals							X[13]			
Adverse Events[6]		X	X	X	X	X	X	X		X
Study Treatment		X				X				
Lipid metabolic genotype (if scientifically warranted) See Appendix 2		X[15]								
Pregnancy F/U call									X[10]	

* Repeat if > 2 weeks from Screening
1. Symptom-directed PEs to be performed by visit as necessary.
2. ECGs: Measured pre-dose and at 1 and 2 hours post-dose; more frequently per hour if necessary. Performed prior to other invasive procedures.
4. Vitals: Measured pre-dose and at 5 min, 0.5, 1, 2 hours post-dose on dosing days.
5. Urine pregnancy test for females of childbearing potential only.
6. AE/SAE data capture begins from time of informed consent.
7. Clinical Chemistry, Lipase, Hemoglobin A1C, Hematology, Coagulation and Urinalysis pre-dose only
8. Performed for females not of childbearing potential to confirm postmenopausal status
9. Pre-dose
10. Pregnancy Follow-Up phone call: 90 days post last dose ± 5 days. Visit not required for Cohort 7, 7b and 7c subjects electing to enroll in Cohort 9 at Day 113
11. **Cohort 8 only**: Complete GTT post-dose on Day 85 only ±3 days
12. Cohort 8 only: On Day 85 ±3 after an overnight 8 hour fast, serum triglycerides measured 2 hours after a high fat/high carbohydrate standardized meal. Post-dose GTT and post-prandial TG assessment may be completed on separate days. This test is NOT to be completed in patients with history of or at risk for post-prandial hypertriglyceridemia related abdominal pain or pancreatitis.
13. Cohort 8 only: Provide high fat/high carbohydrate standardized after 8 hour fast, 2 hours prior to post-prandial TG measurement on Day 85 ±3 days.
14. Pre-dose on Day 1; also taken on Day 57 and Day 113 or Early Termination
15. Lipid metabolic genetic analysis may be drawn on Day 1 or any time after Day 1 through EOS.
16. For Cohort 7/7b/7c patients NOT electing to continue receiving drug in Cohort 9 will proceed with standard EOS assessments. Patients electing to enroll in Cohort 9 are to complete Day 113 visit as per **Table 1e** after being consented for Cohort 9.

Table 1d: Cohorts 2b, 3b, 4b Two Q28 day doses in NHVs (applies to NZ only)

Assessment	Screen (Days -60 to -2)	Day -2 Confine	Day 1	Day 2 Discharge	Day 3	Day 8, 15, 22	Day 29 Confine	Day 30 Discharge	Day 31	Day 43, 57, 71, 85, 99	Day 119	Day 113 EOS	Early Term
Informed Consent	X												
Eligibility Criteria	X	X											
Body Mass Index	X											X	X
Demographics	X												
Medical History	X	X*											
Drug Screen	X	X											
Alcohol Breath Test	X	X											
Hepatitis/HIV Serology Screen	X												
Physical Exam[1]	X	X*		X	X	X	X	X	X	X		X	X
FSH	X[9]												
Pregnancy test (at Screening and pre-dose on dosing days)	X[6]		X[10]				X[10]					X	X
ECG	X		X[2]	X			X[2]	X					X
Vital Signs (BP, temp, RR, heart rate)	X		X[4]	X	X	X	X[4]	X	X	X		X	X
Clinical Labs (heme, coag, chem, serum glucose, lipase, serum insulin, hemoglobin A1C, C-peptide, UA)	X	X	X[8]	X	X	X	X[8]	X	X	X		X	X
GTT (may be completed Day -2 to Day -1 after 8 hour overnight fast)		X								X[13]			
Post-prandial TGs (may be completed Day -2 to Day -1 after 8 hour overnight fast)		X								X[14]			
Serum ANGPTL3, apoC-III, LDL-C, VLDL-C, HDL-C, Lp(a), Triglycerides, Total cholesterol, Non-HDL-cholesterol, ApoB-100, ApoB-48, ApoA-I (pre-dose on dosing days)	X		X		X	X	X			X		X	X
PK[3]			X	X	X		X	X	X				

Lipid metabolic genotype (if scientifically warranted) See Appendix 2			X[17]										
Urine collection for PK/excretion and metabolite ID[16]			X	X		X	X	X					
Concomitant Meds/Therapies	X	X	X	X	X	X	X	X	X	X		X	X
Meals[5]		X	X	X			X	X		X[15]			
Adverse Events[7]		X	X	X	X	X	X	X	X	X		X	X
Study Treatment			X				X						
Pregnancy F/U call											X[12]		

* Repeat if > 2 weeks from Screening
1. Symptom-directed PEs to be performed by visit as necessary.
2. ECGs: Measured pre-dose and at 1 and 2 hours post-dose; more frequently per hour if necessary. Performed prior to other invasive procedures.
3. PK: (plasma) Blood samples collected 0 min pre-dose, 15 min, 0.5, 1, 2, 3, 6, 9, 12, 18, 24 & 48 hours post-dose.
4. Vitals: Measured pre-dose and at 5 min, 0.5, 1, 2, 3, and 6 hours post-dose on Days 1 and 29.
5. Meals: Lunch, Dinner, and snack(s) on Day 1 aligned with fasting requirements stated in section 7.5.
6. Urine pregnancy test for females of childbearing potential only.
7. AE/SAE data capture begins from time of informed consent.
8. Clinical Chemistry, Hematology, Coagulation, Lipase, hemoglobin A1C and Urinalysis pre-dose only.
9. Performed for females not of childbearing-potential to confirm postmenopausal status.
10. Pre-dose on dosing days.
12. Pregnancy Follow-Up phone call: 90 days post last dose ± 5 days.
13. Complete GTT post-dose on Day 85 only ±3 days after an 8 hour overnight fast.
14. On Day 85 ±3 after an overnight 8 hour fast, serum triglycerides measured 2 hours after a high fat/high carbohydrate standardized meal. Post-dose GTT and post-prandial TG assessment must be completed on separate days within the Day 85 window.
15. Provide high fat/high carbohydrate standardized meal after 8 hour fast, 2 hours prior to post-prandial TG measurement on Day 85 ±3 days.
16. PK (Urine): Urine collected cumulatively from 0-6 hours, 6-24 hours post dose for both doses (Day 1 and 29). In addition, spot collection on Days 1 (predose), 8, 15, 22 and 29 (predose). Urine creatinine will be measured on all urine samples (interval and spot collections), Metabolite ID will be performed on urine samples collected (pooled analysis).
17. Lipid metabolic genetic analysis may be drawn on Day 1 or any time after Day 1 through EOS.

Table 1e: Cohort 9: Four Q12 week doses in FH patients who have completed all doses in Cohorts 7, 7b or 7c.

Assessment	Day 113 of Cohort 7, 7b or 7c	Day 141	Day 169	Day 197	Day 225	Day 253	Day 281	Day 309	Day 337	Day 365	Day 449 EOS	Early Term
Informed Consent	X											
Body Mass Index	X										X	X
Medical History	X											
Physical Exam[1]	X	X	X	X	X	X	X	X	X	X	X	X
Pregnancy test[2]	X			X			X			X	X	X
ECG	X[5]											
Vital Signs (BP, temp, RR, heart rate) [3]	X	X	X	X	X	X	X	X	X	X	X	X
Clinical Labs (heme, coag, chem, Hemoglobin A1C, serum glucose, C-peptide, serum insulin, lipase, UA[4]	X	X	X	X	X	X	X	X	X	X	X	X
Serum ANGPTL3, apoC-III, LDL-C, VLDL-C, HDL-C, Lp(a), Triglycerides, Total cholesterol, Non-HDL-cholesterol, ApoB-100, ApoB-48, ApoA-I, apoC-II, apoA-V (pre-dose on dosing days)[4]	X	X	X	X	X	X	X	X	X	X	X	X
Anti-drug antibodies[4]	X											
Concomitant Meds/Therapies	X	X	X	X	X	X	X	X	X	X	X	X
Adverse Events	X	X	X	X	X	X	X	X	X	X	X	X
Study Treatment	X			X			X			X		

1. Symptom-directed PEs to be performed by visit as necessary.
2. Pre-dose on dosing days in women of child-bearing potential.
3. Vitals: Measured pre-dose and at 5 min, 0.5, 1 hours post-dose on dosing days.
4. Pre-dose on dosing days
5. ECGs: Measured pre-dose and at 1 and 2 hours post-dose; more frequently per hour if necessary. Performed prior to other invasive procedures.

TABLE OF CONTENTS

1 PROTOCOL SYNOPSIS ... 2
2 STUDY INFORMATION AND SIGNATURES ... 24
3 LIST OF ABBREVIATIONS AND TERMS ... 25
4 INTRODUCTION ... 27
4.1 Background Information ... 27
4.2 Therapeutic Rationale and Mechanism of Action of ARO-ANG3 ... 27
4.3 ARO-ANG3 Pre-Clinical Pharmacology Studies ... 28
4.4 ARO-ANG3 Pre-Clinical Pharmacokinetic and Product Metabolism Studies ... 28
4.5 ARO-ANG3 Pre-Clinical Toxicology Studies ... 28
4.6 ARO-ANG3 Clinical Experience ... 29
4.7 Rationale for the Study ... 29
4.8 Risk Assessment for Participants ... 30
4.9 Justification for Starting Dose in Humans ... 31
5 OBJECTIVES ... 32
5.1 Primary Objectives ... 32
5.2 Secondary Objectives ... 32
5.3 Exploratory Objectives ... 33
6 STUDY PLAN ... 33
6.1 Study Design ... 33
6.2 Rationale for Study Design ... 37
Criteria for Dose-escalation and Stopping Rules ... 38
6.3 38
6.4 Duration of the Study ... 40
7 SUBJECT SELECTION ... 40
7.1 Number of Subjects ... 40
7.2 Inclusion Criteria ... 42
7.3 Exclusion Criteria ... 43
7.4 Participant Withdrawal Criteria ... 45
7.5 Restrictions and Concomitant Medications ... 46
8 INVESTIGATIONAL PRODUCT ... 47
8.1 Description, Identification and Dosage ... 47
8.2 Supply, Preparation, Storage and Labelling of ARO-ANG3 ... 47
8.3 Study Drug Handling ... 48
8.4 Accountability of Study Supplies ... 49
8.5 Retention of Investigational Product Vials ... 49
8.6 Allocation to Treatment ... 50
8.7 Blinding and Code-break ... 50
9 STUDY METHODS AND SCHEDULES ... 51

9.1 Overview of Procedures 51
9.2 Selection and Screening 52
9.3 On-Study Procedures/Assessments 53
9.3.1 Study Procedures: Clinical Facility Confinement 53
9.3.2 Demographics/Medical History 53
9.3.3 Physical Exam 54
9.3.4 Glucose Tolerance Test (GTT) & post-prandial Triglyceride Test 54
9.3.5 Magnetic Resonance Imaging 54
9.3.6 Electrocardiogram 54
9.3.7 Vital Sign Assessments 54
9.3.8 Clinical Laboratory Tests 55
9.3.9 Pharmacokinetics 56
9.3.10 Concomitant Medications/Therapies 56
9.3.11 Follow-Up Procedures: Pregnancy Follow-Up Telephone Call (90 days (± 5 days) post last dose) 56
9.3.12 Early Termination Procedures 57
9.4 Allocation of Formulations 57
9.5 Study Formulation Administration 57
9.6 Timing of Treatments and Procedures 58
9.7 Safety Measurements 59
9.8 Blood Sampling for Pharmacokinetic, Pharmacodynamic Analysis 60
10 ADVERSE EVENTS 61
10.1 Definitions 61
10.2 Clinical Laboratory Abnormalities and Other Abnormal Assessments as AEs 62
10.3 Timing, Frequency, and Method of Detecting AEs 62
10.4 Recording of AEs 63
10.5 Evaluating AEs 63
10.6 Follow-up of AEs 65
10.7 Prompt Reporting of SAEs 66
10.8 Regulatory Requirements for Reporting of SAEs 67
10.9 Post-study AEs 67
10.10 SAEs Related to Study Participation 67
11 DATA ANALYSIS AND STATISTICAL CONSIDERATIONS 67
11.1 Sample Size Considerations 67
11.2 Screening Data 67
11.3 Safety/Tolerability Data 67
11.4 Pharmacokinetic Data 68
11.5 Pharmacodynamic Data 69
11.6 Data Recording and Quality Control 69
12 STUDY APPROVAL AND CONDUCT 70
12.1 Regulatory Approval 70
12.2 Ethics Committee (EC) Approval 70
12.3 Ethical Considerations 70
12.4 Written Informed Consent 71

12.5 Emergency Contact with Principal Investigator ... 71
12.6 Notification of General Practitioner ... 71
12.7 Clinical Laboratory Certification and Reference Ranges ... 72
12.8 Protocol Deviations ... 72
12.9 Termination of the Study ... 73
13 STUDY ADMINISTRATION ... 73
13.1 Study Monitoring ... 73
13.2 Quality Assurance ... 74
13.3 Records Retention ... 74
14 INFORMATION DISCLOSURE AND INVENTIONS ... 76
14.1 Ownership ... 76
14.2 Confidentiality ... 76
14.3 Publication ... 76
15 REFERENCES ... 78

2 STUDY INFORMATION AND SIGNATURES

Investigator's Statement:

I have read and understood the information in this protocol and agree to conduct the trial according to the protocol (subject to any amendments) and in accordance with the principles of Good Clinical Practice. I have read and agree to comply with the Investigator obligations stated in this protocol. Any changes in procedure will only be made if necessary to protect the safety, rights or welfare of participants.

I agree to conduct in person or to supervise the trial.

I agree to ensure that all that assist me in the conduct of the study are aware of their obligations.

Principal Investigator:

________________________________ ________________________________
Signature Date

____
Printed Name

3 LIST OF ABBREVIATIONS AND TERMS

AE	Adverse Event
ANGPTL3	Angiopoietin like protein three
ALT	Alanine aminotransferase
API	Active Pharmaceutical Ingredient
apo(B)	Apolipoprotein B
apoA-I	Apolipoprotein A-I
apoA-V	Apolipoprotein A-V
apoC-II	Apolipoprotein C-II
apoC-III	Apolipoprotein C-III
AST	Aspartate transaminase
ARO	Arrowhead Pharmaceuticals, Inc
ARO-ANG3 Injection	Clinical drug product solution ready for SC injection
ARO-ANG3	Short name for ARO-ANG3 Injection
AUC	Area Under the Curve
AUC_{inf}	Area Under the Curve from time 0 to infinity
BMI	Body Mass Index
BP	Blood Pressure
cGCP	current Good Clinical Practice
cGMP	current Good Manufacturing Practice
C_{max}	Concentration maximum (peak)
CRA	Clinical Research Associate
CRF	Case Report Form
CRO	Contract Research Organization
CTN	Clinical Trial Notification
CVA	Cerebrovascular Accident
dL	deciliter
DSC	Data Safety Committee
EC	Ethics Committee
ECG	Electrocardiogram
eCRF	Electronic Case Report Form
EDTA	Ethylenediamine Tetra-acetic Acid
EOS	End of Study
FDA	Food and Drug Administration
FSH	Follicle-Stimulating Hormone
GGT	Gamma glutamyl transferase
GLP	Good Laboratory Practice
GTT	Glucose Tolerance Test
HBV	Hepatitis B virus
HCV	Hepatitis C virus
HREC	Human Research Ethics Committee
HDL-C	High density lipoprotein cholesterol
HIV	Human Immunodeficiency Virus
ICH	International Conference on Harmonisation
IRB	Institutional Review Board

ISR	Injection Site Reaction
IUD	Intrauterine Device
IWRS	Interactive Web Response System
kg	kilogram
LDH	Lactate Dehydrogenase
LDL-C	Low density lipoprotein cholesterol
Lp(a)	Lipoprotein(a)
LPL	Lipoprotein lipase
MAD	Multiple Ascending Dose
MCP-1	Monocyte chemoattractant protein-1
MCH	Mean Cell Hemoglobin
MCHC	Mean Cell Hemoglobin Concentration
MCV	Mean Cell Volume
MedDRA	Medical Dictionary for Regulatory Activities
mg	milligram
mmHg	millimeters of mercury
MRI	Magnetic Resonance Imaging
NHV	Normal Healthy Volunteer
non-HDL-C	Non-Low density lipoprotein cholesterol
OTC	Over the Counter
PD	Pharmacodynamic
PBO	Placebo
PI	Principal Investigator
PK	Pharmacokinetic
PT	Prothrombin Time or Preferred Term
PTT	Partial thromboplastin time
Q28	Once every 28-days
Q4W	Once every four weeks
QRS	QRS duration (complex) - a structure on the ECG that corresponds to the depolarization of the ventricles
QT	QT interval - a measure of the time between the start of the Q wave and the end of the T wave in the heart's electrical cycle
QTc	QT interval corrected for heart rate
RNA	Ribonucleic acid
RNAi	RNA interference
SAD	Single Ascending Dose
SAE	Serious Adverse Event
SD	Standard Deviation
siRNA	Short interfering RNA oligonucleotides
SOA	Schedule of Assessments
SOC	System Organ Class
$t_{1/2}$	terminal elimination half-life
TIA	Transient Ischemic Attack
TG	Triglyceride
ULN	Upper Limit of Normal

4 INTRODUCTION

4.1 *Background Information*

Angiopoietin like protein three (ANGPTL3) is a primarily hepatocyte synthesized member of the angiopoietin like family of proteins. Its key role is as a regulator of low-density lipoprotein cholesterol (LDL-C), high-density lipoprotein cholesterol (HDL-C) and Triglyceride (TG) metabolism. More specifically, ANGPTL3 inhibits lipoprotein lipase (LPL) which is responsible for triglyceride hydrolysis in peripheral tissues (e.g. adipose tissue, muscle). ANGPTL3 also inhibits endothelial lipase driven HDL-C metabolism and inhibits hepatocyte uptake of apoB containing lipoproteins (LDL-C and VLDL-C) through mechanisms at least partially independent of the LDL receptor. Given ANGPTL3's inhibitory role of various lipoproteins and triglycerides, reduced expression and reduced circulating levels of ANGPTL3 would be expected to increase clearance of LDL-C, HDL-C and TGs. As expected, individuals with ANGPTL3 loss-of-function mutations from birth present with very low levels of TGs, LDL-C and HDL-C (Minicocci et al. 2012; Musunuru et al. 2010; Romeo et al. 2009) . Patients with compound heterozygous or homozygous loss-of-function mutations can have undetectable serum ANGPTL3 with reductions in LDL-C of >65%, TG by >70% and reductions in HDL-C by approximately 40% when compared to controls (Minicocci et al. 2013). It has also been reported that individuals heterozygous for ANGPTL3 loss-of-function mutations demonstrate reduced LDL-C, HDL-C, TGs as well as reduction in the odds of developing atherosclerotic cardiovascular disease. To date no adverse clinical phenotype has been reported in ANGPTL3 deficient subjects (Dewey et al. 2017). The genetic validation consisting of low LDL-C and TGs coupled with reduced cardiovascular disease risk in ANGPTL3 deficient patients and proposed mechanism for these metabolic findings has promoted interest in methods capable of suppressing ANGPTL3. One method of targeting serum ANGPTL3 is with a monoclonal antibody approach. Evaluations with evinacumab, targeting circulating ANGPTL3 in healthy volunteers (Dewey et al. 2017) and in familial hypercholesterolemia (Gaudet et al. 2017) patients have shown potent reductions in LDL-C, HDL-C and TGs. However, an antibody approach would miss intra-hepatocyte ANGPTL3 which may be important for improvement of intra-hepatocyte triglyceride accumulation and insulin resistance (Graham et al. 2017).

4.2 *Therapeutic Rationale and Mechanism of Action of ARO-ANG3*

One method of inhibiting both intra-hepatic and circulating ANGPTL3 protein activity is through RNA interference (RNAi)-mediated gene silencing of ANGPTL3 protein production by hepatocytes. RNA interference (RNAi)-based therapeutics have the potential to silence the expression of any disease gene. RNAi is a naturally-occurring process by which short interfering RNA oligonucleotides (siRNAs)

trigger a sequence-specific down-modulation of gene expression. Hepatocyte synthesis is the dominant source of ANGPTL3 in humans. By delivering siRNAs targeting ANGPTL3 sequences to hepatocytes, it is possible to knock down expression of ANGPTL3 mRNAs in hepatocytes which reduces the synthesis of the ANGPTL3 protein, reducing both intra-hepatic and circulating ANGPTL3 levels. Reductions in expression of ANGPTL3 protein is expected to result in corresponding reductions in LDL-C, HDL-C, TGs and ApoB.

Arrowhead Pharmaceuticals, Inc. has developed a drug candidate, ARO-ANG3 to treat dyslipidemia through an RNAi-mediated mechanism. ARO-ANG3 is a novel hepatocyte targeted RNAi trigger molecule which is conjugated to N-acetyl-galactosamine to facilitate hepatocyte endocytosis through the asialoglycoprotein receptor. ARO-ANG3 is highly effective at knocking down the ANGPTL3 mRNA gene transcript and at reducing the production of hepatic ANGPTL3 protein with the expected corresponding reductions in LDL-C, TGs, and HDL-C as has been shown in animal studies.

4.3 *ARO-ANG3 Pre-Clinical Pharmacology Studies*

Preclinical pharmacology of ARO-ANG3 was evaluated in wild type mice, normal diet cynomolgus monkeys and in various animal models of dyslipidemia. In mice, treatment with ARO-ANG3 resulted in dramatically reduced serum ANGPTL3 protein levels, which correlated with reduced serum triglycerides and increased serum HDL. ARO-ANG3 treatment in lean chow-fed cynomolgus monkeys resulted in a maximum reduction in serum ANGPTL3 of approximately 90% in animals receiving two Q4W 3 mg/kg doses of ARO-ANG3. Further information on the pre-clinical pharmacology studies in cynomolgus monkeys, rhesus monkeys and in various mouse and monkey dyslipidemia models is provided in the Investigator's Brochure.

4.4 *ARO-ANG3 Pre-Clinical Pharmacokinetic and Product Metabolism Studies*

PK parameters for ARO-ANG3 have been evaluated in both rats and monkeys. Results of these studies can be found in the Investigator's Brochure.

4.5 *ARO-ANG3 Pre-Clinical Toxicology Studies*

ARO-ANG3 has been clinically well tolerated in rats and in non-human primate toxicology studies including as described in 6-month monkey and 6-month rat toxicology studies. Details regarding GLP and non-GLP toxicology results are provided in the Investigator's Brochure.

4.6 *ARO-ANG3 Clinical Experience*

Summary pharmacodynamic and safety data describing the experience to date with ARO-ANG3 is provided in the Investigator's Brochure.

4.7 *Rationale for the Study*

Treatment with ARO-ANG3 is expected to reduce hepatic production of ANGPTL3 via RNAi, leading to reductions in serum LDL-C, TG, VLDL-C and ApoB. The magnitude of the reduction and duration of effect will depend on the dose. Since to date there has been no human clinical exposure to ARO-ANG3, an effective therapeutic dose to administer to patients with dyslipidemia is unknown, although in the RNAi field, non-human primate potency and duration is usually similar to that seen in humans. This study uses a single-ascending-dose (SAD) in healthy volunteers to determine the dose required to reach and sustain maximal knockdown in ANGPTL3 serum levels, and the dose-response relationship in humans. In Cohort 5, ARO-ANG3 will be evaluated in healthy volunteer subjects with hepatic steatosis as inhibition of intra-hepatic ANGPTL3 may lead to reduced intra-hepatic fat. Cohort 6 will evaluate the ability of ANGPTL3 inhibition to further reduce LDL-C levels in subjects already on lipid lowering therapy such as HMG-CoA-reductase inhibitors ("statins"). Cohorts 7 and 8 are intended to evaluate preliminary evidence of safety and drug activity in patient populations likely to benefit from ARO-ANG3 treatment.

The rationale for addition of multiple dose NHV cohorts is to better understand multidose pharmacodynamic and pharmacokinetics at escalating doses.

The rationale for addition of Cohorts 7b and 7c is to better understand dose response in patients with familial hypercholesterolemia. The proposed doses of 100 and 300 mg have been evaluated previously in this study in healthy volunteers.

Cohort 9 is intended to allow HeFH patients enrolled in Cohorts 7, 7b, 7c continued access to ARO-ANG3 with the addition of four Q12 week doses. As shown in the Investigator's Brochure, in Cohort 7, 200 mg of ARO-ANG3 has demonstrated mean reductions in ANGPTL3 of 80% on Day 29 corresponding to mean nadir LDL-C reductions of 29% (mean absolute reductions of 47.7 md/dL) in patients with HeFH. This finding is highly clinically significant as Cohort 7 patients entered the study with mean LDL-C of 154 mg/dL (Range 100-181) despite the baseline use of statins and/or ezetimibe and in some cases, PCSK-9 inhibitors. This additional reduction in LDL-C with the addition of ARO-ANG3 on top of maximal standard therapy could help these patients reach and maintain target LDL-C

goals. Lower LDL-C is associated with reduced risk of atherosclerotic cardiovascular disease in the HeFH/HoFH population. Also as described in the Investigator's Brochure, the safety profile of ARO-ANG3 is supportive of continued dose administration at the dose levels used in this clinical study as is the 6-month rodent and 6-month monkey GLP toxicology data.

4.8 *Risk Assessment for Participants*

- Limited GLP toxicology studies have been conducted. Accordingly, eligible participants enrolled in this study, both male and female (including partners), must agree to use two highly effective forms of contraception during the study and for 3 months post-dose, or agree to abstinence (acceptable only if this method is in alignment with the normal life style of the patient).

- ARO-ANG3 targets the liver. siRNA literature has described ALT changes associated with off-target effects of the siRNA seed region on microRNAs in the hepatocyte (Janas et al. 2018). The siRNA sequence of the ARO-ANG3 sense and antisense molecules have been screened for potential mRNA and microRNA homology and sequences with homology were excluded from consideration. Thus, no such off-target effects are anticipated. Minimal changes in ALT and AST have been seen with high doses of ARO-ANG3 in rat GLP toxicity studies at high doses (See Investigator Brochure). However, no such changes were seen in the monkey GLP toxicity studies. To mitigate this risk, the proposed study protocol has built in stopping rules for ALT and AST elevation. Blood samples will be drawn frequently to evaluate liver injury and liver function. The Data Safety Committee (DSC) will review all available safety data including laboratory data prior to dose escalation. Additionally, the planned starting dose of 35 mg is approximately 1/600th (assuming weight-based conversion and a 70kg subject) of the No-Observed-Adverse-Effect-Level (NOAEL) of 300 mg/kg in monkey GLP toxicity studies and 1/30th of the NOAEL of 15 mg/kg observed in rat GLP toxicity studies.

- Other subcutaneously administered modified siRNA drug candidates evaluated in clinical studies have been associated with mild to moderate injection site reactions (e.g. pain, erythema). This study includes a protocol for evaluation and grading of injection site reactions based on predefined criteria for mild, moderate and severe. Injection site reactions will be photographed for tracking resolution and/or progression. Additionally, steps will be taken to minimize injection site reactions such as rotating injection sites and allowing the ARO-ANG3 solution to come to room temperature prior to injecting.

4.9 *Justification for Starting Dose in Humans*

Regulatory guidance for calculation of starting dose in healthy volunteer studies indicates that the No-Observed-Adverse-Effect-Level (NOAEL) in the most relevant animal species should be used (Guideline on strategies to identify and mitigate risks for first-in-human and early clinical trials with investigational medicinal products, European Medicines Agency 20 July 2017). FDA guidance also recommends calculation of human starting dose based on the NOAEL in the most relevant animal model (Guidance for Industry Estimating the Maximum Safe Starting Dose in Initial Clinical Trials for Therapeutics in Adult Healthy Volunteers, July 2005). The siRNA sequence used in ARO-ANG3 shares cross-reactivity with mice, rats and monkeys and effectively silences liver ANGPTL3 production in these species. The most pharmacologically relevant animal model for calculating the NOAEL is the monkey. Consensus statements regarding appropriate pharmacologically relevant animal models in toxicology studies of oligonucleotide therapeutics also support use of non-human primates (Marlowe et al. 2017).

Reported pharmacokinetic properties for several oligonucleotide subclasses across species, including humans (Yu et al. 2001; Geary, Yu, and Levin 2001), indicate that the most appropriate method for extrapolating animal doses to human equivalent doses is the comparison of dose per unit body weight (mg/kg), rather than dose per surface area (mg/m2) or plasma exposure (AUC or C_{max}). The human equivalent dose for oligonucleotide therapeutics can be extrapolated directly from monkeys to humans with a scaling factor of 1.0 on mg/kg dose administrations (Yu et al. 2015). Arrowhead has historically used 1:1 mg/kg scaling factor based on monkey NOAELs to determine starting dose in other siRNA first-in-human studies (ARO-HBV and ARO-AAT first in human studies).

The proposed starting dose of 35 mg will be administered to healthy volunteers in Cohort 1 and represents 1/600th and 1/30th respectively (extrapolated based on weight) of the monkey and rat NOAEL of 300 mg/kg and 15 mg/kg respectively based on GLP toxicity studies. The maximum proposed dose in the study of 300 mg is approximately 1/70th of the monkey NOAEL.

The proposed dose to be used in Cohorts 5, 6, 7 and 8 is 200 mg. This dose is justified for the following reasons:

1. There have been no reported drug related SAEs or severe AEs at any dose.
2. There have been no dose limiting toxicities or patterns of increased frequency or intensity of AEs with increasing dose. From a safety standpoint, the 35, 100, 200 and 300 mg dose levels are indistinguishable.

3. There is a dose response in reductions in serum ANGPTL3 and serum triglycerides up through the 200 mg dose level. 200 and 300 mg pharmacodynamic results are very similar indicating that 200 mg is the approximate top of the dose-response curve. No meaningful additional reduction in serum ANGPTL3 or triglycerides is achieved with dose escalation to 300 mg.

In summary, the lack of a safety difference amongst dose levels and the best pharmacodynamic responses emerging at the 200 mg dose level, without significant improvement in ANPTL3 or triglyceride reduction at 300 mg, supports 200 mg as having the best risk benefit profile for the patient cohorts (cohorts 5, 6, 7 and 8) in this ongoing study. Patients with familial hypercholesterolemia have monogenic or polygenic causes of impaired cholesterol metabolism. These patients may respond differently to siRNA-mediated ANGPTL3 inhibition. For this reason, Cohorts 7b and 7c are being added to explore dose response in FH patients.

The planned dose level for Cohort 9 is 200 mg. In Cohort 7 (open label enrolling HeFH and HoFH patients), 200 mg has demonstrated mean reductions in ANGPTL3 of 80% and mean nadir reductions in LDL-C of 29% on post-dose Day 29. There has not been evidence of a difference in safety profile in Cohort 7 as compared to NHV cohorts. The 200 mg dose level appears effective with encouraging available safety profile to date in patients with familial hypercholesterolemia. Additionally, in multi-dose NHV cohorts 2b, 3b, 4b, dose escalation from 200 mg to 300 mg did not confer significant additional reductions in ANGPTL3 or LDL-C based on available data.

5 OBJECTIVES

5.1 *Primary Objectives*

The Primary Objective of this study is to determine the incidence and frequency of adverse events possibly or probably related to treatment as a measure of the safety and tolerability of ARO-ANG3 using escalating single and multiple doses in healthy volunteers and multiple doses in dyslipidemic patients.

5.2 *Secondary Objectives*

The Secondary Objectives are:

- To evaluate the single-dose and multi-dose pharmacokinetics of ARO-ANG3 in healthy volunteers.

- To determine the reduction in fasting serum ANGPTL3 from baseline in response to a single dose and multiple doses of ARO-ANG3 as a measure of drug activity in healthy volunteers and in response to multiple doses of ARO-ANG3 in dyslipidemic patients (all values drawn after at least 8 hour fast).

5.3 *Exploratory Objectives*

- To evaluate the effect of ARO-ANG3 on change from baseline in fasting LDL-C, Total Cholesterol, non-HDL-C, HDL-C, VLDL-C, Triglycerides, Lp(a), apoB-48, apoB-100, apoC-III, apoC-II, apoA-V, lipoprotein lipase mass (if feasible), hepatic lipase mass (if feasible), CETP mass (if feasible) and apoA-I (all values drawn after at least 8 hour fast).
- To evaluate the effect of doses of ARO-ANG3 on changes from baseline in BMI.
- To evaluate the effect of ARO-ANG3 on changes from baseline in fasting serum blood glucose, hemoglobin A1C, C-peptide, GTT and fasting serum insulin.
- To evaluate the effect of ARO-ANG3 on change from baseline liver fat content using Magnetic Resonance Imaging (MRI-PDFF) in Cohort 5 only.
- To evaluate the effect of ARO-ANG3 on change from baseline in post-prandial (post standardized high fat/high carbohydrate meal) serum TGs in specified cohorts.
- To evaluate excretion of ARO-ANG3 (full length and metabolites) and identify metabolites in plasma and urine in the multi-dose healthy volunteer cohorts.

6 STUDY PLAN

6.1 *Study Design*

Participants who have signed an EC approved informed consent form and have met all the protocol eligibility criteria during screening may be enrolled into the study in a double-blind or open label fashion depending on the cohort. Cohorts 1 through 4 will begin with administration of ARO-ANG3 or PBO to two sentinel participants (one ARO-ANG3, one PBO). Following the Day 3 evaluation in these participants, if there are no significant safety concerns, the remaining participants in the cohort will be treated at the discretion of the Principal Investigator (PI). Dosing of participants will be staggered by at least 30 minutes such that no two participants will be dosed simultaneously.

Dose levels by cohort are outlined in Figures 1 and 2. Cohorts 1 through 4 will enroll sequentially. Cohorts 5, 6, 7 and 8 may be opened after review of cumulative safety data from all previous cohorts

including through Day 8 of Cohort 4. After review of such cumulative safety data by the DSC, an amended protocol justifying the dose for Cohorts 5, 6, 7 and 8 will be submitted to the EC for approval (See Section 4.8). Screening and enrollment in Cohorts 5-8 will not occur until this amended protocol is approved by the EC. These multi-dose patient cohorts may enroll in parallel after they are opened for enrollment by the Data Safety Committee (DSC) and after EC approval of the amended protocol.

In the dose escalation part of the study (Cohorts 1 through 4), dose escalation will require approval by the DSC based on all cumulative available safety data for prior cohorts, and through at least Day 8 of the current NHV cohort (i.e. cohorts 1 through 4). Based on available safety data through Day 8, the DSC will vote to approve opening for enrollment of the next planned cohort/dose level. DSC decisions will be based on all aggregate safety data available including all data available at least through Day 8 of the current cohort as shown in Figure 2. Escalation to the next highest dose level will proceed until the dose level of 300 mg is completed, or the trial is halted prematurely by the PI, DSC, or Sponsor due to safety or other reasons. All subjects who withdraw from the study prior to their End of Study visit, for reasons other than an adverse event, may be replaced.

Cohort 9 is only open to patients who have completed Cohorts 7, 7b and 7c. At End of Study for Cohorts 7, 7b and 7c, patients may elect to proceed with the End of Study visit or continue to receive up to four additional doses of ARO-ANG3 administered every 12 weeks in Cohort 9.

Clinical facility confinement will be approximately 3 days for first dose administration (Day -2 through 24-hour assessments) for Cohorts 1 through 4 with discharge on Day 2 and 2 hours on the dosing day for Cohorts 5 through 9. Cohorts 2b, 3b and 4b will be confined approximately 1.5 days at Day 29 (Day 29 through 24 hour PK sample collection with an outpatient PK visit on Day 31.) Blood samples will be drawn pre-dose on Day 1 for baseline measurements. Height and weight will be measured at Screening to calculate BMI and as otherwise specified in the Schedule of Assessments.

In double-blind cohorts, blinding will be preserved to the extent possible (or unless otherwise specified); however, treatment un-blinding will occur, at the PI’s discretion, where deemed necessary for treatment of an AE or for a decision to be made regarding trial continuation. After all subjects in a cohort have completed the final planned study visit on Day 113 (not including the 90-day follow-up call), Sponsor may be unblinded at Sponsor’s request but PI and study participants will remain blinded. For clarity, Cohort 5 may be unblinded to Sponsor on Day 113, prior to Day 168 MRI.

Sponsor may request an interim descriptive analysis of the change from baseline in ANGPTL3, apoC-III and other measured lipid parameters any time after all subjects planned for enrollment in each cohort have received at least one dose of ARO-ANG3 or PBO. This interim analysis is for the planning of future studies and will not impact the conduct of this study. Sponsor will remain blinded to all subject treatment assignments. Descriptive statistics (that do not inadvertently unblind the trial) for change from baseline in pharmacodynamic measures will be calculated for all active subjects per cohort and for a pooled PBO group by an unblinded statistician and provided to Sponsor. For any AEs occurring more than once, the frequency of AEs for a specific preferred term will be calculated for pooled active and pooled PBO groups in such a way not to inadvertently break the subject-blind of the trial.

Single and multiple doses of ARO-ANG3 will be evaluated in a sequential manner as shown in Figure 2.

Figure 1: Cohort Summary

Cohort	Population	Blinding	# Subjects	Dosing Schedule
1	NHVs TGs > 100 mg/dL (1.13 mmol/L), LDL-C > 70 mg/dL (1.81 mmol/L)	Double-blind	10 (6 active: 4 PBO)	35 mg on Day 1 only
2	NHVs TGs > 100 mg/dL (1.13 mmol/L), LDL-C > 70 mg/dL (1.81 mmol/L)	Double-blind	10 (6 active: 4 PBO)	100 mg Day 1 only
2b	NHVs	Open-label	4 active	100 mg Day 1, 29
3	NHVs TGs > 100 mg/dL (1.13 mmol/L), LDL-C > 70 mg/dL (1.81 mmol/L)	Double-blind	10 (6 active: 4 PBO)	200 mg Day 1 only
3b	NHVs	Open-label	4 active	200 mg Day 1, 29
4	NHVs TGs > 100 mg/dL (1.13 mmol/L), LDL-C > 70 mg/dL (1.81 mmol/L)	Double-blind	10 (6 active: 4 PBO)	300 mg Day 1 only
4b	NHVs	Open-label	4 active	300 mg Day 1, 29
5	NHVs with liver fat based on MRI-PDFF ≥ 10%	Double-Blind	9 (6 active: 3 PBO)	200 mg Day 1, 29
6	LDL-C > 70 mg/dL (1.81 mmol/L) on stable statin regimen	Double-Blind	9 (6 active: 3 PBO)	200 mg Day 1, 29
7	Familial Hypercholesterolemia	Open-label	≤ 6 active	200 mg Day 1, 29
7b	Familial Hypercholesterolemia	Open-label	≤ 6 active	100 mg Day 1, 29
7c	Familial Hypercholesterolemia	Open-label	≤ 6 active	300 mg Day 1, 29
8	TGs ≥ 300 mg/dL (3.39 mmol/L)	Open-label	≤ 6 active	200 mg Day 1, 29
9	Familial Hypercholesterolemia Extension Cohort (have completed Cohort 7, 7b or 7c)	Open-label	≤ 18 active	200 mg Days 113, 197, 281, 365

Figure 2: Dose Escalation Schedule

**Single Dose Healthy Volunteers (double blind in Cohorts 1, 2, 3, 4)**			**Multi-dose Patients (Double-Blind in Cohorts 5, 6, Open-label in 2b, 3b, 4b, 7, 7b, 7c, 8, 9)**
Cohort*	Dose (Day 1)	Day 8 safety evaluation	Dose Regimen
Cohort 1**	35 mg		NA
Cohort 2**	100 mg		NA
Cohort 3**	200 mg		NA
Cohort 4**	300 mg		NA
			Cohort 5***: 200 mg or PBO dosed on Day 1, 29
			Cohort 6***:200 mg or PBO doses on Day 1, 29
			Cohort 8***:200 mg doses on Day 1, 29
			Cohort 2b†: 100 mg dosed on Day 1, 29
			Cohort 3b†: 200 mg dosed on Day 1, 29
			Cohort 4b†: 300 mg dosed on Day 1, 29
			Cohort 7***: 200 mg dosed on Day 1, 29
			Cohort 7b†: 100 mg dosed on Day 1, 29
			Cohort 7c†: 300 mg dosed on Day 1, 29
			Cohort 9 ‡: 200 mg dosed on Days 113, 197, 281, 365

*Cohorts 1, 2, 3, and 4 will use sentinel subjects.

** Dose escalation to the next highest dose level or to multiple dosing will occur after cumulative safety data through Day 8 for Cohorts 1, 2, 3, and 4 have been evaluated by the DSC

*** Screening or enrolment into Cohorts 5, 6, 7 and 8 may not occur until an amended protocol justifying the dose to be used in these cohorts has been approved by the EC.

† No DSC vote is required to open cohorts 2b, 3b and 4b. These cohorts may enroll in parallel.

† No DSC vote is required to open cohorts 7b, 7c. These cohorts may enroll in parallel.

‡ Patients from Cohorts 7, 7b and 7c may elect to continue to receive up to four 200 mg doses administered approximately every 12 weeks. No DSC vote is required for patient to roll over into Cohort 9.

6.2 *Rationale for Study Design*

This first-in-human study plans to investigate ARO-ANG3 in adult healthy volunteers to evaluate the drug's safety and tolerability as well as its pharmacokinetics and pharmacodynamics following single or multiple subcutaneous doses. The study initiates in healthy volunteers because the risk is considered low. The study will utilize healthy volunteers with elevated LDL-C and TGs to better identify pharmacologic effect in subjects with elevated baseline values. As it is expected that patients being treated for dyslipidemia with ARO-ANG3 will require multiple doses, this study in healthy volunteers, transitions from single dose to a multi-dose study if safety data is acceptable to an independent DSC.

Cohorts 1 through 4 in the study are double-blind to limit the occurrence of conscious and unconscious bias in trial conduct and interpretation. Blinding will be achieved using a PBO product (0.9% normal saline). Inclusion of participants receiving PBO will reduce bias in the assessment of drug safety and tolerability. Cohorts 1, 2, 3, and 4 are randomized with a 6:4 (active:PBO) ratio to reduce bias.

Cohorts 5 and 6 are also double-blind and will be utilized to assess pharmacologic effect in subjects with liver steatosis (Cohort 5) and in subjects also on a stable regimen of cholesterol lowering medications which must include a "statin" (Cohort 6).

Cohorts 7, 7b, 7c and 8 are open-label and will assess pharmacologic effect and dose response in patients diagnosed with heterozygous or homozygous familial hypercholesterolemia, defined as documented positive genetic test OR Dutch Lipid Clinic Network Score ≥ 8 with LDL-C > 100 despite standard of care therapy OR with LDL-C > 70 mg/dL (1.81 mmol/L) while on a PCSK-9 inhibitor OR with LDL-C > 70 mg/dL (1.81 mmol/L) in the presence of documented atherosclerotic cardiovascular disease (Cohort 7, 7b, 7c) and those with fasting serum triglycerides of at least 300 mg/dL or 3.39 mmol/L (Cohort 8).

The rationale for addition of multiple dose NHV cohorts is to better understand multidose pharmacodynamic and pharmacokinetics at escalating doses.

6.3 *Cohort 9 is intended to allow HeFH patients enrolled in Cohorts 7, 7b, 7c continued access to ARO-ANG3 with the addition of four Q12 week doses to maintain reductions seen with ARO-ANG3 treatment. Criteria for Dose-escalation and Stopping Rules*

Dose escalation will require approval by the DSC based on all cumulative available safety data through Day 8 of the current cohort. Cohorts 2b-4b and 5-8 will be dosed on Day 1 and Day 29. DSC decisions will be based on all aggregate safety data available including all data available at least through Day 8 of the current cohort as shown in **Figure 1.** Dose escalation will proceed until all cohorts are fully enrolled or until the study is stopped or the DSC votes to not escalate to the next dose.

If a serious adverse event (SAE) at least possibly related to study drug should occur for a single participant, subsequent dosing within that cohort may be put on hold pending a complete review of safety data by the DSC to determine if participant enrollment at the same dose may proceed, or if additional enrollment/dosing should stop. If following the DSC safety review, it is deemed appropriate to restart dosing/enrollment, subject enrollment, dosing and dose escalation may proceed as planned.

Escalation to the next cohort will proceed according to the study design until the 300 mg dose level is completed, unless the trial is stopped early by the DSC or Sponsor. The dose for Cohorts 5 through 8 will be determined based on available safety data at the Cohort 4 DSC meeting. Justification for this dose level for Cohorts 5, 6, 7 and 8 will be provided to the EC via amended protocol which must receive approval prior to commencement of screening and enrollment into Cohorts 5, 6, 7 and 8 (See Section 4.8 for Cohort 5, 6, 7 and 8 dose justification). A decision to stop the trial early or discontinue drug in an individual subject or group of subjects **may** be indicated based on any of the following:

- Two or more similar Serious Adverse Event (SAE, defined in Section 9.1) considered at least possibly related to ARO-ANG3.
- One of the following abnormal results at least possibly related to ARO-ANG3:
    - o Treatment emergent AST and/or ALT > 8X ULN which must be confirmed by repeat blood draw within 48 hours of initial results OR a treatment emergent AST or ALT >3X ULN with a total bilirubin >2X ULN which must be confirmed by repeat blood draw within 48 hours of initial results OR a treatment emergent AST or ALT >3X ULN with

an INR > 1.5 (both of which must be confirmed on repeat) OR AST or ALT >3X ULN (which must be confirmed on repeat) with symptoms (e.g. nausea & vomiting, RUQ pain, fever, rash) or with eosinophilia. For Cohorts 5, 6, 7 and 8, patients with NAFLD and/or dyslipidemia or on statins may have elevated ALT and/or ASL at baseline. For this reason, liver related stopping rules will follow those proposed by Chalasani et al., 2016 which are described in Appendix 1.

- Two or more occurrences of treatment emergent platelet count < 70,000 per microliter which must be confirmed by repeat blood draw within 48 hours of initial results.

- Two or more occurrences of treatment emergent serum creatinine increase of > 0.3 mg/dL (26. 5 µmol/L) AND >50% increase from pre-dose baseline both of which must occur in the first 8 days of dosing and which must be confirmed by repeat blood draw within 48 hours of initial results.

Sponsor or PI can discontinue any subject at any time with or without DSC consultation. If such events (as described in #1, #2 above) occur and the subject is not discontinued from the study, the reason for not discontinuing the subject will be included in DSC meeting minutes. Including, but not limited to the events listed above, the DSC may pause the study to additional dosing or dose escalation to provide time to evaluate safety data and recommend the action to be taken, which may include, but is not limited to, one of the following:

- Discontinuation of a subject or group of subjects from the study
- The study is stopped immediately with no further dosing
- The study will continue until the current cohort is completed
- The study will continue, but the next dose escalation will be to a level midway between the current level and the next level specified in Section 6.3
- The study will continue as planned

6.4 *Duration of the Study*

For each subject in the study, the duration of the study clinic visits is approximately 25 weeks from screening to the Day 113 End-of-Study examination (not including 90-day follow-up phone call). The full duration of the study for Cohort 9 participants is approximately 73 weeks.

7 SUBJECT SELECTION

7.1 *Number of Subjects*

A total of up to 94 subjects may be enrolled in the study (not including replacements). Each potential participant must meet the inclusion and exclusion criteria to qualify for admission onto the study.

This study will be conducted in adult males and females, aged 18-65 years (up to age 70 for Cohorts 7, 7b, 7c,8 and 9 if otherwise healthy and at the discretion of the investigator) with BMI between 19.0 and 40.0 kg/m² and:

- Cohorts 1, 2, 3 and 4: All subjects will have fasting Screening triglycerides > 100 mg/dL (1.13 mmol/L) and fasting Screening LDL-C > 70 mg/dL (1.81 mmol/L) and not on any lipid lowering therapy. Each double-blind cohort will enroll ten (10) subjects (6 active: 4 PBO) with all cohorts planned to receive single escalating doses of ARO-ANG3 or PBO at escalating dose levels as per Figure 1 and 2 of 35, 100, 200, and 300 mg.

- Cohort 5: Cohort is double-blind with up to 9 subjects (6 active: 3 PBO, all receiving multiple doses of ARO-ANG3 or PBO. All subjects will have a liver fat fraction of ≥10% based on MRI-PDFF conducted at Screening.

- Cohort 6: Cohort is double-blind with up to 6 active and 3 PBO subjects, all receiving multiple doses of ARO-ANG3 or PBO. All Cohort 6 subjects will be on a stable drug treatment regimen for elevated LDL-C including a statin for at least 6 months fasting Screening LDL-C > 70 mg/dL (1.81 mmol/L).

- Cohort 7, 7b, 7c: Cohorts are open-label with up to 6 patients each with a diagnosis of heterozygous or homozygous familial hypercholesterolemia, defined as documented positive genetic test OR Dutch Lipid Clinic Network Score ≥ 8 with LDL-C > 100 despite standard of care therapy OR with LDL-C > 70 mg/dL (1.81 mmol/L) while on a PCSK-9 inhibitor OR with LDL-C > 70 mg/dL (1.81 mmol/L) in the presence of documented atherosclerotic cardiovascular disease. All subjects to receive multiple doses of ARO-ANG3.

- Cohort 8: Cohort is open-label with up to 6 patients with fasting serum triglycerides of at least 300 mg/dL (3.39 mmol/L). All subjects to receive multiple doses of ARO-ANG3.

- Cohorts 2b, 3b, 4b: Cohorts are open-label with 4 NHVs. Each open-label cohort will enroll four (4) subjects with all cohorts planned to receive multiple escalating doses of ARO-ANG3 at escalating dose levels as per Figure 1 and 2 of 100, 200, and 300 mg. Cohorts 2b-4b will be enrolled in New Zealand only.

- Cohort 9: Open label extension cohort for patients who have completed Cohorts 7, 7b and 7c. Up to 18 HeFH and HoFH patients electing to proceed in Cohort 9 will receive up to four additional Q12 week 200 mg doses of ARO-ANG3.

Participants who are withdrawn or discontinue prior to EOS for reasons other than an adverse event, may be replaced at Sponsor’s discretion.

Cohorts 1 through 4 will enroll sequentially. Cohorts 5, 6, 7 and 8 may be opened by the DSC after review of cumulative safety data through Day 8 of Cohort 4 and only after approval by the HREC of an amended protocol updating safety information from Cohorts 1 through 4 which will include a rationale for the dose level to be used in Cohorts 5, 6, 7and 8 (See Section 4.8). These multi-dose patient cohorts may enroll in parallel after they are opened for enrollment by the Data Safety Committee (DSC) and after the amended protocol has been approved by the HREC. Screening for Cohorts 5, 6, 7,and 8 may begin after the described amended protocol has been approved by the EC. At End of Study for Cohorts 7, 7b and 7c, patients may elect to proceed with the End of Study visit or continue to receive up to four additional quarterly doses of ARO-ANG3 in Cohort 9.

Cohorts 1, 2, 3, & 4 will be randomized to receive ARO-ANG3 or PBO (6 active:4 PBO) at single or multiple escalating doses of 35, 100, 200, and 300 mg administered as a subcutaneous injection. Cohorts 5 and 6 will be randomized to receive ARO-ANG3 or PBO (6 active:3 PBO) at multiple doses of 200 mg. Cohorts 7, 7b, 7c and 8 will be open-label and will receive multiple subcutaneous doses of ARO-ANG3 at dose levels of 100 mg (Cohort 7b), 200 mg (Cohorts 7 and 8) and 300 mg (Cohort 7c). Patients from Cohorts 7, 7b and 7c may elect to continue to receive up to four 200 mg doses administered approximately every 12 weeks. Cohorts 2b, 3b, 4b will be open-label and will receive multiple subcutaneous doses of ARO-ANG3 at a dose level 100 mg, 200 mg and 300 mg respectively. Additional intermediate dose cohorts may be added if approved by Sponsor, EC and the DSC.

7.2 *Inclusion Criteria*

To be eligible for enrollment, participants must meet all the following inclusion criteria:

1. Male or female volunteers 18-65 years of age. In cohorts 7, 7b, 7c,8 and 9 subjects up to age 70 may be eligible if otherwise healthy and at the discretion of the investigator.

2. Able and willing to provide written informed consent prior to the performance of any study specific procedures

3. Participants with a BMI between 19.0 and 40.0 kg/m^2, inclusive and on a stable diet for at least 4 weeks with no plans to significantly alter diet or BMI over course of study

4. A 12-lead ECG at Screening and pre-dose assessment that, in the opinion of the PI, has no abnormalities that compromise participant's safety in this study

5. Non-nursing females

6. Fasting serum triglycerides > 100 mg/dL (1.13 mmol/L) at Screening (applicable to Cohorts 1, 2, 3 and 4 only. Does not apply to Cohorts 2b, 3b, 4b.)

7. Fasting serum LDL-C > 70 mg/dL (1.81 mmol/L) at Screening (applicable to Cohorts 1, 2, 3 and 4 only. Does not apply to Cohorts 2b, 3b, 4b.)
8. Participants using two highly effective forms of contraception (both male and female partners) during the study and for 3 months following the dose of ARO-ANG3. Males must not donate sperm for at least 3 months post-dose of the last study treatment. Male partners of female participants and female partners of male participants must also use contraception, if they are of childbearing potential. Females of childbearing potential must have a negative urine pregnancy test at Screening and on Day 1. Females not of childbearing potential must be post-menopausal (defined as cessation of regular menstrual periods for at least 12 months), confirmed by follicle-stimulating hormone (FSH) level in the post-menopausal reference range.

   - Using twice the normal protection of birth control by using a condom AND one other form of the following:

     - Birth control pills (The Pill)

     - Depot or injectable birth control

     - IUD (Intrauterine Device)

     - Birth Control Patch (e.g., Othro Evra)

     - NuvaRing®

   Surgical sterilization (i.e. tubal ligation or hysterectomy for women or vasectomy for men or other forms of surgical sterilization) which can be verified in the subject's medical history is acceptable as a single form of contraception.

Rhythm methods will not be considered as highly effective methods of birth control. Subject abstinence for the duration of the study and three months after the dose of ARO-ANG3 is acceptable only when this method is in alignment with the normal life style of the patient.

9. Participants who are willing and able to comply with all study assessments and adhere to the protocol schedule

10. Must have suitable venous access for blood sampling

11. AST and ALT < 1.5X ULN at Screening for Cohorts 1 through 4 and 2b through 4b (one repeat screen test allowed)

12. AST and ALT < 3X ULN at Screening for Cohorts 5, 6, 7, 7b, 7c, 8 (one repeat screen test allowed)

13. Creatinine levels ≤ upper limit of normal at Screening (one repeat screen test allowed)

14. MRI-PDFF indicating a liver fat content of ≥10% (Cohort 5 only)

15. On a stable regimen of statin therapy for at least 6 months and LDL-C > 70 mg/dL (1.81 mmol/L) at Screening (Cohort 6 only)

16. Documented genetic diagnosis consistent with familial hypercholesterolemia (homozygous or heterozygous) with genotype documented in a verifiable source document OR Dutch Lipid Clinic Network Score ≥ 8 (Cohort 7, 7b, 7c only)

17. LDL-C > 100 mg/dL (2.59 mmol/L) despite standard of care therapy OR LDL-C > 70 mg/dL (1.81 mmol/L) while on a PCSK-9 inhibitor OR LDL-C > 70 mg/dL (1.81 mmol/L) in the presence of documented atherosclerotic cardiovascular disease (Cohort 7, 7b, 7c only)

18. Screening fasting triglycerides ≥ 300 mg/dL (3.39 mmol/L) (Cohort 8 only). Up to two repeated fasting triglyceride tests during Screening are acceptable.

19. Cohort 9 only: must have completed all doses in Cohort 7, 7b or 7c.

7.3 *Exclusion Criteria*

A potential subject will be excluded from the study if *any* of the following criteria apply:

1. Female subjects with a positive pregnancy test or are lactating

2. Acute signs of hepatitis (e.g., moderate fever, jaundice, nausea, vomiting, abdominal pain) at Screening or at baseline

3. Use of prescription medication that in the opinion of the study Investigator or the Sponsor would interfere with study conduct. Stable regimens to lower LDL-C or TGs or to treat cardiovascular disease, stable regimens of anti-hypertensives and stable regimens of antiplatelet agents or anti-coagulants are acceptable for cohorts 5, 6, 7 and 8 as long as subject meets other criteria. Stable regimen is defined as on treatment for at least 3 months. Topical products without systemic absorption, OTC and prescription pain medication or hormonal contraceptives (females) are acceptable at the Investigator's discretion

4. Use of more than two tobacco/nicotine containing or cannabis products (e.g. two cigarettes) per month within 6 months prior to the first study drug administration (Applicable only to healthy volunteer cohorts 1, 2, 3, 4, 2b, 3b, 4b).

5. Human immunodeficiency virus infection, as shown by the presence of anti-HIV antibody (sero-positive)

6. Seropositive for HBV or HCV (HCV seropositivity requires positive test for antibodies confirmed with positive test for HCV RNA)

7. Has uncontrolled hypertension defined as blood pressure > 170/100 mmHg at screening confirmed by repeat

8. A history of torsades de pointes, ventricular rhythm disturbances (e.g., ventricular tachycardia or fibrillation), pathologic symptomatic bradycardia, 2nd degree or 3rd degree heart block, congenital long QT syndrome, prolonged QT interval due to medications, or new elevation or depression in the part of an ECG immediately following the QRS complex and merging into the T wave (ST segment) or new pathologic inverted T waves, or new pathologic Q waves on ECG that are deemed clinically significant in the opinion of the PI. Subjects with a history of atrial arrhythmias should be discussed with the Sponsor Medical Monitor and CRO Medical Monitor.

9. A family history of congenital long QT syndrome, Brugada syndrome or unexplained sudden cardiac death

10. Symptomatic heart failure (per NYHA guidelines), unstable angina, myocardial infarction, severe cardiovascular disease (ejection fraction < 20%, transient ischemic attack (TIA) or cerebrovascular accident (CVA) within 6 months prior to study entry. For Cohorts 7, 7b, 7c and 8 known stable (no clinically significant adverse change in last 6 months) cardiovascular or coronary artery disease is acceptable.

11. History of malignancy within the last 1 year except for basal cell carcinoma, squamous cell skin cancer, superficial bladder tumors, or in situ cervical cancer. Participants with other treated malignancies who have no evidence of metastatic disease and >1 years without evidence of active malignancy may be entered following approval by the Sponsor Medical Monitor

12. History of major surgery within 3 months of Screening

13. Regular use of alcohol within one month prior to the Screening visit (i.e., more than fourteen units for females and twenty-one units for males per week [1 Unit = 150 mL of wine, 360 mL of beer, or 45 mL of 40% alcohol])

14. Cardiac troponin (troponin-I) above upper limit of normal at Screening

15. Recent (within 3 months) use of illicit drugs (such as cocaine, phencyclidine [PCP], MDMA,) or positive test for such drugs of abuse at Screening. Subjects who are on prescription medications that cause a positive result on urine drug screen will not be excluded. Subjects with a positive urine drug screen for cannabinoids will not be excluded.

16. Use of an investigational agent or device within 30 days prior to dosing or current participation in an investigational study

17. Any concomitant medical or psychiatric condition or social situation or any other situation that would make it difficult to comply with protocol requirements or put the participant at additional safety risk (For cohorts 5, 6, 7 and 8, stable diabetes mellitus based on PI discretion, requiring or not requiring insulin is not exclusionary)

18. Has a history of clinically meaningful coagulopathy, bleeding diathesis, stroke or myocardial infarction within 6 months of baseline, and/or concurrent anticoagulant medication(s)

19. Subjects with any of the following laboratory abnormalities:

    a. International normalized ratio (INR) > 1.5 × ULN at Screening

    b. Platelets < 100,000 at Screening

20. Participants who are unable to return for all scheduled study visits

21. Participants with any contraindications to MRI (Cohort 5 only).

22. Donation or loss of whole blood (excluding the volume of blood that will be drawn during the Screening procedures of this study) prior to administration of the study treatment as follows: 50 mL to 499 mL of whole blood within 30 days, or more than 499 mL of whole blood within 56 days prior to study treatment administration

When laboratory value cut offs are used for Inclusion or Exclusion, up to two repeat tests (after the initial Screening test) are acceptable and values from repeat testing may be used to determine study eligibility.

7.4 *Participant Withdrawal Criteria*

Participants will be advised that they are free to withdraw from the study at any time for any reason or, if necessary, the PI, or medically trained designee, may withdraw a participant from the study, per the following criteria, to protect the participant's health:

- the need to take medication which may interfere with study measurements;
- intolerable/unacceptable adverse experiences;
- major violation of or deviation from study protocol procedures;
- non-compliance of participant with protocol;
- participant unwilling to proceed and/or consent is withdrawn; or
- withdrawal from the study if, in the PI’s judgement, it is in the participant’s best interest.

The reasons for withdrawal will be recorded on the case report form (CRF) and included in the final clinical study report, along with any adverse events and any necessary medical treatment.

If a participant is withdrawn from the study due to significant AE or SAE, the PI, or medically trained designee, will evaluate the urgency of the event. If the situation warrants, the PI, or medically trained designee, will take appropriate diagnostic and therapeutic measures. If the situation is not an immediate emergency, the PI, or medically trained designee, at the clinical study facility will attempt to contact the Arrowhead Pharmaceuticals, Inc. Medical Monitor or medically qualified designee for consultation. No medical help, diagnosis, or advice will be withheld from the participant due to an inability to contact the Medical Monitor. The participant will be encouraged to remain available for follow-up medical monitoring. The Sponsor will be notified as soon as possible of any participant withdrawals.

Participants who are withdrawn or discontinue prior to EOS visit for reasons other than an adverse event, may be replaced at Sponsor discretion.

7.5 *Restrictions and Concomitant Medications*

1. ***Confinement:*** For each participant in Cohorts 1 through 4 and 2b through 4b, clinical facility confinement will be approximately 3 days, starting on Day -2, with discharge on Day 2 (after the 24-hour post-dose assessments) and dosing on Day 1. Cohorts 2b, 3b and 4b will be confined approximately 1.5 days at Day 29 (Day 29 through 24 hour PK sample collection.) For all other cohorts, clinical facility confinement will be approximately 2 hours on the dosing day unless additional monitoring at PI discretion is needed for safety reasons.

   Participants will return to the clinical facility for out-patient visits as per Schedule of Assessments. Participants will be observed post-second dose for 2-4 hours or as clinically indicated as per PI.

2. ***Fasting:*** On the day of dosing or on other days with blood draws, participants will have fasted from food for at least 8 hours prior to study treatment administration or blood draw unless otherwise specified or as otherwise required by study procedures (e.g. GTT and post-prandial triglycerides).

3. ***Recreational Drugs & Alcohol:*** Participants will be instructed to abstain from consuming alcohol for at least 48 hours prior to admission, and while confined to the clinical facility. In addition, participants will be instructed to refrain from regular use of alcohol (i.e., more than fourteen units for females and twenty-one units for males per week [1 Unit = 150 mL of wine, 360 mL of beer, or 45 mL of 40% alcohol]) for the study duration. Participants must abstain from use of recreational drugs throughout the study.

4. ***Concomitant Medications:*** Statins are acceptable when applicable by cohort. Use of fish oil, PCSK-9 inhibitors or fibrates are acceptable if subject has been on a stable regimen for at least 3 months and only in cohorts 5, 6, 7, 7b, 7c,8 and 9. Subjects who have used of fish oil or fibrates for less than 3 months prior to enrollment may still be eligible if a washout period of 5-half-lives is completed. For clarity, use of anti-hypertensive, anti-coagulants and anti-platelet agents are acceptable for Cohorts 5, 6, 7, 7b, 7c,8 and 9. Use of other concomitant medications may be approved by sponsor medical monitor and PI. Subjects will be instructed to inform the PI of the details (indication, dose and dates of administration) if they do take any medication, and these

details will be recorded in the CRF. If necessary, paracetamol may be used during the study as necessary.

8 INVESTIGATIONAL PRODUCT

8.1 *Description, Identification and Dosage*

Arrowhead Pharmaceuticals, Inc. is responsible for the supply of active drug supplies together with detailed instructions (in a pharmacy manual) describing preparation of ARO-ANG3. The PBO (normal saline 0.9%) will be supplied by the clinical site.

Accordingly, ARO-ANG3 will be supplied as single sterile 2-mL vials containing ARO-ANG3, with the correct dose of ARO-ANG3 prepared by the Pharmacy prior to dosing participants.

The placebo (PBO) will be 0.9% normal saline administered subcutaneously.

<u>Doses administered per Dose Level:</u>

Each single dose of either active drug (ARO-ANG3) or PBO (normal saline 0.9%), will be administered by subcutaneous injection. Injections will be made into the subcutaneous tissue at an appropriate site (e.g. abdomen, thigh, upper arm, etc.) using a 25-30 Gauge, ½ inch needle. The abdomen is the preferred site. Injection site is to be varied (no multiple injections into the same exact site. Alternating various locations on the abdomen is acceptable). Injection site location is to be recorded in the eCRF. Prior to dose administration, the ARO-ANG3 vial must be allowed sufficient time to come to room temperature. Do not inject into areas of active skin disease or injury such as sunburns, skin rashes, inflammation or skin infections. Injection volume per site should not exceed approximately 1.5 mL.

There will be no dose escalation within a cohort (i.e., the same drug dose will be administered to each participant within a cohort). An intermediate dose (i.e., between dose levels) or additional dose levels may be evaluated depending upon the safety profile with approval of the PI and EC and with input from the DSC. Each participant will only receive a single dose at the assigned dose level. The randomization schedule will be provided to each clinical site and will be maintained along with any other materials that could jeopardize the blind in a secured area of the pharmacy.

8.2 *Supply, Preparation, Storage and Labelling of ARO-ANG3*

ARO-ANG3 will be supplied as a sterile Type-1 glass 2.0-mL vial (1.2 mL nominal volume, 1.0 mL withdrawable volume).

Strength: 200 mg/mL

Appearance:	Clear, colorless to light yellow solution
Inactive ingredients:	0.5 mM sodium phosphate monobasic, 0.5 mM sodium phosphate dibasic in water for injection
Shipment and Storage:	Refrigerated, 2-8ºC

ARO-ANG3 will be prepared, per the Pharmacy Manual, by a pharmacist or qualified staff at the clinical sites. Aseptic technique will be used to ensure sterility of the solution to be injected. The time of preparation for active drug must be documented and tracked to demonstrate administration within prepared drug stability boundaries. Please refer to the Pharmacy Manual for more detailed instructions.

The investigational product vials will be labeled per Good Manufacturing Practice (cGMP)/Good Clinical Practice (cGCP).

Study drug supplies will be stored at clinical sites securely under the appropriate conditions.

8.3 *Study Drug Handling*

The Sponsor will provide the PI with a sufficient quantity of clinical drug supplies. The PI must ensure that deliveries of investigational product from the Sponsor are correctly received by a responsible person, that all receipts of drug shipments are recorded on the appropriate Drug Accountability forms prepared by the pharmacy at the clinical site and that the products are stored in a secure area under recommended storage conditions. It is also the responsibility of the PI to ensure that the integrity of packaged study product not be jeopardized prior to dispensing.

Only participants enrolled in the study may receive study drug, in accordance with all applicable regulatory requirements. Only authorized site staff may supply or administer study drug. The study drug must be stored in a secure area with access limited to the PI and authorized staff and under the physical conditions that are consistent with the study drug-specific requirements.

An authorized and trained staff member at each clinical trial site will dispense the study drug per predefined drug dispensing requirements. The dispensing and administration will be verified by a second member of site staff.

ARO-ANG3 will be supplied by Arrowhead Pharmaceuticals, Inc. and labeled with the drug name, batch number, expiration date (as applicable) and storage conditions. Individual doses will be dispensed by clinical trial site staff members on the morning of dosing and recorded in the drug accountability records. A Pharmacy Manual will be prepared to define the procedures for dispensing.

Standard Operating Procedures will be followed for the receipt, handling and accountability of the study formulations.

8.4 *Accountability of Study Supplies*

All material supplied is for use only in this clinical study and should not be used for any other purpose. The PI is responsible for the investigational product accountability, reconciliation and record maintenance at the investigational site. In accordance with all applicable regulatory requirements, the PI or designated site staff must maintain investigational product accountability records throughout the course of the study. This person will document the amount of investigational product received from Arrowhead Pharmaceuticals, Inc. and the amount administered to participants. A non-blinded Clinical Research Associate (CRA) will perform initial and ongoing study drug kit and placebo accountability. The non-blinded CRA will protect the integrity of the assignment blind and will not participate in data review for study participants. Used vials of ARO-ANG3 will be retained sequestered per participant and cohort (where allowable by local policy) and made available to the non-blinded CRA during study drug and placebo reconciliation.

A Drug Dispensing Log must be kept current and will contain the following information:

- the identification of the participant to whom the drug was dispensed; and
- the date(s) and quantity of the drug dispensed to the participant.

The date and time of dose preparation and release will be maintained to support administration of study drug/PBO. The authorized pharmacist or qualified staff will be un-blinded to the doses. The pharmacy will dispense the study medication and the study center will administer the study medication only to participants included in this study following the procedures set out in the study protocol. Each participant will be given only the study medication carrying his/her study number. Study drug administration will be documented on the CRFs and/or other study drug record. The inventory must be available for inspection by the non-blinded monitor during the study. Drug supplies, excluding partially used or empty containers, will either be collected at the end of the study by the study monitor or returned by the PI or designee to Arrowhead Pharmaceuticals Inc. or the designated Arrowhead approved depot.

8.5 *Retention of Investigational Product Vials*

For this study, used and partially used drug vials will be retained for an adequate period to allow accountability by the non-blinded CRA. No additional study drug samples will be retained.

8.6 *Allocation to Treatment*

All potential participants who sign an informed consent at Screening will receive a unique 6-digit number (i.e. a Screening Number). The first 3 digits will represent the assigned site number and will be the same for each participant that screens at an individual site. The next 3 digits will be assigned sequentially (starting with 001). For patients who are deemed eligible, this 6-digit screening number will become the subject's permanent study ID number.

Eligible NHVs in double-blind cohorts will be allocated a unique randomization number, in accordance with the randomization schedule. In each cohort, the first two subjects (sentinels) will be randomized separately to one active and one PBO. Each subject will be assigned to either active (ARO-ANG3) or PBO treatment. The allocation of active treatment or PBO will be performed using a block randomization algorithm.

Participants who drop out prior to their EOS visit for reasons other than an adverse event, may be replaced.

8.7 *Blinding and Code-break*

For blinded cohorts, blinding of study drug/PBO assignment is critical to the integrity of this clinical trial. It is expected that in most cases, AEs can be properly managed without the need for unblinding. However, in the event of a medical emergency in which knowledge of an individual participant's assignment is considered critical to the participant's well-being and management, the PI or documented designated treating physician may request permission to unblind the treatment assignment from the Arrowhead Pharmaceuticals, Inc. Medical Monitor. If the situation is not an immediate emergency, the PI should contact the responsible Medical Monitor to discuss the participant and circumstances requiring the unblinding. The blind will be broken only for the specific participant under discussion. Unblinding in situations that are not an immediate emergency may only take place with the notification and agreement of the responsible Medical Monitor. The randomization schedules will be maintained under controlled access. The personnel involved in the dispensing of investigational products will be accountable for ensuring compliance to randomization schedules. The non-blinded CRA will review the randomization schedule in comparison to the dispensing log to verify correct randomization.

If the PI considers an adverse event to be of such severity as to require immediate specific knowledge of the identity and dose of the relevant product, unblinding will be completed via IWRS system. The

'Medical Emergency Unblinding' form in IWRS is only accessible to the designated unblinded Pharmacist, PI and Sub-I. The study monitor should be informed promptly.

If a participant requires emergent unblinding (with or without a discussion between the Investigator and the Medical Monitor preceding the unblinding), the Investigator may also be required to complete a 'Drug Safety Unblinding Request/Notification Form' to document the medical rationale necessitating the unblinding. This form is then forwarded to the local Medical Monitor.

After the completion of the final study visit (not including 90-day follow-up phone call) for each cohort, unblinding for Sponsor analysis will occur at Sponsor discretion. However, the site will remain blinded to treatment assignment.

Sponsor **may** request an interim descriptive analysis of the change from baseline in ANGPTL3, apoC-III and other measured lipid parameters any time after all NHV subjects planned for enrollment in each cohort have received at least one dose of ARO-ANG3 or PBO. This interim analysis is for planning of future studies and will not impact the conduct of this study. Sponsor will remain blinded to all subject treatment assignments. Descriptive statistics (that does not inadvertently unblind the trial) for change from baseline in pharmacodynamic measures will be calculated for all active subjects per cohort and for a pooled PBO group by an unblinded statistician and provided to Sponsor. For any AEs occurring more than once, the frequency of AEs for a specific preferred term will be calculated for pooled active and pooled PBO groups in such a way not to inadvertently break the subject-blind of the trial.

9 STUDY METHODS AND SCHEDULES

9.1 *Overview of Procedures*

Participants, who have consented to participate, had the screening examination and have met all of the protocol eligibility criteria will be randomized to receive subcutaneous injections of either PBO or ARO-ANG3 in double-blind fashion (for Cohorts 1 through 4, 5 and 6), or may be enrolled into an open-label cohort to receive ARO-ANG3 (Cohorts 2b-4b, 7, 7b, 7c,8 and 9). Cohorts 1, 2, 3, and 4 are each made up of 10 eligible participants (4 PBO, 6 active ARO-ANG3). Cohorts 5 and 6 will be made up of up to 9 subjects (3 PBO, 6 active ARO-ANG3); and Cohorts 7, 7b, 7c and 8 will be made up of up to 6 subjects all receiving ARO-ANG3. Up to 18 patients from Cohorts 7, 7b and 7c may elect to continue to receive up to four 200 mg doses administered approximately every 12 weeks. Cohorts 2b, 3b, 4b will be made up of 4 healthy volunteers all receiving ARO-ANG3. Subjects will be evaluated per **Figure 1**, starting at dose level 1 (35 mg) for Cohort 1. Cohorts 1, 2, 3, and 4 will begin with administration of ARO-ANG3

or PBO to two sentinel participants (one ARO-ANG3, one PBO). Following the Day 3 evaluation in these participants, if there are no significant safety concerns based on PI discretion, the remaining participants in the cohort may be treated.

At regular intervals during the study, participants will undergo the following evaluations: medical history, physical examinations, vital sign measurements (blood pressure, temperature, heart rate, respiratory rate), weight, adverse events monitoring, ECGs, pregnancy test (females), concurrent medication, sample collection for HDL, LDL and VLDL cholesterol, triglycerides and other specified lipid or metabolic parameters, and blood sample collection for hematology, serum lipase, hemoglobin A1C, coagulation, chemistry analysis and urinalysis. Blood samples will also be collected from each participant for pharmacokinetic analysis. Abdominal MRI (MRI-PDFF) will be completed to evaluate for changes in liver fat fraction.

Clinical facility confinement will begin on Day -2 with discharge on Day 2 (following the 24-hour post-dose assessments) for all subjects except those that are enrolled into Cohorts 5, 6, 7, 7b, 7c, 8 and 9. Cohorts 2b, 3b and 4b will be confined approximately 1.5 days at Day 29 (Day 29 through 24 hour PK sample collection.) Out-patient visits to the Clinical Facility will occur as per the Schedule of Assessments. A telephone follow-up will occur on Day 90 (± 5) post last dose to verify compliance with contraceptive measures and absence of any known pregnancy. Clinically significant changes including adverse events will be followed until resolution is achieved or considered medically stable. Participants will have fasted from food for at least 8 hours pre-dose. Meals and water will be provided while participants are confined at the clinical facility. Refer to Schedule of Assessments for additional information.

The PI (or medically qualified designee) will be required to remain within the clinical study facility for 2 hours after dosing on dosing visits and will remain on call for the duration of the study. Participants should refrain from strenuous physical activities throughout the study.

9.2 *Selection and Screening*

Prior to commencement of any screening procedures, the PI, or designee, will inform the participant about the nature and purpose of the study, including the risks and benefits involved, possible AEs, the fact that their participation is voluntary and provide a copy of the EC-approved Informed Consent Form for review. Each participant will acknowledge receipt of this information by giving written informed consent for their involvement in the study in the presence of the PI, or designee, who will also sign and date the Informed Consent Form. The original signed consent form will be retained by the PI and a copy

of the original will be given to the participant. Informed consent will be performed per the Principles of the International Conference on Harmonisation (ICH) Good Clinical Practice (cGCP) procedures.

Having given Informed Consent, potential participants will undergo procedures outlined in the Schedule of Assessments, to be performed within 60 days of the scheduled dosing date, to determine that they meet the inclusion/exclusion criteria specified in Sections 7.2 and 7.3.

9.3 *On-Study Procedures/Assessments*

9.3.1 *Study Procedures: Clinical Facility Confinement*

For subjects confined overnight, eligible participants will present at the Clinical Facility on Day -2 after an 8 hour fast. Pre-dose GTT (optional in patients with diabetes mellitus) will be conducted on Day -2 and pre-dose post-prandial triglyceride testing will be conducted on Day -1 or vice-versa. Note that study dose administration is on Day 1, which must occur within 60 days of screening. Participants will be confined to the clinical facility until after the 24-hour post-dose (Day 2) assessments. Cohorts 2b, 3b and 4b will be confined approximately 1.5 days at Day 29 (Day 29 through 24 hour PK sample collection.)

On arrival at the clinical facility on Day -2, the PI, or designee, will meet with participants to reiterate all study procedures and encourage participants to ask any questions. All participants shall undergo a check-in procedure during which questions will be asked regarding protocol compliance and safety monitoring.

Documentation of the participant's fulfillment of the entry criteria, for all participants considered for the study and subsequently included or excluded, is to be completed by the PI, or medically qualified designee. Documentation of screening failure details will be recorded using eligibility screening forms or a participant screen failure log. Procedures outlined in the Schedule of Assessments will be performed. Meals and water will be provided while participants are confined at the clinical facility. Timing will abide by fasting restrictions outlined in Section 7.5.

9.3.2 *Demographics/Medical History*

Medical History will include medication use over the previous 30 days, including vitamins, over-the-counter medications, prescription drugs, recreational drugs or supplements and alcohol and tobacco use.

9.3.3 *Physical Exam*

A complete physical exam will be performed at Screening and as per Schedule of Assessments. At Screening, height (centimetres, without shoes) and weight (kilograms, without shoes) will be obtained to determine BMI. At all other time points outlined in the Schedule of Assessments, a symptom-directed physical exam will performed if indicated.

9.3.4 *Glucose Tolerance Test (GTT) & post-prandial Triglyceride Test*

GTT and post-prandial triglyceride testing will be measured at time points outlined as per the Schedule of Assessments in accordance with site procedural standards for all applicable cohorts. Subjects are required to consume ≥ 25% of the high fat/high carbohydrate meal provided by the site. The percentage of the consumed meal shall be recorded in the subject's source documents. The same percentage of the meal should be consumed by the subject post dose at Day 85 +/- 3 days for consistency. Post consumption blood samples should be drawn 2 hours from the end of the meal +/- 10 mins. In Cohort 8, post-prandial TG evaluation is only to be completed in patients who are not at risk for post-prandial hypertriglyceridemia related abdominal pain or pancreatitis. GTT is optional in any patient with diabetes mellitus.

9.3.5 *Magnetic Resonance Imaging*

MRI using MRI-PDFF for the evaluation of % liver fat will be conducted at time points outlined in the Schedule of Assessments in accordance with procedural standards detailed in the Imaging Acquisition Manual for all applicable cohorts. MRI protocol used should be consistent across all individual patient visits (same MRI imaging technique/protocol will be used pre-dose, Day 71, Day 168 on a patient by patient basis). Please refer to the Imaging Acquisition Manual for further instructions.

9.3.6 *Electrocardiogram*

A single 12-lead ECG measurement will be obtained at time points outlined in the Schedule of Assessments after the participant is semi-supine for at least 3 minutes. Any clinically-significant abnormal ECGs will be repeated in triplicate, with each measurement approximately 1 minute apart. ECGs will be performed prior to venepuncture and other invasive procedures.

9.3.7 *Vital Sign Assessments*

Systolic/diastolic blood pressure, temperature, heart rate, respiratory rate (breaths/min) will be obtained at time points outlined in the Schedule of Assessments after the participant is semi-supine for at least 3 minutes. Vitals signs will be obtained prior to venepuncture and other invasive procedures.

9.3.8 *Clinical Laboratory Tests*

Blood and urine samples will be collected to perform clinical laboratory tests. Participants will be required to fast for the screening and other pharmacodynamic sample collections.

At the screening visit, up to 60 days prior to the first dose of study medication, a blood and urine sample will be collected for the laboratory tests detailed below, to establish baseline data and eligibility for enrolment. One repeat Screening lab draw is allowed per assessment to establish eligibility. The results will be assessed by the PI, or medically qualified designee, before study enrolment. Any abnormality in laboratory values (that are confirmed on repeat) deemed clinically significant by the PI, or medically qualified designee (i.e., those that would jeopardize the safety of the participant or impact on the validity of the study results), will result in exclusion of that participant. Clinical laboratory tests will be performed on participants' blood and urine at specified time-points listed in the Schedule of Assessments.

The Day 1 value will be used as each participant's baseline value for data analysis purposes or as otherwise specified. If Day 1 or as otherwise specified values are erroneous or not available and repeat blood draw is not possible, Screening value may be used as baseline.

Biochemistry**:** Sodium, potassium, chloride, bicarbonate, glucose, urea, creatinine (including calculated creatinine clearance), creatine kinase, uric acid, phosphate, total calcium, anion gap, cholesterol, albumin, globulins, protein, total bilirubin, lipase, hemoglobin A1C, C-peptide, conjugated bilirubin, gamma glutamyltransferase (GGT), alkaline phosphatase (ALP), alanine aminotransferase (ALT), aspartate transaminase (AST), lactate dehydrogenase (LD), triglycerides, C-reactive protein and Troponin I.

Hematology: Hemoglobin, red blood cell count (RBC), hematocrit, mean cell volume (MCV), mean cell hemoglobin (MCH), mean cell hemoglobin concentration (MCHC), platelets, white cell count, neutrophils, lymphocytes, monocytes, eosinophils and basophils.

Coagulation: Partial thromboplastin time (PTT), Prothrombin time (PT) with INR and Fibrinogen.

Urinalysis: Leucocytes, nitrites, urobilinogen, protein, pH, blood, specific gravity, ketone, bilirubin and glucose. Creatinine will be measured only on select timepoints that PK (Urine) is examined (Day 1 (predose), 8, 15, 22, 29 (predose), including separate analysis of 0-6 hour and 6-24 hour post-dose cumulative samples taken after dosing on Day 1 and 29).

Microscopic urinalysis will be performed if indicated: White Blood Cells, Red Blood Cells, Epithelial cells, Bacteria.

Serology: Hepatitis B surface antigen, Hepatitis C antibody and HIV antibody screen. If necessary, participants will be counseled by the PI, or medically trained designee, concerning the blood tests for Hepatitis B surface antigen, Hepatitis C and HIV antibodies, and their subsequent results.

FSH: Post-menopausal status will be confirmed by follicle-stimulating hormone (FSH) level consistent with post-menopausal state.

Drug and Alcohol Use Screen: Urine drug screen for Benzodiazepines, Amphetamines, Barbiturates, Methamphetamines, Methadone, Opiates, Phencyclidine, Cannabinoids, MDMA and Cocaine. Alcohol Breath Test will be done to test for alcohol consumption.

Pregnancy: Females of childbearing potential will have a urine pregnancy test. If urine pregnancy test is positive, patient will be referred to their primary care provider for follow up.

Lipid Parameters: The following lipid parameters will be measured as per the Schedule of Assessments: Fasting ANGPTL3, LDL-C, Total Cholesterol, non-HDL-C, HDL-C, VLDL-C, Triglycerides, Lp(a), apoB-100, apoB-48, apoC-III, apoC-II, apoA-I, lipoprotein lipase mass (if feasible), hepatic lipase mass (if feasible), CETP mass (if feasible), apoA-V, lipid metabolic genotype (drawn on all consenting subjects but analyzed only if scientifically warranted at the discretion of the sponsor).

Serum insulin levels: Insulin levels will be measured as per the Schedule of Assessments.

Serum glucose levels: Blood glucose levels will be measured as per the Schedule of Assessments. Glucose level included in metabolic panel is acceptable.

9.3.9 *Pharmacokinetics*

Samples for analysis of circulating ARO-ANG3 will be obtained at time points following study drug administration as outlined in the Schedule of Assessments (plasma and urine). ARO-ANG3 metabolites will be identified in pooled samples taken at timepoints per the schedule of assessments.

9.3.10 *Concomitant Medications/Therapies*

Participants will be instructed to inform the PI of the details (indication, dose and dates of administration) if they do take any medication, and these details will be recorded in the CRF. If necessary, paracetamol may be used during the study as necessary. Any other medication or therapy other than blood pressure medication must be approved by the Medical Monitor or PI prior to administration. Statins are allowed as applicable in the study.

9.3.11 *Follow-Up Procedures: Pregnancy Follow-Up Telephone Call (90 days (± 5 days) post last dose)*

Document telephone contact with each participant to verify compliance with contraceptive measures and absence of any known pregnancy. Information regarding any reported pregnancy should be collected for at least 1 year after birth or longer if it is decided that addition follow-up is required or until the end of the pregnancy. For Cohort 9 patients, pregnancy follow-up will be assessed at Day 449 EOS visit.

9.3.12 *Early Termination Procedures*

The reason for Early Termination will be documented in source documents and eCRF. Procedures as outlined in the Schedule of Assessments will be completed.

9.4 *Allocation of Formulations*

In Cohorts 1, 2, 3, and 4, 6 participants will receive active treatment and 4 participants will receive PBO. In Cohorts 5 and 6, 6 participants will receive active treatment and 3 participants will receive PBO. In Cohorts 2b, 3b, 4b, 7, 7b, 7c, 8 and 9, all participants will receive active treatment. Treatments will be administered per the randomized (where applicable) sequence or sequential number (if open-label) kept by the pharmacy or in a secure place at the clinical site, under control of the un-blinded staff member.

9.5 *Study Formulation Administration*

Appropriately trained employees of the clinical site will administer the study treatment. Each dose will be administered as a single subcutaneous injection. Two separate subcutaneous injections, each at a separate injection site may be used for higher dose volumes (e.g. > 1.5 mL injection volume). The date, time and location of administration will be recorded in the source notes and witnessed by a second person from the clinical facility. The site of injection will be marked and mapped for later observation. The preferred site of injection is the abdomen. Optional additional sites are the upper arms and thighs.

Table 2: Injection number and volume per cohort

Cohort	Dose	Concentration	Total Injection Volume	# Injections per planned dose
1	35 mg	200 mg/mL	0.175 mL	Single
2	100 mg	200 mg/mL	0.5 mL	Single
2b	100 mg	200 mg/mL	0.5 mL	Single
3	200 mg	200 mg/mL	1.0 mL	Single
3b	200 mg	200 mg/mL	1.0 mL	Single
4	300 mg	200 mg/mL	1.5 mL	Single
4b	300 mg	200 mg/mL	1.5 mL	Single
5	200 mg	200 mg/mL	1.0 mL	Single
6	200 mg	200 mg/mL	1.0 mL	Single
7	200 mg	200 mg/mL	1.0 mL	Single
7b	100 mg	200 mg/mL	0.5 mL	Single
7c	300 mg	200 mg/mL	1.5 mL	Single
8	200 mg	200 mg/mL	1.0 mL	Single
9	200 mg	200 mg/mL	1.0	Single

9.6 *Timing of Treatments and Procedures*

Actual times of procedures for each participant will vary depending on scheduling and will be recorded in the CRF.

In the event of multiple procedures scheduled at the same time, non-invasive procedures (i.e. ECGs, AE assessment) will be conducted prior to invasive procedures (i.e., blood sample collection). Timing of activities may be adjusted slightly to accommodate all procedures.

The following windows are allowed for study assessments/visits:

Pre-dose:	Within 120 minutes prior to dosing
Plasma PK/PD through 6 hours post dose:	± 2 minutes
All other procedures through 6 hours post dose:	± 10 minutes
Plasma PK/PD from 8 to 48 hours post dose:	± 5 minutes
All other procedures from 8 to 48 hours post dose:	± 15 minutes
Day 8:	± 8 hours
Day 15 to Day 29:	± 3 day
Visits beyond Day 29:	± 3 days
Visits Beyond Day 113	± 10 days
Pregnancy F/U Phone Call: (90 days post last dose)	± 5 days

9.7 *Safety Measurements*

The safety of ARO-ANG3 will be evaluated by collection of the following measurements performed at specified time points:

- Monitoring of AEs/SAEs
- Physical examinations
- Vital signs
- ECG measurements
- Injection Site Reactions (Mild, Moderate or Severe): Photographic images will be taken of all injection site reactions at the time of reporting and at the time of resolution.
- Clinical laboratory tests (hematology, chemistry, hemoglobin A1C, coagulation, lipase, urinalysis, Stool occult blood test)
- Concomitant medications/therapy, and
- Reasons for treatment discontinuation due to toxicity

The AE/SAE reporting period for an enrolled participant will begin when the participant provides informed consent. Treatment-Emergent AEs/SAEs will be those defined as following dose administration. All AEs/SAEs that occur during the AE reporting period specified in the protocol must be reported to Arrowhead Pharmaceuticals, Inc., regardless of the relationship of the AE to study treatment. Any known untoward event that occurs beyond the AE reporting period that the PI considers an SAE and possibly related to study treatment will be reported to Arrowhead.

9.8 *Blood Sampling for Pharmacokinetic, Pharmacodynamic Analysis*

Blood samples will be collected from participants through an indwelling cannula or through a fresh vein puncture. The actual blood collection time will be recorded in the source documents. All deviations outside the range allowed above will be documented as protocol deviations. In all such cases, appropriate time corrections, for the actual time of sample collection will be incorporated at the time of data analysis. Blood samples will be collected at time points outlined in the Schedule of Assessments.

The target sample times will be printed in the CRFs. The actual sample times (times samples taken) will be recorded alongside the nominal times in the CRF and will be entered at the time of or as soon as possible after sampling. All times must be recorded in the 24-hour format. An explanation must be given for any blood sample taken outside of the set sampling times.

If a subject’s serum ANGPTL3 level has not returned to above 50% of baseline value by EOS then additional monthly follow up visits may be completed (per Sponsor discretion) until serum ANGPTL3 level is above 50% of baseline. The sampling at these optional follow-up visits would include ANGPTL3 measurements, along with lipid parameters described (LDL-C, HDL-C and Triglycerides).

9.8.1 Sample Processing and Analysis for Pharmacokinetic Samples

Approximately 5 mL of whole blood will be collected and processed per the Laboratory Manual.

Plasma samples will be assayed by a validated hybridization-ligation method. The criteria for repeat analysis, as defined in the respective in-house procedure, will be followed.

The validation study conducted by the appointed bioanalytical laboratory to establish validity including accuracy, precision, reproducibility, specificity, recovery and frozen stability of the analytical method will be appended to the final report.

10 ADVERSE EVENTS

The PI and clinical facility staff are responsible for detection, recording and reporting of events that meet the criteria and definition of various adverse events as listed below. Adverse events will be recorded from time of signed consent through to end of study; only AEs that occur post-dose will be considered treatment-emergent. The PI and clinical facility staff are responsible for detection, recording and reporting of pregnancy and appropriate follow up.

10.1 *Definitions*

An **Adverse Event (AE)** is any untoward medical occurrence in a patient or clinical investigation subject administered a pharmaceutical product and which does not necessarily have to have a causal relationship with this treatment. An AE can therefore be any unfavorable and unintended sign (including an abnormal laboratory finding or diagnostic test), symptom, or disease temporally associated with the use of a medicinal (investigational/experimental) product, whether related to this product or not. (Refer to International Conference on Harmonisation [ICH] E2a: Clinical Safety Data Management: Definitions and Standards for Expedited Reporting, 27 October 1994).

Treatment emergent AEs will be defined as AEs with onset after administration of the study drug, or when a preexisting medical condition increases in severity or frequency after study drug administration.

AEs will not include:

- A medical or surgical procedure such as surgery, endoscopy, tooth extraction, or transfusion (although the condition that leads to the procedure may be an AE)
- A pre-existing disease or condition present at the start of the study that does not worsen during the study
- Any situation where an untoward medical occurrence has not occurred (for example, hospitalizations for cosmetic elective surgery or "social" admissions)
- An overdose of either the investigational product or a concurrent medication without any resulting signs or symptoms.

A **Serious Adverse Event (SAE)** is an AE that:

- Results in death,
- Is life-threatening, (NOTE: The term 'life-threatening' in the definition of 'serious' refers to an event/reaction in which the participant was at immediate risk of death at the time of the

event/reaction; it does not refer to an event/reaction which hypothetically might have caused death, if it were more severe)

- Requires inpatient hospitalization or prolongation of an existing hospitalization
- Results in persistent or significant disability/incapacity
- Is a congenital anomaly/birth defect
- Is a medically important event or reaction

Medical and scientific judgment should be exercised in deciding whether other situations, should be considered serious such as important medical events that may not be immediately life-threatening or result in death or hospitalization but might jeopardize the participant or might require medical or surgical intervention to prevent one of the other serious outcomes listed in the above definition. These should also be considered serious. Examples of such events are intensive treatment in an emergency room or at home for allergic bronchospasm, blood dyscrasias or convulsions that do not result in hospitalization, or development of drug dependency or drug abuse.

10.2 *Clinical Laboratory Abnormalities and Other Abnormal Assessments as AEs*

Abnormal assessments (e.g., ECGs and vital signs) that are judged by the PI as clinically significant or result in clinical sequelae will be recorded as AEs. Laboratory abnormalities will be reported by the Investigator as AEs if the abnormality is considered clinically significant or result in clinical sequelae. Laboratory abnormalities not reported as AEs are not to be reported as Clinically Significant (CS) in the study database.

Clinically significant abnormal laboratory findings or other abnormal assessments that are detected during the study or are present at baseline and significantly worsen following the start of the study will be reported as AEs.

The PI (or medically qualified designee) will exercise his or her medical and scientific judgment in deciding whether an abnormal laboratory finding or other abnormal assessment is clinically significant.

10.3 *Timing, Frequency, and Method of Detecting AEs*

Any pre-existing conditions or signs and/or symptoms present in a participant prior to the start of the study (i.e., before informed consent) should be recorded as Medical/Surgical History.

All AEs occurring after informed consent and on or before the final visit must be reported as AEs; only AEs that occur post-dose will be considered treatment-emergent. All AEs must be recorded irrespective of whether they are considered drug-related.

At each visit/assessment in the period defined above, AEs will be evaluated by the PI (or medically qualified designee) and recorded.

10.4 *Recording of AEs*

When an AE occurs, it is the responsibility of the PI or medically qualified designee to review all documentation (e.g., hospital progress notes, laboratory, and diagnostics reports) relative to the event. The PI or medically qualified designee will then record the AE on the AE CRF. Additional reporting requirements for an AE meeting serious criteria are discussed in Section 10.7 below.

The PI or medically qualified designee will attempt to establish a diagnosis of the event based on signs, symptoms, and/or other clinical information. In all cases, when available, the diagnosis should be reported as the event and not the individual signs/symptoms. It is not acceptable for the PI to send photocopies of the subject's medical records to the Sponsor in lieu of completion of the appropriate AE CRF pages.

10.5 *Evaluating AEs*

10.5.1 Assessment of Intensity

The PI or medically qualified designee will assess intensity for each AE reported during the study. The assessment will be based on the PI's (or medically qualified designee's) clinical judgment. The intensity should be assigned to one of the following categories:

Mild: An event that is easily tolerated by the participant, causing minimal discomfort and not interfering with everyday activities.

Moderate: An event that is sufficiently discomforting to interfere with normal everyday activities.

Severe: An event that prevents normal everyday activities.

An AE that is assessed as severe should not be confused with a SAE. Severity is a category utilized for rating the intensity of an event; and both AEs and SAEs can be assessed as severe. An event is defined as 'serious' when it meets one of the predefined outcomes as described in Section 10.1.

10.5.2 Injection site reactions

- Injection site reactions will be graded as either Mild, Moderate or Severe. For purposes of data analysis, a local injection site reaction (LISR) is defined as an adverse reaction (usually immunologic) developing at the site of injection and lasting at least 48 hours and based on the specified MedDRA preferred times is provided in 16.2. Injection site reactions are graded Mild, Moderate or Severe based on symptoms. Photographs of local reactions around injection site should be obtained at the time of reporting and at the approximate time of resolution. For data analysis purposes AEs at the injection site with reported terms of bruising or hematoma will not be considered injection site reactions. Mild: Tenderness with or without associated symptoms (e.g., warmth, erythema, itching), mild pain or mild edema.
- Moderate: Pain with associated phlebitis or lipodystrophy
- Severe: Tissue ulceration or necrosis with associated severe tissue damage or if operative intervention is indicated

10.5.2 Assessment of Causality

The PI (or medically qualified designee) is obligated to assess the relationship between investigational product and the occurrence of each AE. The PI (or medically qualified designee) will use clinical judgment to determine the relationship. Alternative causes, such as natural history of the underlying diseases, concomitant therapy, other risk factors, and the temporal relationship of the event to the investigational product will be considered and investigated. The PI (or medically qualified designee) will also consult the Investigator's Brochure in the determination of his/her assessment.

There may be situations when an SAE has occurred and the PI has minimal information to include in the initial SAE report. However, it is very important that the PI (or medically qualified designee) always assess causality for every event prior to transmission of the SAE report form. The PI (or medically qualified designee) may change his/her opinion of causality considering follow-up information, amending the SAE report form accordingly. The causality assessment is one of the criteria used when determining global regulatory reporting requirements.

The PI (or medically qualified designee) will provide the assessment of causality utilizing three possible categories: Not Related, Possibly Related and Probably Related.

An AE will be considered "not related" to the use of the product if any of the following tests are met:

- An unreasonable temporal relationship between administration of the product and the onset of the AE (e.g., the event occurred either before, or too long after administration of the product for it to be considered product-related);

- A causal relationship between the product and the AE is biologically implausible (e.g., death as a passenger in an automobile accident)

- A clearly more likely alternative explanation for the AE is present (e.g., typical adverse reaction to a concomitant drug and/or typical disease-related event)

An AE will be considered "Possibly related" when there is a reasonable possibility that the incident, experience, or outcome may have been caused by the product under investigation.

An AE will be considered "Probably related" when there are facts, evidence, or arguments to suggest that the event is related to the product under investigation.

10.6 *Follow-up of AEs*

After the initial AE, the PI is required to proactively follow each participant and provide further information on the participant's condition as deemed appropriate.

All AEs will be followed until resolution, until the condition stabilizes, until the event is otherwise explained, or until the participant is lost to follow-up. Once resolved, the appropriate AE CRF page and SAE report form (if event is serious) will be updated. The PI, or medically qualified designee, will ensure that follow-up includes any supplemental investigations as may be indicated to elucidate the nature and/or causality of the AE or SAE. This may include additional laboratory tests or investigations, histopathological examinations, or consultation with other health care professionals. In the event of a fatal outcome in an SAE, the PI, or medically qualified designee, will attempt to obtain postmortem findings, including histopathology, and provide all additional information in a follow up SAE report.

New or updated information regarding an SAE will be recorded on a new SAE report form marked as follow-up with the appropriate follow-up number added to the report. The follow-up report will be signed and dated by the PI.

10.7 *Prompt Reporting of SAEs*

AEs meeting serious criteria MUST be reported promptly to the designated Pharmacovigilance CRO, and the EC.

10.7.1 Completion and Transmission of the SAE reports

Once an Investigator becomes aware that an SAE has occurred in a study participant, she/he will report the information on an SAE report form to the designated Pharmacovigilance CRO within 24 hours. The SAE report form will always be completed as thoroughly as possible with all available details of the event and signed by the PI (or medically qualified designee). If the PI does not have all information regarding an SAE, he/she will not wait to receive additional information before reporting the event. The SAE report form will be updated when additional information is received.

The PI (or medically qualified designee) will always provide an assessment of causality at the time of the initial report as described in Section 10.5.2.

Facsimile or email transmission of the SAE report form are the preferred methods to transmit this information to the designated Pharmacovigilance CRO. In rare circumstances, notification by telephone is acceptable, with a copy of the SAE CRF sent by overnight mail. Initial notification via the telephone does not replace the need for the PI, or medically qualified designee, to complete and sign the SAE report form within the outlined time frames.

The Sponsor will provide a list of project contacts for SAE receipt, fax numbers, telephone numbers, and mailing addresses. Any event that in the opinion of the PI may be of immediate or potential concern for the participant's health or well-being will be reported to the Sponsor emergency contact listed below.

Sponsor Emergency Contact
[redacted]

10.7.2 Serious Adverse Event Reports to the EC

The PI, or responsible person per local requirements, will comply with the applicable local regulatory requirements related to the reporting of SAEs to regulatory authorities and the EC.

10.8 *Regulatory Requirements for Reporting of SAEs*

The PI (or medically qualified designee) will promptly report all SAEs in accordance with the procedures detailed in Section 10.7. Prompt notification of SAEs by the PI **is essential** so that the Sponsor may comply with its regulatory obligations.

10.9 *Post-study AEs*

A post-study AE is defined as any event that occurs outside of the AE detection period defined in Section 10.3.

Investigators are not obligated to actively seek AEs in former study participants. However, if the Investigator learns of any SAE, including a death, at any time after a participant has been discharged from the study, and he/she considers the event reasonably related to the investigational product, the PI will promptly notify Arrowhead.

10.10 *SAEs Related to Study Participation*

An SAE considered related to study participation (e.g., procedures, invasive tests, a change in existing therapy), even if it occurs during the pre- or post-treatment period, will be reported promptly (refer Section 10.7).

11 DATA ANALYSIS AND STATISTICAL CONSIDERATIONS

11.1 *Sample Size Considerations*

This study represents a proof of principle study, and as such no formal sample size calculation was performed. Results from this study will be utilized in sample size calculations for subsequent studies.

11.2 *Screening Data*

Demographics will be tabulated by participant and summarized by cohort and treatment group. Eligibility assessments at baseline, including medical/surgical history data and physical examination data (including height and weight), will be listed for each participant.

11.3 *Safety/Tolerability Data*

In general, safety analyses will be performed and the results summarized by-cohort and treatment group.

Treatment-emergent AEs will be summarized using the latest version of MedDRA by System Organ Class (SOC) and Preferred Term (PT), classified from verbatim terms. The incidence and percentage of participants with at least 1 occurrence of a PT will be included, per the most severe grade using a 3-point scale (mild, moderate, severe). The number of events per Preferred Term will also be summarized. Causality (relationship to study treatment) will be summarized separately.

The incidence and frequency of AEs, SAEs, related AEs, related SAEs and AEs leading to withdrawal, dose modification, or treatment discontinuation will be summarized by dose and treatment group per SOC and Preferred Terms. AEs will also be summarized in listings. The duration of AEs will be determined and included in listings, along with the action taken and outcome.

The incidence of laboratory abnormalities will be summarized. Results for variables that are not coded will be presented in the listings as “below, within, and above” the normal limits of the laboratory. Pregnancy test results will be summarized separately by time point.

Vital sign measurements will be summarized at each scheduled time point using descriptive statistics. Physical examination findings will be summarized by time point and presented in subject listings.

ECG parameter changes overall, changes from baseline and qualitative assessments will be summarized.

11.4 *Pharmacokinetic Data*

Plasma concentrations of ARO-ANG3 collected at specified time points post-dose from all participants at different dose levels will be used to calculate the following single dose pharmacokinetic parameters:

AUC_{0-24}:	The area under the plasma concentration versus time curve from the zero to 24 hours.
AUC_{inf}:	The area under the plasma concentration versus time curve from zero to infinity.
C_{max}:	The maximum plasma concentration will be obtained directly from the plasma concentration time profile.
t_{max}:	The time to maximum plasma concentration will be obtained by inspection.
$t_{½}$:	The half-life will be calculated by the equation $t_{½} = \ln(2)/k_{el}$.

The pharmacokinetic parameters will be determined using non-compartmental method(s). Descriptive statistics of pharmacokinetic parameters will include mean, standard deviation (SD), and coefficient of variation (CV), minimum and maximum. Dose-related trends in pharmacokinetic parameters will be assessed.

Pharmacokinetic parameters will be tabulated and summarized by dose level. The concentration-time profiles for each participant and the mean concentration-time profiles by dose level will be plotted with concentration presented on both linear and logarithmic scales.

Statistical analysis will be performed on the pharmacokinetic parameters using validated statistical software.

11.5 *Pharmacodynamic Data*

Blood collected for pharmacodynamic analysis following a single and multiple doses of ARO-ANG3 at different dose levels will undergo analysis for changes in the following lipid parameters: Fasting ANGPTL3, LDL-C, Total Cholesterol, non-HDL-C, HDL-C, VLDL-C, Triglycerides, Lp(a), apoB-48, apoB-100, apoC-II, apoC-III, apoA-I, apoA-V, lipoprotein lipase mass (if feasible), hepatic lipase mass (if feasible), CETP mass (if feasible). All samples should be drawn after at least an 8-hour fast. Fasting blood glucose and serum insulin levels will also be evaluated. For each of the listed parameters, percentage change and absolute change in measured levels from Day 1 pre-dose baseline to nadir will be analyzed and summarized by dose cohort and treatment group. Duration of response from nadir back to at or above 30% of baseline if occurring before EOS will be analyzed and summarized by dose cohort and treatment group. If a subject's serum ANGPTL3 level has not returned to above 50% of baseline value by EOS then additional monthly follow up visits **may** be completed (per Sponsor discretion) until serum ANGPTL3 level is above 50% of baseline. The sampling at these optional follow-up visits would include ANGPTL3 measurements, along with lipid parameters described (LDL-C, HDL-C and Triglycerides).

Changes in pre-dose glucose tolerance and insulin tolerance will be compared to baseline and descriptive statistics for change from baseline will be determined.

Descriptive statistics for change from baseline to post-dose as described in the SOA for fat fraction on MRI-PDFF, and GTT will also be determined.

11.6 *Data Recording and Quality Control*

Source documents must be maintained for each participant in the study, consisting of all demographic and medical information, including clinical laboratory data, etc. A copy of the signed informed consent form must be retained. All information on the e-CRFs must be traceable to these source documents in the participant's file.

Data recorded in all participants' eCRFs will be subjected to a quality control review.

12 STUDY APPROVAL AND CONDUCT

The following conditions will be met.

12.1 *Regulatory Approval*

The requirements for the conduct of clinical trials in accordance with local applicable regulations will be met before commencement of this study.

12.2 *Ethics Committee (EC) Approval*

Prior to initiation of the study, written EC approval of the Protocol and Informed Consent Forms, based on the principles of ICH cGCP procedures, will be received. A copy of the signed and dated letter of approval will be provided to the clinical site and Arrowhead Pharmaceuticals, Inc. prior to study commencement. Any written information and/or advertisements to be used for volunteer recruitment will be approved by the EC prior to use. A list of the EC voting members, their titles or occupations, FWA number (where applicable) and their institutional affiliations will be requested before study initiation.

Protocol modifications that may impact subject safety or the validity of the study will be approved by the EC, following written agreement from the Sponsor.

12.3 *Ethical Considerations*

This study will be carried out per the Declaration of Helsinki 1964, as modified by the 64th World Medical Assembly, Fortaleza, Brazil, October 2013, the Notes for Guidance on Good Clinical Practice (cGCP) (2000) (CPMP/ICH/135/95), and the Principles of the ICH cGCP. The protocol will be submitted for approval to the EC, and written approval obtained before subjects are enrolled. The composition of the EC will also be provided to the Sponsor. If approval is suspended or terminated by the EC, the PI will notify the Sponsor immediately.

Where applicable, the clinical site and Arrowhead Pharmaceuticals, Inc. agree to abide by the local compensation guidelines for injury resulting from participating in a company-sponsored research project. Compensation will only be provided on the understanding that the provision of compensation

does not amount to an admission of legal liability and is subject to the proposed recipient signing a full and complete release of the company from all claims, damages and costs.

12.4 *Written Informed Consent*

Informed consent will be obtained before the volunteer can participate in the study. The contents and process of obtaining informed consent will be in accordance with all applicable regulatory requirements. Study participation includes all screening procedures, as well as any wash-out of excluded medications.

It is the responsibility of the PI (or medically qualified designee) to obtain a written informed consent from everyone participating in this study after adequate explanation of the aims, methods, objectives, and potential hazards of the study. The PI (or medically qualified designee) must also explain to the volunteers that they are completely free to refuse to enter the study or to withdraw from it at any time. Appropriate forms for documenting a written consent will be provided by the PI or by Arrowhead Pharmaceuticals, Inc.

For this study, each eligible participant will be required to provide written informed consent before participation in the study.

All eligible participants will have the study explained by the PI or designee. They will receive a full explanation, in lay terms, of the aims of the study, the discomforts, risks and benefits in taking part as well as of insurance and other procedures for compensation in case of injury. It will be explained that the study is for research purposes only and is not expected to provide any therapeutic benefit to the individual. It will be pointed out that they can withdraw from the study at any time without prejudice. Each participant will acknowledge receipt of this information by giving written informed consent for participation in the study. The volunteer will be given a copy of the signed Informed Consent Form to retain.

12.5 *Emergency Contact with Principal Investigator*

Suitable arrangements will be made for participants to contact the PI or medically trained designee in the event of an emergency.

12.6 *Notification of General Practitioner*

It is the responsibility of the PI or designee, to notify, where applicable, with the consent of the participant, the general practitioner of the subject's participation in the trial, by sending a letter stating

the nature of the trial, treatments, expected benefits or adverse events and concomitant drugs to be avoided.

12.7 *Clinical Laboratory Certification and Reference Ranges*

Before the initiation of this study, the PI, or designee, will obtain a copy of the certification form, with certification number and expiration date for all clinical laboratories (excluding central laboratories) used in the study. Reference ranges for each clinical laboratory test used in this study will be obtained from the appropriate laboratory, which will perform the test for the study.

12.8 *Protocol Deviations*

A protocol deviation is defined as any intentional or unintentional change to, or noncompliance with, the approved protocol procedures or requirements. The PI will conduct the study in compliance with the approved protocol and will not implement any deviation from or changes to the protocol without prior agreement by the Sponsor and review and documented approval from the EC of an amendment, except where necessary to eliminate an immediate hazard to study subjects.

Deviations may result from the action or inaction of the participant, PI, or site staff. Examples of deviations include, but are not limited to:

- Failure to adhere to study exclusion and inclusion criteria
- Failure to comply with dispensing or dosing requirements
- Use of medications, food, drink, herbal remedies, or supplements that are specifically prohibited in the protocol
- Missed or out-of-window visits
- Drug dosing not administered within the time frame specified in the protocol
- Failure to adhere to test requirements, including vital signs, laboratory tests, physical examinations, PK blood draws, medical history, etc. – either tests not done, incorrect tests done, or not done within the time frame specified in the protocol
- Procedural deviations such as incorrect storage of study drug, failure to update the ICF when new risks become known, failure to obtain EC approvals for the protocol and ICF revisions

Protocol deviations impacting subject safety or eligibility will be reported to the Sponsor or CRO within 2 business days of occurrence and to the EC/competent regulatory authority per local regulatory requirements.

The PI is responsible for ensuring that any known protocol deviations are recorded and reported as agreed.

12.9 *Termination of the Study*

The Sponsor reserves the right to discontinue the trial at any time. Reasons will be provided in the event of this happening. The PI reserves the right to discontinue the study for safety reasons at any time in collaboration with the Sponsor.

13 STUDY ADMINISTRATION

13.1 *Study Monitoring*

Arrowhead Pharmaceuticals, Inc. is responsible for assuring the proper conduct of the study about protocol adherence and validity of the data recorded on the CRFs. Participant confidentiality will be maintained.

In accordance with applicable regulations, cGCP, and Arrowhead Pharmaceuticals, Inc. procedures, Arrowhead Pharmaceuticals, Inc. will be responsible for assigning a study monitor (CRA) who will contact the site to organize a visit prior to participant enrolment to review the protocol and data collection procedures with site staff. In addition, the assigned study monitor will periodically contact the site, including conducting on-site visits. The extent, nature and frequency of on-site visits will be based on such considerations as the study objective and/or endpoints, the purpose of the study, study design complexity, and enrolment rate.

During these site visits, the study monitor will:

- Check the progress of the study.
- Review study data collected.
- Conduct source document verification.
- Identify any issues and address their resolution.
- Check investigational product accountability
- Review blood and urine samples and ensure they are labeled and stored correctly.

This will be done to verify that the:

- Data are authentic, accurate and complete.
- Safety and rights of participants are being protected.
- Study is conducted in accordance with the currently approved protocol (and any amendments), cGCP and all applicable regulatory requirements.

The PI agrees to allow the monitor direct access to all relevant documents and to allocate his/her time and the time of his/her staff to the monitor to discuss findings and any relevant issues.

At study closure, a study monitor will conduct the following activities in conjunction with the PI or site staff as appropriate:

- Return of all study data to Arrowhead Pharmaceuticals, Inc.
- Data queries.
- Accountability, reconciliation and arrangements for unused investigational product(s).
- Inventory and final disposition (e.g., destruction, shipping to repository, etc.).
- Review of site study records for completeness.

Because the study is blinded, an unblinded study monitor will be assigned to visit the site pharmacy during, and at study completion to review the randomization schedule in comparison to the dispensing log to verify correct randomization of study drug.

13.2 *Quality Assurance*

To ensure compliance with cGCP and all applicable regulatory requirements, Arrowhead Pharmaceuticals, Inc. may conduct a quality assurance audit of the study site. Regulatory agencies may also conduct a regulatory inspection of this study. Such audits/inspections can occur at any time during or after completion of the study. If an audit or inspection occurs, the PI and clinical site agree to notify Sponsor as soon as possible following awareness of an impending regulatory inspection. The PI and clinical site agree to allow the auditor/inspector direct access to all relevant documents and allocate his/her time and the time of his/her staff to the auditor/inspector to discuss findings and any relevant issues.

13.3 *Records Retention*

Following closure of the study, the PI must maintain all site study records in a safe and secure location. The records must be maintained to allow easy and timely retrieval, when needed (e.g., audit or

inspection) and whenever feasible, to allow any subsequent review of data in conjunction with assessment of the facility, supporting systems and staff. When permitted by local laws/regulations or institutional policy, some of these records can be maintained in a format other than hard copy (e.g., microfiche, scanned, electronic); however, caution needs to be exercised before such action is taken. The PI must assure that all reproductions are legible and are a true and accurate copy of the original and meet accessibility and retrieval standards, including re-generating a hard copy, if required. Furthermore, the PI must ensure there is an acceptable back-up of these reproductions and that an acceptable quality control process exists for making these reproductions.

Arrowhead Pharmaceuticals, Inc. will inform the PI of the time period for retaining these records to comply with all applicable regulatory requirements. The minimum retention time will meet the strictest standard applicable to that site for the study, as dictated by any institutional requirements or local laws or regulations, or Arrowhead Pharmaceuticals, Inc. standards/procedures; otherwise, the retention period will default to 15 years.

The material to be stored shall include, but is not limited to, the following:

- Signed and dated copy of the final study protocol and any amendments.
- Signed and dated letter of EC approval, letter of constitution of the EC and copies of any other correspondence relevant to the study with the EC or regulatory authorities.
- The EC approved Informed Consent Form.
- Current *curriculum vitae* (signed and dated) of the Principal Investigator and co-workers with major responsibilities in the trial.
- Site Signature and Delegation of Responsibility Log
- FDA Form 1572 (where applicable)
- Financial Disclosure Form(s)
- Blank CRF/eCRF.
- Signed participant informed consent forms.
- Laboratory reference ranges (signed and dated).
- The completed CTN Application Form (where applicable).
- The Final Study Report.

- Clinical raw data including the Source Data Forms, all clinical laboratory report forms, subject CRFs, drug accountability forms, and dispensing records, etc.

14 INFORMATION DISCLOSURE AND INVENTIONS

14.1 [redacted]

[redacted]

[redacted]

[redacted]

14.2 [redacted]

[redacted]

14.3 [redacted]

[redacted]

[redacted]

[redacted]

[redacted]

15 REFERENCES

Chalasani, Naga and Regev, Arie et al. Drug-Induced Liver Injury in Subjects with Preexisting Chronic Liver Disease in Drug Development: How to Identify and Manage? Gastroenterology, 2016, Volume 151, Issue 6, 1046 – 1051

Dewey, F. E., V. Gusarova, R. L. Dunbar, C. O'Dushlaine, C. Schurmann, O. Gottesman, S. McCarthy, C. V. Van Hout, S. Bruse, H. M. Dansky, J. B. Leader, M. F. Murray, M. D. Ritchie, H. L. Kirchner, L. Habegger, A. Lopez, J. Penn, A. Zhao, W. Shao, N. Stahl, A. J. Murphy, S. Hamon, A. Bouzelmat, R. Zhang, B. Shumel, R. Pordy, D. Gipe, G. A. Herman, W. H. H. Sheu, I. T. Lee, K. W. Liang, X. Guo, J. I. Rotter, Y. I. Chen, W. E. Kraus, S. H. Shah, S. Damrauer, A. Small, D. J. Rader, A. B. Wulff, B. G. Nordestgaard, A. Tybjaerg-Hansen, A. M. van den Hoek, H. M. G. Princen, D. H. Ledbetter, D. J. Carey, J. D. Overton, J. G. Reid, W. J. Sasiela, P. Banerjee, A. R. Shuldiner, I. B. Borecki, T. M. Teslovich, G. D. Yancopoulos, S. J. Mellis, J. Gromada, and A. Baras. 2017. 'Genetic and Pharmacologic Inactivation of ANGPTL3 and Cardiovascular Disease', *N Engl J Med*, 377: 211-21.

Gaudet, D., D. A. Gipe, R. Pordy, Z. Ahmad, M. Cuchel, P. K. Shah, K. Y. Chyu, W. J. Sasiela, K. C. Chan, D. Brisson, E. Khoury, P. Banerjee, V. Gusarova, J. Gromada, N. Stahl, G. D. Yancopoulos, and G. K. Hovingh. 2017. 'ANGPTL3 Inhibition in Homozygous Familial Hypercholesterolemia', *N Engl J Med*, 377: 296-97.

Geary, R. S., R. Z. Yu, and A. A. Levin. 2001. 'Pharmacokinetics of phosphorothioate antisense oligodeoxynucleotides', *Curr Opin Investig Drugs*, 2: 562-73.

Graham, M. J., R. G. Lee, T. A. Brandt, L. J. Tai, W. Fu, R. Peralta, R. Yu, E. Hurh, E. Paz, B. W. McEvoy, B. F. Baker, N. C. Pham, A. Digenio, S. G. Hughes, R. S. Geary, J. L. Witztum, R. M. Crooke, and S. Tsimikas. 2017. 'Cardiovascular and Metabolic Effects of ANGPTL3 Antisense Oligonucleotides', *N Engl J Med*, 377: 222-32.

Janas, M. M., C. E. Harbison, V. K. Perry, B. Carito, J. E. Sutherland, A. K. Vaishnaw, N. D. Keirstead, and G. Warner. 2018. 'The Nonclinical Safety Profile of GalNAc-conjugated RNAi Therapeutics in Subacute Studies', *Toxicol Pathol*: 192623318792537.

Marlowe, J. L., V. Akopian, P. Karmali, D. Kornbrust, J. Lockridge, and S. Semple. 2017. 'Recommendations of the Oligonucleotide Safety Working Group's Formulated Oligonucleotide Subcommittee for the Safety Assessment of Formulated Oligonucleotide-Based Therapeutics', *Nucleic Acid Ther*, 27: 183-96.

Minicocci, I., A. Montali, M. R. Robciuc, F. Quagliarini, V. Censi, G. Labbadia, C. Gabiati, G. Pigna, M. L. Sepe, F. Pannozzo, D. Lutjohann, S. Fazio, M. Jauhiainen, C. Ehnholm, and M. Arca. 2012. 'Mutations in the ANGPTL3 gene and familial combined hypolipidemia: a clinical and biochemical characterization', *J Clin Endocrinol Metab*, 97: E1266-75.

Minicocci, I., S. Santini, V. Cantisani, N. Stitziel, S. Kathiresan, J. A. Arroyo, G. Marti, L. Pisciotta, D. Noto, A. B. Cefalu, M. Maranghi, G. Labbadia, G. Pigna, F. Pannozzo, F. Ceci, E. Ciociola, S. Bertolini, S. Calandra, P. Tarugi, M. Averna, and M. Arca. 2013. 'Clinical characteristics and plasma lipids in subjects with familial combined hypolipidemia: a pooled analysis', *J Lipid Res*, 54: 3481-90.

Musunuru, K., J. P. Pirruccello, R. Do, G. M. Peloso, C. Guiducci, C. Sougnez, K. V. Garimella, S. Fisher, J. Abreu, A. J. Barry, T. Fennell, E. Banks, L. Ambrogio, K. Cibulskis, A. Kernytsky, E. Gonzalez, N. Rudzicz, J. C. Engert, M. A. DePristo, M. J. Daly, J. C. Cohen, H. H. Hobbs, D. Altshuler, G. Schonfeld, S. B. Gabriel, P. Yue, and S. Kathiresan. 2010. 'Exome sequencing, ANGPTL3 mutations, and familial combined hypolipidemia', *N Engl J Med*, 363: 2220-7.

Romeo, S., W. Yin, J. Kozlitina, L. A. Pennacchio, E. Boerwinkle, H. H. Hobbs, and J. C. Cohen. 2009. 'Rare loss-of-function mutations in ANGPTL family members contribute to plasma triglyceride levels in humans', *J Clin Invest*, 119: 70-9.

Yu, R. Z., R. S. Geary, J. M. Leeds, T. Watanabe, M. Moore, J. Fitchett, J. Matson, T. Burckin, M. V. Templin, and A. A. Levin. 2001. 'Comparison of pharmacokinetics and tissue disposition of an

antisense phosphorothioate oligonucleotide targeting human Ha-ras mRNA in mouse and monkey', *J Pharm Sci*, 90: 182-93.

Yu, R. Z., J. S. Grundy, S. P. Henry, T. W. Kim, D. A. Norris, J. Burkey, Y. Wang, A. Vick, and R. S. Geary. 2015. 'Predictive dose-based estimation of systemic exposure multiples in mouse and monkey relative to human for antisense oligonucleotides with 2'-o-(2-methoxyethyl) modifications', *Mol Ther Nucleic Acids*, 4: e218.

APPENDIX 1. Guidelings for Study Modification and Discontinuation Rules for Patients with Pre-dose Elevated Transaminases (Chalasani et al., 2016)

Treatment-Emergent ALT	Treatment-Emergent Total Bilirubin(TBL)	Liver Symptoms	Action
Normal baseline:<br>ALT > 5x ULN<br><br>Elevated baseline:<br>ALT > 3x baseline<br>or > 300 U/L<br>(whichever occurs first)	Normal	None	Repeat ALT, AST, ALP, TBL, in 2–3 days<br><br>Follow-up for symptoms.
Normal baseline:<br>ALT > 8x ULN<br><br>Elevated baseline:<br>ALT > 5x baseline<br>or > 500 U/L<br>(whichever occurs first)	Normal	None	Interrupt study drug. Initiate close observation and workup for competing etiologies. (see below)<br><br>Study drug can be restarted only if an alternative etiology is identified and liver enzymes return to baseline.
Normal baseline:<br>ALT > 3x ULN<br><br>Elevated baseline:<br>ALT > 2x baseline<br>or > 200 U/L<br>(whichever occurs first)	TBL > 2x ULN	None	Interrupt study drug. Initiate close observation and workup for competing etiologies.<br><br>Study drug can be restarted only if an alternative etiology is identified and liver enzymes return to baseline.
Normal baseline:<br>ALT > 3x ULN<br><br>Elevated baseline:<br>ALT > 2x baseline<br>or > 200 U/L<br>(whichever occurs first)	Normal or elevated	Symptoms of clinical hepatitis - severe fatigue, nausea, vomiting, right upper quadrant pain	Interrupt study drug. Initiate close observation and workup for competing etiologies.<br><br>Study drug should not be restarted

APPENDIX 2. Genes and Polymorphism Examined in Consented Subjects

Cohorts 7, 7b, 7c: Genes/Polymorphisms examined in Familial Hypercholesterolemia Panel (Phosphorus Diagnostics)

Gene ID	Full Gene Sequence or SNP	SNP ID (If applicable)
APOB	Full Gene	NA
LDLR	Full Gene	NA
LDLRAP1	Full Gene	NA
PCSK9	Full Gene	NA
ABCB1	SNP	rs2032582
ABCG2	SNP	rs2231142
APOE	SNP	rs7412
KIF6	SNP	rs20455

Cohorts 1-4, 2b-4b, 5, 6 and 8: Genes examined in Pan Dyslipidemia Panel (Phosphorus Diagnostics)

Gene ID	
ABCA1	GPD1
ABCG5	GPIHBP1
ABCG8	LCAT
ANGPTL3	LDLR
APOA1	LDLRAP1
APOA5	LIPA
APOB	LIPC
APOC2	LMF1
APOC3	LPL
CYP27A1	PCSK9
SAR1B	

APPENDIX 3. Local Injection Site Reactions (LISRs)

The following MedDRA Preferred Terms determined by the Sponsor's pharmacovigilance personnel represent the local injection site reaction:

Injection site discomfort	Injection site abscess
Injection site discoloration	Injection site abscess sterile
Injection site erythema	Injection site atrophy
Injection site irritation	Injection site calcification
Injection site inflammation	Injection site cellulitis
Injection site induration	Injection site dermatitis
Injection site pain	Injection site erosion
Injection site oedema	Injection site fibrosis
Injection site pruritus	Injection site indentation
Injection site rash	Injection site necrosis
Injection site urticaria	Injection site nodule
Injection site reaction	Injection site ulcer
Injection site swelling	

LISRs will only include events that start on the day of injection and persist for at least 48 hours post injection (i.e., event onset date on the day of injection and resolution date not on the day of injection or the day after the injection) will be included. Events with onset date on the day of injection and missing resolution date will also be included in the summary.

The following calculation will be utilized to determine the percentage of injections leading to local injection site reactions:

(A/B)*, where A = number of injections with a local injection site reactions, and B = total number of injections.

NOVOTECH
The Asia Pacific CRO

STATISTICAL ANALYSIS PLAN

A PHASE 1 SINGLE AND MULTIPLE DOSE STUDY TO EVALUATE THE SAFETY, TOLERABILITY, PHARMACOKINETICS AND PHARMACODYNAMIC EFFECTS OF ARO-ANG3 IN ADULT HEALTHY VOLUNTEERS AND IN DYSLIPIDEMIC PATIENTS

Protocol No.: AROANG1001

Product Code: ARO-ANG3

**PREPARED FOR:**	Arrowhead Pharmaceuticals, Inc.<br>177 East Colorado Boulevard, Suite 700<br>Pasadena, CA 91105<br>USA
**PREPARED BY:**	Novotech (Australia) Pty Ltd<br>Level 3, 235 Pyrmont Street<br>Pyrmont, NSW, 2009<br>Australia
**DATE OF ISSUE:**	14 July 2021
**VERSION/STATUS:**	Version 9.0 (14 July 2021)<br>Version 8.0 (14 December 2020)<br>Version 7.0 (30 September 2020)<br>Version 6.0 (01 September 2020<br>Version 5.0 (11 August 2020)<br>Version 4.0 (20 July 2020)<br>Version 3.0 (16 June 2020)<br>Version 2.0 (26 May 2020)<br>Version 1.0 (17 March 2020)
**VERSION DATE:**	14 July 2021
**AUTHOR:**	Alan Herschtal (V9.0)

SAP APPROVAL

By my signature, I confirm that this SAP has been reviewed by Novotech Inc., and has been approved for use on the AROANG1001 study:

Name	Title / Company	Signature	Date
Alan Herschtal	Principal Statistician, Biometrics, / Novotech	[redacted]	16-Jul-2021

By my signature, I confirm that this SAP has been reviewed by Arrowhead Pharmaceuticals Inc., and has been approved for use on the AROANG1001 study:

Name	Title / Company	Signature	Date
Mei Dai	Associate Director, Clinical Pharmacology / Arrowhead	*mei dai* Electronically signed by: mei dai Reason: I have reviewed Date: Jul 16, 2021 14:42 EDT	16-Jul-2021
Iris Chen	Associate Director, Biostatistics / Arrowhead	*Iris Chen* Electronically signed by: Iris Chen Reason: I approve Date: Jul 16, 2021 10:38 PDT	16-Jul-2021
Armando Lira Pineda	Sr. Director, Global Clinical Development / Arrowhead	*Armando Lira Pineda* Electronically signed by: Armando Lira Pineda Reason: I approve Date: Jul 16, 2021 [illegible]:31 PDT	16-Jul-2021

Table of contents

1. INTRODUCTION *5*
2. PROJECT OVERVIEW *6*
2.1 Study Design 6
2.2 Objectives 9
2.3 Study Endpoints 10
2.4 Sample Size 12
2.5 Randomization 12
3. STATISTICAL CONSIDERATIONS *13*
3.1 Adverse event imputation rules 14
3.2 Concomitant medication imputation rules 14
3.3 Procedure date imputation rules 14
4. ANALYSIS POPULATIONS *15*
5. PARTICIPANT DISPOSITION *16*
6. PROTOCOL DEVIATIONS *17*
7. DEMOGRAPHIC AND BASELINE INFORMATION *18*
7.1 Demographics 18
7.2 Medical history 18
7.3 Informed Consent and Eligibility 18
7.4 Pregnancy Test (Urine) 18
7.5 Serology Screen (Hepatitis/HIV) 18
7.6 Urine Drug Screen 18
7.7 Alcohol Breath Test 18
8. TREATMENT EXPOSURE *19*
9. PHARMACOKINETICS (PK) *20*
9.1 Pharmacokinetic Parameters 20
9.2 Biostatistical methods 21
10. SAFETY *23*
10.1 Adverse Events 23
10.2 Adverse Events (AEs) at the Injection Site and Local Injection Site Reactions (LISR) 24
10.3 Concomitant medication 25
10.4 Laboratory (excluding PK/PD) 25
10.5 Vital Signs 28
10.6 Physical examination 28
10.7 12-lead ECG 29

10.8 Pharmacodynamics 30
11. IMMUNOGENICITY *33*
12. HANDLING OF MISSING DATA *34*
13. CHANGES TO THE PLANNED ANALYSIS *35*
14. INTERIM AND FINAL ANALYSIS *36*
14.1 Dose Escalation, Data Safety Committee (DSC) Analyses 36
14.2 Interim Analyses 36
14.3 Final Analysis (End of Study) 37
15. SOFTWARE *37*
16. TABLES *38*
17. LISTINGS *52*
18. FIGURES *58*
19. APPENDIX A NONCOMPARTMENTAL PHARMACOKINETIC ANALYSIS *61*
19.1 Handling Missing or Non-Quantifiable Data 61
19.2 Pharmacokinetic Parameter Calculation 61
19.3 Treatment of Outliers in Pharmacokinetic Analysis 62
20. APPENDIX B EARLY TERMINATION ANALYSIS WINDOWS *63*
21. REFERENCES *64*

1. INTRODUCTION

The following Statistical Analysis Plan (SAP) provides the outline for the statistical analysis of the data from AROANG1001 study.

This Statistical Analysis Plan (SAP) is an adjunct to the Arrowhead Pharmaceuticals protocol number AROANG1001 (Version 7.1, 15 Jan 2020). The planned analyses identified in this SAP may be included in clinical study reports (CSRs), regulatory submissions, or future manuscripts. Also, post hoc analyses not necessarily identified in this SAP may be performed to further examine study data. Any post hoc, or unplanned, exploratory analyses performed will be clearly identified as such in the final CSR.

2. PROJECT OVERVIEW

2.1 Study Design

This is a phase 1 single and multiple dose-escalating study to evaluate the safety, tolerability, Pharmacokinetics and Pharmacodynamic Effects of ARO-ANG3 in Adult Healthy Volunteers and in Dyslipidemic Patients.

ARO-ANG3 is a drug candidate developed by Arrowhead Pharmaceuticals, Inc. to treat dyslipidemia. ARO-ANG3 is administered through subcutaneous injection.

This study will include the following cohorts, with the dose escalation schedule plotted as below. Dose escalation and start of new cohort are subject to Data Safety Committee (DSC) approval based on the evaluation of all available safety data.

- Cohorts 1, 2, 3 and 4: Healthy Volunteers (HV) with fasting TG>100mg/dL and fasting LDL-C >70mg/dL.

  Each of cohorts 1 – 4 cohort will recruit 10 healthy volunteers, with subjects randomized to receive placebo (4 subjects) or active ARO-ANG3 (6 subjects) in a double blinded fashion. The first two subjects in each cohort serve as sentinel subjects (one ARO-ANG3 and one placebo).
  Cohorts 1 – 4 will be single dosed as follows:
  - Cohort 1: 35 mg ARO-ANG3 or PBO, at Day 1
  - Cohort 2: 100 mg ARO-ANG3 or PBO, at Day 1
  - Cohort 3: 200 mg ARO-ANG3 or PBO, at Day 1
  - Cohort 4: 300 mg ARO-ANG3 or PBO, at Day 1
- Cohorts 2b, 3b and 4b: HV's.

  Each of cohorts 2b – 4b will recruit four (4) healthy volunteers in an open label fashion with all cohorts planned to receive multiple escalating doses of ARO-ANG3 at escalating dose levels of 100, 200, and 300 mg.
  Cohorts 2b – 4b will be multiple dosed as follows:
  - Cohort 2b: 100 mg ARO-ANG3, at Days 1 and 29
  - Cohort 3b: 200 mg ARO-ANG3, at Days 1 and 29
  - Cohort 4b: 300 mg ARO-ANG3, at Days 1 and 29
- Cohorts 5 with liver fat fraction of ≥ 10%:

  Cohort 5 will recruit 9 subjects (6 active and 3 PBO) in a double blinded fashion who have a liver fat fraction of ≥10% based on MRI-PDFF conducted at Screening.

- Cohorts 6: Patients on a stable statin regimen with LDL-C >70 mg/dL

  Cohort 6 will recruit 9 subjects (6 active and 3 PBO) in a double blinded fashion who are on a stable drug treatment regimen for elevated LDL-C including a statin for at least 6 months and with fasting Screening LDL-C > 70 mg/dL. All patients in cohort 6 will receive 200mg ARO-ANG3 or PBO, at Days 1 and 29

- 7, 7b and 7c: familial hypercholesterolemia.

  Cohort 7, 7b and 7c will each recruit up to 6 patients with a diagnosis of heterozygous or

homozygous familial hypercholesterolemia, defined as a documented positive genetic test OR Dutch Lipid Clinic Network Score ≥ 6 for NZ participants or ≥ 8 for all other participants, with LDL-C > 100 mg/dL (2.59 mmol/L) despite standard of care therapy OR with LDL-C > 70 mg/dL (1.81 mmol/L) while on a PCSK-9 inhibitor OR with LDL-C > 70 mg/dL (1.81 mmol/L) in the presence of documented atherosclerotic cardiovascular disease.
Cohorts 7, 7b and 7c will be multiple dosed as follows:
  - Cohort 7: 200 mg ARO-ANG3, at Days 1 and 29
  - Cohort 7b: 100 mg ARO-ANG3, at Days 1 and 29
  - Cohort 7c: 300 mg ARO-ANG3, at Days 1 and 29

- Cohort 8: TGs ≥ 300 mg/dL (3.39 mmol/L)

  Cohort 8 is open-label with up to 6 patients with fasting serum triglycerides of at least 300 mg/dL (3.39 mmol/L). All subjects to receive 200 mg of ARO-ANG3 on Days 1, 29

- Cohort 9: an open-label extension (continued access) cohort into which patients from Cohorts 7, 7b and 7c may elect to enroll after completing the scheduled regimen of treatment under their original cohort.
  - 200 mg ARO-ANG3, at Days 113, 197, 281 and 365

Study part will be used in this SAP to describe the following:

- Healthy Volunteers (SAD)
  - Cohorts: 1, 2 ,3 and 4
- Healthy Volunteers (MAD)
  - Cohorts: 2b ,3b and 4b
- Disease Cohorts (MAD)
  - Cohort 5, 6, 7, 7b, 7c, 8 and 9

Dose Escalation Schedule

Single Dose Healthy Volunteers (double blind in cohorts 1, 2, 3, 4)			Multi-dose Patients (double blinded in cohorts 5, 6, open label in 2b, 3b, 4b, 7, 7b, 7c, 8)
Cohort*	Dose (Day 1)	Day 8 safety evaluation	Dose Regimen
Cohort 1**	35 mg		NA
Cohort 2**	100 mg		NA
Cohort 3**	200 mg		NA
Cohort 4**	300 mg		NA
			Cohort 5***: 200 mg or PBO dosed on Day 1, 29
			Cohort 6***:200 mg or PBO dosed on Day 1, 29
			Cohort 8***: 200 mg dosed on Day 1, 29
			Cohort 2b****: 100 mg dosed on Day 1, 29
			Cohort 3b****: 200 mg dosed on Day 1, 29
			Cohort 4b****: 300 mg dosed on Day 1, 29
			Cohort 7***: 200 mg dosed on Day 1, 29
			Cohort 7b****: 100 mg dosed on Day 1, 29
			Cohort 7c****: 300 mg dosed on Day 1, 29
			Cohort 9*****: 200 mg dosed on Days 113, 197, 281 and 365

* Cohorts 1, 2, 3 and 4 will use 2 sentinel subjects

**Dose escalation to the next highest dose level or to multiple dosing will occur after cumulative safety data through Day 8 for Cohorts 1, 2, 3, and 4 have been evaluated by the DSC

***Screening and enrolment into Cohorts 5, 6, 7 and 8 may not occur until an amended protocol justifying the dose to be used in these cohorts has been approved by the EC.

****No DSC vote is required to open these cohorts. These cohorts may enroll in parallel

***** Patients from Cohorts 7, 7b and 7c may elect to continue to receive up to four 200 mg doses administered approximately every 12 weeks. No DSC vote is required for patient to roll over into Cohort 9.

Participants who are withdrawn or discontinue prior to EOS for reasons other than an adverse event, may be replaced at Sponsor's discretion.

Cohorts 1 through 4 will enroll sequentially. Cohorts 5, 6, 7 and 8 may be opened by the DSC after review of cumulative safety data through Day 8 of Cohort 4 and only after approval by the HREC of an amended protocol updating safety information from Cohorts 1 through 4 which will include a rationale for the dose level to be used in Cohorts 5, 6, 7and 8 (See Section 4.8). These multi-dose patient cohorts may enroll in parallel after they are opened for enrollment by the Data Safety Committee (DSC) and after the amended protocol has been approved by the HREC. Screening for Cohorts 5, 6, 7 and 8 may begin after the described amended protocol has been approved by the EC.

Pharmacokinetic (PK) intensive sampling will be conducted for cohorts 1, 2, 3, 4, 2b, 3b and 4b on Day 1 (dosing day) at the time of 0 (pre-dose), 15 minutes, 0.5, 1, 2, 3, 6, 24 and 48 hours post-dose.

Additionally, PK intensive sampling will be conducted for cohorts 2b, 3b and 4b at the same time points relative to the Day 29 dose.

2.2 Objectives

2.2.1 Primary objective

To determine the incidence and frequency of adverse events possibly or probably related to treatment as a measure of the safety and tolerability of ARO-ANG3 using escalating single and multiple doses in healthy volunteers and multiple doses in dyslipidemic patients.

2.2.2 Secondary objectives

- To evaluate the single-dose and multi-dose pharmacokinetics of ARO-ANG3 in healthy volunteers.
- To determine the reduction in fasting serum ANGPTL3 from baseline in response to single and multiple doses of ARO-ANG3 as a measure of drug activity in healthy volunteers and in response to multiple doses of ARO-ANG3 in dyslipidemic patients (all values drawn after at least 8 hour fast).

2.2.3 Exploratory objectives

- To evaluate the effect of single or multiple doses of ARO-ANG3 on change from baseline in:
    - Fasting LDL-C
    - Total Cholesterol
    - non-HDL-C
    - HDL-C
    - VLDL-C
    - Triglycerides
    - Lp(a)
    - Total ApoB
    - apoB-48
    - apoB-100
    - apoC-III
    - apoC-II
    - apoA-V
    - lipoprotein lipase mass (if feasible)
    - CETP mass (if feasible)
    - apoA-I (all values drawn after at least 8 hour fast)
    - .
- To evaluate the effect of single or multiple doses of ARO-ANG3 on changes from baseline in BMI.
- To evaluate the effect of single or multiple doses of ARO-ANG3 on changes from baseline in fasting serum blood glucose, C-peptide, hemoglobin A1C, GTT and fasting serum insulin.

- To evaluate the effect of multiple doses of ARO-ANG3 on change from baseline liver fat content using Magnetic Resonance Imaging (using MRI-PDFF, Cohort 5 only).
- To evaluate the effect of multiple doses of ARO-ANG3 on change from baseline in post-prandial (post standardized high fat/high carbohydrate meal) serum TGs in specified cohorts.
- To evaluate excretion of ARO-ANG3 (full length and metabolites) and identify metabolites in plasma and urine in the multi-dose healthy volunteer cohorts (2b, 3b and 4b).

2.3 Study Endpoints

2.3.1 Primary Endpoints

- AEs/SAEs –
  - System Organ Class and Preferred Term;
  - Grade (mild, moderate or severe)
  - Causality (relationship to study treatment: Not Related, Possibly Related or Probably Related)
- Physical examinations –
  - Height
  - Weight
  - BMI
- Vital signs (Systolic/diastolic blood pressure, temperature, heart rate, respiratory rate)
- ECG measurements (using single 12-lead ECG)
- Injection Site Reactions (Mild, Moderate or Severe)
- Clinical laboratory tests (Biochemistry (including hemoglobin A1C), Hematology, Coagulation, Urinalysis, Microscopic urinalysis (if indicated), Serology, FSH, Drug and Alcohol use, Pregnancy, Lipid Parameters, Serum insulin levels, Serum glucose levels, Stool occult blood test)
- Concomitant medications/therapy, and
- Reasons for treatment discontinuation due to toxicity

2.3.2 Secondary Endpoints

Plasma Pharmacokinetics (Cohorts 1, 2, 3, 4, 2b, 3b and 4b)

- Blood samples will be collected for cohorts 1, 2, 3 and 4 at the time points of 0 (pre-dose), 15 min, 0.5, 1, 2, 3, 6, 9, 12, 18, 24 & 48 hours post-dose on Day 1. Blood samples will also be collected for cohorts 2b, 3b and 4b at the same time points relative to each of the dosing days (Days 1 and 29).

Urine pharmacokinetics (Cohorts 2b, 3b and 4b)

- Urine will be collected cumulatively from 0-6 hours and 6-24 hours post-dose for both doses (Day 1 and 29). In addition, spot collection will be performed on Days 1 (pre-dose), 8, 15, 22 and 29 (pre-dose). Urine creatinine will be measured on all urine samples (interval and spot collections), Metabolite ID will be performed on urine samples collected (pooled analysis).

Pharmacodynamics

- fasting serum ANGPTL3

2.3.3 Exploratory endpoints

Pharmacodynamics

- fasting LDL-C
- Total Cholesterol
- Fasting non-HDL-C
- Fasting HDL-C
- Fasting VLDL-C
- Fasting Triglycerides
- Fasting Lp(a)
- Fasting Total ApoB
- Fasting apoB-48
- Fasting apoB-100
- Fasting apoC-III
- Fasting apoC-II
- Fasting apoA-V
- lipoprotein lipase mass (if feasible)
- CETP mass (if feasible)
- Fasting apoA-I
- Hemoglobin A1c
- C-peptide
- fasting serum blood glucose
- C-peptide
- hemoglobin A1C
- GTT
- fasting serum insulin
- post-prandial Triglyceride (TG) Test
- liver fat content using Magnetic Resonance Imaging

Physical Exam

- BMI

2.4 Sample Size

2.5 This study represents a proof of principle study, and as such no formal sample size calculation was performed. Randomization

Eligible subjects in double-blind cohorts (cohorts 1, 2, 3, 4, 5 and 6) will be allocated a unique randomization number, in accordance with the randomization schedule. In each cohort, the first two subjects (sentinels) will be randomized separately to one active (ARO-ANG3) and one PBO. Each participant will be assigned to either active or PBO treatment. The allocation of active treatment or PBO will be performed using a block randomization algorithm.

All other cohorts (2b, 3b, 4b, 7, 7b, 7c, 8 and 9) are open label and participants in these cohorts will not be randomized.

3. STATISTICAL CONSIDERATIONS

Data will be handled and processed according to the sponsor's representative (Novotech (Australia) Pty Ltd) Standard Operating Procedures (SOPs), which are written based on the principles of GCP.

All data collected on the eCRFs will be presented in the data listings and will be listed and sorted by participant number and visit, where applicable. All dates in listings will be presented in YYYY-MM-DD format. Summaries will be presented for the active drug patients in each cohort considered separately and for placebo patients pooled across cohorts, as well as overall (all active drug and placebo patients combined), as applicable.

Unless otherwise stated, the following basic descriptive statistics will be provided:

- Continuous variables: the number of non-missing values (N), mean, standard deviation (SD), median, minimum, maximum. For PK variables, the coefficient of variance (CV) the geometric mean, the geometric CV, and the number below the minimum level of quantification (n BLQ) will also be provided.
- Categorical variables: frequency counts and percentages per category. Percentages will be rounded to one decimal place, with the denominator being the number of subjects in the relevant population with non-missing data, unless otherwise specified.

Baseline values will be defined as the last non-missing observation (pre-dose value closest to the first dose) for each participant prior to the dosing of study medication (i.e. start of injection on Day 1).

Medpace Research Labs (MRL) will be used as the preferred data source for calculations/analysis for the following PD markers: ANGPTL3, fasting LDL-C, Total Cholesterol, non-HDL-C, HDL-C, VLDL-C, Triglycerides, total apoB, apoB-48, apoB-100, apoC-III, apoA-I, apoC-II, apoA-V, lipoprotein lipase mass (if feasible), CETP mass (if feasible), Hemoglobin A1c and C-peptide.

If there is a repeat assessment, the repeat result would be used in the analysis table and the original result will only appear in listings. All repeat assessments captured in the Electronic Data Capture (EDC) system will be presented in the data listings.

Generally, no missing data will be imputed.

For continuous valued PD variables that have samples reported as BLOQ (post-baseline/day 1 visit), a nominal value of ½ LLOQ will be used for calculations. For samples reported as above the upper limit of quantitation, a nominal value equal to ULOQ will be used in calculations.

Continuously valued safety variables will be reported to the same precision as the source data. Derived variables will be reported using the same precision as the value(s) from which they were derived. For the reporting of descriptive statistics, the mean, median and SD will be reported to 1 decimal place more than the source data; the minimum and the maximum values will be presented to the same precision as the source data. Rounding is not allowed in the middle of calculations. It only takes place at the last step to report the final result. Post-dose time points/visits will be calculated relative to start time of injection on Day 1. Study day will be defined as the assessment date minus the dosing date +1.

Baseline demographic statistics will be tabulated (for categorical variables) and summarized (for continuously valued variables) by cohort for active drug patients, for placebo patients pooled and for all patients (active drug and placebo) considered collectively. Eligibility assessments at

baseline, including medical/surgical history data and physical examination data (including height and weight), will be listed for each participant.

3.1 Adverse event imputation rules

Adverse events will be flagged as treatment emergent using valid answers to the questions "For events that occurred on Day 1, did the event occur before start of study drug administration?" or "Did the event start before first dose of investigational product?" on the eCRF regardless of whether or not the AE onset date is complete. Adverse events that cannot be definitely determined as occurring prior to study drug administration will be counted as treatment emergent adverse events unless either the partial start date/time or a partial or complete end date/time documents the AE as occurring prior to treatment.

TEAE Start date:

- TEAE imputed dates will not be earlier than the participant's Day 1 date.
- If all year, month, and day are missing then use the participant's Day 1 date.
- If year is available but day and month are missing, the day and month for the start date will be set to the 1st of January of the onset year.
- If year and month are available but day is missing, the day will be set to the 1st of the month of the onset year.

End date will not be imputed.

3.2 Concomitant medication imputation rules

Medications with missing or partial end dates will be assumed to be concomitant unless a partial end date documents the Concomitant Medication as ending prior to treatment.

Concomitant medication start date:

- If all year, month, and day are missing then use the participant's Day 1 date.
- If year is available but day and month are missing, the day and month for the start date will be set to the 1st of January of the onset year.
- If year and month are available but day is missing, the day will be set to the 1st of the month of the onset year.

3.3 Procedure date imputation rules

Procedures with missing or partial end dates will be counted as concomitant unless a partial end date documents the procedure as ending prior to the participant's Day 1 date.

4. ANALYSIS POPULATIONS

In this study, three analysis populations are defined: Safety Population, Pharmacokinetic Population, and Pharmacodynamic population. Data for Screen Failures will not be included in any summary tables, figures, or data listings. Subjects to be included in the defined (various) analysis populations will be decided upon at the blinded data review meeting prior to unblinding of double blinded cohorts. Unblinding of healthy volunteer cohorts may occur at Sponsor discretion on a cohort by cohort basis after all subjects in a cohort have completed their last planned on-site study visit (Day 113 End of Study). Study participants as well as sites will remain blinded. The same process will be applied to the open label cohorts even though there is no blinding for these cohorts.

Furthermore, any additional analysis populations not identified in the SAP will be identified in the final CSR as post hoc analyses. This may include additional study populations or subgroups of interest.

The number and percentage of subjects in each analysis population will be summarized.

Safety Population

All subjects who receive at least one dose of study treatment (ARO-ANG3 or PBO) will be included in the Safety Population. Subjects will be summarized according to the treatment they received.

All safety, treatment exposure, demographic and baseline characteristic data will be listed and summarized using the Safety Population.

Pharmacokinetic (PK) Population

Subjects who have received the active treatment (ARO-ANG3) and have adequate PK data to characterize PK profile will be included in the PK Population.

Subjects who prematurely discontinue from the study will be included in the Safety Population but may not be included in the PK Population if they do not have PK data to contribute to the analyses. Subjects with missing sample concentrations will be included in the PK analyses provided their PK parameters can be adequately characterized based upon the remaining data. Subjects who received placebo will be excluded from the PK population. The PK population will be used for the summaries of all PK data.

Subjects with protocol violations will be assessed on a participant-by-participant basis for inclusion in the PK Population.

Pharmacodynamic (PD) Population

Subjects who received at least one dose of study treatment (ARO-ANG3 or placebo) and had PD assessment from baseline and ≥1 assessment from post-baseline will be included in the PD population. Subjects will be summarized according to the treatment they received.

Subjects with protocol violations will be assessed on a participant-by-participant basis for inclusion in the PD Population. The determination of study populations will be made, prior to unblinding the PD data, at the blinded data review meeting before the final analyses. The PD population will be used for the summaries of all pharmacodynamic endpoints.

5. PARTICIPANT DISPOSITION

All subjects who provide informed consent and are randomized (Cohorts 1, 2, 3, 4, 5 and 6) or enrolled (Cohorts 2b, 3b, 4b, 7, 7b, 7c, 8 and 9) will be accounted for in this study. Participant disposition will be summarized using the Safety Population.

By-participant data listings for participant disposition will be generated, including informed consent date, randomization date/number (double blinded subjects), completion status, date of withdrawal and reason for withdrawal from the study, or date of screen failure and reason for screen failure if applicable.

The number of subjects randomized / enrolled, as well as the number and percentage of subjects completing the study and withdrawn from the study will be presented by study part and treatment group (dose level), for placebo patients pooled and overall. The reason for withdrawal will also be summarized for all subjects who do not complete the study.

6. PROTOCOL DEVIATIONS

Any protocol deviations will be presented for each participant in the by-participant data listings. Frequent deviations will be listed individually and rare ones will be combined as other or important deviations will be listed separately.

Prior to database lock, all protocol deviations will be reviewed by medical monitors and assigned a category (see below).

A protocol deviation is defined as any intentional or unintentional change to, or noncompliance with, the approved protocol procedures or requirements. Deviations may result from the action or inaction of the patient, investigator, or site staff. All deviations will be tracked and should be reported to IRBs in accordance with their reporting policy. Examples of deviations include, but are not limited to:

- Failure to adhere to study exclusion and inclusion criteria;
- Failure to comply with dispensing or dosing requirements;
- Use of medications, food, drink, herbal remedies, or supplements that are specifically prohibited in the protocol;
- Missed or out-of-window visits;
- Drug dosing not administered within the time frame specified in the protocol;
- Failure to adhere to test requirements, including vital signs, laboratory tests, physical examinations, blood draws, medical history, etc. – either tests not done, incorrect tests done, or not done within the time frame specified in the protocol;
- Procedural deviations such as incorrect storage of study drug, failure to update the ICF when new risks become known, failure to obtain IRB/EC approvals for the protocol and ICF revisions.

7. DEMOGRAPHIC AND BASELINE INFORMATION

Demographic and baseline body measurements will be summarized using the Safety Population.

7.1 Demographics

Demographic data, including age, gender, race, and physical examination data (weight, height and BMI), will be summarized by study part and treatment group (dose level) for active drug patients, for placebo patients pooled and overall. A by-participant data listing for demographic characteristics will be generated by cohort.

Baseline disease characteristics including serum ANGPTL3, fasting LDL-C, Total Cholesterol, non-HDL-C, HDL-C, VLDL-C, Triglycerides, Lp(a), total apoB, apoB-48, apoB-100, apoC-III Hemoglobin A1c, C-peptide and apoA-I will be summarized by study part and treatment group (dose level) for active drug patients, for placebo patients pooled and overall. A by-participant data listing for these characteristics will be generated.

7.2 Medical history

Past medical history will be coded using the Medical Dictionary for Regulatory Activities, MedDRA® with the latest available version. Medical history data, including the MedDRA codes, will be presented in the by-participant data listings.

7.3 Informed Consent and Eligibility

Informed consent date, and inclusion/exclusion eligibility criteria information, including any criteria not met, will be listed for each participant.

7.4 Pregnancy Test (Urine)

Child-bearing potential (yes/no), and if No, the reason (post menopause, surgically sterile, and other), and pregnancy test results will be included in the by-participant data listings. This includes the urine dipstick pregnancy test results assessed regularly at the scheduled study visits. Screening Follicle-Stimulating Hormone (FSH) will also be listed.

7.5 Serology Screen (Hepatitis/HIV)

Data for Hepatitis B, Hepatitis C and HIV assessment at screening will be listed for each participant.

7.6 Urine Drug Screen

Urine Drug Screen results at Screening will be listed for each participant.

7.7 Alcohol Breath Test

Alcohol Breath Test results at Screening will be listed for each participant to test for alcohol consumption.

8. TREATMENT EXPOSURE

Study drug administration results will be presented using the Safety Population. Study drugs include ARO-ANG3 and placebo.

Each single dose of either active drug (ARO-ANG3) or PBO (normal saline 0.9%), will be administered by subcutaneous injection. Injections will be made into the subcutaneous tissue at an appropriate site (e.g. abdomen, thigh, upper arm, etc.). The abdomen is the preferred site. Injection site is to be varied (no multiple injections into the same exact site. Alternating various locations on the abdomen is acceptable). Injection site location is to be recorded in the eCRF.

A by-participant data listing will be generated for study drug (ARO-ANG3 or Placebo) administrations. This listing will include study drug administration date, time, dose, and injection site. If study drug was not administered, the reason why the drug was not administered will be reported.

9. PHARMACOKINETICS (PK)

Pharmacokinetic Assessment:

As noted above, plasma PK samples are collected for cohorts 1, 2, 3, 4, 2b, 3b and 4b on Day 1 (dosing day) at the time of 0 (pre-dose), 15 minutes, 0.5, 1, 2, 3, 6, 9,12,18, 24 and 48 hours post-dose, and additionally for cohorts 2b, 3b and 4b at the same time points relative to the Day 29 dose.

Urine PK samples will be collected for cohorts 2b, 3b and 4b from 0-6 hours and from 6-24 hours post-dose for both doses (Days 1 and 29). Spot urine collection will take place on Days 1 (pre-dose), 8, 15, 22 and 29 (pre-dose).

9.1 Pharmacokinetic Parameters

Pharmacokinetic parameters for ARO-ANG3 will be calculated from the plasma concentration-time and urine excretion data using noncompartmental methods (Phoenix™ WinNonlin®, Version 8.1.0 or later, Princeton, NJ.) and actual sample collection times. The following Plasma and urine PK parameters for ARO-ANG3 will be reported on Day 1 for subjects in cohorts 1, 2, 3, 4, 2b, 3b and 4b, as well as on Day 29 for subjects in cohorts 2b, 3b and 4b, whenever data applicable:

Variable	Definition
Plasma PK parameters on Day 1 and Day 29	
AUC_{last}	The area under the plasma concentration-time curve, from time 0 (time of dosing) to the last time point with measurable analyte concentration.
AUC_{0-24}	Area under the plasma concentration-time curve, from time 0 to the 24-hour time point.
AUC_{inf}	Area under the plasma concentration-time curve extrapolated to infinite time
$AUC_{\%extrap}$	The extrapolated portion of AUC_{inf} calculated as: $AUC_{\%extrap} = 100 \times \frac{C_{last}/\lambda_z}{AUC_{inf}}$
C_{max}	Maximum observed PK concentration.
t_{max}	Time to reach C_{max}. If the maximum observed concentration value occurs at more than 1 time point, t_{max} is defined as the first time point with this value.
$t_{½}$	Terminal elimination half-life computed as: $t_{½} = \frac{\ln(2)}{\lambda_z}$ Where ln(2) is the natural logarithm of 2.
CL/F	Apparent total clearance of the drug from plasma calculated as: $CL/F = \frac{Dose}{AUC_{inf}}$
V_z/F	Apparent volume of distribution during the elimination phase calculated as: $V_z/F = \frac{Dose}{\lambda_z \cdot AUC_{inf}}$

Urine PK Parameters on Day 1 and Day 29	
$Ae_{0\text{-}24}$	Amount of unchanged drug excreted in the urine 0-24 hours postdose
$Fe_{0\text{-}24}$	Fraction excreted (or equivalently the percent of dose excreted) in the urine, calculated by 100 • (Ae, 0-24 h/ Dose).
CL_R	Renal clearance calculated as: $$CL_R = \frac{Ae_{0-24}}{AUC_{0-24}}$$

Additional PK parameters such as dose normalized C_{max} and AUCs, or BW normalized PK parameters may be estimated as appropriate.

The Sponsor will provide NCA analysis of plasma and urine PK data, details about the PK parameters calculation in Appendix A.

9.1.1 Non-Quantifiable Concentrations

All concentration values reported as no results (not collected or not determined) values will be treated as missing. For the calculation of concentration summaries, all concentrations below the quantifiable limit (BLQ) will be treated as 0, however, for estimating geometric mean and geometric CV% they will be treated as missing.

9.2 Biostatistical methods

9.2.1 Listings and Descriptive Summary Statistics – Plasma PK

ARO-ANG3 plasma concentrations will be listed by cohort, participant, dosing day (for cohorts 2b, 3b and 4b) and collection time. ARO-ANG3 concentrations will be summarized descriptively by study part, treatment group (dose level), dosing day and nominal sampling time for the PK Analysis Population, including n, arithmetic mean, SD, minimum, median, maximum, coefficient of variation [CV(%)], geometric mean (GM), geometric SD, geometric CV%.

The actual blood sampling dates and times relative to dosing time will be listed by study cohort, participant and nominal sampling time, with time deviation calculated, for all subjects with available plasma concentration data, including subjects receiving placebo or excluded from the PK Analysis Population.

Individual plasma concentration of ARO-ANG3 will be plotted on a linear and semi-log scales versus nominal time for each part/treatment. For each cohort, the spaghetti plots of individual plasma ARO-ANG3 on a linear and semi-log scales will also be presented.

Mean plasma PK concentration vs. nominal time will be plotted on both linear and semi-log scales for ARO-ANG3 by part and treatment. The mean concentration vs. nominal time may also be plotted by dose level across different populations if no significant difference between populations.

Plasma PK parameters for ARO-ANG3 will be listed by cohort and summarized by study part and treatment group (dose level) descriptively, including n, arithmetic mean, SD, minimum, median, maximum, coefficient of variation [CV(%)], geometric mean (GM), geometric SD, geometric CV%; For t_{max}, only n, minimum, median, and maximum will be reported. Diagnostic PK parameters listed in appendix A will be listed only. PK parameters may also be summarized as appropriate by dose level which included different populations.

Geometric CV% = 100*(exp(SD^2)-1)^0.5, where SD is the standard deviation of the log-transformed data.

Actual sampling times that are outside the sampling window (± 2 minutes for <6 hour timepoints; ± 5 minutes for the 8 - 48 hour timepoints;) will be listed and used for PK parameters calculation but will be excluded from summary of PK concentration.

9.2.2 Listings and Descriptive Summary Statistics – Urine PK

ARO-ANG3 urine concentrations/volume and PK parameters will be listed for each subject cohort, dosing day, and sampling time (for the case of spot collection) or time interval. ARO-ANG3 urine PK parameters will be summarized by study part, treatment (dose level) and dosing day using the PK Analysis Population.

Summary tabulations will display the number of observations, mean, SD, CV (%), median, minimum and maximum.

9.2.3 Pharmacokinetic Dose Proportionality

The dose proportionality of the Day 1 plasma PK parameters (AUC_{0-t}, AUC_{0-inf}, and C_{max}) for cohort 1-4, and cohort 2b, 3b, and 4b, will be investigated using the following power model,

ln(PK parameter) = $\beta_0 + \beta_1$ * ln(dose), where β_0 is the intercept and β_1 is the slope.

Each log-transformed PK parameter will be fit with a power model with a fixed effect term for log-transformed dose. For each PK parameter, the slope and associated 90% CI will be presented. A minimum of 3 values per dose cohort must be available for a given parameter to estimate dose proportionality with the power model.

The following is the example SAS code to be implemented:

```
PROC MIXED DATA=PKPARM;
      BY PARAMCD;
      MODEL LOGPK= LOGDOSE / SOLUTION CL;
      ESTIMATE "SLOPE" LOGDOSE 1 / ALPHA = 0.1 CL;
      ESTIMATE "INTERCEPT" INTERCEPT 1 / ALPHA = 0.1 CL;
      ODS OUTPUT SOLUTIONF =SOLNF;
      ODS OUTPUT ESTIMATES =ESTIMATE;
RUN;
```

Dose proportionality will be indicated if the 90%CIs for β all contain 1.

Plasma PK dose proportionality will also be assessed using the Day 29 PK parameters.

The dose proportionality may also be performed by dose level which included different population as appropriate.

10. SAFETY

Statistical methods for the safety analyses will be primarily descriptive in nature. Safety for each study part and treatment group (dose level) for active drug patients and for placebo patients pooled will be summarized separately.

Safety endpoints included in the analysis are AEs, injection site reactions, concomitant medications, clinical laboratory assessments, vital signs, physical examination, 12-lead ECG and reasons for treatment discontinuation. Safety endpoints will be analyzed using the Safety Population. Adverse Events, ISRs and Concomitant Medications are discussed in section 10 below. Laboratory Abnormalities, Pregnancies, Vital Signs, Physical Exam and ECGs are discussed later in their respective sections.

10.1 Adverse Events

Adverse events (AEs) will be coded using the Medical Dictionary for Regulatory Activities (MedDRA®, the latest available version), and data will be summarized by System Organ Class (SOC) and Preferred Term (PT). The number and percent of subjects reporting each AE will be summarized for each cohort and treatment group as well as overall. A participant with two or more AEs within the same level of summarization (i.e. SOC or PT) will be counted only once in that level. Percentages will be based on the number of subjects in the Safety Population within each dose level (cohort). The number of AEs reported will also be presented. A count of the total number of AEs for each cohort and treatment group, including multiple AEs per participant will also be provided.

Treatment-emergent AEs (TEAEs) are defined as pre-treatment existing conditions that worsen after study drug administration, or events that occur during the course of the study, during or after administration of study drug. Only TEAEs will be included in the AE summary tables, which will present data by cohort for active treatment patients and pooled for normal healthy volunteer cohort placebo subjects (cohorts 1, 2, 3 and 4) and patient cohort placebo subjects (cohorts 5 and 6). In the case of a missing AE start date or stop date, the most conservative approach will be followed, whereby an AE is classified as a TEAE unless it is not possible that the AE could be treatment emergent.

A table providing an overall summary of AEs will be produced. This will include the number of TEAEs; the number and percentage of subjects reporting at least one: TEAE, serious TEAE, grade 3 (severe) or higher TEAE, TEAE related to study treatment (possibly or probably related to ARO-ANG3), serious TEAE related to study treatment, grade 3 or higher TEAE related to the study drug and TEAE leading to drug or study withdrawal. TEAEs will also be tabulated by severity and by relationship to treatment.

For each cohort considered separately, additional AE tables will be generated as follows:

- TEAEs by SOC and PT in decreasing order of frequency
- TEAEs by PT in decreasing order of frequency
- TEAEs by Severity
- TEAEs by Relationship to Study Drug (ARO-ANG3)
- Treatment Emergent SAEs
- Study Drug (ARO-ANG3 or placebo) Related SAEs by SOC and PT
- TEAEs Leading to Study Drug or Study Withdrawal
- TEAEs occurring in >1 study participant in decreasing order of frequency

A by-participant AE data listing, including verbatim term, MedDRA (latest version) SOC and PT, severity, outcome and relationship to study treatment, will be provided. Separate listings will be generated for SAEs. AEs that started during the follow-up period will be flagged.

10.2 Adverse Events (AEs) at the Injection Site and Local Injection Site Reactions (LISR)

For purposes of data analysis, a local injection site reaction (LISR) is defined as an adverse reaction (usually immunologic) developing at the site of injection, lasting at least 48 hours, and is based on the specified MedDRA preferred terms provided below. For data analysis purposes AEs at the injection site with reported terms of bruising or hematoma will not be considered injection site reactions.

LISRs will only include events that start within 24 hours of the injection and persist for at least 48 hours from time of onset. Events for which it is not possible to determine whether the event duration was at least 48 hours (due to a partial start or end date or time) will be considered as LISRs.

Local Injection Site Reactions (LISR) will be reviewed based on description of symptoms, level of severity (mild, moderate or severe) and outcome of the reactions as well as other relevant data elements such as de/re-challenge, medical history, and possible confounding variables. Based on the review, the reactions will be specifically categorized and collated for the analysis.

The results will be summarized by cohort across all dose levels and percentages for the reported LISR by using the MedDRA coding system by System Organ Class (SOC), General disorders and administration site conditions and Preferred Terms that are associated with local injection site reactions.

The following MedDRA Preferred Terms determined by the Sponsor's pharmacovigilance personnel represent the LISR:

Injection site discomfort	Injection site abscess
Injection site discoloration	Injection site abscess sterile
Injection site erythema	Injection site atrophy
Injection site irritation	Injection site calcification
Injection site inflammation	Injection site cellulitis
Injection site induration	Injection site dermatitis
Injection site pain	Injection site erosion
Injection site oedema	Injection site fibrosis
Injection site pruritus	Injection site indentation
Injection site rash	Injection site necrosis
Injection site urticaria	Injection site nodule
Injection site reaction	Injection site ulcer
Injection site swelling	

Those summaries will only include events that start within 24 hours of injection and persist for at least 48 hours from the onset of event. Events with onset within 24 hours of injection and missing resolution date will also be included in the summary.

The percentage of injections leading to local injection site reactions will be summarized using descriptive statistics.

The following calculation will be utilized to determine the percentage of injections leading to local injection site reactions for each participant:

(A/B)*, where A = number of injections with a local injection site reaction, and B = total number of injections.

The results of local injections site reactions will be provided in summary tabulation.

The same analysis applied to LISR's will be applied to all AE's at the injection site.

For the extension cohort (Cohort 9), an event that starts post the first dosing of Cohort 9, will be attributed to Cohort 9 only.

10.3 Concomitant medication

Concomitant medications and non-drug therapies will be coded using the World Health Organization Drug Dictionary (WHO-DD, the latest available version). Concomitant medications are medications taken at least once after the start of first study-drug administration. Medications stopped prior to the day of the start of first study-drug administration will not be considered concomitant medication. Prior medications will not be summarized, but they will be listed along with concomitant medications. Only concomitant medications will be summarized. Medications that started during the follow-up period will only be included in the follow-up concomitant summary table and will be flagged in the listing.

Individual data listings will be presented for each participant and summarized by WHO-DD Anatomical Therapeutic Chemical (ATC) anatomical group, and preferred term using frequency counts and percentages. Subjects who take the same medication more than once will be counted only once for that preferred term.

The number of concomitant medications reported, and the number and percentage of subjects reporting at least one concomitant medication will be presented per cohort. The number and percentage of patients reporting concomitant medications associated with each ATC anatomical group and preferred term will be reported by cohort.

For the extension cohort (Cohort 9), concomitant medications that are taken at least once after the first dosing of Cohort 9, will be attributed to Cohort 9. If the concomitant medication start date/time is before the first dosage given for Cohort 9, the concomitant medication will be assigned to the Cohort 9 as well as the previous cohort the participant was assigned to. Otherwise the concomitant medication will be assigned Cohort 9.

10.4 Laboratory (excluding PK/PD)

10.4.1 Definition of variables

Hematology Parameters

- Hemoglobin
- Red Blood Cell Count
- Hematocrit
- Mean Cell Volume (MCV)
- Mean Cell Hemoglobin (MCH)
- Mean Cell Hemoglobin Concentration (MCHC)
- Platelets
- White Blood Cell Count
- Neutrophils
- Lymphocytes
- Monocytes
- Eosinophils
- Basophils

Chemistry Parameters

- Sodium
- Potassium
- Chloride
- Bicarbonate

- Glucose
- Urea
- Creatinine (including calculated creatinine clearance)
- Creatine kinase
- Uric acid
- Phosphate
- Total calcium
- Anion gap
- Albumin
- Globulins
- Protein
- Total bilirubin
- Conjugated bilirubin
- Gamma glutamyl transferase (GGT)
- Alkaline phosphatase (ALP)
- Alanine aminotransferase (ALT)
- Aspartate transaminase (AST)
- Lactate dehydrogenase (LD)
- Lipase
- C-reactive protein
- Troponin I

Coagulation Parameters

- Partial Thromboplastin Time (PTT)
- Prothrombin Time (PT)
- INR
- Fibrinogen

*Urinalysis Parameters**

- Leukocytes
- Nitrites
- Urobilinogen
- Protein
- pH
- Blood
- Specific Gravity
- Ketone
- Bilirubin
- Glucose

*Microscopic urinalysis will be performed if indicated: White blood cells, red blood cells, epithelial cells, and bacteria.

Serology

- Hepatitis B surface antigen
- Hepatitis C antibody
- HIV antibody screen

Serum insulin levels

- Insulin levels will be measured as per the Schedule of Assessments.

Serum glucose levels

- Blood glucose levels will be measured as per the Schedule of Assessments. Glucose level included in metabolic panel is acceptable.

FSH

Post-menopausal status will be confirmed by follicle-stimulating hormone (FSH) level consistent with post-menopausal state.

Drug and Alcohol Use Screen

The following will be tested for by a urine drug screen

- Benzodiazepines
- Amphetamines
- Barbiturates
- Methamphetamines
- Methadone
- Opiates
- Phencyclidine
- Cannabinoids
- MDMA
- Cocaine

An alcohol breath test will be done to test for alcohol consumption.

Pregnancy

Females of childbearing potential will have a urine pregnancy test.

Other

- Stool occult blood
- lipid metabolic genotype (drawn on all consenting subjects but analyzed only if scientifically warranted at sponsor discretion)

10.4.2 Biostatistical methods

All hematology, chemistry, coagulation and urinalysis parameters, as well as blood glucose levels and insulin levels will be summarized using descriptive statistics for each dose level (cohort) for all time points assessed, including change from baseline (last valid pre-dose value) for all post-dose assessments.

For specific laboratory values (ALT, AST, platelets, CK, ALP, T bilirubin, Creatinine, lipase, GGT) shift tables will be provided cross-tabulating the laboratory value grade at the baseline visit against the worst grade at any of the post-baseline visits. For this purpose, laboratory values will be graded according to the CTCAE criteria v5.

All remaining laboratory values will be compared to the normal range of the single local laboratory, and values that fall outside of the normal ranges will be flagged as: H (High) or L (Low) in the data listings. Results that were flagged as 'LP' will be reported as 'L' and results flagged as 'HP' will be reported as 'H'. Shift tables from Day 1 (pre-dose) to all follow-up visits will be generated for each of these hematology, chemistry and coagulation laboratory parameters with values of Within Normal Limits (WNL), High, and Low used for the shift categories.

Early termination visit value will be kept as a nominal visit value, as well as mapped to the closest Study Visit using visit window. The closest value to the scheduled visit date will be used for the Scheduled Visits value in case of multiple records. The visit windows can be viewed in Appendix B of this SAP.

All laboratory data will be included in the by-participant data listings. Microscopic urinalysis will only be listed. Listings will be provided for both Central Laboratory readings and Local Laboratory readings separately.

Drug and Alcohol Use results will be listed. FSH and pregnancy test results will also be listed. Pregnancy test results will be summarized separately by time point.

The above methods will exclude cohort 9, as separate summary tables will be produced for cohort 9 for hematology, chemistry and coagulation parameters, considering the different assessment time points for these laboratory values for this cohort. Basic descriptive statistics will be provided for each time point and each laboratory parameter, considering both the actual values and the change from baseline.

10.5 Vital Signs

10.5.1 Definition of variables

- Systolic Blood Pressure (SBP) (mmHg)
- Diastolic Blood Pressure (DBP) (mmHg)
- Pulse Rate (beats/min)
- Body Temperature (°C)
- Respiratory rate (breaths/minute)

10.5.2 Biostatistical methods

All vital sign parameters will be summarized using descriptive statistics for each dose level (cohort) for all time points assessed, including change from baseline (last valid pre-dose value) for all post-dose assessments. Pre-dosing vital signs on Day 1 will be used as baseline data. If the Day 1 pre-dose baseline value is missing, then the data from screening will be used as baseline data as appropriate.

Early termination visit value will be kept as a nominal visit value, as well as mapped to the closest Study Visit using visit window. The closest value to the scheduled visit date will be used for the Scheduled Visits value in case of multiple records. The visit windows can be viewed in Appendix B of this SAP.

All vital sign parameters will be listed per cohort for active drug subjects, for placebo subjects pooled across cohorts, as well as for all patients (active drug and placebo) considered collectively, for all time points assessed.

The above methods will exclude cohort 9, as a separate summary table of the vital signs will be produced for cohort 9, considering the different assessment time points for vital signs for this cohort. Basic descriptive statistics will be provided for each time point for each vital sign parameter, considering both the actual values and the change from baseline.

10.6 Physical examination

10.6.1 Definition of variables

The following body systems will be assessed:

- General Appearance
- HEENT (Head, Eyes, Ears, Nose and Throat)
- Cardiovascular
- Lungs
- Abdomen
- Lymph nodes
- Genitourinary
- Extremities
- Neurological
- Skin
- Musculoskeletal
- Other

Abnormal results of physical examinations will be categorized as follows:

- Abnormal NCS (Not Clinically Significant)
- Abnormal CS (Clinically Significant).

10.6.2 Biostatistical methods

Physical examination will be summarized by study visit and treatment cohort for active drug patients, for placebo patients pooled across cohorts, as well as for all patients (active drug and placebo) considered collectively. The frequency counts and percentages of subjects with different physical examination results and their clinical significance (for abnormal) will be summarized.

According to the schedule of assessments, a complete physical exam will be performed at Screening. Symptom-directed physical examinations will only be conducted at other visits as necessary. Only complete exams (screening) will be summarized.

Early termination visit value will be kept as a nominal visit value, as well as mapped to the closest Study Visit using visit window. The closest value to the scheduled visit date will be used for the Scheduled Visits value in case of multiple records. The visit windows can be viewed in Appendix B of this SAP.

By-participant data listings will be generated for all the physical examination data, including the Investigator assessment of clinical significance for abnormal findings, for all time points assessed.

10.7 12-lead ECG

10.7.1 Definition of variables

- Heart Rate (beats/min)
- PR interval (msec)
- QRS interval (msec)
- QT interval (msec)
- QTcF interval (msec)
- Overall interpretation of 12-lead ECG:
    - Normal
    - Abnormal NCS (Not Clinically Significant)
    - Abnormal CS (Clinically Significant)

10.7.2 Biostatistical methods

Descriptive statistics will be calculated for heart rate, PR interval, QRS interval, QT interval, and QTcF interval, including change from baseline (last valid pre-dose value on Day 1) for each dose level (cohort) for all time points assessed.

12-lead ECG measurements will be obtained at the scheduled time points after the participant is semi-supine for at least 3 minutes. Any abnormal ECGs will be repeated in triplicate, with each measurement approximately 1 minute apart. If triplicate ECG parameters are available, the average of the triplicate ECG parameters will be used. Unscheduled visits will be excluded from summary tables. However, the findings from the unscheduled visits will be listed.

In addition, the overall interpretation of 12-lead ECG results will be classified using frequency counts and percentages for the categories of Normal, Abnormal NCS and Abnormal CS for each treatment group for all time points assessed.

Early termination visit value will be kept as a nominal visit value, as well as mapped to the closest Study Visit using visit window. The closest value to the scheduled visit date will be used for the Scheduled Visits value in case of multiple records. The visit windows can be viewed in Appendix B of this SAP.

All ECG data will be presented in the by-participant data listings.

10.8 Pharmacodynamics

10.8.1 Definition of variables

- Fasting serum ANGPTL3
- LDL-C direct
- LDL-C (by PUC)
- Total Cholesterol
- non-HDL-C
- HDL-C
- VLDL-C
- Fasting triglycerides
- Lp(a)
- Total apoB
- apoB-100
- apoB-48
- apoC-III
- apoC-II
- apoA-I
- lipoprotein lipase mass (if feasible)
- CETP mass (if feasible)
- apoA-V
- Serum insulin
- Fasting glucose
- Hemoglobin A1c
- C-peptide

10.8.2 Biostatistical methods

Pharmacodynamic measurements (lipid values) at baseline (last valid pre-dose value) as well as their nadir value and change from baseline to nadir (absolute and percentage) will be summarized using descriptive statistics for each cohort considered separately for active drug patients, for placebo patients pooled across cohorts, as well as for all patients (active drug and placebo) considered collectively. All PD parameters above will be summarized using SI units as well as standard US units as required for IND submission.

For each laboratory parameter and Cohort, the nadir to be used is the value at the time point at which the average of the parameter value is lowest or highest from baseline across that Cohort, depending on the direction of the change. Early termination visit value will be kept as a nominal visit value, as well as mapped to the closest Study Visit using visit window. The closest value to the scheduled visit date will be used for the Scheduled Visits value in case of multiple records. The visit windows can be viewed in Appendix B of this SAP.

At each post-baseline assessment time point, (Day 8, Day 15, Day 29, Day 43, Day 57, Day 71, Day 85, Day 99 and Day 113), as well as a nominal early termination visit, summary statistics of the pharmacodynamic measurements, their absolute change from baseline and their percentage change from baseline will be provided.

All PD parameter data will also be listed by participant.

For each of the pharmacodynamic measurements, paired t-tests will be used to estimate differences between baseline and each post-baseline time point for each treatment group, as well as the corresponding 95% CI and p-value.

Percent change from baseline in ANGPTL3 and other selected PD parameters (fasting LDL-C, Total Cholesterol, non-HDL-C, HDL-C, VLDL-C, Triglycerides, total apoB, apoB-48, apoB-100, and apoA-I) will be evaluated using a linear mixed model repeated measures (MMRM) approach. The repeated measures are the percentage change from baseline values measured at scheduled Week 2-16 study visits. The model will include fixed effects for treatment, week, treatment by week interaction, baseline value as a continuous covariate, and baseline by treatment interaction. Only cohorts that have placebo control participants will be analyzed using MMRM. Specifically, up to three separate MMRM models will be constructed, corresponding to the following three comparisons, each considered separately:

1. compare active drug participants from Cohorts 1, 2, 3 and 4 against pooled placebo participants from these 4 cohorts;
2. compare active drug participants from Cohort 5 against placebo participants from the same Cohort (participant to sufficient data being available);
3. compare active drug participants from Cohort 6 against placebo participants from the same Cohort (participant to sufficient data being available).

The analysis will be carried out using the SAS PROC MIXED procedure using an unstructured within-patient covariance structure and Kenward-Roger approximation to estimate the degrees of freedom for tests of fixed effects. In case of any convergence issues, additional covariance structures will be investigated, or the interaction term of treatment and week may be omitted from the model. Model-based estimates of least squares means, standard errors, treatment differences in least squares means, and 95% confidence intervals will be reported for each time point while p-values will be presented for the Week 16 time point only.

SAS code is provided below.

```
PROC MIXED DATA=XXX;
CLASS SUBJID TREATMENT VISIT;
MODEL PCTCHGBL=TREATMENT VISIT TREATMENT*VISIT BASE TREATMENT*BASE/
DDFM=KR;
REPEATED VISIT/SUBJECT=SUBJID TYPE=UN;
LSMEANS TREATMENT*VISIT/ALPHA=0.05 CL DIFF;
ESTIMATE "TRT DIFF AT WEEK 2" TREATMENT 1 -1 TREATMENT*WEEK 1 0 0 0 0 0
0 0  -1 0 0 0 0 0 0 0/CL;
RUN;
```

This summary table will exclude cohort 9, as a separate summary table of the pharmacodynamic measurements will be produced for cohort 9, considering the different assessment time points for the pharmacodynamic measurements for this cohort. Basic descriptive statistics will be provided for the nadir value, change between baseline and nadir value, both in absolute and relative terms. The value recorded at the last assessment prior to the original entry into the study under Cohort 7, 7b or 7c will be taken as the baseline value. Similarly, the nadir will be the lowest value recorded since the original entry into the study.

GTT and post-prandial triglyceride (PPTG) testing will be measured at time points outlined in the Schedule of Assessments. In Cohort 8, post-prandial TG evaluation is only to be completed in patients who are not at risk for post-prandial hypertriglyceridemia related abdominal pain or pancreatitis. GTT is optional in any patient with diabetes mellitus.

Change from baseline in glucose tolerance, insulin tolerance and % liver fat fraction from MRI-PDFF will be summarized by descriptive statistics.

For ANGPTL3, triglycerides and LDL-C, the proportion of participants achieving durable response will be summarized per cohort and overall. Durable response will be defined as achieving a laboratory parameter value below a pre-specified threshold at or before the Day 113 follow-up visit and maintaining it until the Day 113 follow-up visit. Results will be reported for three separate thresholds – 20% of baseline, 40% of baseline and 80% of baseline. The following four durable response categories will be considered.

- **Never Achieved Response** – Participants with no results meeting the response level at any timepoint up until and including Day 113.
- **Achieved and Maintained Response –** Participants who achieved the response level and maintained the response at every subsequent timepoint up until and including Day 113.
- **Achieved and Lost Response** - Participants who achieved the response level, however, at least one subsequent timepoint did not achieve the response. This includes participants that re-achieved the response at Day 113. Participants who were observed to lose the response but whose outcome at Day 113 is unknown due either to withdrawal/termination or due to the data being unavailable as a result of the data cut-off were also included.
- **Censored** - Participants who either have or have not achieved the response, but for whom the outcome at Day 113 is unknown either due to withdrawal/termination or due to the data being unavailable as a result of the data cut-off.

The proportion of participants achieving durable response will be further summarized per cohort and overall, at assessment visits prior to Day 113. For the purpose of this summary, the participant's durable response status will be considered to be in one of the following 3 categories at each assessment visit.

- **Below Response Threshold** –parameter value below the threshold of interest (20%, 40% or 80% of baseline) at the assessment visit under consideration.
- **Above Response Threshold –** above or at the threshold of interest (20%, 40% or 80% of baseline) at the assessment visit under consideration
- **Assessment Not Available** – parameter value was not available at the assessment visit under consideration.

11. IMMUNOGENICITY

For Cohorts 5, 6, 7, 7b, 7c and 8, blood samples for anti-drug antibodies testing will be collected at pre-dose, Day 57, and at the End of Study visit (Day 113) or at Early Termination as per Schedule of Assessments. Anti-drug antibodies will be analyzed using the Safety Population.

For continuous antibody measurement data, the observed assay value and change from baseline (last pre-dose value on Day 1) for each dose level (cohort) for all time points assessed will be summarized descriptively. In addition to other descriptive statistics, the summary statistics will also include geometric mean in the table. If categorical data (negative/positive) are reported for antibody measurement data, these values will be summarized as well by frequency counts and percentages.

All anti-drug antibodies measures will be presented in the by-participant data listings.

12. HANDLING OF MISSING DATA

Missing date/time imputation rules for AEs, concomitant medications and procedures are described in sections 3.1, 3.2 and 3.3 of this SAP.

The imputation rules for non-quantifiable PK concentration values are described in section 9.1.1 and 19.1 of this SAP.

13. CHANGES TO THE PLANNED ANALYSIS

Any changes to the analyses outlined in the approved SAP will be detailed in the Clinical Study Report (CSR).

14. INTERIM AND FINAL ANALYSIS

14.1 Dose Escalation, Data Safety Committee (DSC) Analyses

Dose escalation will require approval by the DSC based on all cumulative available safety data through Day 8 of the current cohort. Cohorts 2b-4b and 5-8 will be dosed on Day 1 and Day 29. DSC decisions will be based on all aggregate safety data available including all data available at least through Day 8 of the current cohort as shown in Figure 1. Dose escalation will proceed until all cohorts are fully enrolled or until the study is stopped or the DSC votes to not escalate to the next dose.

Blinding will be preserved to the extent possible for the HVs; however, treatment un-blinding may occur, at the PI's discretion, where deemed necessary for treatment of an AE or for a decision to be made regarding trial continuation.

A formal charter will be in place prior to study start and will establish the rules, meeting frequency and scope of responsibilities of the DSC.

14.2 Interim Analyses

Sponsor may request an interim descriptive analysis of the change from baseline in ANGPTL3, apoC-III and other measured lipid parameters any time after all HV subjects planned for enrollment in each cohort have received at least one dose of ARO-ANG3 or PBO. This interim analysis is for planning of future studies and will not impact the conduct of this study. Sponsor will remain blinded to all participant treatment assignments.

Sponsor may request an interim descriptive analysis of the change from baseline in ANGPTL3, apoC-III and other measured lipid parameters any time after all healthy volunteer participants planned for enrollment in each cohort have received at least one dose of ARO-ANG3 or Placebo. This interim analysis is for the planning of future studies and will not impact the conduct of this study.

An independent unblinded team comprising a Data Manager, Statistician and Statistical Programmers will perform analysis on the interim dataset. The Sponsor may be unblinded, however, the primary study team will remain blinded to all participant treatment assignments.

Interim Tables, Listings and Figures will be based on data available until the interim data cut-off date. The listings and summary tables produced for AEs will be based on analysis datasets meeting the following conditions:

All EDC AE data will be employed where the AE has been coded under MEDDRA.

AE's that were recorded in the screening period for participants that were not subsequently treated will not be included in the summary tables.

If the AE start date is incomplete, such as when the start day is unknown, the day will be imputed as the end of the month for the purposes of determining if the AE is treatment-emergent.

The listings, figures and summary tables for the safety and PD laboratory data will be based on data available until the interim data cut-off date from both the Central Safety and PD laboratories and will be based on analysis datasets meeting the following conditions:

No results from samples collected after the interim data cut-off date will be listed or summarized.

Primarily for the safety lab summaries, the central local laboratory data will be employed.

Medpace Research Labs (MRL) will be used as the preferred data source for calculations/analysis for the following PD markers: fasting direct LDL-C, LDL-C by PUC, Total Cholesterol, non-HDL-C, HDL-C, VLDL-C, Triglycerides, Total ApoB, apoB-48, apoB-100, apoC-III, apoA-I, apoC-II, apoA5, lipoprotein lipase mass, CETP mass. Where results are not available in the PD lab dataset for a sample, if possible, the results will be employed from the central lab dataset.

If necessary, data may be converted to accommodate a difference in reporting units between the primary laboratory data and substitute laboratory data.

Parameters with no data will not be reported.

All other interim outputs will be based on EDC data available until the interim data cut-off date.

14.3 Final Analysis (End of Study)

The end of study analysis will be based on the final version of the SAP.

15. SOFTWARE

The following software will be used to perform the statistical analyses: SAS® Version 9.4 or higher (SAS Institute, Cary, North Carolina, USA).

16. TABLES

As indicated in the table of tables below, tables will be produced separately for healthy volunteer cohorts (Cohorts 1, 2, 3, 4, 5, 2b, 3b and 4b), and Patient Cohorts (Cohorts 6, 7, 7b, 7c, 8 and 9).

All tables that relate to parameters summarized in the baseline disease characteristics tables will be produced twice, once considering SI units and once considering US units. This includes the baseline disease characteristics tables themselves, as well as all PD measurement tables and all MMRM tables.

For brevity these tables do not appear in the list of tables below, but in the actual TFL's they will be included with a table number of a.2 where the table a.1 is the corresponding table in SI units.

No.	Title	Analysis Population	Include in interim analysis?
**14.1**	**Demographics and Other Baseline Characteristics**		
14.1.1.1	Participant Enrolment and Disposition, Healthy Volunteer Cohorts	Safety Population	X
14.1.1.2	Participant Enrolment and Disposition, Disease Cohorts	Safety Population	X
14.1.2.1	Demographics and Baseline Characteristics, Healthy Volunteer Cohorts	Safety Population	X
14.1.2.2	Demographics and Baseline Characteristics, Disease Cohorts	Safety Population	X
14.1.3.1	Pregnancy Test Results by Time Point, Healthy Volunteer Cohorts	Safety Population	X
14.1.3.2	Pregnancy Test Results by Time Point, Disease Cohorts	Safety Population	X
14.1.4.x.1	Baseline Pharmacodynamic Characteristics, Healthy Volunteer Cohorts	Pharmacodynamic Population	X
14.1.4.x.2	Baseline Pharmacodynamic Characteristics, Disease Cohorts	Pharmacodynamic Population	X
**14.2**	**PK/PD**		
14.2.1.1	Plasma Concentrations of ARO-ANG3 (ng/mL) by Dose, Healthy Volunteer Cohorts	Pharmacokinetic Population	
14.2.2.1.1	Plasma Pharmacokinetic Parameters of ARO-ANG3, Healthy Volunteer Cohorts	Pharmacokinetic Population	

No.	Title	Analysis Population	Include in interim analysis?
14.2.2.1.1b	Dose-normalized Plasma ARO-ANG3 of C^{max} and AUCs by Sex, Healthy Volunteer Cohorts	Pharmacokinetic Population	
14.2.2.2.1	Urine Pharmacokinetic Parameters of ARO-ANG3, Healthy Volunteer Cohorts	Pharmacokinetic Population	
14.2.2.3.1	Analysis of Dose Proportionality for ARO-ANG3 using the Power Model, Healthy Volunteer Cohorts	Pharmacokinetic population	
14.2.3.2	Anti-drug Antibodies, Disease Cohorts	Safety Population	
14.2.4.1.x.1	Pharmacodynamic Measurements and Change Between Baseline and Nadir Values, Healthy Volunteer Cohorts	Pharmacodynamic Population	X
14.2.4.1.x.2	Pharmacodynamic Measurements and Change Between Baseline and Nadir Values, Disease Cohorts	Pharmacodynamic Population	X
14.2.4.2.x.1	Fasting serum ANGPTL3 - Absolute Values and Change from Baseline, Healthy Volunteer Cohorts	Pharmacodynamic Population	X
14.2.4.2.x.2	Fasting serum ANGPTL3 - Absolute Values and Change from Baseline, Disease Cohorts	Pharmacodynamic Population	X
14.2.4.3.x.1	LDL-C direct - Absolute Values and Change from Baseline, Healthy Volunteer Cohorts	Pharmacodynamic Population	X
14.2.4.3.x.2	LDL-C direct - Absolute Values and Change from Baseline, Disease Cohorts	Pharmacodynamic Population	X
14.2.4.4.x.1	LDL-C (by PUC) - Absolute Values and Change from Baseline, Healthy Volunteer Cohorts	Pharmacodynamic Population	X
14.2.4.4.x.2	LDL-C (by PUC) - Absolute Values and Change from Baseline, Disease Cohorts	Pharmacodynamic Population	X
14.2.4.5.x.1	Total Cholesterol - Absolute Values and Change from Baseline, Healthy Volunteer Cohorts	Pharmacodynamic Population	X

No.	Title	Analysis Population	Include in interim analysis?
14.2.4.5.x.2	Total Cholesterol - Absolute Values and Change from Baseline, Disease Cohorts	Pharmacodynamic Population	X
14.2.4.6.x.1	non-HDL-C - Absolute Values and Change from Baseline, Healthy Volunteer Cohorts	Pharmacodynamic Population	X
14.2.4.6.x.2	non-HDL-C - Absolute Values and Change from Baseline, Disease Cohorts	Pharmacodynamic Population	X
14.2.4.7.x.1	HDL-C - Absolute Values and Change from Baseline, Healthy Volunteer Cohorts	Pharmacodynamic Population	X
14.2.4.7.x.2	HDL-C - Absolute Values and Change from Baseline, Disease Cohorts	Pharmacodynamic Population	X
14.2.4.8.x.1	VLDL-C - Absolute Values and Change from Baseline, Healthy Volunteer Cohorts	Pharmacodynamic Population	X
14.2.4.8.x.2	VLDL-C - Absolute Values and Change from Baseline, Disease Cohorts	Pharmacodynamic Population	X
14.2.4.9.x.1	Fasting triglycerides - Absolute Values and Change from Baseline, Healthy Volunteer Cohorts	Pharmacodynamic Population	X
14.2.4.9.x.2	Fasting triglycerides - Absolute Values and Change from Baseline, Disease Cohorts	Pharmacodynamic Population	X
14.2.4.10.x.1	Lp(a) - Absolute Values and Change from Baseline, Healthy Volunteer Cohorts	Pharmacodynamic Population	
14.2.4.10.x.2	Lp(a) - Absolute Values and Change from Baseline, Healthy Volunteer Cohorts	Pharmacodynamic Population	
14.2.4.11.x.1	Total apoB - Absolute Values and Change from Baseline, Healthy Volunteer Cohorts	Pharmacodynamic Population	X
14.2.4.11.x.2	Total apoB - Absolute Values and Change from Baseline, Disease Cohorts	Pharmacodynamic Population	X
14.2.4.12.x.1	apoB-100 - Absolute Values and Change from Baseline, Healthy Volunteer Cohorts	Pharmacodynamic Population	X

No.	Title	Analysis Population	Include in interim analysis?
14.2.4.12.x.2	apoB-100 - Absolute Values and Change from Baseline, Disease Cohorts	Pharmacodynamic Population	X
14.2.4.13.x.1	apoB-48 - Absolute Values and Change from Baseline, Healthy Volunteer Cohorts	Pharmacodynamic Population	X
14.2.4.13.x.2	apoB-48 - Absolute Values and Change from Baseline, Disease Cohorts	Pharmacodynamic Population	X
14.2.4.14.x.1	apoC-III - Absolute Values and Change from Baseline, Healthy Volunteer Cohorts	Pharmacodynamic Population	X
14.2.4.14.x.2	apoC-III - Absolute Values and Change from Baseline, Disease Cohorts	Pharmacodynamic Population	X
14.2.4.15.x.1	apoC-II - Absolute Values and Change from Baseline, Healthy Volunteer Cohorts	Pharmacodynamic Population	X
14.2.4.15.x.2	apoC-II - Absolute Values and Change from Baseline, Disease Cohorts	Pharmacodynamic Population	X
14.2.4.16.x.1	lipoprotein lipase mass - Absolute Values and Change from Baseline, Healthy Volunteer Cohorts	Pharmacodynamic Population	X
14.2.4.16.x.2	lipoprotein lipase mass - Absolute Values and Change from Baseline, Disease Cohorts	Pharmacodynamic Population	X
14.2.4.17.x.1	CETP mass - Absolute Values and Change from Baseline, Healthy Volunteer Cohorts	Pharmacodynamic Population	X
14.2.4.17.x.2	CETP mass - Absolute Values and Change from Baseline, Disease Cohorts	Pharmacodynamic Population	X
14.2.4.18.x.1	apoA-V - Absolute Values and Change from Baseline, Healthy Volunteer Cohorts	Pharmacodynamic Population	X
14.2.4.18.x.2	apoA-V - Absolute Values and Change from Baseline, Disease Cohorts	Pharmacodynamic Population	X

No.	Title	Analysis Population	Include in interim analysis?
14.2.4.19.x.1	Serum insulin - Absolute Values and Change from Baseline, Healthy Volunteer Cohorts	Pharmacodynamic Population	X
14.2.4.19.x.2	Serum insulin - Absolute Values and Change from Baseline, Disease Cohorts	Pharmacodynamic Population	X
14.2.4.20.x.1	Fasting glucose - Absolute Values and Change from Baseline, Healthy Volunteer Cohorts	Pharmacodynamic Population	X
14.2.4.20.x.2	Fasting glucose - Absolute Values and Change from Baseline, Disease Cohorts	Pharmacodynamic Population	X
14.2.4.21.x.1	Hemoglobin A1c - Absolute Values and Change from Baseline, Healthy Volunteer Cohorts	Pharmacodynamic Population	X
14.2.4.21.x.2	Hemoglobin A1c - Absolute Values and Change from Baseline, Disease Cohorts	Pharmacodynamic Population	X
14.2.4.22.x.1	C-peptide (ng/mL) - Absolute Values and Change from Baseline, Healthy Volunteer Cohorts	Pharmacodynamic Population	X
14.2.4.22.x.2	C-peptide (ng/mL) - Absolute Values and Change from Baseline, Disease Cohorts	Pharmacodynamic Population	X
14.2.4.23.x.3	Pharmacodynamic Parameters - Absolute Values and Change from Baseline, Cohort 9	Pharmacodynamic Population	X
14.2.4.24.1	Durable Response Categorization at Day 113, Healthy Volunteer Cohorts	Pharmacodynamic Population	X
14.2.4.24.2	Durable Response Categorization at Day 113, Disease Cohorts	Pharmacodynamic Population	X
14.2.4.25.1	Proportion of Participants with Durable Response, Healthy Volunteer Cohorts	Pharmacodynamic Population	X
14.2.4.25.2	Proportion of Participants with Durable Response, Disease Cohorts	Pharmacodynamic Population	X
14.2.4.26.x.1	MMRM Analysis of Serum ANGPTL3 up to Day 99 and Change from Baseline, Healthy Volunteer Cohorts	Pharmacodynamic Population	X

No.	Title	Analysis Population	Include in interim analysis?
14.2.4.26.x.2	MMRM Analysis of Serum ANGPTL3 up to Day 99 and Change from Baseline, Disease Cohorts	Pharmacodynamic Population	X
14.2.4.27.x.1	MMRM Analysis of Serum ANGPTL3 at Day 113 (EOS) and Change from Baseline, Healthy Volunteer Cohorts	Pharmacodynamic Population	X
14.2.4.27.x.2	MMRM Analysis of Serum ANGPTL3 at Day 113 (EOS) and Change from Baseline, Disease Cohorts	Pharmacodynamic Population	X
14.2.4.28.x.1	MMRM Analysis of Serum LDL-C up to Day 99 and Change from Baseline, Healthy Volunteer Cohorts	Pharmacodynamic Population	X
14.2.4.28.x.2	MMRM Analysis of Serum LDL-C up to Day 99 and Change from Baseline, Disease Cohorts	Pharmacodynamic Population	X
14.2.4.29.x.1	MMRM Analysis of Serum LDL-C at Day 113 (EOS) and Change from Baseline, Healthy Volunteer Cohorts	Pharmacodynamic Population	X
14.2.4.29.x.2	MMRM Analysis of Serum LDL-C at Day 113 (EOS) and Change from Baseline, Disease Cohorts	Pharmacodynamic Population	X
14.2.4.30.x.1	MMRM Analysis of Serum Total Cholesterol up to Day 99 and Change from Baseline, Healthy Volunteer Cohorts	Pharmacodynamic Population	X
14.2.4.30.x.2	MMRM Analysis of Serum Total Cholesterol up to Day 99 and Change from Baseline, Disease Cohorts	Pharmacodynamic Population	X
14.2.4.31.x.1	MMRM Analysis of Serum Total Cholesterol at Day 113 (EOS) and Change from Baseline, Healthy Volunteer Cohorts	Pharmacodynamic Population	X
14.2.4.31.x.2	MMRM Analysis of Serum Total Cholesterol at Day 113 (EOS) and Change from Baseline, Disease Cohorts	Pharmacodynamic Population	X
14.2.4.32.x.1	MMRM Analysis of Serum non-HDL-C up to Day 99 and Change from Baseline, Healthy Volunteer Cohorts	Pharmacodynamic Population	X
14.2.4.32.x.2	MMRM Analysis of Serum non-HDL-C up to Day 99 and Change from Baseline, Disease Cohorts	Pharmacodynamic Population	X

No.	Title	Analysis Population	Include in interim analysis?
14.2.4.33.x.1	MMRM Analysis of Serum non-HDL-C at Day 113 (EOS) and Change from Baseline, Healthy Volunteer Cohorts	Pharmacodynamic Population	X
14.2.4.33.x.2	MMRM Analysis of Serum non-HDL-C at Day 113 (EOS) and Change from Baseline, Disease Cohorts	Pharmacodynamic Population	X
14.2.4.34.x.1	MMRM Analysis of Serum HDL-C up to Day 99 and Change from Baseline, Healthy Volunteer Cohorts	Pharmacodynamic Population	X
14.2.4.34.x.2	MMRM Analysis of Serum HDL-C up to Day 99 and Change from Baseline, Disease Cohorts	Pharmacodynamic Population	X
14.2.4.35.x.1	MMRM Analysis of Serum HDL-C at Day 113 (EOS) and Change from Baseline, Healthy Volunteer Cohorts	Pharmacodynamic Population	X
14.2.4.35.x.2	MMRM Analysis of Serum HDL-C at Day 113 (EOS) and Change from Baseline, Disease Cohorts	Pharmacodynamic Population	X
14.2.4.36.x.1	MMRM Analysis of Serum VLDL-C up to Day 99 and Change from Baseline, Healthy Volunteer Cohorts	Pharmacodynamic Population	X
14.2.4.36.x.2	MMRM Analysis of Serum VLDL-C up to Day 99 and Change from Baseline, Disease Cohorts	Pharmacodynamic Population	X
14.2.4.37.x.1	MMRM Analysis of Serum VLDL-C at Day 113 (EOS) and Change from Baseline, Healthy Volunteer Cohorts	Pharmacodynamic Population	X
14.2.4.37.x.2	MMRM Analysis of Serum VLDL-C at Day 113 (EOS) and Change from Baseline, Disease Cohorts	Pharmacodynamic Population	X
14.2.4.38.x.1	MMRM Analysis of Serum Triglycerides up to Day 99 and Change from Baseline, Healthy Volunteer Cohorts	Pharmacodynamic Population	X
14.2.4.38.x.2	MMRM Analysis of Serum Triglycerides up to Day 99 and Change from Baseline, Disease Cohorts	Pharmacodynamic Population	X
14.2.4.39.x.1	MMRM Analysis of Serum Triglycerides at Day 113 (EOS) and Change from Baseline, Healthy Volunteer Cohorts	Pharmacodynamic Population	X

No.	Title	Analysis Population	Include in interim analysis?
14.2.4.39.x.2	MMRM Analysis of Serum Triglycerides at Day 113 (EOS) and Change from Baseline, Disease Cohorts	Pharmacodynamic Population	X
14.2.4.40.x.1	MMRM Analysis of Serum apoB up to Day 99 and Change from Baseline, Healthy Volunteer Cohorts	Pharmacodynamic Population	X
14.2.4.40.x.2	MMRM Analysis of Serum apoB up to Day 99 and Change from Baseline, Disease Cohorts	Pharmacodynamic Population	X
14.2.4.41.x.1	MMRM Analysis of Serum apoB at Day 113 (EOS) and Change from Baseline, Healthy Volunteer Cohorts	Pharmacodynamic Population	X
14.2.4.41.x.2	MMRM Analysis of Serum apoB at Day 113 (EOS) and Change from Baseline, Disease Cohorts	Pharmacodynamic Population	X
14.2.4.42.x.1	MMRM Analysis of Serum apoB-48 up to Day 99 and Change from Baseline, Healthy Volunteer Cohorts	Pharmacodynamic Population	X
14.2.4.42.x.2	MMRM Analysis of Serum apoB-48 up to Day 99 and Change from Baseline, Disease Cohorts	Pharmacodynamic Population	X
14.2.4.43.x.1	MMRM Analysis of Serum apoB-48 at Day 113 (EOS) and Change from Baseline, Healthy Volunteer Cohorts	Pharmacodynamic Population	X
14.2.4.43.x.2	MMRM Analysis of Serum apoB-48 at Day 113 (EOS) and Change from Baseline, Disease Cohorts	Pharmacodynamic Population	X
14.2.4.44.x.1	MMRM Analysis of Serum apoB-100 up to Day 99 and Change from Baseline, Healthy Volunteer Cohorts	Pharmacodynamic Population	X
14.2.4.44.x.2	MMRM Analysis of Serum apoB-100 up to Day 99 and Change from Baseline, Disease Cohorts	Pharmacodynamic Population	X
14.2.4.45.x.1	MMRM Analysis of Serum apoB-100 at Day 113 (EOS) and Change from Baseline, Healthy Volunteer Cohorts	Pharmacodynamic Population	X
14.2.4.45.x.2	MMRM Analysis of Serum apoB-100 at Day 113 (EOS) and Change from Baseline, Disease Cohorts	Pharmacodynamic Population	X

No.	Title	Analysis Population	Include in interim analysis?
14.2.4.46.x.1	MMRM Analysis of Serum apoA-I up to Day 99 and Change from Baseline, Healthy Volunteer Cohorts	Pharmacodynamic Population	X
14.2.4.46.x.2	MMRM Analysis of Serum apoA-I up to Day 99 and Change from Baseline, Disease Cohorts	Pharmacodynamic Population	X
14.2.4.47.x.1	MMRM Analysis of Serum apoA-I at Day 113 (EOS) and Change from Baseline, Healthy Volunteer Cohorts	Pharmacodynamic Population	X
14.2.4.47.x.2	MMRM Analysis of Serum apoA-I at Day 113 (EOS) and Change from Baseline, Disease Cohorts	Pharmacodynamic Population	X
14.2.4.48.1	Glucose Tolerance Test and Change Between Baseline and Day 85 Values, Healthy Volunteer Cohorts	Pharmacodynamic Population	X
14.2.4.48.2	Glucose Tolerance Test and Change Between Baseline and Day 85 Values, Disease Cohorts	Pharmacodynamic Population	X
14.2.4.49.1	Post Prandial Triglycerides Test (PPTG) Results, Healthy Volunteer Cohorts	Pharmacodynamic Population	X
14.2.4.49.2	Post Prandial Triglycerides Test (PPTG) Results, Disease Cohorts	Pharmacodynamic Population	X
14.2.4.50.1	Percentage Fat Fraction on MRI-PDFF	Pharmacodynamic Population	X
**14.3**	**Safety**		
14.3.1.1	Concomitant Medications and Non-Drug Therapies, Healthy Volunteer Cohorts	Safety Population	X
14.3.1.2	Concomitant Medications and Non-Drug Therapies, Disease Cohorts	Safety Population	X
**14.3.3**	**Adverse Events**		
14.3.3.1.1	Overall Summary of Treatment Emergent Adverse Events, Healthy Volunteer Cohorts	Safety Population	X
14.3.3.1.2	Overall Summary of Treatment Emergent Adverse Events, Disease Cohorts	Safety Population	X
14.3.3.2.1	Treatment Emergent Adverse Events by System Organ Class and Preferred	Safety Population	X

No.	Title	Analysis Population	Include in interim analysis?
	Term in Descending Order of Frequency, Healthy Volunteer Cohorts		
14.3.3.2.2	Treatment Emergent Adverse Events by System Organ Class and Preferred Term in Descending Order of Frequency, Disease Cohorts	Safety Population	X
14.3.3.3.1	Treatment Emergent Adverse Events by System Organ Class and Preferred Term in Decreasing Order of Frequency, Cohorts Pooled by Dose	Safety Population	X
14.3.3.4.1	Treatment Emergent Adverse Events in Descending Order of Frequency by Preferred Term, Healthy Volunteer Cohorts	Safety Population	X
14.3.3.4.2	Treatment Emergent Adverse Events in Descending Order of Frequency by Preferred Term, Disease Cohorts	Safety Population	X
14.3.3.5.1	Related Treatment Emergent Adverse Events by System Organ Class and Preferred Term in Descending Order of Frequency, Healthy Volunteer Cohorts	Safety Population	X
14.3.3.5.2	Related Treatment Emergent Adverse Events by System Organ Class and Preferred Term in Descending Order of Frequency, Disease Cohorts	Safety Population	X
14.3.3.6.1	Treatment Emergent Adverse Events by System Organ Class and Preferred Term and by Severity, Healthy Volunteer Cohorts	Safety Population	X
14.3.3.6.2	Treatment Emergent Adverse Events by System Organ Class and Preferred Term and by Severity, Disease Cohorts	Safety Population	X
14.3.3.7.1	Treatment Emergent Adverse Events by System Organ Class and Preferred Term and by Relationship to Study Drug Healthy Volunteer Cohorts	Safety Population	X
14.3.3.7.2	Treatment Emergent Adverse Events by System Organ Class and Preferred Term and by Relationship to Study Drug, Disease Cohorts	Safety Population	X

No.	Title	Analysis Population	Include in interim analysis?
14.3.3.8.1	Serious Treatment Emergent Adverse Events by System Organ Class and Preferred Term in Descending Order of Frequency, Healthy Volunteer Cohorts	Safety Population	X
14.3.3.8.2	Serious Treatment Emergent Adverse Events by System Organ Class and Preferred Term in Descending Order of Frequency, Disease Cohorts	Safety Population	X
14.3.3.9.1	Serious Study Drug Related Treatment Emergent Adverse Events by System Organ Class and Preferred Term, Healthy Volunteer Cohorts	Safety Population	X
14.3.3.9.2	Serious Study Drug Related Treatment Emergent Adverse Events by System Organ Class and Preferred Term, Disease Cohorts	Safety Population	X
14.3.3.10.1	Treatment Emergent Adverse Events Leading to Study Drug or Study Withdrawal, Healthy Volunteer Cohorts	Safety Population	X
14.3.3.10.2	Treatment Emergent Adverse Events Leading to Study Drug or Study Withdrawal, Disease Cohorts	Safety Population	X
14.3.3.11.1	Treatment Emergent Adverse Events Occurring in >1 Study Participant in Descending Order of Frequency by Preferred Term, Healthy Volunteer Cohorts	Safety Population	X
14.3.3.11.2	Treatment Emergent Adverse Events Occurring in >1 Study Participant in Descending Order of Frequency by Preferred Term, Disease Cohorts	Safety Population	X
14.3.3.12.1	Injections Leading to Adverse Events at the Injection Site by System Organ Class and Preferred Term, Healthy Volunteer Cohorts	Safety Population	X
14.3.3.12.2	Injections Leading to Adverse Events at the Injection Site by System Organ Class and Preferred Term, Disease Cohorts	Safety Population	X
14.3.3.13.1	Injections Leading to Adverse Events at the Injection Site by System Organ	Safety Population	X

No.	Title	Analysis Population	Include in interim analysis?
	Class and Preferred Term and by Severity, Healthy Volunteer Cohorts		
14.3.3.13.2	Injections Leading to Adverse Events at the Injection Site by System Organ Class and Preferred Term and by Severity, Disease Cohorts	Safety Population	X
14.3.3.14.1	Treatment Emergent Adverse Events Related to Local Injection Site Reactions	Safety Population	X
14.3.3.14.2	Treatment Emergent Adverse Events Related to Local Injection Site Reactions	Safety Population	X
14.3.3.15.1	Treatment Emergent Adverse Events Related to Local Injection Site Reactions	Safety Population	X
14.3.3.15.2	Treatment Emergent Adverse Events Related to Local Injection Site Reactions	Safety Population	X
14.3.3.16.1	Treatment Emergent Adverse Events Leading to Death by System Organ Class and Preferred Term, Healthy Volunteer Cohorts	Safety Population	X
14.3.3.16.2	Treatment Emergent Adverse Events Leading to Death by System Organ Class and Preferred Term, Disease Cohorts	Safety Population	X
14.3.4.1.1	Laboratory - Hematology: Summary and Change from Baseline, Healthy Volunteer Cohorts,	Safety Population	X
14.3.4.1.2	Laboratory - Hematology: Summary and Change from Baseline, Disease Cohorts	Safety Population	X
14.3.4.1.3	Laboratory - Haematology for extension Cohort 9	Safety Population	X
14.3.4.2.1	Laboratory - Chemistry: Summary and Change from Baseline, Healthy Volunteer Cohorts	Safety Population	X
14.3.4.2.2	Laboratory - Chemistry: Summary and Change from Baseline, Disease Cohorts	Safety Population	X
14.3.4.2.3	Laboratory - Chemistry for extension Cohort 9	Safety Population	X

No.	Title	Analysis Population	Include in interim analysis?
14.3.4.3.1	Laboratory - Coagulation: Summary and Change from Baseline, Healthy Volunteer Cohorts	Safety Population	X
14.3.4.3.2	Laboratory - Coagulation: Summary and Change from Baseline, Disease Cohorts	Safety Population	X
14.3.4.3.3	Laboratory - Coagulation for extension Cohort 9	Safety Population	X
14.3.4.4.1	Laboratory - Urinalysis: Summary and Change from Baseline, Healthy Volunteer Cohorts	Safety Population	X
14.3.4.4.2	Laboratory - Urinalysis: Summary and Change from Baseline, Disease Cohorts	Safety Population	X
14.3.4.5.1	Haematology Shifts from Baseline, Healthy Volunteer Cohorts	Safety Population	X
14.3.4.5.2	Haematology Shifts from Baseline, Disease Cohorts	Safety Population	X
14.3.4.6.1	Biochemistry Shifts from Baseline, Healthy Volunteer Cohorts	Safety Population	X
14.3.4.6.2	Biochemistry Shifts from Baseline, Disease Cohorts	Safety Population	X
14.3.4.7.1	Coagulation Shifts from Baseline, Healthy Volunteer Cohorts	Safety Population	X
14.3.4.7.2	Coagulation Shifts from Baseline, Disease Cohorts	Safety Population	X
14.3.4.8.1	Shift from Baseline Grade to Worst Post-Baseline Grade, Healthy Volunteer Cohorts	Safety Population	X
14.3.4.8.2	Shift from Baseline Grade to Worst Post-Baseline Grade, Disease Cohorts	Safety Population	X
**14.3.5/6/7**	**Other Safety**		
14.3.5.1	Vital Signs, Healthy Volunteer Cohorts	Safety Population	
14.3.5.2	Vital Signs, Disease Cohorts	Safety Population	
14.3.5.3	Vital Signs for extension Cohort 9	Safety Population	

No.	Title	Analysis Population	Include in interim analysis?
14.3.6.1	Physical Examination, Healthy Volunteer Cohorts	Safety Population	
14.3.6.2	Physical Examination, Disease Cohorts	Safety Population	
14.3.7.1.1	ECG Values, Healthy Volunteer Cohorts	Safety Population	
14.3.7.1.2	ECG Values, Disease Cohorts	Safety Population	
14.3.7.2.1	ECG Findings, Overall Interpretation, Healthy Volunteer Cohorts	Safety Population	
14.3.7.2.2	ECG Findings, Overall Interpretation, Disease Cohorts	Safety Population	

Table to be produced with US Units and SI units separately (*x = 1 for SI units and x = 2 for US units*).

17. LISTINGS

As indicated in the table of listings below, listings will be produced separately for healthy volunteer cohorts (Cohorts 1, 2, 3, 4, 5, 2b, 3b, and 4b), and Patient Cohorts (Cohorts 6, 7, 7b, 7c, 8 and 9).

All listings that relate to parameters summarized in the baseline disease characteristics tables (will be produced twice, once considering SI units and once considering US units. The numbering convention used for these additional US unit listings will be the same as that used for the tables, as stated in section 16.

No.	Title	Analysis Population	Include in interim analysis?
**16.2.1**	**Participant Disposition**		
16.2.1.1.1	Listing of Enrolment, Healthy Volunteer Cohorts	Safety Population	X
16.2.1.1.2	Listing of Enrolment, Disease Cohorts	Safety Population	X
16.2.1.2.1	Listing of Screen Failures, Healthy Volunteer Cohorts	Screening Population	X
16.2.1.2.2	Listing of Screen Failures, Disease Cohorts	Screening Population	X
16.2.1.3.1	Listing of Patient Eligibility, Healthy Volunteer Cohorts	Safety Population	X
16.2.1.3.2	Listing of Patient Eligibility, Disease Cohorts	Safety Population	X
16.2.2.1.1	Listing of Protocol Deviations, Healthy Volunteer Cohorts	Safety Population	X
16.2.2.1.2	Listing of Protocol Deviations, Disease Cohorts	Safety Population	X
16.2.3.1.1	Listing of Disposition, Healthy Volunteer Cohorts	Safety Population	X
16.2.3.1.2	Listing of Disposition, Disease Cohorts	Safety Population	X
16.2.3.2.1	Listing of Randomization, Healthy Volunteer Cohorts	Safety Population	X
16.2.3.2.2	Listing of Randomization, Disease Cohorts	Safety Population	X
**16.2.4**	**Demographic and Other Baseline Data**		
16.2.4.1.1	Listing of Demographics, Healthy Volunteer Cohorts	Safety Population	X
16.2.4.1.2	Listing of Demographics, Disease Cohorts	Safety Population	X
16.2.4.2.1	Listing of Medical History, Healthy Volunteer Cohorts	Safety Population	X
16.2.4.2.2	Listing of Medical History, Disease Cohorts	Safety Population	X
16.2.4.3.1	Listing of Follicle-Stimulating Hormone (FSH) Results, Healthy Volunteer Cohorts	Safety Population	X
16.2.4.3.2	Listing of Follicle-Stimulating Hormone (FSH) Results, Disease Cohorts	Safety Population	X
16.2.4.4.1	Listing of Child-bearing Potential and Pregnancy Test Results, Healthy Volunteer Cohorts	Safety Population	X

No.	Title	Analysis Population	Include in interim analysis?
16.2.4.4.2	Listing of Child-bearing Potential and Pregnancy Test Results, Disease Cohorts	Safety Population	X
16.2.4.5.1	Listing of Serology Screen, Healthy Volunteer Cohorts	Safety Population	X
16.2.4.5.2	Listing of Serology Screen, Disease Cohorts	Safety Population	X
16.2.4.6.1	Listing of Drug Screen, Healthy Volunteer Cohorts	Safety Population	X
16.2.4.6.2	Listing of Drug Screen, Disease Cohorts	Safety Population	X
16.2.4.7.1	Listing of Alcohol Screen, Healthy Volunteer Cohorts	Safety Population	X
16.2.4.7.2	Listing of Alcohol Screen, Disease Cohorts	Safety Population	X
16.2.4.8.1	Listing of Lipid Metabolic Genotype Test Results, Healthy Volunteer Cohorts	Safety Population	X
16.2.4.8.2	Listing of Lipid Metabolic Genotype Tests, Disease Cohorts	Safety Population	X
16.2.4.9.1	Listing of Log of Lipid Collection, Healthy Volunteer Cohorts	Safety Population	X
16.2.4.9.2	Listing of Log of Lipid Collection, Disease Cohorts	Safety Population	X
16.2.4.10.x.1	Listing of Baseline Disease Characteristics, Healthy Volunteer Cohorts	Safety Population	X
16.2.4.10.x.2	Listing of Baseline Disease Characteristics, Disease Cohorts	Safety Population	X
16.2.4.11.1	Listing of Genes Tested for Mutations Associated with Dyslipidemia, Healthy volunteer Cohorts	Safety Population	X
16.2.4.11.2	Listing of Genes Tested for Mutations Associated with Dyslipidemia, Disease Cohorts	Safety Population	X
**16.2.5**	**Treatment Administration**		
16.2.5.1.1	Listing of Study Drug Administrations, Healthy Volunteer Cohorts	Safety Population	X
16.2.5.1.2	Listing of Study Drug Administrations, Disease Cohorts	Safety Population	X
16.2.5.2.1	Listing of Concomitant/Prior Medications and Non-Drug Therapies, Healthy Volunteer Cohorts	Safety Population	X
16.2.5.2.2	Listing of Concomitant/Prior Medications and Non-Drug Therapies, Disease Cohorts	Safety Population	X
**16.2.6**	**PK/PD**		
16.2.6.1.1	Listing of Individual ARO-ANG3 Plasma Concentrations, Single Dosed Healthy Volunteer Cohorts	Pharmacokinetic Population	

No.	Title	Analysis Population	Include in interim analysis?
16.2.6.2.1	Listing of Individual ARO-ANG3 Plasma Concentrations, Multiple Dosed Healthy Volunteer Cohorts	Pharmacokinetic Population	
16.2.6.3.1	Listing of Individual ARO-ANG3 Urine Concentration (ng/mL), Healthy Volunteer Cohorts	Pharmacokinetic Population	
16.2.6.4.1	Listing of Individual ARO-ANG3 Plasma Pharmacokinetic Parameters, Healthy Volunteer Cohorts	Pharmacokinetic Population	
16.2.6.5.1	Listing of Individual ARO-ANG3 Urine Pharmacokinetic Parameters, Healthy Volunteer Cohorts	Pharmacokinetic Population	
16.2.6.6.2	Listing of Anti-drug Antibodies, Disease Cohorts	Safety Population	X
16.2.6.7.1	Listing of Glucose Tolerance Test (GTT) Adherence, Healthy Volunteer Cohorts	Pharmacodynamic Population	X
16.2.6.7.2	Listing of Glucose Tolerance Test (GTT) Adherence, Disease Cohorts	Pharmacodynamic Population	X
16.2.6.8.1	Listing of Glucose Tolerance Test (GTT) and Change Between Baseline and Day 85 Values, Healthy Volunteer Cohorts	Pharmacodynamic Population	X
16.2.6.8.2	Listing of Glucose Tolerance Test (GTT) and Change Between Baseline and Day 85 Values, Healthy Volunteer Cohorts	Pharmacodynamic Population	X
16.2.6.9.1	Listing of Post-Prandial Triglyceride (TG) Adherence, Healthy Volunteer Cohorts	Pharmacodynamic Population	X
16.2.6.9.2	Listing of Post-Prandial Triglyceride (TG) Adherence, Disease Cohorts	Pharmacodynamic Population	X
16.2.6.10.1	Listing of Post-Prandial Triglyceride (TG) Test Results and Change Between Baseline and Day 85 Values, Healthy Volunteer Cohorts	Pharmacodynamic Population	X
16.2.6.10.2	Listing of post-prandial triglyceride (TG) Test results and Change Between Baseline and Day 85 Values, Disease Cohorts	Pharmacodynamic Population	X
16.2.6.11.x.1	Listing of Pharmacodynamic Parameters, Healthy Volunteer Cohorts	Safety Population	X
16.2.6.11.x.2	Listing of Pharmacodynamic Parameters, Disease Cohorts	Safety Population	X
16.2.6.12.1	Listing of Serum Insulin, Healthy Volunteer Cohorts	Safety Population	X

No.	Title	Analysis Population	Include in interim analysis?
16.2.6.12.2	Listing of Serum Insulin, Disease Cohorts	Safety Population	X
16.2.6.13.2	Listing of Percentage Liver Fat Fraction on MRI-PDFF	Safety Population	X
**16.2.7**	**Adverse Events**		
16.2.7.1.1	Listing of Adverse Events, Healthy Volunteer Cohorts	Safety Population	X
16.2.7.1.2	Listing of Adverse Events, Disease Cohorts	Safety Population	X
16.2.7.2.1	Listing of Serious Adverse Events, Healthy Volunteer Cohorts	Safety Population	X
16.2.7.2.2	Listing of Serious Adverse Events, Disease Cohorts	Safety Population	X
16.2.7.3.1	Listing of Adverse Events Leading to Study Drug Withdrawn or Study Withdrawn, Healthy Volunteer Cohorts	Safety Population	X
16.2.7.3.2	Listing of Adverse Events Leading to Study Drug Withdrawn or Study Withdrawn, Disease Cohorts	Safety Population	X
16.2.7.4.1	Listing of Adverse Events at the Injection Site, Healthy Volunteer Cohorts	Safety Population	X
16.2.7.4.2	Listing of Adverse Events at Injection Site, Disease Cohorts	Safety Population	X
16.2.7.5.1	Listing of Adverse Events Related to Local Injection Site Reactions, Healthy Volunteer Cohorts	Safety Population	X
16.2.7.5.2	Listing of Local Injection Site Reactions, Disease Cohorts	Safety Population	X
16.2.7.6.1	Listing of Adverse Events Leading to Death, Healthy Volunteer Cohorts	Safety Population	X
16.2.7.6.2	Listing of Adverse Events Leading to Death, Disease Cohorts	Safety Population	X
**16.2.8**	**Laboratory Parameters**		
16.2.8.1.y.1	Listing of Hematology – Central Laboratory, Healthy Volunteer Cohorts	Safety Population	X
16.2.8.1.y.2	Listing of Hematology – Central Laboratory, Disease Cohorts	Safety Population	X

No.	Title	Analysis Population	Include in interim analysis?
16.2.8.2.y.1	Listing of Hematology – Central Laboratory, Clinically Significant Values, Healthy Volunteer Cohorts	Safety Population	X
16.2.8.2.y.2	Listing of Hematology – Central Laboratory, Clinically Significant Values, Disease Cohorts	Safety Population	X
16.2.8.3.y.1	Listing of Biochemistry – Central Laboratory, Healthy Volunteer Cohorts	Safety Population	X
16.2.8.3.y.2	Listing of Biochemistry – Central Laboratory, Disease Cohorts	Safety Population	X
16.2.8.4.y.1	Listing of Biochemistry – Central Laboratory, Clinically Significant Values, Healthy Volunteer Cohorts	Safety Population	X
16.2.8.4.y.2	Listing of Biochemistry – Central Laboratory, Clinically Significant Values, Disease Cohorts	Safety Population	X
16.2.8.5.y.1	Listing of Coagulation – Central Laboratory, Healthy Volunteer Cohorts	Safety Population	X
16.2.8.5.y.2	Listing of Coagulation – Central Laboratory, Disease Cohorts	Safety Population	X
16.2.8.6.y.1	Listing of Coagulation – Central Laboratory, Clinically Significant Values, Healthy Volunteer Cohorts	Safety Population	X
16.2.8.6.y.2	Listing of Coagulation – Central Laboratory, Clinically Significant Values, Disease Cohorts	Safety Population	X
16.2.8.7.y.1	Listing of Urinalysis – Central Laboratory, Healthy Volunteer Cohorts	Safety Population	X
16.2.8.7.y.2	Listing of Urinalysis – Central Laboratory, Disease Cohorts	Safety Population	X
16.2.8.8.y.1	Listing of Urinalysis – Central Laboratory, Clinically Significant Values, Healthy Volunteer Cohorts	Safety Population	X
16.2.8.8.y.2	Listing of Urinalysis – Central Laboratory, Clinically Significant Values, Disease Cohorts	Safety Population	X
16.2.8.9.y.1	Listing of Microscopic Urinalysis – Central Laboratory, Healthy Volunteer Cohorts	Safety Population	X

No.	Title	Analysis Population	Include in interim analysis?
16.2.8.9.y.2	Listing of Microscopic Urinalysis – Central Laboratory, Disease Cohorts	Safety Population	X
16.2.8.10.y.1	Listing of Microscopic Urinalysis – Central Laboratory, Clinically Significant Values, Healthy Volunteer Cohorts	Safety Population	X
16.2.8.10.y.2	Listing of Microscopic Urinalysis – Central Laboratory, Clinically Significant Values, Disease Cohorts	Safety Population	X
**16.2.9/10/11**	**Other Safety**		
16.2.9.1	Listing of Vital Signs, Healthy Volunteer Cohorts	Safety Population	X
16.2.9.2	Listing of Vital Signs, Disease Cohorts	Safety Population	X
16.2.10.1	Listing of Physical Examination, Healthy Volunteer Cohorts	Safety Population	X
16.2.10.2	Listing of Physical Examination, Disease Cohorts	Safety Population	X
16.2.11.1.1	Listing of ECG Values, Healthy Volunteer Cohorts	Safety Population	X
16.2.11.1.2	Listing of ECG Values, Disease Cohorts	Safety Population	X
16.2.11.2.1	Listing of ECG Clinically Significant Values, Healthy Volunteer Cohorts	Safety Population	X
16.2.11.2.2	Listing of ECG Clinically Significant Values, Disease Cohorts	Safety Population	X
16.2.11.3.1	Listing of ECG Findings, Disease Cohorts	Safety Population	X
16.2.11.3.2	Listing of ECG Findings, Disease Cohorts	Safety Population	X

Listing to be produced with US Units and SI units separately (x = 1 for SI units and x = 2 for US units)
Listing to be produced for central and local laboratories separately (*y = 1 for Central and y = 2 for Local laboratories*).

18. FIGURES

PK Figures will be produced for HV cohorts 1, 2, 3, 4, 2b, 3b and 4b. PD and Safety figures will be produced for all cohorts.
All figures that relate to parameters summarized in the baseline disease characteristics tables will be produced twice, once considering SI units and once considering US units. The numbering convention used for these additional US unit figures will be the same as that used for the tables, as stated in section 16.

No.	Title	Analysis Population	Include in interim analysis?
14.2.1.1.1a	Mean (+/-SD) Plasma Concentrations of ARO-ANG3 for healthy volunteers versus Time by Dose (Linear Scale): SAD	PK	
14.2.1.1.1b	Mean (+/-SD) Plasma Concentrations of ARO-ANG3 for healthy volunteers versus Time by Dose (Semi-logarithmic Scale): SAD	PK	
14.2.1.2.1a	Mean (+/-SD) Plasma Concentrations of ARO-ANG3 for healthy volunteers versus Time by Dose (Linear Scale): MAD Day 1	PK	
14.2.1.2.1b	Mean (+/-SD) Plasma Concentrations of ARO-ANG3 for healthy volunteers versus Time by Dose (Semi-logarithmic Scale): MAD Day 1	PK	
14.2.1.3.1a	Mean (+/-SD) Plasma Concentrations of ARO-ANG3 for healthy volunteers versus Time by Dose (Linear Scale): MAD Day 29	PK	
14.2.1.3.1b	Mean (+/-SD) Plasma Concentrations of ARO-ANG3 for healthy volunteers versus Time by Dose (Semi-logarithmic Scale): MAD Day 29	PK	
14.2.1.4.1a	Mean (+/-SD) Plasma Concentrations of ARO-ANG3 for healthy volunteers versus Time by Dose (Linear Scale): SAD and MAD Day 1	PK	
14.2.1.4.1b	Mean (+/-SD) Plasma Concentrations of ARO-ANG3 for healthy volunteers versus Time by Dose (Semi-logarithmic Scale): SAD and MAD Day 1	PK	
14.2.2.1.1a	Spaghetti Plot of Plasma Concentrations of ARO-ANG3 for healthy volunteers versus Time by Treatment (Linear Scale): SAD	PK	
14.2.2.1.1b	Spaghetti Plot of Plasma Concentrations of ARO-ANG3 for healthy volunteers versus Time by Treatment (Semilogarithmic Scale) : SAD	PK	
14.2.2.2.1a	Spaghetti Plot of Plasma Concentrations of ARO-ANG3 for healthy volunteers versus Time by Treatment (Linear Scale): MAD Day 1	PK	
14.2.2.2.1b	Spaghetti Plot of Plasma Concentrations of ARO-ANG3 for healthy volunteers versus Time by Treatment (Semilogarithmic Scale) : MAD Day 1	PK	
14.2.2.3.1a	Spaghetti Plot of Plasma Concentrations of ARO-ANG3 for healthy volunteers versus Time by Treatment (Linear Scale): MAD Day 29	PK	
14.2.2.3.1b	Spaghetti Plot of Plasma Concentrations of ARO-ANG3 for healthy volunteers versus Time by	PK	

No.	Title	Analysis Population	Include in interim analysis?
	Treatment (Semilogarithmic Scale) : MAD Day 29		
14.2.3.1.1a	Individual Plasma Concentrations of ARO-ANG3 for healthy volunteers versus Time (Linear Scale): SAD	PK	
14.2.3.1.1b	Individual Plasma Concentrations of ARO-ANG3 for healthy volunteers versus Time (Semi-logarithmic Scale): SAD	PK	
14.2.3.2.1a	Individual Plasma Concentrations of ARO-ANG3 for healthy volunteers versus Time (Linear Scale) : MAD Day 1	PK	
14.2.3.2.1b	Individual Plasma Concentrations of ARO-ANG3 for healthy volunteers versus Time (Semi-logarithmic Scale): MAD Day 1	PK	
14.2.3.3.1a	Individual Plasma Concentrations of ARO-ANG3 for healthy volunteers versus Time (Linear Scale): MAD Day 29	PK	
14.2.3.3.1b	Individual Plasma Concentrations of ARO-ANG3 for healthy volunteers versus Time (Semi-logarithmic Scale): MAD Day 29	PK	
14.2.4.1.1	Linear Regression Plot of ARO-ANG3 Parameters for healthy volunteers: SAD	PK	
14.2.4.2.1	Linear Regression Plot of ARO-ANG3 Parameters for healthy volunteers: MAD Day 1	PK	
14.2.4.3.1	Linear Regression Plot of ARO-ANG3 Parameters for healthy volunteers: SAD and MAD Day 29	PK	
14.2.4.4.1	Linear Regression Plot of ARO-ANG3 Parameters for healthy volunteers: SAD and MAD Day 1	PK	
14.2.5.1.1	Box Plots of Dose Normalized Plasma ARO-ANG3 Parameters for healthy volunteers by Gender: SAD.	PK	
14.2.5.2.1	Box Plots of Dose Normalized Plasma ARO-ANG3 Parameters for healthy volunteers by Gender: MAD Day 1	PK	
14.2.5.3.1	Box Plots of Dose Normalized Plasma ARO-ANG3 Parameters for healthy volunteers by Gender: MAD Day 29	PK	
14.2.5.4.1	Box Plots of Dose Normalized Plasma ARO-ANG3 Parameters for healthy volunteers by Gender: MAD and SAD Day 1	PK	
14.3.1.1.x.1	Mean (+/-SD) Plasma Concentrations of Triglycerides versus Time by Dose	PD	X
14.3.1.2.x.1	Mean (+/-SD) Plasma Concentrations of LDL-C versus Time by Dose	PD	X
14.3.1.3.x.1	Mean (+/-SD) Plasma Concentrations of HDL-C versus Time by Dose	PD	X
14.3.1.4.x.1	Mean (+/-SD) Plasma Concentrations of non-HDL versus Time by Dose	PD	
14.3.1.5.x.1	Mean (+/-SD) Plasma Concentrations of ANGPTL3 versus Time by Dose	PD	X

No.	Title	Analysis Population	Include in interim analysis?
14.3.1.6.x.1	Mean (+/-SD) Percentage Reduction from Baseline in Plasma Concentrations of Triglycerides versus Time by Dose	PD	
14.3.1.7.x.1	Mean (+/-SD) Percentage Increase from Baseline in Plasma Concentrations of HDL-C versus Time by Dose	PD	
14.3.1.8.x.1	Mean (+/-SD) Percentage Reduction from Baseline in Plasma Concentrations of non-HDL-C versus Time by Dose	PD	
14.3.1.9.x.1	Mean (+/-SD) Percentage Reduction from Baseline in Plasma Concentrations of ARO-ANG versus Time by Dose	PD	
14.4.1.1.1	eDISH plot for healthy volunteer cohorts (active and placebo) for peak post-dose Total bilirubin vs ALT	Safety	X
14.4.1.1.2	eDISH plot for disease cohorts (active v placebo) EOS Total bilirubin vs ALT	Safety	X
14.4.1.2.1	eDISH plot for healthy volunteer cohorts (active and placebo) EOS Total bilirubin vs ALT	Safety	X
14.4.1.2.2	eDISH plot for disease cohorts (active and placebo) for peak post-dose Total bilirubin vs ALT.	Safety	X
14.4.2.1	Mean ALT over time, active and placebo cohorts	Safety	X
14.4.2.2	Mean AST over time, active and placebo cohorts	Safety	X
14.4.2.3	Mean Creatinine over time, active and placebo cohorts	Safety	X
14.4.2.4	Mean Lipase over time, active and placebo cohorts	Safety	X
14.4.2.5	Mean Total bilirubin over time, active and placebo cohorts	Safety	X

Figures to be produced with US Units and SI units separately (x = 1 for SI units and x = 2 for US units)

19. APPENDIX A NONCOMPARTMENTAL PHARMACOKINETIC ANALYSIS

19.1 Handling Missing or Non-Quantifiable Data

For Noncompartmental analysis (NCA), plasma concentrations below the limit of quantification (BLQ) will be assigned a value of 0. The following rules apply with special situations defined below:

- If an entire concentration-time profile is BLQ, it will be excluded from PK analysis.
- Where 2 or more consecutive concentrations are BLQ at the end of a profile, the profile will be deemed to have terminated and any further quantifiable concentrations will be set to missing for the calculation of the PK parameters, unless they are considered to be a true characteristic of the profile of the drug.
- If a predose plasma concentration is missing, it may be set to zero by default, for first dose only.
- If an embedded BLQ value is considered anomalous within the concentration time profile, this value will be set as missing and excluded from the summary statistics

For Urine PK parameters calculation and summary, all BLQ will be set to zero.

19.2 Pharmacokinetic Parameter Calculation

Standard PK parameters will be determined, where possible, from the plasma and urine concentrations of ARO-ANG3 using noncompartmental methods (NCA) in validated software program Phoenix WinNonlin (Certara USA, Inc. version 8.1 or higher).

Pharmacokinetic analysis will be carried out where possible using actual blood sampling times postdose. If an actual time is missing, the sample concentration result will be treated as missing unless there is scientific justification to include the result using the nominal time. Cmax and tmax will be obtained directly from the concentration-time profiles. For multiple peaks, the highest postdose concentration will be reported as Cmax. In the case that multiple peaks are of equal magnitude, the earliest tmax will be reported.
AUC will be estimated using the linear trapezoidal rule for increasing concentrations and the log-trapezoidal rule for decreasing concentrations (i.e., "linear up / log down" trapezoidal rule in Phoenix WinNonlin). The total AUCinf was calculated as AUClast + Ct/λz. The apparent clearance (CL/F) was estimated as Dose/AUCinf, and the terminal-phase volume of distribution (Vz/F) was estimated as Dose/[AUCinf*λz].

19.2.1 Criteria for the Calculation of Apparent Terminal Elimination Rate Constant and Half-life

The start of the terminal elimination phase for each subject will be defined by visual inspection and generally will be the first point at which there is no systematic deviation from the log-linear decline in concentrations.

The apparent terminal elimination rate constant (λz) will only be calculated when a reliable estimate can be obtained using at least 3 data points, not including Cmax, and the adjusted coefficient for determination of exponential fit (R2-adj) of the regression line is ≥0.75. Parameters requiring λz for their calculation (eg, AUCinf, t1/2, CL, and Vz) will only be calculated if the R2-adj value of the regression line is ≥0.75.

The following regression-related diagnostic PK parameters will be determined, when possible.

Parameter	Units	Definition
λz	1/h	apparent terminal elimination rate constant
λz N	NA	number of data points included in the log-linear regression
λz Span Ratio	NA	time period over which λz was determined as a ratio of t1/2
R2-adj	NA	adjusted coefficient for determination of exponential fit

Where possible, the span of time used in the determination of λz (ie, the difference between λz Upper and λz Lower) should be ≥2 half-lives. If the λz Span Ratio is <2, the robustness of the t1/2 values will be discussed in the PK report.

19.2.2 Criteria for Calculation and Reporting of Area Under the Concentration-time Curve

The minimum requirement for the calculation of area under the concentration-time curve (AUC) will be the inclusion of at least 3 consecutive concentrations above the lower limit of quantification. If there are only 3 consecutive concentrations, at least 1 should follow Cmax.

If the extrapolated area is >20%, AUCinf (and derived parameters) will be flagged and excluded from the summary statistics and statistical analysis.

19.3 Treatment of Outliers in Pharmacokinetic Analysis

If a value is considered to be anomalous due to being inconsistent with the expected PK profile, it may be appropriate to exclude the value from the PK analysis. However, the exclusion of any data must have strong justification and will be documented in the CSR.

Any quantifiable predose concentration value before the first dose will be considered anomalous and set to missing for the PK analysis.

20. APPENDIX B EARLY TERMINATION ANALYSIS WINDOWS

The early termination visit value will be mapped to the closest scheduled visit within the specified analysis windows below:

Visit	Analysis Window (Study Day)
Day 1	[1, 2)
Day 3	[2, 6)
Day 8	[6, 12)
Day 15	[12, 19)
Day 22	[19, 26)
Day 29	[26, 36)
Day 43	[36, 50)
Day 57	[50, 64)
Day 71	[64, 78)
Day 85	[78, 92)
Day 99	[92, 106)
Day 113	[106, EOS]

21. REFERENCES

1) Smith BP, Vandenhende FR, DeSante KA, et al. Confidence interval criteria for assessment of dose proportionality. Pharm Res. 2000;17(10): 1278–1283.
