# Supplementary material for: RNA interference targeting ANGPTL3 for triglyceride and cholesterol lowering: phase 1 basket trial cohorts
Source: Nat Med. 2023 Aug 25;29(9):2216–23. doi: 10.1038/s41591-023-02494-2 (PMC10504078; doi:10.1038/s41591-023-02494-2)
Supplement: Supplementary file 2 — Reporting Summary [file 41591_2023_2494_MOESM2_ESM.pdf]

nature portfolio

Corresponding author(s): Gerald Watts

Last updated by author(s): Jun 19, 2023

Reporting Summary

Nature Portfolio wishes to improve the reproducibility of the work that we publish. This form provides structure for consistency and transparency in reporting. For further information on Nature Portfolio policies, see our Editorial Policies and the Editorial Policy Checklist.

Statistics

For all statistical analyses, confirm that the following items are present in the figure legend, table legend, main text, or Methods section.

n/a	Confirmed	
☐	☒	The exact sample size (*n*) for each experimental group/condition, given as a discrete number and unit of measurement
☒	☐	A statement on whether measurements were taken from distinct samples or whether the same sample was measured repeatedly
☐	☒	The statistical test(s) used AND whether they are one- or two-sided<br>*Only common tests should be described solely by name; describe more complex techniques in the Methods section.*
☐	☒	A description of all covariates tested
☐	☒	A description of any assumptions or corrections, such as tests of normality and adjustment for multiple comparisons
☐	☒	A full description of the statistical parameters including central tendency (e.g. means) or other basic estimates (e.g. regression coefficient) AND variation (e.g. standard deviation) or associated estimates of uncertainty (e.g. confidence intervals)
☐	☒	For null hypothesis testing, the test statistic (e.g. *F*, *t*, *r*) with confidence intervals, effect sizes, degrees of freedom and *P* value noted<br>*Give P values as exact values whenever suitable.*
☒	☐	For Bayesian analysis, information on the choice of priors and Markov chain Monte Carlo settings
☒	☐	For hierarchical and complex designs, identification of the appropriate level for tests and full reporting of outcomes
☒	☐	Estimates of effect sizes (e.g. Cohen's *d*, Pearson's *r*), indicating how they were calculated

Our web collection on statistics for biologists contains articles on many of the points above.

Software and code

Policy information about availability of computer code

Data collection	Medidata EDC data collection
Data analysis	SAS version 8.2

For manuscripts utilizing custom algorithms or software that are central to the research but not yet described in published literature, software must be made available to editors and reviewers. We strongly encourage code deposition in a community repository (e.g. GitHub). See the Nature Portfolio guidelines for submitting code & software for further information.

Data

Policy information about availability of data

All manuscripts must include a data availability statement. This statement should provide the following information, where applicable:

- Accession codes, unique identifiers, or web links for publicly available datasets
- A description of any restrictions on data availability
- For clinical datasets or third party data, please ensure that the statement adheres to our policy

Arrowhead is committed to sharing anonymized data from our clinical trials without compromising the privacy of trial participants. Statistical Analysis Plan and Final Study Protocol (Original and Final) will be made available upon publication. Data requests may be sent by email to info@arrowheadpharma.com. Arrowhead is developing a process to fulfill requests for research proposals that demonstrate scientific merit. Analyses based on research proposals that demonstrate scientific

merit will be considered. The mechanism for which data will be made available will be determined on a case-by-case basis in view of the circumstances surrounding each request. Arrowhead intends to only share data once a trial has completed and the product/indication has been approved at least the US and EU.

Human research participants

Policy information about studies involving human research participants and Sex and Gender in Research.

Reporting on sex and gender	Sex and race were self reported by all participants.
Population characteristics	All cohorts: Eligible subjects were male or female volunteers 18 to 65 years of age. Participants were required to use 2 highly effective forms of contraception during the study and for 3 months following the last dose of ARO-ANG3. Participants had a BMI between 19.0 and 40.0 kg/m2, inclusive and on a stable diet for at least 4 weeks with no plans to significantly alter diet or BMI over course of study. Single ascending dose cohorts only: fasting serum TGs > 100 mg/dL (1.13 mmol/L) at Screening and fasting serum LDL C > 70 mg/dL (1.81 mmol/L) at Screening. Single dose and multiple ascending dose cohorts: AST and ALT < 1.5× ULN at Screening. Hepatic steatosis cohort: AST and ALT < 3× ULN at Screening, MRI PDFF indicating a liver fat content of ≥ 10% .
Recruitment	NHV and patient cohorts were recruited via internal patient database as well as local advertising. Any accompanying material provided to the participant, as well as any advertising or compensation given to the participant, was reviewed and approved in writing by the study's Independent Ethics Committees (IECs). Study conduct oversight was provided by the sponsor to ensure compliance with the protocol, all applicable regulatory and ethics requirement and ICH/GCP in order to minimize selection bias. Sponsor required 100% source data verification (SDV). All participant data was monitored to ensure all enrolled participant met enrollment criteria requirements, which may minimize selection bias. In addition, the protocol received Māori approval in New Zealand and was assessed as fulfilling the Māori review requirement as per Te Ara Tika – Guidelines for Maaori Research Ethics. This approval ensures access to the study in the Māori population.
Ethics oversight	The protocol, amendments, and informed consent forms were approved by Institutional Review Boards at each study site: Linear Clinical Research, Bellberry HREC; Auckland Clinical Studies, Middlemore Clinical Trials, Lipid and Diabetes Research Group Northern B HDEC; Royal Adelaide Hospital, Royal Prince Alfred Hospital, Central Adelaide Local Health Network (CALHN) HREC.

Note that full information on the approval of the study protocol must also be provided in the manuscript.

Field-specific reporting

Please select the one below that is the best fit for your research. If you are not sure, read the appropriate sections before making your selection.

☒ Life sciences ☐ Behavioural & social sciences ☐ Ecological, evolutionary & environmental sciences

For a reference copy of the document with all sections, see nature.com/documents/nr-reporting-summary-flat.pdf

Life sciences study design

All studies must disclose on these points even when the disclosure is negative.

Sample size	This study represents a proof of principle study, and as such no formal sample size calculation was performed.
Data exclusions	No data were excluded from the analysis
Replication	Double statistical programming was applied to produce the experimental findings. The data was prepared in standard format (SDTM), and the TLFs can be re-produced.
Randomization	Subjects in double-blind SAD and hepatic steatosis cohorts were allocated a unique randomization number, in accordance with the randomization schedule. In each cohort, the first two subjects (sentinels) were randomized separately to one active (ARO-ANG3) and one PBO. The remaining participants were subsequently assigned to either active or PBO treatment. The allocation of active treatment or PBO was performed using a block randomization algorithm.
Blinding	SAD and hepatic steatosis cohorts were conducted under a double blind. After completion of the double blind cohorts, the Sponsor was unblinded, but the PI and study participants remained blinded.

Reporting for specific materials, systems and methods

We require information from authors about some types of materials, experimental systems and methods used in many studies. Here, indicate whether each material, system or method listed is relevant to your study. If you are not sure if a list item applies to your research, read the appropriate section before selecting a response.

Materials & experimental systems

n/a	Involved in the study
☒	☐ Antibodies
☒	☐ Eukaryotic cell lines
☒	☐ Palaeontology and archaeology
☒	☐ Animals and other organisms
☐	☒ Clinical data
☒	☐ Dual use research of concern

Methods

n/a	Involved in the study
☒	☐ ChIP-seq
☒	☐ Flow cytometry
☒	☐ MRI-based neuroimaging

Clinical data

Policy information about clinical studies

All manuscripts should comply with the ICMJE guidelines for publication of clinical research and a completed CONSORT checklist must be included with all submissions.

Clinical trial registration	NCT03747224
Study protocol	Included in Supplementary Information
Data collection	This study was conducted at 6 clinical study sites in Australia and New Zealand. Subjects enrollment occurred between 04JAN2019 and 24FEB2020, with last participant completing on 17MAY2021. This study was conducted and data collected by trained study staff in compliance with study protocol, local regulation and ICH/GCP with sponsor oversight. Data were collected on source document (medical chart) then transferred to EDC by clinical coordinator.
Outcomes	For all endpoints, methods for assessments were standard for clinical studies and were predefined in detail in the approved study protocol, with a schedule of timing for when each assessment was to occur. All study sites were trained on the protocol and how to conduct assessments. The primary endpoints were the subject incidence of treatment-emergent adverse events (reported at patient visits and assessed by investigators), vital signs (systolic/diastolic blood pressure, temperature, heart rate, respiratory rate), electrocardiogram measurements, injection site reactions, clinical laboratory tests (serum chemistry [including hemoglobin A1C], hematology, coagulation, urinalysis, microscopic urinalysis [if indicated], serology, follicle stimulating hormone [FSH], drug and alcohol use, pregnancy, lipid parameters, serum insulin levels, serum glucose levels, and stool occult blood test), concomitant medications/therapy, and reasons for treatment discontinuation due to toxicity. Secondary endpoints included reduction in fasting serum ANGPTL3 from baseline, assessed by analysis of fasting serum ANGPTL3 concentration.
